# Accurate profiling of single-cell alternative transcript start sites by correcting RNA degradation

**DOI:** 10.1038/s41467-026-72298-8

**Published:** 2026-04-28

**Authors:** Zijie Xu, Zhen Zhou, Chao Tang, Yating Zhang, Bin Mao, Yiming Zhang, Li Chen, Dan Zhang, Junwei Song, Xiuran Zheng, Defu Liu, Huiying Wang, Zhifeng He, Tingfeng Chen, Jing-wen Lin, Lu Chen

**Affiliations:** 1 Division of Pulmonary and Critical Care Medicine, State Key Laboratory of Biotherapy, West China Hospital, Sichuan University, Chengdu, China; 2 Biosafety Laboratory of West China Hospital, Center for Biological and Translational Research, West China Hospital, Sichuan University, Chengdu, China; 3 Department of Laboratory Medicine, West China Second University Hospital. Key Laboratory of Birth Defects and Related Diseases of Women and Children, Ministry of Education, Sichuan University, Chengdu, China; 4 Institute of Basic Medicine, School of Basic Medical Sciences and Forensic Medicine, North Sichuan Medical College, Nanchong, Sichuan China

**Keywords:** Software, Bioinformatics, Non-small-cell lung cancer, Transcriptomics

## Abstract

The generation of transcript variants via alternative utilisation of transcription start sites (TSSs) is a pivotal regulatory mechanism in physiological and pathological states. Recent advancements in 5’ single-cell RNA sequencing (scRNA-seq) have enabled TSS analysis at the single-cell level. However, RNA degradation leads to non-uniform read coverage, posing a critical challenge that significantly compromises accurate TSS quantification of scRNA-seq data. To address RNA degradation and improve TSS quantification, we develop scATS (single-cell alternative transcription start site) to estimate RNA degradation at both isoform and sample levels, and provide TSS quantification with or without degradation correction. Application of scATS reveals dynamic and context-dependent regulation of TSSs in haematopoiesis and disease, providing additional information on TSS isoforms that aids cell clustering at a finer resolution. Furthermore, we establish a machine-learning pipeline, lung cancer relevance score (LRS), to identify TSSs associated with lung cancer. We analyse TSS isoforms of *CCR6*, *CCR2* and *RTKN2* in lung cancer cell lines and confirm that isoforms highly transcribed in lung cancer promote cell proliferation and migration. Combined, we present a robust tool to accurately quantify TSS by accounting for RNA degradation, a common issue that confounds transcript quantification, and experimentally demonstrate the important roles of TSS-mediated gene regulation in tumourigenesis.

## Abstract (web summary)

Alternative transcription start sites generate transcript diversity but are difficult to detect due to RNA degradation in 5’ scRNA-seq. Here, the authors present scATS, which corrects degradation to quantify TSS usage, improve cell clustering, and identify lung cancer-promoting TSS isoforms.

## Introduction

The 5’ untranslated region (UTR) plays a crucial role in regulating cellular metabolism through the intricate interplay of transcription factors (TFs)^1^, enhancers^2^, insulators^3^ and silencers^4^. The alternative utilisation of transcription start site (TSS) generates distinct 5’ ends of isoforms, thereby expanding transcriptional diversity and reshaping the cellular transcriptome through the production of multiple mRNA isoforms. Indeed, alternative transcription start site (ATS) serves as a pivotal mechanism to enhance transcriptional complexity across more than half of all genes in the mammalian genome^5^. For instance, the activation of Wnt signalling via distal initiation at the *Runx1* promoter was found to drive haematopoietic differentiation^6^. ATSs can also be exploited by pathogens, for example, the SARS-CoV-2 infection induced ATSs in the infected cells that facilitates chimeric RNA formation, potentially enhancing the viral immune evasion^7^. Pan-cancer analyses further reveal cancer-specific ATS patterns with prognostic significance, exemplified by proximal *TIMM13* promoter usage that promotes oncogenesis through increased protein expression^8^. Therefore, a comprehensive analysis of ATSs is essential for further elucidating gene regulation in both physiological and pathological contexts^9–13^.

Recent advancements in single-cell RNA sequencing (scRNA-seq) have revolutionised the analysis of both gene expression and TSSs at the single-cell level^14,15^. Specifically, the 10× Genomics 5’ scRNA-seq platform employs a template switch oligo-based enrichment to capture the 5’ end of RNA molecules^16^, thereby enabling TSS profiling alongside transcriptome analysis. However, accurate quantification of TSS profiles in scRNA-seq remains challenging, primarily due to the limited and fragile nature of single-cell RNA^17^, which is more susceptible to transcript-specific degradation than bulk RNA^18^. Furthermore, variations in RNA quality across samples result in incomplete capture of transcript 5’ ends, which may confound accurate identification and quantification of TSSs in scRNA-seq data^19^. This underscores the importance of incorporating robust quality control metrics at the sample level to identify and mitigate such biases. Despite these well-characterised technical challenges, the existing tools for single-cell TSS inference, including SCAFE^20^, CamoTSS^21^, scTSS^22^ and a method developed for bulk RNA-seq, TSSr^23^, lack robust strategies to quantify and correct the systematic biases introduced by RNA degradation.

Here, we introduce single-cell alternative transcription start site (scATS), a novel computational framework specifically designed to address the quantitative inaccuracies in single-cell TSS analysis caused by RNA degradation. Our method combines an empirical cumulative distribution function (ECDF) for precise and robust TSS identification with an expectation-maximisation (EM) algorithm to quantify RNA degradation, and subsequently corrects TSS transcription levels. scATS significantly outperforms existing methods in TSS identification and quantification on both simulated and real datasets. We applied scATS to analyse 5’ scRNA-seq datasets of mouse haematopoietic stem and progenitor cells (mHSPCs), peripheral blood mononuclear cells (PBMCs) from individuals recovered from COVID-19 and lung cancer cells, and revealed cell-type and disease-specific TSS usage that improve the resolution of cell clustering and elucidate fine-tuned transcriptional regulation during cell differentiation and tumourigenesis. Furthermore, we developed the lung cancer relevance score (LRS), a machine-learning model that harnesses 5’ scRNA-seq data to prioritise TSSs associated with lung cancer. Using the LRS model, we predicted and experimentally validated the roles of ATS-derived isoforms of *CCR6*, *CCR2*, and *RTKN2* in promoting tumourigenesis. Our study reveals a dynamic landscape of active ATSs that shapes the cellular transcriptome, providing a novel and robust computational framework to investigate the intricate heterogeneous transcriptional regulation in cell differentiation, infectious diseases and tumour biology.

## Results

### Overview of scATS

**Fig. 1:**
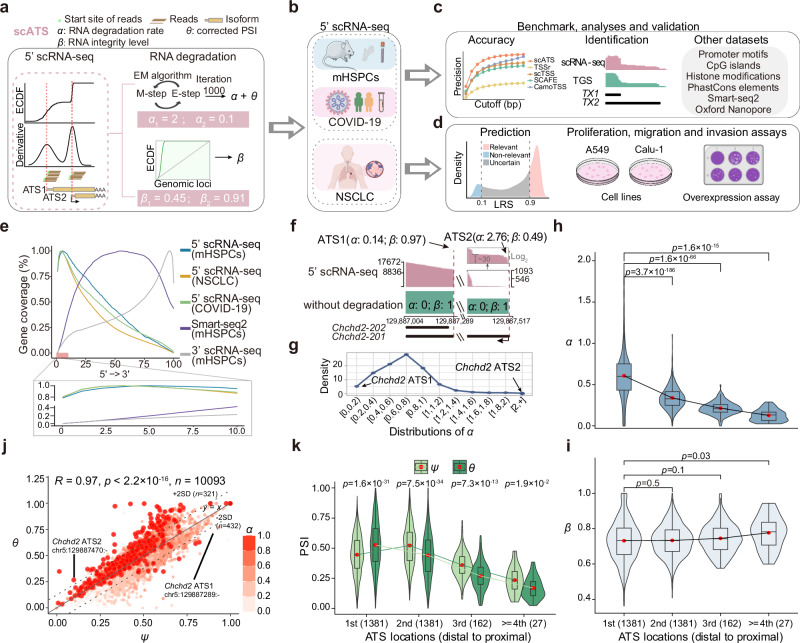
The scATS framework for identifying and quantifying TSSs with correction of RNA degradation. **a–d** scATS model construction and study design. **a** Identification of TSS (Transcription Start Site) and calculation of $\alpha$, based on expectation-maximisation (EM), and $\beta$, based on empirical cumulative distribution function (ECDF). scATS provides raw ($\psi$) and corrected ($\theta$) TSS usage. **b** Evaluation using mHSPCs (mouse haematopoietic stem and progenitor cells), COVID-19 PBMC (peripheral blood mononuclear cell), and NSCLC (non-small cell lung cancer) datasets. Thin mouse icon provided by Servier Medical Art (https://smart.servier.com), licensed under CC BY 3.0 Unported (https://creativecommons.org/licenses/by/3.0/). bone marrow icon created by El-Jayawant, licensed under CC BY 4.0 Unported (https://creativecommons.org/licenses/by/4.0/). COVID-19, people and blood sample icons adapted from our previous study^110^. NSCLC icon created in BioRender. Xu, Z. (2026) https://BioRender.com/2d4q2kf. **c** scATS benchmarking versus other methods. TGS, Third-generation sequencing; TX, transcript. **d** Functional TSSs via LRS (lung cancer relevance score) analysed in overexpression assays using lung cancer cell lines. **e** Read coverage from 5’ to 3’ across datasets, with first 10% of the gene body highlighted (lower panel). **f** Sashimi plots of *Chchd2* in mHSPC dataset. Predicted TSSs (red dashed lines), non-degraded states (green boxes), and GENCODE v25 annotations. **g** Density of RNA degradation ($\alpha$) for *Chchd2* across TSSs in mHSPC datasets, with arrows marking the loci of ATS1 and 2 from (**f**). **h**, **i** Violin plots showing RNA degradation metrics $\alpha$ (**h**) and $\beta$ (**i**) across different TSSs in genes with ATSs. **j** Correlation between raw ($\psi$) and corrected ($\theta$) PSI (percent of spliced-in) for all TSSs, with ATSs of *Chchd2* highlighted. Dashed lines: ±2 standard deviations (SD) from *y* = *x*. *R*, correlation coefficients; *n*, TSS number. **k**, Violin plots showing raw ($\psi$) and corrected ($\theta$) PSI values across different TSSs in genes with ATSs. For statistical analysis: two-sided Wilcoxon tests with BH (Benjamini–Hochberg) correction (**h**, **i**, **k**) and two-sided Pearson correlation test (**j**). Data in (**h**, **i**, **k**) shown as mean (red dots) and median (central line), boxes indicate interquartile range (IQR), whiskers ±1.5 × IQR. TSS numbers in parentheses. Exact *p*-values shown in figures.

We developed a computational method, scATS, to de novo infer the relative usage of TSSs and correct RNA degradation in paired-end 5’ scRNA-seq data. scATS consists of three main steps: (1) Identification of TSSs within each gene based on the ECDF of the start sites of read 1 (R1); (2) Quantification of RNA degradation using two metrics: an EM model-based metric ($\alpha$), and an ECDF-based metric ($\beta$); (3) Calculation of the TSS usage values (percent of spliced-in, PSI) before ($\psi$) and after degradation-correction ($\theta$) (Fig. 1a; see “Methods”).

We further applied scATS to two publicly available 5’ scRNA-seq datasets generated from clinical samples, including PBMCs from female patients recovered from COVID-19^24^ and cancer cells from a non-small cell lung cancer (NSCLC, 10× Genomics) patient, as well as an in-house 5’ scRNA-seq dataset generated from mouse HSPCs (mHSPCs) (Fig. 1b and Supplementary Data 1; see “Methods”). Benchmarking was performed using the mHSPC datasets. The accuracy of our method was evaluated by examining the enrichment of promoter motifs, epigenetic markers and evolutionary conservation, and further validated using the long-read RNA sequencing data (Fig. 1c). We also developed a machine-learning method, LRS model, to predict and prioritise the potentially relevant TSSs in NSCLC. To verify the accuracy of our prediction, we experimentally validated the top candidates using the overexpression assays (Fig. 1d).

### Widespread RNA degradation in 5’ scRNA-seq datasets

To assess the extent of RNA degradation in scRNA-seq, we used 5 datasets (Supplementary Data 1), including the three 5’ scRNA-seq datasets: the COVID-19^24^ and public NSCLC (10× Genomics) datasets, and an in-house mHSPC dataset. Additionally, we included an mHSPC Smart-seq2 dataset^25^ previously published by our group and generated an mHSPC 3’ scRNA-seq dataset for data validation (see “Methods”).

To investigate the degradation level, we first compared the transcript coverage evenness^26^ across the 5 datasets. As expected, 3’ scRNA-seq and 5’ scRNA-seq datasets showed coverage biases at the 3’ UTR and 5’ UTR, respectively. However, even within 5’ UTR, we observed that 5’ scRNA-seq datasets also exhibited highly variable evenness and 3’ biases (Fig. 1e). For instance, the COVID-19 and NSCLC datasets showed relatively higher evenness and lower 3’ biases compared to the mHSPC dataset. These results indicate that RNA degradation is a prevalent issue at the 5’ end of transcripts following 5’ scRNA-seq and Smart-seq2 protocols, highlighting the critical need for a correction to accurately identify and quantify TSSs.

To correct the 3’ positional biases caused by RNA degradation, we developed two complementary metrics: $\alpha$, representing the RNA degradation rate, and $\beta$, reflecting the transcript integrity. Both metrics can be applied at the TSS and sample levels. For the $\alpha$ metric, our approach adapts a robust quantitative model that was originally developed for bulk RNA-seq data^27^. We extended this framework to 5’ scRNA-seq data by redefining each TSS cluster (originating from the same annotated gene) as an independent transcriptional unit and using the cumulative number of its R1 read start sites as input. We then constructed a likelihood function based on the probability of the observed R1 start-site distribution under given parameters ($\alpha$ and $\theta$), and employed an EM algorithm to iteratively estimate the optimal parameters that maximise the likelihood of the observed data (Fig. 1a; see “Methods” and Supplementary Note 1). To complement the assessment provided by $\alpha$, we introduced a novel metric, $\beta$, which represents the relative area under the cumulative distribution curve for each TSS (Fig. 1a). A smaller $\beta$ value indicates that a greater proportion of reads originate from the degraded transcripts, resulting in a slower growth of the curve (see Supplementary Note 2), therefore a higher $\alpha$ or a smaller $\beta$ value indicates a greater degree of RNA degradation. scATS provides both parameters together to ensure a robust and comprehensive assessment of RNA integrity.

We analysed the level of RNA degradation in the mHSPC, COVID-19 and NSCLC datasets. The mHSPC dataset had a higher RNA degradation with the highest mean $\alpha$ (0.687) and lowest mean $\beta$ (0.737) (Supplementary Fig. 1a, b), consistent with the coverage analysis (Fig. 1e). For example, scATS identified *Chchd2* gene in the mHSPC dataset, whose proximal ATS exhibited low degradation ($\alpha$ = 0.14, $\beta$ = 0.97), while the distal ATS showed a high degradation level ($\alpha$ = 2.76, $\beta$ = 0.49) (Fig. 1f). The two TSSs occupied opposite extremes of the degradation spectrum (Fig. 1g), demonstrating that ATSs of the same gene can display highly divergent degradation rates. In general, distal TSSs exhibit more pronounced degradation than proximal ones (Fig. 1h, i). However, 259 genes (18.8%) exhibit at least one proximal ATS with a higher degradation rate than that of the most distal ATS (Supplementary Fig. 1c). *Mier1*, which is involved in early haematopoietic development through the regulation of haemogenic endothelium formation^28^, showed higher degradation at the proximal ATS ($\alpha$ = 0.51, $\beta$ = 0.60) than at the distal one ($\alpha$ = 0.29, $\beta$ = 0.75; Supplementary Fig. 1d).

To further correct degradation-dependent biases, scATS outputs two metrics to quantify the relative TSS usage: the PSIs with degradation-correction ($\theta$) or without correction ($\psi$). We observed a strong correlation between $\psi$ and $\theta$ values (*R* = 0.97, Pearson correlation; Fig. 1j), indicating that the profile of TSS usage is retained following correction. Importantly, TSSs with higher degradation rates ($\alpha$) exhibited elevated transcriptional levels after correction ($\theta$), indicating that $\theta$ compensates for degradation-induced underestimation (Fig. 1j). Notably, the corrected PSIs ($\theta$) are higher than the raw PSIs ($\psi$) at the most distant TSSs (Fig. 1k), where degradation rates are significantly more pronounced (Fig. 1h, i). In summary, our study highlighted the need to correct for RNA degradation in TSS quantification, which was previously overlooked in scRNA-seq analyses. We demonstrated that scATS can effectively compute RNA degradation at both the sample and isoform level, thereby enabling more accurate TSS identification and quantification in 5’ scRNA-seq datasets.

### Performance of scATS on simulated datasets

To evaluate the robustness of scATS to RNA degradation, we simulated a 5’ scRNA-seq dataset consisting of 3000 cells and 2000 genes based on the GRCh38 reference genome, using a negative binomial distribution^29^ to account for biological variability and technical noise. Raw reads were first aligned to generate BAM files, after which RNA degradation was simulated by applying an exponential decay function to R1 start sites (see “Methods” and Supplementary Note 3).

We first benchmarked the performance of different methods (Supplementary Data 2) using simulated data with mild RNA degradation ($\alpha$ = 0.2). For TSS identification, scATS and TSSr detected TSSs from all simulated genes, whereas SCAFE and scTSS detected TSSs of 638 genes. CamoTSS was excluded from this comparison, as its design focuses on TSS cluster-level analysis and therefore detected no TSSs under this simulation setting (see Supplementary Note 4). scATS showed the highest precision, recall and F1 score among all methods in TSS identification (Supplementary Fig. 1e), and also achieved the highest correlation with the ground truth (*R* = 0.96, Pearson correlation; Supplementary Fig. 1f).

In real 5’ scRNA-seq datasets, we observed that more than 99% of estimated $\alpha$ values were below 2, indicating that higher degradation levels rarely occur or are beyond the capability of scATS to identify TSSs. Therefore, we adopted this empirical range and simulated RNA degradation ranging from 0.2 to 2, with three independent replicates generated for each condition. DeepKINET^30^ is a recently proposed method designed to model RNA splicing and degradation effects in scRNA-seq data. We next compared scATS with DeepKINET using these simulated datasets. scATS accurately recovered the simulated $\alpha$ values (Supplementary Fig. 1g), with a mean correlation coefficient of 0.91 between the estimated and true $\alpha$ values (Supplementary Fig. 1h). In contrast, DeepKINET showed limited sensitivity to the increasing degradation severity (mean correlation coefficient = 0.20), consistent with its design focus on modelling internal transcript structure for inferring kinetic rates, cell clustering and trajectories rather than 5’ end–specific degradation.

### Robust identification of TSSs using scATS

**Fig. 2:**
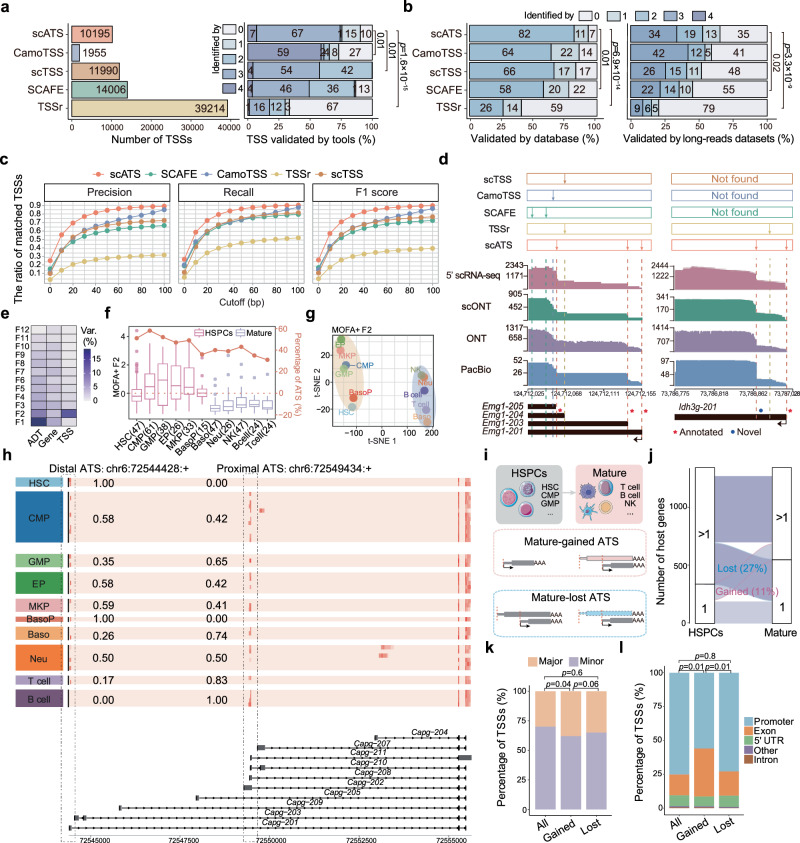
scATS robustly identifies TSSs and dynamic changes in TSS usage during haematopoietic cell differentiation. **a** Bar plots of total TSS number identified by scATS, CamoTSS, scTSS, SCAFE, and TSSr (left), and proportion of TSSs supported by $\ge 1$ other methods (right). Supporting source counts (0, none). **b** TSS validation using independent evidence within 50 base pair (bp): annotation databases (GENCODE, refTSS; left) and long-read sequencing datasets (scONT, ONT, PacBio; right). **c** TSS detection capability: precision (left), recall (middle), and F1 score (right) at varying bp cutoffs around GENCODE-annotated TSSs. **d** Sashimi plots of *Emg1* (left) and *Idh3g* (right) in 5’ scRNA-seq (pink), scONT (green), ONT (purple) and PacBio (blue) datasets. Arrows (upper) and dashed lines indicate predicted ATSs. Annotations below show annotated (red stars) and novel (blue circles) TSSs. **e** MOFA (Multi-Omics Factor Analysis) variance decomposition showing percentage variance (Var.) explained by factor (F) for ADT (antibody-derived tags), gene, or TSS clustering. **f** MOFA+ F2 values (box plots, left Y) and ATS percentages (line, right Y) comparing HSPCs (pink) and mature (purple) cells. Cell numbers in parentheses, box plots show median (center line) and IQR, whiskers ±1.5 × IQR. **g** t-SNE (t-distributed Stochastic Neighbour Embedding) plot of MOFA + F2 showing clustering of HSPCs (orange) and mature cells (blue). **h** Sashimi plots of *Capg* transcription in mHSPC 5’ scRNA-seq dataset. TSS usage and loci are indicated, with GENCODE v25 annotations. **i** Schematic of TSS dynamics during haematopoietic differentiation. Mature-gained and mature-lost ATSs indicate higher transcription in mature cells and HSPCs, respectively. Dendritic cell, myeloid stem cell, NK cell, haematopoietic stem cell and lymphoid stem cell icons provided by Servier Medical Art (https://smart.servier.com), licensed under CC BY 3.0 Unported (https://creativecommons.org/licenses/by/3.0/). B cell and mast cell icons created by El-Jayawant and Sebastian-Reinig, respectively, licensed under CC BY 4.0 Unported (https://creativecommons.org/licenses/by/4.0/). **j** Alluvial plots of TSS host gene distributions (1 versus >1 TSS) in HSPCs and mature cells. Gained and lost as in (**i**). **k**, **l** Bar charts of TSS types (**k**) and localisation (**l**). Major (**k**) and exon (**l**) ATSs are statistically compared. For statistical analysis: two-sided Proportion tests with BH correction (**a**, **b**, **k**, **l**). Exact *p* values shown in figures. Unlabeled comparisons are not significant.

To evaluate the performance of scATS, we applied scATS, along with four existing methods, SCAFE, CamoTSS, TSSr and scTSS, to quantify TSSs during haematopoiesis using the mHSPC 5’ scRNA-seq dataset (Supplementary Fig. 2a). TSSr, which was developed for bulk RNA-seq, identified most TSSs (*n* = 39,214). Three single-cell methods SCAFE (*n* = 14,006), scTSS (*n* = 11,990) and scATS (*n* = 10,195; Supplementary Data 3), recovered comparable numbers of TSSs, and CamoTSS detected the fewest TSSs (*n* = 1955) (Fig. 2a, left; see Supplementary Note 4). Next, we assessed the overlap among these five methods by calculating the percentage of TSSs supported by at least one of the other methods. scATS (90%) showed the highest overlap with other methods, followed by SCAFE (87%), CamoTSS (73%) and TSSr (33%). Note that scTSS was fully overlapped due to its reliance on the SCAFE and TSSr intersection and therefore was excluded in this comparison (Fig. 2a, right panel). To evaluate the accuracy in TSS identification, we evaluated six aspects: (i) concordance with two public TSS databases, GENCODE^31^ and refTSS^32^; (ii) the presence of 11 core promoter motifs; (iii) co-localisation with CpG islands, (iv) enrichment of histone marks, including H3K4me3 and H3K27ac for active promoters, and H3K4me1 for enhancers^33^; (v) enrichment of ATAC-seq peaks and (vi) evolutionary conservation (see “Methods”). Notably, 82% of TSSs were annotated by both GENCODE v25^31^ and refTSS^32^, substantially exceeding SCAFE (58%, Proportion test, *p* = 0.01) and TSSr (26%, *p* = 6.9 × 10^−14^; Fig. 2b, left panel). TSSs identified by scATS also exhibited high position accuracy, with a median distance of 8 bp from the GENCODE-annotated TSSs compared to median distance of 23 bp by SCAFE (Supplementary Fig. 2b). Moreover, scATS achieved the highest precision, recall, and F1 scores at all cutoff distances around the GENCODE-annotated TSSs (Fig. 2c), demonstrating superior accuracy in TSS identification.

Analysing the features of TSSs, we found that 53% of scATS-identified TSSs contained core promoter motifs, markedly surpassing those identified by all other tools (Proportion test, *p* < 0.0001; Supplementary Fig. 2c). Moreover, 79% (8031) of scATS-identified TSSs overlapped with CpG islands, lower than that by CamoTSS (89%), likely because CamoTSS recovers TSSs based on the ranked ATAC-seq peaks, but higher than the remaining methods (Supplementary Fig. 2d). Thirdly, we observed a characteristic signal dip for the promoter markers H3K4me3 and H3K27ac from −300 to +50 bp around the TSSs, while the enhancer marker H3K4me1 was enriched in the downstream regions (Supplementary Fig. 2e). These profiles matched with the patterns of annotated TSSs by GENCODE and CAGE-seq, which was not demonstrated in randomly selected loci. Additionally, the scATS-identified TSSs showed a high degree of ATAC-seq peaks (Supplementary Fig. 2f) and evolutionary conservation estimated by PhastCons (Supplementary Fig. 2g).

To validate the accuracy of scATS in TSS identification, we further generated three long-read sequencing datasets for mHSPCs, by single-cell Oxford Nanopore Technology (scONT), bulk ONT and Pacifc Bioscience (PacBio) (Supplementary Data 1). scATS showed the highest validation percentage (65%) by at least one long-read sequencing dataset, higher than that of SCAFE (45%, Proportion test, *p* = 0.02) and TSSr (21%, *p* = 3.3 × 10^−9^; Fig. 2b, right panel) and other methods. For the *Emg1* gene, scATS identified three annotated TSSs in the 5’ scRNA-seq dataset, all of which were validated by the long-read datasets. In contrast, TSSr and scTSS identified one annotated TSS that lacked of long-read validation, the other methods incorrectly identified the ATSs. Similarly, scATS was the only method that identified 2 TSSs in the *Idh3g* gene, one annotated and one novel TSS, but TSSr detected only a novel TSS and the other methods failed to recover the full TSS structure (Fig. 2d). In summary, scATS provides a balanced framework for accurate TSS identification in 5’ scRNA-seq data, supported by the annotation concordance, enrichment of the regulatory features and validation by the long-reads.

### Dynamic expression of TSSs during haematopoiesis

As the alternative 5’ UTR usage has been reported during stem cell differentiation^34^, we applied scATS to the mHSPC 5’ scRNA-seq dataset to identify TSSs with dynamic expression. Using MOFA+ to decompose the multimodal data^35^, we identified 12 factors (F). F1 was primarily driven by the variance in antibody-derived tags (ADTs), whereas F2 captured the variance in TSS usage (Fig. 2e and Supplementary Fig. 3a). Interestingly, the values of F2 were higher in HSPCs compared to mature cells. Consistently, the HSPCs expressed genes with higher percentages of ATSs (Fig. 2f). This clustering was also validated by t-distributed stochastic neighbour embedding (t-SNE)^36,37^ (Fig. 2g). The *Capg* gene, which is involved in the NF-κB signalling pathway and influences the growth and development of haematopoietic cells^38^, demonstrated dynamic TSS regulation. To identify the ATS that are highly variable during haematopoiesis, we performed a pseudo-bulk analysis to compare the ATS changes in different cell types. The proximal ATS of *Capg* (chr6:72549434:+) showed a high variability (variance = 0.080; Standard Deviation [SD] = 0.283), and its two TSSs are cell-type specific, indicating its contribution to the cell clustering. HSPCs preferentially utilised the distal ATS (chr6:72544428:+) of *Capg*, while the proximal ATS (chr6:72549434:+) was more highly transcribed in the mature cells (Fig. 2h and Supplementary Fig. 3b). This pattern of differential TSS usage was confirmed using an independent Smart-seq2 dataset from Tabula Muris consortium^39^ (Supplementary Fig. 3c).

To detect the dynamic changes of TSS usage between HSPCs and mature cells (Fig. 2i), we compared the TSSs across all cell-types and observed 38% were dynamically utilised during the differentiation process (Fig. 2j). Notably, a prevalent loss of TSSs that were more highly transcribed in HSPCs was seen in the mature cell-types (Supplementary Fig. 3d). We observed that the majority of mature-lost TSSs originated from proximal sites (61%; Supplementary Fig. 3e), and these TSSs showed a higher proportion of exonic localisation (Supplementary Fig. 3f) and lower downstream sequence conservation (Supplementary Fig. 3g). For instance, the *Ctse* gene, known to interact with mature T cells and affect PD-1 effectiveness^40^, showed expression of a proximal exonic ATS in HSPCs that was lost in the mature cells (Supplementary Fig. 3h, i). Moreover, the TSSs active in HSPCs (mature-lost ATSs) were enriched in pathways such as “stem cell population maintenance” and “primitive hemopoiesis”, while those gained in mature cells (mature-gained ATSs) were enriched with pathways of “positive regulation of developmental growth”, “regulation of myeloid cell differentiation” and “neutrophil activation” (Supplementary Fig. 3j). Interestingly, ATSs gained during cell differentiation contained a higher proportion of major isoform and exonic ATSs compared to mature-lost ATSs (Fig. 2k, l; see “Methods”), suggesting that they may play important roles.

To reconstruct gene regulatory networks (GRN) associated with TSSs, we analysed TF activity using SCENIC^41^. This analysis identified five differential regulons from 504 host genes carrying gain or loss TSSs (Supplementary Fig. 3k, l). Among these, Spi1, a TF reported to regulate haematopoietic lineage commitment^42^, exhibited a higher regulon specificity score and higher gene expression level in HSPCs and preferentially targeted genes with mature-lost TSSs. In contrast, Stat3, which was reported to regulate CD8 + T cell differentiation^43^, was highly activated at both the regulon and gene level in mature cells and preferentially targeted genes carrying mature-gained TSSs (Supplementary Fig. 3m). Taken together, scATS reveals a dynamic TSS usage and offers a framework to explore cell-type-specific regulatory profiles during haematopoiesis.

### Altered TSS usage in the immune cells in response to SARS-CoV-2 infection

**Fig. 3:**
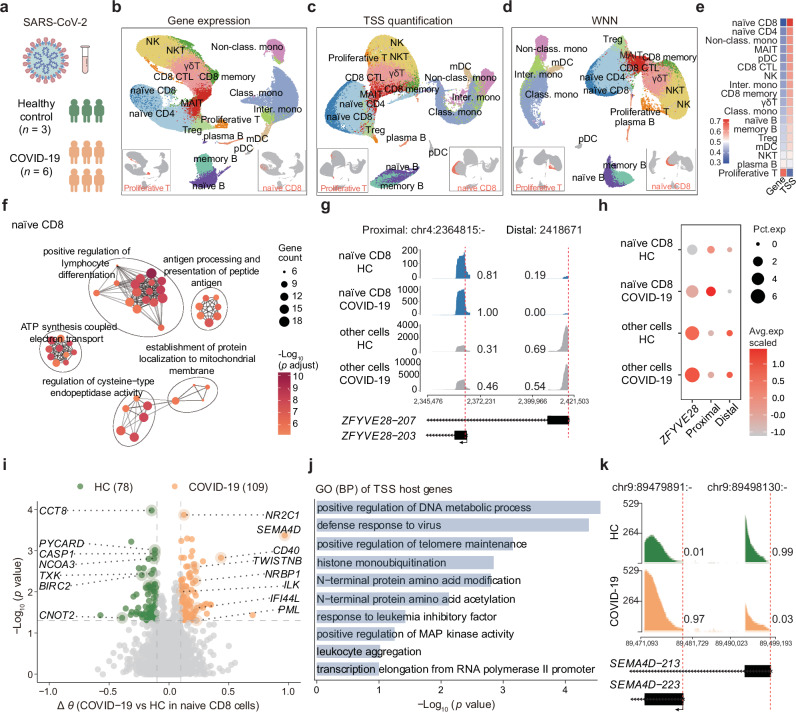
TSS-derived isoforms enhance the resolution of cell clustering. **a** Summary of the COVID-19 datasets, PBMCs of 6 female patients recovered from SARS-CoV-2 infection are analysed by 5’ scRNA-seq together with 3 healthy controls (HC). COVID-19, people and blood sample icons adapted from our previous study^110^. **b**–**d** UMAP plots showing cell clustering based on gene expression (**b**), TSS quantification (**c**) and the integration of gene and TSS metrics by the WNN (Weighted Nearest-Neighbour) model (**d**). Cell-types are indicated by different colors. The insets highlight naïve CD8 and proliferative T cells (red). **e** Mean modality weights for each cell-type, clustered based on gene expression (**b**) and TSS quantification (**c**). The color scale indicates the weights derived from the WNN analysis. **f** Gene Ontology (GO) enrichment network of TSSs highly transcribed in naïve CD8 cells. GO terms (nodes) with high similarity are grouped into networks, and related terms are circled. Node size indicates the number of enriched genes, line thickness represents similarity scores, and node color shows −log10 adjusted *p*-values. **g**, **h**, Sashimi plots (**g**) and dot plots (**h**) showing the expression levels of *ZFYVE28* gene and TSSs in naïve CD8 cells and other cells. PSI values in (**g**), percent cells (dot size), and average expression (dot color) in (**h**) shown. **i** Volcano plot showing differentially utilised TSSs in naïve CD8 cells from COVID-19 compared to HC groups. The X- and Y-axes represent Δ$\theta$ and the −log10 *p* value, respectively, with cutoffs of |Δ$\theta$| > 0.1 and *p* value < 0.05. Yellow and green indicate high transcription in COVID-19 and HC, respectively; gray dots, non-significant; counts in parentheses. **j** GO enrichment analysis of host genes with differentially used TSSs is shown. BP biological processes. The bars depict −log10 *p* values. **k** Sashimi plots showing the transcription level of *SEMA4D* TSSs in naïve CD8 cells. Red dashed lines and PSI values are indicated. For statistical analysis, the following tests were used: one-sided hypergeometric tests with BH correction (**f**, **j**), and two-sided Proportion tests (**i**). Exact *p* values are provided in the figures.

To identify TSS usage during pathogen infection, we applied scATS to a 5’ scRNA-seq dataset profiling peripheral blood mononuclear cells (PBMCs) from female patients recovered from COVID-19 (*n* = 6) and healthy controls (HC, *n* = 3)^24^ (Fig. 3a). scATS identified 21,287 TSSs in 7012 genes across 90,127 cells of 18 immune cell-types (Supplementary Data 3), including both the lymphoid and myeloid lineages (Supplementary Fig. 4a, b).

To dissect the contributions of gene expression and TSS usage in defining cell clusters, we treated them as “different modalities” by using weighted nearest-neighbour (WNN) in Seurat^44^. Cell clusterings by gene expression and TSSs revealed relatively consistent cell classifications (Fig. 3b–d), while they resolved better in certain cell types. For example, proliferative T cells were clustered better by gene expression (Fig. 3b). In contrast, naïve CD8 cells, which were not separated from naïve CD4 cells in the gene-expression clustering, were distinctly clustered using TSS quantification (Fig. 3c). These results are consistent with the WNN weights, with proliferative T cells and naïve CD8 cells having higher WNN values in genes and TSSs, respectively (Fig. 3e). Together, an integrated UMAP combining both gene and TSS modalities provided a more refined cell clustering (Fig. 3d).

To explore the cell-type-specific TSS regulation, we identified 5291 differentially utilised TSSs across the 18 cell-types (absolute Δ $\theta$ > 0.1, Wilcoxon test *p* value < 0.05) (Supplementary Data 4). Gene Ontology (GO) enrichment analysis of their host genes revealed pathways highly relevant to cell-type-specific functions (Supplementary Data 5). In naïve CD8 cells, 522 host genes of cell-specific TSSs were enriched in the “positive regulation of lymphocyte differentiation” pathway (Fig. 3f). For example, the proximal ATS (chr4:2364815:−) of *ZFYVE28* exhibited predominant transcription level in naïve CD8 cells, while the distal ATS (chr4:2418671:−) was more highly transcribed in other cell-types, a pattern shared in both HC and COVID-19 groups (Fig. 3g, h). Furthermore, we compared these 5291 differentially utilised TSSs (representing 3455 host genes) with 3441 differentially expressed genes identified across cell types. In 13 out of 18 cell types, fewer than 10% of genes were differentially expressed at both the TSS and gene levels, ranging from 1% in MAIT cells to 30% in pDCs (Supplementary Fig. 4c). This observation demonstrates that significant shifts in TSS preference can occur even when overall transcript abundance remains stable, highlighting a layer of regulation not captured by gene-level analysis.

Next, scATS identified 187 TSSs with differential TSS usage in naïve CD8 cells between COVID-19 and HC individuals (Fig. 3i). The host genes including *NR2C1*, *SEMA4D* and *CD40*, were enriched in pathways such as “defense response to virus” and “leukocyte aggregation” (Fig. 3j). For example, *SEMA4D*, which was enriched in “leukocyte aggregation” in both our analysis and a previous study^45^, showed different TSS usage between the two groups. The proximal ATS (chr9:89479891:−) was highly transcribed in PBMCs of COVID-19 patients, while the distal ATS (chr9:89498130:−) was more highly utilised in HC individuals (Fig. 3k and Supplementary Fig. 4d). Furthermore, we applied SCENIC to identify the upstream TFs regulating *SEMA4D* in naïve CD8 cells and revealed TCF7 as a potential regulator. TCF7 was reported to play a crucial role in maintaining T cell self-renewal and antiviral immune function^46^, which is also associated with increased regulon activity and gene expression levels in COVID-19 individuals (Supplementary Fig. 4e, f). These findings underscore the dynamic regulation during immune responses against SARS-CoV-2 infection.

### Tumour-specific TSS expression in human NSCLC

**Fig. 4:**
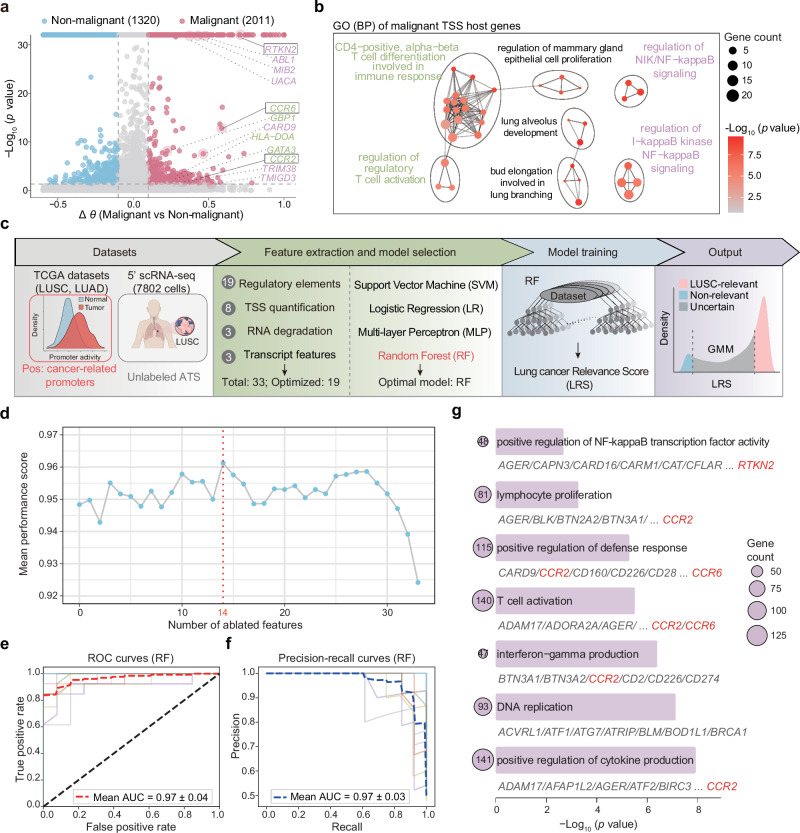
LRS predicts TSSs related to human NSCLC. **a** Volcano plot showing differentially used TSSs between malignant and non-malignant cells in human NSCLC dataset. The X- and Y-axes represent Δ$\theta$ and the −log10 *p* value, respectively, with cutoffs of |Δ$\theta$| > 0.1 and *p* value < 0.05. Pink and blue indicate high transcription in malignant and non-malignant cells, respectively; gray dots, non-significant; counts in parentheses. **b** Enrichment analysis of host genes with differentially utilised TSSs in malignant cells (pink dots in **a**). The associated terms (nodes) are circled. Node size indicates the number of enriched genes, line thickness represents similarity scores, and node color shows −log10 adjusted *p* values. NF-kappaB (purple) and T cells (green) terms highlighted with corresponding genes in matching colors in (**a**), and three experimentally analysed genes are boxed. **c** The LRS (lung cancer relevance score) workflow: (1) Training positive TSSs from LUSC (Lung Squamous Cell Carcinoma) and LUAD (Lung Adenocarcinoma) datasets^8^; (2) RF (Random Forest) model selection and feature ablation; (3) model training using RF model and 19 features; (4) Classification of TSSs as relevant, non-relevant, or uncertain using a Gaussian Mixture Model (GMM). Created in BioRender. Xu, Z. (2026) https://BioRender.com/2d4q2kf. **d** Line plot showing model performance with different numbers of ablated features. Mean performance scores are calculated as the average of AUROC (Area Under the Receiver Operating Characteristic curve) and AUPRC (Area Under the Precision-Recall Curve). The red dashed line indicates the number of ablated features that yields the best performance. **e**, **f** Performance of the final LRS model assessed using AUROC (**e**) and AUPRC (**f**). The red and blue dashed lines represent the overall AUROC and AUPRC from tenfold cross-validation, respectively. These mean AUCs ± SD are shown. **g** GO enrichment analysis of host genes with lung cancer-relevant TSSs. Numbers indicate gene counts per term, with contributing genes partially listed below. Bars represent −log10 *p* values, and three experimentally analysed genes are highlighted in red. For statistical analysis, the following tests were used: two-sided Proportion tests (**a**), and one-sided hypergeometric tests (**b**, **g**). Exact *p* values are provided in the figures.

Epithelial cells, as the principal cellular components of lung cancer and influence early detection, prognosis assessment, and therapeutic strategies of NSCLC^47,48^. To explore the potential roles of ATS in lung squamous cell carcinoma (LUSC), we employed scATS on a 5’ scRNA-seq dataset containing 4299 epithelial cells from the 10× Genomics public database. Cells were classified as malignant or non-malignant using copy number variants (CNVs)^49–53^ (Supplementary Fig. 5a). The malignant cells exhibited amplifications on chromosomes 7, 11 and 20 (Supplementary Fig. 5b), as well as deletions on chromosomes 6, 14 and 21 (Supplementary Fig. 5c). These observed CNV patterns were consistent with reported characteristics of LUSC^54^. We further identified 3331 TSSs that exhibited differential usage between the malignant and non-malignant cells (Fig. 4a). The host genes of TSSs upregulated in malignant cells were enriched in the “regulation of NIK/NF-kappaB signalling” (e.g., *RTKN2*, *ABL1* and *MIB2*) and “regulation of regulatory T cell activation” (e.g., *GATA3*, *CCR2* and *CCR6*) pathways (Fig. 4b). These pathways have been implicated in inflammatory response, immune evasion, and tumour progression in NSCLC^55–58^. To dissect these TSS-level shifts, we compared genes with differentially utilised TSSs to those with differential gene expression. Notably, 96% of genes with differential TSS usage showed no significant change in gene expression (Supplementary Fig. 5d). *C1QA*, which contributes to an immunosuppressive tumour microenvironment in NSCLC^59^, showed disease specificity at both the gene expression and TSS usage (Supplementary Fig. 5e). In contrast, *CCR2*, which is linked to T-cell recruitment and favourable prognosis in NSCLC^60^, showed disease specificity only at the TSS usage (Supplementary Fig. 5f).

To characterise the potential regulation between TFs and TSSs, we identified 26 regulons organised into 4 major modules (Supplementary Fig. 5g). For example, the STAT1 and ELF1 TFs were linked to immune-related and invasive processes in NSCLC^61,62^, and both preferentially targeted genes with dominant TSS usage in malignant cells, including *CCR6* and *RTKN2* (Supplementary Fig. 5h, i).

### Predictive machine-learning model for lung cancer relevance score (LRS)

To identify key TSSs in lung cancer, we further developed a machine-learning model to calculate a LRS for each TSS (Fig. 4c). We included 129 TSSs previously identified in a large-scale pan-cancer transcriptome study based on TCGA datasets^8^. The host genes of these TSSs have been reported to be involved in cancer prognosis (Supplementary Data 6), making them a relevant reference set for our predictions. We utilised a Positive-Unlabeled (PU) learning spy algorithm by incorporating 10% of the positive TSSs as “spies” and randomly selected 129 TSSs as the negative set^63^ (see “Methods” and Supplementary Note 5). Next, we trained a LRS model that predicts TSSs that are closer to the positive set and farther from the negative set. To achieve this, we extracted 33 features spanning four categories (Supplementary Data 7): (1) regulatory elements (19 features): histone modification signals at the TSSs and within ±2500 bp, TF motifs within ±1000 bp, CpG-related metrics, and conservation scores; (2) TSS quantification (8 features): TSS expression levels in different cell-types; (3) RNA degradation (3 features): TSS degradation metrics ($\alpha$ and $\beta$); (4) transcript features (3 features): properties of the TSS transcripts, including distances to reference TSSs, coding status of TSS transcript, and strand orientations (see “Methods”).

We next compared the performance of 4 machine-learning models, including random forest (RF), logistic regression (LR), multilayer perceptron (MLP) and support vector machine (SVM) (Supplementary Fig. 6a–d), on the training set using tenfold cross-validation (see “Methods”). As the RF model demonstrated the highest performance (mean area under the receiver operating characteristic curve [AUROC] = 0.94, mean area under the precision–recall curve [AUPRC] = 0.95), we selected RF as the baseline model for the subsequent analyses (see Supplementary Note 6). The importance of all features was evaluated based on their Gini scores (Supplementary Data 7), and feature ablation studies were performed to select the optimal sets of features (Fig. 4d, Supplementary Fig. 6e and Supplementary Data 8). After removing 14 bottom-ranked features, the final LRS model exhibited optimal performance, showing a mean AUROC of 0.97 and a mean AUPRC of 0.97 (Fig. 4e, f).

Next, we applied the LRS model to 25,121 TSSs that were transcribed in the epithelial cells and assigned the prediction scores ranging from 0 to 1, where a score closer to 1 indicates a higher likelihood of relevance with LUSC (Supplementary Data 9). After fitting the LRS distribution of all predicted TSSs with a random-component Gaussian Mixture Model (GMM), we categorised the TSSs into three classes: “LUSC-relevant” (LRS > 0.70), “non-relevant” (LRS < 0.47) and “uncertain” (LRS between 0.47 and 0.70). In total, we identified 8818 TSSs likely to be LUSC-relevant. GO analysis revealed that the host genes of the relevant TSSs were enriched in immune-related pathways, including “lymphocyte proliferation”, “T cell activation”, “interferon-gamma production” and “positive regulation of cytokine production”. Additional enriched pathways comprised “positive regulation of NF-kappaB TF activity” and “DNA replication” (Fig. 4g). These pathways have been reported to play important roles in LUSC^55–58^, demonstrating the ability of LRS in identifying key TSSs in lung cancer. To further validate the results of LRS, we performed experiments in ATS of three genes with LRS values ranging from 0.7 to 0.9. The first two genes are chemokine receptors *CCR6* and *CCR2*, which have been repeatedly shown in the immune-related pathways, with LRS values of 0.95 and 0.84, respectively. And *RTKN2*, from the “positive regulation of NF-kappaB TF activity” pathway, and its TSS (chr10:62268845:−) showed a LRS of 0.72 (Fig. 4g and Supplementary Data 9).

### Isoforms of *CCR6* and *CCR2* transcribed in non-malignant cells demonstrate tumour suppressor effect by reducing the level of HMGCR

**Fig. 5:**
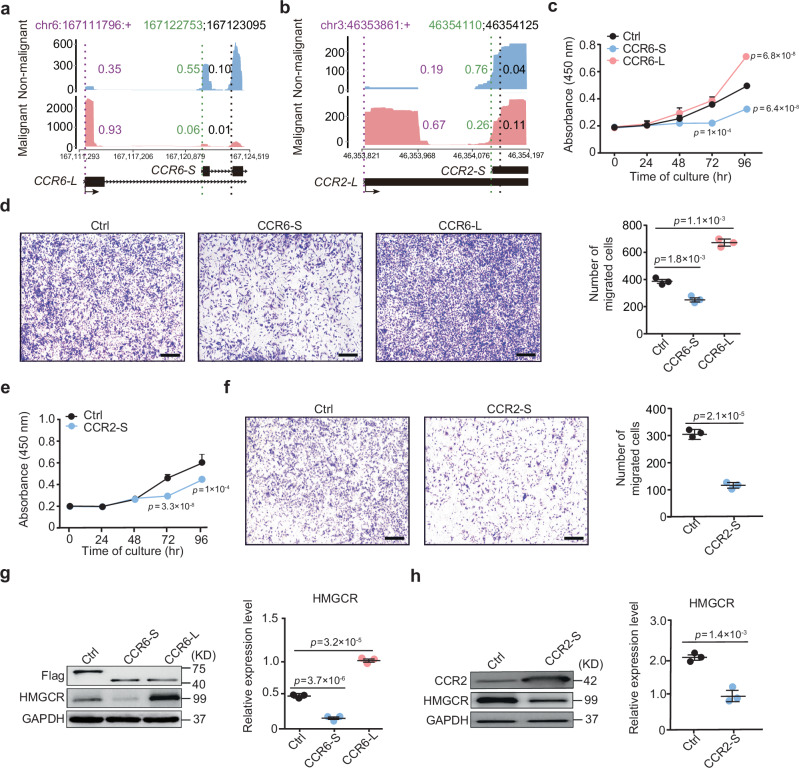
*CCR6* and *CCR2* isoforms exhibit different effects in the proliferation and migration of NSCLC cells. **a**, **b** Sashimi plots showing the transcription level of *CCR6* (**a**) and *CCR2* (**b**) in the malignant (red) and non-malignant cells (blue) of NSCLC 5’ scRNA-seq dataset. The dashed lines indicate the loci of predicted ATSs inferred by scATS with PSIs of the corresponding ATS-derived isoform provided near the peaks. The ATSs with higher expression in the malignant cells are highlighted in purple, ATSs transcribed higher in non-malignant cells are highlighted in green, and those with no significant differences are shown in black. Annotations provided below the sashimi plots. **c**–**f** Calu-1 cells were transfected with lentivirus expressing Flag-tagged Catalase (CAT, Ctrl), CCR6-S, CCR6-L or CCR2-S and treated with puromycin for 3 days before subjecting to cell counting kit (CCK)-8 or transwell assay. Absorbance at 450 nm (*n* = 4) was measured at different time points (**c**, **e**). Representative images of the transwell assay taken at 24 h after seeding the overexpressing cells (left panels, ×40) and the numbers of migrated cells are shown (right panels, *n* = 3) (**d**, **f**). Scale bars, 40 μm. **g**, **h**, Representative immunoblots (left panels) of HMGCR in Calu-1 cells overexpressing Flag-tagged CAT (Ctrl), CCR6-S, CCR6-L (**g**) or CCR2-S (**h**). The level of HMGCR was detected by anti-HMGCR antibody, the levels of CAT and CCR6 were detected by anti-Flag antibody (**g**), and CCR2 was detected by anti-CCR2 antibody (**h**). The relative signals of HMGCR/GAPDH were quantified using ImageJ (right panels). For statistical analysis, the following tests were used: Multiple unpaired two-tailed Student’s *t*-tests (**c**–**h**). Experiments of (**c**, **e**) were repeated four times and (**d**, **f**) were repeated three times, consistently yielding similar results in each iteration. Data in (**c**–**h**) are represented as mean ± SEM. Data in (**g**, **h**) pooled from three independent experiments and (**c**–**f**) pooled from two independent experiments. Source data are provided as a Source Data file.

*CCR6*^64^ and *CCR2*^60^ were predicted to have high LRS TSSs and exhibited differential $\theta$ values between the malignant and non-malignant NSCLC cells (Supplementary Data 9 and Fig. 5a, b). Specifically, the distal ATS of *CCR6* (chr6:167111796:+) and *CCR2* (chr3:46353861:+) were primarily utilised in the malignant cells, producing a long isoform (*CCR6-L*, $\theta$ = 0.93) and a non-protein-coding transcript *CCR2-L* ($\theta$ = 0.67), respectively. In contrast, the *CCR6-S* (chr6:167122753:+, $\theta$ = 0.55) and *CCR2-S* (chr3:46354110:+, $\theta$ = 0.76) were transcribed higher in non-malignant cells (Fig. 5a, b).

We further analysed the functions of the ATS isoforms of *CCR6* and *CCR2*. We overexpressed CCR6-S and CCR6-L and their 5’ UTR in Calu-1, an NSCLC cell line of LUSC origin (Supplementary Fig. 7a). Notably, CCR6-L significantly promoted cell proliferation, while CCR6-S exerted an inhibitory effect. As early as 48 h after culture, the cell number was 5% lower in the CCR6-S-expressing cells compared to the control cells overexpressing CAT in the Cell Counting Kit-8 assay. At 72 h CCR6-L-expressing cells started to show significant increase and at 96 h, the increase was 56% higher, while CCR6-S-expressing cells were 34% lower than the control (Fig. 5c). Moreover, the transwell assays demonstrated that CCR6-L overexpression resulted in an approximately 75% increase in the migrated cell number at 24 h after seeding of the cells; in contrast, CCR6-S led to a 38% reduction compared to the control (Fig. 5d). Similarly, non-malignant cells expressing CCR2-S significantly inhibited the proliferation of Calu-1 cells, as the cell number was 43% lower than the control cells at 72 h after culture (Fig. 5e and Supplementary Fig. 7b). And CCR2-S also led to an approximately 64% reduction in the migrated cell number compared to the control (Fig. 5f).

Next, we repeated the analyses in A549, an NSCLC cell line of lung adenocarcinoma (LUAD) origin (Supplementary Fig. 7c, d). In line with the results observed in Calu-1 cells, CCR6-L led to significantly higher cell proliferation, while CCR6-S resulted in lower cell proliferation. Similarly, CCR2-S significantly inhibited the proliferation of A549 cells (Supplementary Fig. 7e, f). The transwell assays showed that CCR6-L resulted in a threefold increase in the migrated cell number compared to the control, and CCR6-S and CCR2-S led to 60% and 67% reductions, respectively (Supplementary Fig. 7g, h). Furthermore, it was reported that CCR6 interacted with HMGCR^65^, and HMGCR promoted the proliferation and migration of lung cancer cells^66^. To explore whether HMGCR was affected by CCR6 and CCR2, we performed Western blot analysis of the three isoform-overexpressing cell lines. Our results showed that CCR6-L increased HMGCR expression by 45% compared to the control; in contrast, CCR6-S and CCR2-S both reduced HMGCR expression by 50% and 57%, respectively (Fig. 5g, h).

Taken together, we demonstrate that the chemokines *CCR6* and *CCR2* have ATSs that are selectively utilised in the malignant or non-malignant cells. The ATS isoforms dominant in the non-malignant cells, CCR6-S and CCR2-S, exhibit tumour-suppressive properties evident by the overexpression leading to lower cell proliferation and migration. In contrast, the distal TSS of *CCR6* that is highly activated in the malignant cells generates CCR6-L, resulting in higher cell proliferation and migration. Interestingly, both CCR6-S and CCR2-S reduce HMGCR expression, while CCR6-L promotes the expression.

### The long isoform *RTKN2* transcribed lower in the malignant cells suppresses cell proliferation and migration by interaction with SREBP2

**Fig. 6:**
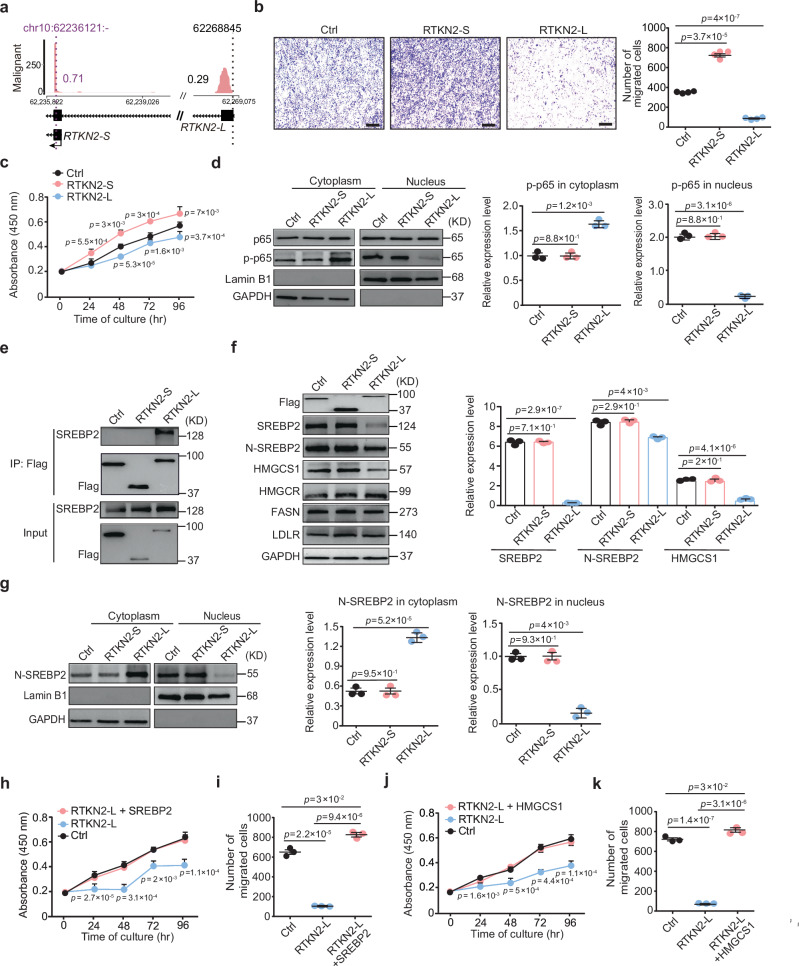
The long isoform *RTKN2* expressed lower in the malignant cells suppresses cell proliferation and migration by inhibiting nuclear translocation of N-SREBP2 and p-p65. **a** Sashimi plot showing the transcription level of *RTKN2* ATSs in the malignant cells of NSCLC 5’ scRNA-seq dataset. The dashed lines indicate the loci of predicted ATSs inferred by scATS with PSIs of the corresponding ATS-derived isoform provided near the peaks. **b**, **c** Calu-1 cells overexpressing Flag-tagged CAT (Ctrl), RTKN2-S, or RTKN2-L were analysed by transwell (*n* = 4) (**b**) and CCK-8 proliferation assay (*n* = 4) (**c**) after 3 days of puromycin selection. Absorbance at 450 nm. Scale bars, 40 μm. **d** Immunoblot analysis of the p-p65 expression in the cytoplasmic and nuclear fractions. The level of p-p65 was detected by anti-p-p65 antibody (left panel). The relative signals of p-p65/GAPDH and p-p65/Lamin B1 were quantified by ImageJ (right panel). **e** HEK293T cells transiently transfected with Flag-tagged Ctrl, RTKN2-S, or RTKN2-L and SREBP2-Myc were cultured for 48 h before collection. Reciprocal co-immunoprecipitation experiments with anti-Flag agarose beads were visualised by immunoblot analysis using anti-Flag and anti-Myc antibodies. **f**, **g** Representative immunoblots and quantification of genes involved in cholesterol biosynthesis (**f**) and N-SREBP2 expression in the cytoplasmic and nuclear fractions (**g**) (left panels). The relative signals of SREBP2/GAPDH, N-SREBP2/GAPDH, HMGCS1/GAPDH (right panel) (**f**), N-SREBP2/GAPDH (middle panel) and N-SREBP2/Lamin B1 (right panel) (**g**) were quantified using ImageJ. **h–k** Calu-1 cells overexpressing Flag-tagged Ctrl, RTKN2-S, or RTKN2-L and treated with corresponding selection drugs were analysed by CCK-8 proliferation (*n* = 4) (**h**, **j**) and transwell assay (*n* = 3) (**i**, **k**) after 3 days of puromycin and G418 selection. Absorbance at 450 nm. Scale bars, 40 μm. For statistical analysis, the following tests were used: Multiple unpaired two-tailed Student’s *t*-tests (**b**–**k**). Experiments of (**b**, **c**, **h**, **j**) were repeated four times and (**i**, **k**) were repeated three times, consistently yielding similar results in each iteration. Data in (**b**–**k**) are represented as mean ± SEM. Data in (**d**, **f**, **g**) pooled from three independent experiments and (**b**, **c**, **e**, **h**–**k**) pooled from two independent experiments. Source data are provided as a Source Data file.

The Rho GTPase effector gene *RTKN2* was reported to inhibit the growth and migration of several cancers^67–72^, including LUAD, through inactivating the NF-κB signalling pathway^73^. We identified two ATSs of *RTKN2*: the proximal ATS (chr10:62236121:−) producing a short isoform (*RTKN2-S*) and the distal ATS (chr10:62268845:−) producing a long isoform (*RTKN2-L*) in the 5’ scRNA-seq and CAGE dataset. *RTKN2-S* was highly activated in the malignant cells ($\theta$ = 0.71), and *RTKN2-L* exhibited lower usage ($\theta$ = 0.29) (Fig. 6a and Supplementary Fig. 8a).

To assess the functions of the *RTKN2* isoforms, we overexpressed RTKN2-S and RTKN2-L in Calu-1 (Supplementary Fig. 8b). Interestingly, the transwell assays indicated that RTKN2-S had a twofold increase in the migrated Calu-1 cells number, in contrast, RTKN2-L-expressing cells led to a 75% reduction in the migrated cell number compared to the control (Fig. 6b). Malignant cell-favored RTKN2-S significantly promoted, while RTKN2-L inhibited, the cell proliferation of Calu-1 cells compared to the control cells overexpressing CAT. As early as 24 h after culture, the cell number was 40% higher in the RTKN2-S-expressing cells and 8% lower in the RTKN2-L-expressing cells compared to the control cells, as shown in the Cell Counting Kit-8 assay. At 96 h, the RTKN2-S-expressing cells were 18% higher than the control, whereas RTKN2-L-expressing cells were 12% lower (Fig. 6c).

We repeated the analyses in A549 and found similar results as in Calu-1. Overexpression of RTKN2-S led to higher cell proliferation, as 50% higher cell number was shown compared to the control as early as 24 h after culture, while lower proliferation (10%) was seen in the RTKN2-L-expressing cells as measured by the Cell Counting Kit-8 assay. At 96 h, RTKN2-S-expressing cells were 20% higher, while RTKN2-L-expressing cells were 30% lower than the control cells (Supplementary Fig. 8e, f). The transwell assay demonstrated that RTKN2-S resulted in a twofold increase in the migrated cell number compared to the control, while RTKN2-L led to an approximately 48% decrease (Supplementary Fig. 8g). Combined, long and short isoforms of *RTKN2* show different effects in lung cancer cells, with the *RTKN2-S* highly transcribed in the malignant cells promoting cell proliferation and migration, while the *RTKN2-L* demonstrates a tumour suppression effect.

We next investigate whether *RTKN2-S* and *RTKN2-L* differ in regulating the NF-κB signalling pathway in LUSC. Both RTKN2-S- and RTKN2-L-expressing cells had comparable protein and phosphorylation levels of NF-κB p65 and IκBα in Calu-1 cells (Supplementary Fig. 8h). Interestingly, although the levels of p65 and p-p65 (Ser536) were similar in both the cytoplasmic and nuclear fractions of RTKN2-S-expressing and control cells, the p-p65 level was higher (up to 67%) in the cytoplasm while significantly lower (up to 90%) in the nucleus of RTKN2-L expressing cells (Fig. 6d), suggesting that RTKN2-L inhibits nuclear translocation of p-p65.

Aside from NF-κB regulation, it was reported that RTKN2 interacts with SREBP2^74^, a TF that regulates cholesterol production^75^. We therefore performed a co-immunoprecipitation assay and found that RTKN2-L, but not RTKN2-S, interacted with SREBP2 (Fig. 6e). Western blot analysis showed that overexpression of RTKN2-L led to reduction of the total level and the N-terminal fragment of SREBP2 (N-SREBP2) compared to the control (Fig. 6f). Notably, RTKN2-L led to a higher retention of N-SREBP2 in the cytoplasm and an 89% decrease of N-SREBP2 in the nucleus (Fig. 6g). The level of the downstream cholesterol-biosynthesis enzyme HMGCS1 was also reduced by 86%, while surprisingly the level of HMGCR was not affected. These findings suggest that by interacting with SREBP2, RTKN2-L inhibits the nuclear translocation of N-SREBP2 and selectively affects the downstream target, HMGCS1.

To validate that the suppression of cell proliferation and migration by RTKN2-L was dependent on its interaction with SREBP2. We overexpressed SREBP2 or HMGCS1 in the RTKN2-L-expressing Calu-1 cells and analysed the cell proliferation and migration (Supplementary Fig. 8c, d). Overexpression of SREBP2 restored the cell proliferation to the level of the control cells (Fig. 6h). Moreover, the migration cell number of RTNK2-L-expressing cell was reduced by 75% compared to the control, while overexpression of SREBP2 upregulated the migration cell number to the control level (Fig. 6i). Similarly, overexpression of HMGCS1 restored the cell proliferation and migration (Fig. 6j, k).

Taken together, *RTKN2-L* acts as a tumour suppressor by inhibiting cell proliferation and migration, whereas *RTKN2-S* functions as an oncogene by enhancing tumourigenicity in lung cancer cells. Importantly, we show that RTKN2-L inactivates NF-κB signalling by inhibiting nuclear translocation of p-p65. In addition, RTKN2-L interacts with SREBP2, suppressing the nuclear translocation of N-SREBP2 and thereby inactivating downstream HMGCS1 expression.

## Discussion

RNA degradation is a major challenge in the analysis of 5’ scRNA-seq^76,77^, which distorts the read coverage and reduces the accuracy of TSS quantification. Existing tools, such as SCAFE and CamoTSS, do not account for this effect, which limits accurate profiling of TSSs, a key regulatory mechanism in biological processes including cell differentiation^6^, immune responses^7^ and tumourigenesis^8^. To overcome this limitation, we developed scATS, a de novo framework with two major innovations. First, it introduces degradation-specific metrics: $\alpha$, a degradation rate inferred using an EM algorithm, and $\beta$, the normalised area under the ECDF curve. Both metrics can be calculated at either the TSS or sample levels, enabling targeted quality control. Second, scATS outputs both raw ($\psi$) and degradation-corrected ($\theta$) TSS usage, with the corrected $\theta$ values substantially improving quantification accuracy in RNA samples with considerable degradation. scATS uncovered unannotated TSSs that were validated by long-read RNA sequencing but not detected by other methods. Third, although methods such as SCAFE and CamoTSS leverage ATAC-seq information to assist TSS inference, matched ATAC–RNA datasets are not routinely available. By contrast, scATS infers TSS usage de novo from RNA reads alone, making it broadly applicable without requiring additional chromatin data. Together, scATS provides a significant technical advance for single-cell TSS profiling across diverse biological contexts.

To demonstrate its performance, we applied scATS to analyse mouse HSPCs and observed dynamic patterns of ATS usage during haematopoiesis that reflect differentiation stage-specific regulatory effects. In the analysis of PBMCs of patients recovered from SARS-CoV-2 infection, TSS usage improved the accuracy and resolution of cell clustering. Furthermore, TSS analysis provided insights into ATS-driven immunoregulation, such as the differential TSS usage of *SEMA4D*. Similarly, scATS identified TSSs that were highly transcribed in the malignant cells of NSCLC, whose host genes were enriched in oncogenic pathways such as NF-κB signalling and T cell differentiation. To further identify key TSSs in lung cancer, we developed the LRS model, which achieved high performance (AUROC = 0.97; AUPRC = 0.99) and provides a novel framework to identify differential TSS usage in tumourigenesis.

We experimentally investigated the roles of ATS isoforms of *CCR6*, *CCR2* and *RTKN2* in lung cancer. Chemokines *CCR6* and *CCR2* were reported to promote lung cancer progression through ERK activation and macrophage recruitment^78,79^. *CCR6-L* was highly transcribed in malignant cells and promoted cell proliferation and migration, while *CCR6-S* and *CCR2-S*, both highly transcribed in non-malignant cells, showed tumour-suppressive effects. Interestingly, both CCR6 and CCR2 isoforms regulated HMGCR expression, a key enzyme in cholesterol metabolism, and were reported to promote lung cancer progression by recruiting immunosuppressive cells and enhancing pro-tumour inflammation^80,81^. Furthermore, we found that *RTKN2-S*, which was highly transcribed in malignant cells, promoted cell proliferation and migration, whereas the *RTKN2-L* showed the opposite effect. *RTKN2* is a Rho GTPase effector, reported to suppress lung adenocarcinoma growth, migration and glycolysis by inactivating NF-κB signalling^82^. *RTKN2-L* inactivated NF-κB signalling by reducing p-p65 (Ser536) level in the nucleus, thereby inhibiting downstream signalling, consistent with previous reports that *RTKN2* inactivated NF-κB activity by suppressing p65 nuclear translocation of LUAD cells^73^. We revealed that RTKN2-L, but not RTKN2-S, binds to SREBP2, inhibiting its nuclear translocation and thus reducing downstream HMGCS1 expression^83^.

Despite the advances, limitations remain in TSS analysis. scATS was optimised for short-read 5’ scRNA-seq, which limits reconstruction of downstream splicing events and complete ATS isoforms. The LRS model is constrained by (1) sufficient positive TSSs associated with cancer or disease conditions for model training; and (2) high-quality 5’ scRNA-seq datasets. Curation with larger datasets of functionally validated ATSs across cancer subtypes would improve its accuracy and application in other cancer types. In this study, we further applied the same modelling strategy to glioblastoma datasets and observed good performance (see Supplementary Note 7), supporting the general applicability of the LRS approach.

Taken together, the integrated scATS-LRS framework establishes a scalable paradigm for single-cell TSS analysis that addresses the biases introduced by RNA degradation and enables the identification of the functional TSSs in NSCLC. By enabling accurate quantification and functional prediction of TSSs, our study provides a foundation for accurate and systematic profiling of single-cell TSSs and uncovering novel transcriptional regulation in malignant cells with the potential to be developed as prognostic biomarkers or even therapeutic targets.

## Methods

All research involving animals was conducted in accordance with relevant ethical regulations and was approved by the Institutional Animal Care and Use Committee of West China Second University Hospital [(2018) Animal Ethics Approval No. 004].

### Section 1: scATS and downstream analysis

#### Overview of scATS pipeline

We developed a stepwise computational method, scATS, to identify TSSs and infer the relative usage of TSSs from 5’ end of scRNA-seq reads (Fig. 1a). In brief, scATS comprises three main steps: (1) TSS detection: identify TSSs of each gene based on the ECDF of the start sites of read 1 (R1), which marked the 5’ end of each sequencing fragment; (2) degradation quantification: quantify the RNA degradation rates ($\alpha$) of each TSS using an EM model by modelling the R1 start site. Additionally, a complementary metric, $\beta$, is calculated as the normalised area under the cumulative distribution curve, representing the RNA integrity for each TSS; (3) transcription correction: an estimation of TSS usage ($\theta$) with correction of RNA degradation, providing a more accurate measure of usage than the raw PSI ($\psi$) value. Strategies for setting key parameter values in the scATS package and evaluation of the impact of sequencing depth are described in Supplementary Note 8.

##### De novo TSS identification

The scATS pipeline begins with TSS identification for each gene by first extracting annotated open reading frames (ORFs) within the first exon of each transcript from the annotation of GENCODE. The aligned paired-end reads and their associated cell barcode tags were retrieved from the BAM files of scRNA-seq data. To ensure specificity and avoid interference from neighbouring genes, we exclusively utilised read pairs for which the corresponding read 2 (R2) was uniquely mapped to the ORFs and first exons of the target gene. This strategy allows for the discovery of novel TSSs outside annotated regions using the R1 mapping position while anchoring the transcript identity to the target gene via the R2 alignment.

The genomic start positions of R1 reads (for the “+” strand) or end positions (for the “−” strand) were used to infer TSSs, as these sites mark the 5’ end of the sequencing fragments. To enhance robustness, scATS provides optional parameters for the users to restrict analysis to uniquely mapped, primarily aligned, and non-duplicated reads. For a more conservative analysis focused on the annotated regions, an additional parameter (UTROnly) allows the users to filter R1 reads to include only those mapped within the annotated 5’ UTR of the target genes.

For each gene with at least *R* (default = 500) aligned read pairs, all R1 initiation sites were sorted according to the transcript direction (from 5’ to 3’). An ECDF was computed to model the cumulative probability of read initiation sites along the gene body using the sorted rank of all original initiation sites. This approach mitigates the confounding influence of spliced intron regions on the distribution. The ECDF ${F}_{n}({{{\rm{x}}}})$ for a given position $i$, is defined as:

$${F}_{n}(i)={{{\rm{P}}}}[{{{\rm{X}}}}\le {{{\rm{x}}}}]=\frac{1}{R}{\sum}_{j=1}^{i}{x}_{j}=\frac{1}{{F}_{n}(L)}{\sum}_{j=1}^{i}{x}_{j},$$

where ${x}_{j}$ is the count of reads that initiate from position $j$, $R$ is the total number of reads of a given gene, and $L$ is the length of expressed base pairs of this gene. The ratio of reads (${x}_{j}/R$) indicates the relative proportion of reads starting from position $j$, which reflects the coverage of raw reads.

To reduce noise and enhance the underlying trends in the data, we employed a Gaussian kernel smoothing filter (with a kernel size of 10) on the raw ECDF values. The smoothed value at position *i* is given by:

$$\widehat{{F}_{n}\left(i\right)}=\frac{1}{\sqrt{2\pi }\sigma }exp \left(-\frac{{({F}_{n}(i)-\mu )}^{2}}{2{\sigma }^{2}}\right)$$

where σ is the standard deviation (SD) of the Gaussian kernel and μ is the mean cumulative distribution probability of the given kernel *w* (default = 10). This technique convolves the input signal with a Gaussian kernel, effectively suppressing high-frequency noise while preserving the overall structure of the data. This process yields a smoothed cumulative distribution probability.

Since the ECDF represents the cumulative probability of read initiation at the observation position, a steeper slope in the ${F}_{n}$ curve indicates a higher local density of reads initiating within that genomic region. To systematically identify these regions of steep ascent, we calculated the first derivative of a smoothed ${F}_{n}$ and detected its local maxima, which we designated as candidate TSSs. The first derivative of the smoothed CDF was calculated using a splinefun function in R. Subsequently, the local maxima of $\hat{{F}_{n}(x){\prime} }$ were extracted as candidate TSSs, as numerous sequencing reads originated from these positions. The raw PSI ($\psi$) of a given TSS can be calculated as:

$${{{{\rm{\psi }}}}}_{t}=\widehat{{F}_{n}\left({i}_{t}\right)}-\widehat{{F}_{n}\left({i}_{t+1}\right)}$$

where ${i}_{t}$ is the position of TSS $t$ and ${i}_{t+1}$ is the position of TSS $t+1$.

Candidate TSSs were subjected to a consecutive filtering pipeline: (1) TSSs were retained only if the derivative value $\hat{{F}_{n}(x)^{\prime} }$ of this position was significantly (default = 0.01) greater than expected under a null model of uniformly distributed reads across the length of a gene ($L$). (2) To avoid the effects of noise and low-transcribed TSSs, TSSs with low abundance were filtered out if their raw usage $\psi$ was below a threshold $r$ (default = 0.05). (3) Optionally, users can retain distally annotated TSSs even if they fail the above filters, ensuring the known regulatory elements are conserved.

Following filtering, the $\psi$ values were recalculated for the final set of TSSs. We then assigned all reads to each TSS based on whether the initiation site of a read falls within the TSS and the corresponding downstream region. Finally, the reads were grouped by their corresponding cell barcode, and the single-cell level of PSI $\psi$ values were calculated for each TSS in each cell.

##### Quantification of ${{{\boldsymbol{\alpha }}}}$ and ${{{\boldsymbol{\theta }}}}$ at the TSS level using the EM algorithm

To quantify RNA degradation at the TSS level, we model the distribution of R1 start sites observed from 5’ scRNA-seq data. For a given gene $g$, R1 reads are grouped into TSS clusters, each representing a putative TSS. Within a TSS cluster $t$ of length ${L}_{{gt}}$, R1 start sites are binned at single base resolution, indexed by $j$. Let ${R}_{{gtj}}$ denote the observed number of R1 reads starting at bin $j$ within TSS cluster t of gene *g*. We assume that the probability of observing an R1 start site at a given distance from the TSS follows an exponential decay^84,85^, leading to an exponentially decreasing pattern in the observed counts ${R}_{{gtj}}$:

1 $${R}_{{gtj}}=c{\theta }_{{gt}}{e}^{-{{{{\rm{\alpha }}}}}_{{gt}}{d}_{{gtj}}},$$

where $c$ is a normalisation constant, ${\theta }_{{gt}}$ represents the relative usage of TSS cluster $t$ for gene *g*, and ${{{{\rm{\alpha }}}}}_{{gt}}$ denotes the TSS-specific RNA degradation rate. The distance ${d}_{{gtj}}$ is defined as the genomic distance, in base pairs, from bin $j$ to the 3’ end of the TSS cluster $t$ (Fig. 1a), and is normalised by the cluster length such that (0 ⩽ ${d}_{{gtj}}$ ⩽ 1).

To formalise the assignment of bins to TSS clusters $t$, we define an index matrix ${{{{\bf{I}}}}}_{g}=\,\{{I}_{{gtj}}\}$, where ${I}_{{gtj}}$ = 1 if bin $j$ belongs to TSS cluster $t$ and ${I}_{{gtj}}$ = 0 otherwise. Based on this notation, we define the effective length of TSS cluster *t* as:

2 $${\omega }_{{gt}}\,\equiv \,{\sum}_{j}{I}_{{gtj}}{e}^{{-\alpha }_{{gt}}{d}_{{gtj}}}.$$

With the notation above, the likelihood of the complete data of a gene can be written as:

3 $${{L}_{g}={\prod }_{t,j}\left(\frac{{\theta }_{{gt}}{\omega }_{{gt}}\frac{{l}_{{gj}}{e}^{{-{{{\rm{\alpha }}}}}_{g}{d}_{{gtj}}}}{{\omega }_{{gt}}}}{{\sum }_{h}{\theta }_{{gh}}{\omega }_{{gh}}}\right)}^{{I}_{{gtj}}{R}_{{gtj}}},$$

note that we aim to maximise $\log {L}_{g}$ as a function of $\left({\theta }_{g1},{\theta }_{g2},\ldots,{\theta }_{{gn}};{\alpha }_{g1},{\alpha }_{g2},\ldots,{\alpha }_{{gn}}\right)$ with the constraint ${\sum }_{t=1}^{n}{\theta }_{{gt}}=1$. The procedure of the EM algorithm is as follows:

**E-step:** Given the current value of ${\varTheta }_{{{{\boldsymbol{g}}}}}^{\left(n\right)}=\left({\theta }_{g1}^{\left(n\right)},{\theta }_{g2}^{\left(n\right)},\ldots,{\theta }_{{gx}}^{\left(n\right)}\right)$ and ${{{{\rm A}}}}_{{{{\boldsymbol{g}}}}}^{\left({{{\boldsymbol{n}}}}\right)}=\left({\alpha }_{g1}^{\left({{{\boldsymbol{n}}}}\right)},{\alpha }_{g2}^{\left({{{\boldsymbol{n}}}}\right)},\ldots,{\alpha }_{{gx}}^{\left({{{\boldsymbol{n}}}}\right)}\right)$ at the $n$ th step, we take the expected value of $\log {L}_{g}$ and let

$${R}_{{gtj}}^{(n)}=E({R}_{{gtj}} | ({R}_{g1},\,{R}_{g2},\ldots,{R}_{{gx}}),{\varTheta }_{g}^{(n)},{{{{\rm{A}}}}}_{g}^{(n)})=\frac{{R}_{{gj}}{I}_{{gtj}}{\theta }_{{gt}}^{(n)}{e}^{{-{{{\rm{\alpha }}}}}_{g}^{(n)}{d}_{{gtj}}}}{{\sum }_{h}{I}_{{ghj}}{\theta }_{{gh}}^{(n)}{e}^{{-{{{\rm{\alpha }}}}}_{g}^{(n)}{d}_{{ghj}}}}$$

**M-step:** We maximise the function $\log {L}_{g}$ and let

4 $${\widehat{\beta }}_{{gt}}^{\left(n+1\right)}=\frac{{\sum }_{j}{I}_{{gtj}}{R}_{{gtj}}^{\left(n\right)}}{{R}_{g}}$$

where ${\beta }_{{gt}}\equiv \,{\theta }_{{gt}}{\omega }_{{gt}}/{\sum }_{h}{\theta }_{{gh}}{\omega }_{{gh}}$ and ${R}_{g}$ is the total cumulative number of start sites of R1 reads from gene $g$ (see Supplementary Note 1 for detailed algorithm).

#### Quantification of ${{{\boldsymbol{\beta }}}}$ for each TSS

For a certain TSS $t$, we also used the ECDF to calculate the cumulative distribution probability of the relative initiation sites of all assigned reads. The $\beta$ value for the TSS is then defined as the normalised area under the cumulative distribution curve:

$$\beta=\frac{\int {Fn}\left(x\right){dx}}{l},$$

where $l$ is the length of the transcribed base pairs of this TSS. A smaller $\beta$ value indicates that a greater proportion of reads were degraded, leading to a more gradual rise of the cumulative distribution curve. In the case of a completely random uniform distribution, the cumulative distribution curve closely follows the diagonal line, and the $\beta$ value approaches 0.5 (Fig. 1a; see Supplementary Note 2).

#### Quantification of ${{{\boldsymbol{\alpha }}}}$ or ${{{\boldsymbol{\beta }}}}$ at the sample level

The overall transcript degradation rate for each sample, denoted as ${D}_{{sample}}$ was computed based on the outputs from the scATS pipeline. For a given sample, let the set of identified $S=\{{{TSS}}_{1},{{TSS}}_{2},\,\ldots,{{TSS}}_{N}\}$, where *N* is the total number of TSSs. The scATS first calculates a specific degradation rate $a$ or $\beta$ for each TSS (${{TSS}}_{{{{\rm{i}}}}}$) in the set $S$. To derive a single, representative metric for the entire sample, we defined the sample-level degradation rate as the arithmetic mean of these individual values. Therefore, ${D}_{{sample}}$ is calculated using the following formula:

$${D}_{{sample}}^{(p)}=\frac{1}{N}{\sum}_{i=1}^{N}{p}_{i},\,p\in \{\alpha,\,\beta \}$$

This approach provides a single, comprehensive value representing the average degradation for all TSSs within the sample.

#### Distribution of the aligned reads

We used the geneBody_coverage.py script of the RSeQC package^26^ to calculate the coverage of mapped reads over the gene body.

#### Epigenetic datasets

**Promoter motifs**. Promoter-related motifs were predicted on the mm10 genome using HOMER^86,87^, including cAMP Response Element, Enhancer Box, E26 Transformation-Specific family motif, Nuclear Factor Y, Nuclear Respiratory Factor, Specificity Protein 1, TATA-binding protein recognition sequence, and Yin Yang 1 motifs. Additional motifs such as Gene Factor X, Gene Factor Y and Gene Factor Y–Selenocysteine tRNA Activating Factor were also identified, which are internal motif labels provided by the HOMER database. Motif enrichment was performed using the findMotifsGenome.pl script with default parameters (-size -50,50, indicating a region of 50 bp centered on the TSS).

**CpG islands**. CpG islands were obtained from the UCSC Table Browser (https://genome.ucsc.edu/).

**Histone modifications, CAGE-seq and ATAC-seq datasets**. Histone modification ChIP-seq (chromatin immunoprecipitation followed by sequencing) datasets were downloaded from ENCODE, including 3 histone marks from mouse multipotent progenitor cells and 6 histone marks from human A549 cell line. CAGE-seq datasets were obtained from the FANTOM5 project (https://fantom.gsc.riken.jp/5/) and included human alveolar epithelial cells and bronchial epithelial cells, as well as mouse stem cells. ATAC-seq datasets were downloaded from ENCODE and included data from the human cerebellum and A549 cell line, and mouse haematopoietic stem cells. The accession IDs are listed in Supplementary Data 10.

**Evolutionary conservation**. Evolutionary conservation scores were obtained from PhastCons tracks downloaded from UCSC Browser (https://genome.ucsc.edu/). Specifically, the analyses on mouse data were based on the mm10.60way.phastCons.bw file, and the analyses on human data used the hg38.phastCons100way file.

#### Clustering and annotation

We used Seurat (v4.0.5)^88^ for the downstream analyses, including data normalisation (NormalizeData function with LogNormalize method, scaling factor of 10,000), data feature scaling (ScaleData function), variable gene detection (FindVariableFeatures function with vst method), and PCA of variable genes (RunPCA function). Next, the original Louvain algorithm (FindClusters function) with a specific clustering resolution was performed to cluster the cells.

#### Benchmark analyses of TSS identification and quantification

We compared scATS with several TSS identification tools, including SCAFE, CamoTSS, TSSr and scTSS (Supplementary Data 2). SCAFE (v1.0.0) and CamoTSS (v0.1.7) were run with their recommended workflows using the matched ATAC-seq datasets corresponding to each 5’ scRNA-seq dataset (Supplementary Data 10). When the output of CamoTSS contained no predicted TSSs, the detection threshold was lowered. TSSr (v0.99.6) was run using the default parameters. scTSS (v0.1.0) relies on an external TSS set to perform TSS cluster quantification and differential TSS usage analysis. Following the recommended workflow, we therefore used the intersection of TSS identification and quantification results from SCAFE (paired-end) and TSSr (R2-based) as the input TSS set for scTSS-based analyses. All command-line parameters, input files and implementation details are provided in Supplementary Note 4.

**Distance analysis**. We compared the inferred TSSs with annotations in GENCODE and refTSS (http://reftss.clst.riken.jp/). For each predicted TSS, the distance to the nearest annotated TSS was calculated using the distanceToNearest function of the GenomicRanges package (v1.38.0)^89^ in R. Predicted TSSs located within ±50 bp of a reference TSS were considered annotated, while others were classified as novel.

**Overlap analysis**. To account for positional variations between the different algorithms, we first expanded the distance to each TSS. Using the resize function from the GenomicRanges package (v1.38.0)^89^, each TSS was symmetrically extended by 50 bp upstream and downstream. We define the extended sets as *S*. Next, all *S* sets from the three methods were combined using the reduce function, which merged any overlapping intervals to create a final, non-redundant master set of TSSs, defined as *T*. To quantify the number of TSSs detected by different methods, we used the findOverlaps function to determine the overlaps between each *S* set of different methods and the combined *T* set. This step allowed us to quantify the number of loci in the combined set *T* that were identified by each method. The results were visualised using a Venn diagram created with the ggvenn (v0.1.10), which illustrates the number of TSSs that were uniquely or jointly identified by the three methods.

**Performance evaluation**. GENCODE-annotated TSSs were used as the reference. A predicted TSS is considered as a match if it is located within a cutoff, i.e., within a specified distance around the nearest annotated TSS. Precision was defined as the proportion of matched TSSs among all predicted TSSs, recall as the proportion of matched TSSs among all annotated TSSs, and the F1 score as the harmonic mean of precision and recall.

#### Benchmark analyses of RNA degradation rate

To benchmark RNA degradation rate estimation, we compared scATS with DeepKINET (v0.2.0), a gene-level kinetic modelling framework. Because DeepKINET operates at the gene level and does not model TSS-specific degradation, we restricted the comparison to genes with a single annotated TSS on simulated 5’ scRNA-seq datasets, to ensure a fair and interpretable benchmark. Spliced and unspliced read counts were generated using the Python package velocyto (v0.17.17)^90^, with the run10× mode and a masked GTF file. The resulting loom files were processed with DeepKINET to estimate splicing and degradation rates at the gene level. For each dataset, cells were filtered and normalised, and the top 2000 genes were selected for model fitting following the official tutorial.

#### Differential genes and TSSs analysis

Cell-type-specific genes were identified using the FindAllMarkers function of Seurat with default parameters on the normalised data. Differential genes with *p* values < 0.05 (Wilcoxon test) and absolute log2 fold change > 0.25 were used for further analysis.

FindMarkers function in scATS was applied to the PSI ($\psi$ or $\theta$) matrix to identify cell-type-specific TSSs. Differential TSSs were defined by the Wilcoxon test with *p* < 0.05 and absolute Δ $\psi$ or $\theta$ > 0.1. Two comparison modes were implemented: (1) 1-vs-1, comparing two cell types that both transcribe the given TSS, and (2) 1-vs-rest, comparing a target cell-type against all other cell-types combined.

#### Multi-modal integration of the mouse haematopoietic stem and progenitor cell (mHSPC) 5’ scRNA-seq dataset

We performed unsupervised multi-omics integration analyses using multi-omics factor analysis (MOFA) by the MOFA + R package (v1.10.0)^35^, which enables variance decomposition of the datasets and factors using the coefficient of determination. We followed the developer’s directions for model selection and downstream multi-omics analysis.

#### Dimensionality reduction and visualisation of pseudo-bulk profiles

To visualise the clustering among pseudo-bulk cell-types, we performed non-linear dimensionality reduction using the t-SNE^36,37^ algorithm on the pseudo-bulk expression matrix. The computation was carried out using the Rtsne package in R, with the random seed set for reproducibility. A key parameter, perplexity, was dynamically adjusted to a conservative value suitable for the small number of input cell-types. The resulting two-dimensional coordinates were then visualised as a scatter plot using the ggplot2 package (v3.5.1)^91^, where each point represents a distinct cell-type. To enhance clarity, the ggrepel package (v0.9.5) was utilised to prevent overlapping of the cell-type labels.

#### Gene ontology (GO) analysis

The host genes of TSS were fed into the clusterProfiler R package (v3.14.3)^92^ to perform GO enrichment analysis with default arguments. Pathway enrichment network plots were visualised using the enrichmentNetwork function from the aPEAR package (v1.0)^93^.

#### Definition of major and minor ATSs

We classified the TSSs into three distinct categories based on their relative usage. Our analysis was focused on the genes that possess two TSSs. For these genes, we first calculated the $\theta$ value for each TSS of a specific group of cells. For a given gene, the TSS with the highest $\theta$ within that group was then designated as “major TSS”, correspondingly, the TSS with a lower $\theta$ was classified as “minor TSS”.

#### Genomic region analysis

To characterise the genomic distribution of the TSSs, we performed a genomic feature analysis using the annotatePeak function from the ChIPseeker Bioconductor package (v1.22.1)^94,95^. This function takes coordinates of a set of TSS coordinates as input and annotates each site based on its location relative to genomic features derived from the TranscriptDb (TxDb) annotation from GENCODE. The primary genomic features include the promoter, 5’ UTR, 3’ UTR, exons, introns, downstream regions, and distal intergenic regions.

#### SCENIC gene regulation network analysis

To examine the regulatory activity of TF, we applied the single-cell regulatory network inference and clustering (pySCENIC, v0.12.1)^41^ by using normalised expression matrices of 5’ scRNA-seq. The pipeline comprises three main steps: infer GRN based on co-expression patterns, predict regulon based on motif discovery (cisTarget), and quantify the predicted regulon activity with AUCell scores. The regulon connection specificity index matrix was calculated^96^. Using this method, we compared (a) the host genes carrying gain or loss TSSs in the mHSPCs dataset, (b) host genes with differential TSSs usage in naïve CD8 T cells in the COVID-19 dataset, and (c) host genes with differential TSSs usage in malignant cells in the NSCLC dataset.

#### Weighted nearest neighbour (WNN) analysis

Gene and TSS expression were integrated using the WNN approach^44^ implemented in Seurat (v4.0.5)^88^. Multimodal neighbours were identified using the FindMultiModalNeighbors function, and clustering was performed on the weighted shared nearest neighbour graph (algorithm = 3, resolution = 1). UMAP embeddings were computed for the WNN graph as well as for individual gene and TSS modalities.

### Section 2: data used in this study

#### Preparing simulated datasets with RNA degradation

To assess the performance of scATS, we simulated data that mimics real 5’ scRNA-seq as follows:

1. Genes were sampled from the reference set based on the reference genome (GRCh38), and only protein-coding positive-strand genes were selected.
2. The numbers of transcripts owned with length $\ge$ 500 nt from gene set were $\in \left\{1,\,2\right\}$, and transcripts with close ATS (200 nt) were excluded.
3. Expressed transcripts were sampled from a gene set with expression levels drawn from a normal distribution $N\left(0.8,\,0.2\right)$, and transcript counts per cell were generated using a negative binomial distribution.
4. The sequences of R1 and R2 were extracted from gene set mentioned before, with the length 111 nt and 150 nt respectively.
5. Start sites of R1 were determined by sampling from a discrete probability distribution, with 90% of the probability assigned to the primary transcription start site and 10% distributed across downstream positions. The end sites of R2 followed normal distribution $N\left(400,\,50\right)$.

After generating the simulated paired-end FASTQ files, Cell Ranger (v4.0.0) was applied to generate aligned BAM files, which were used as input for degradation simulation to introduce transcript-specific RNA degradation as follows:

1. Only read pairs whose R1 reads were mapped to annotated genes were subjected to RNA degradation simulation, whereas all other read pairs were retained unchanged.
2. The R1 start position was resampled by drawing a distance from the original TSS according to the exponential decay model defined in formula 2, which was parameterised by a gene- and TSS-specific degradation rate *α*. In the absence of RNA degradation (*α* = 0), all R1 starts were retained at the original TSS (*d* = 0), whereas increasing values of *α* shifted probability mass toward larger downstream distances (*d* > 0).

- Without degradation
  In the absence of RNA degradation (*α* = 0), all probability mass was concentrated at the TSS:
  $$P\left(d=0\right)=1,$$
  where *d* represents the distance between the observed R1 start site and the corresponding TSS.
- With degradation

Conditional on the read not starting at the TSS (*d* > 0), downstream distances follow a truncated exponential distribution:

$$P\left(d={k|d} > 0\right)\propto {e}^{-\alpha k},\,k=1,\,\ldots,\,W$$

where W represents the size of the degradation window. Consequently, the resulting R1 start-site distance distribution exhibits a peak at the TSS followed by an exponentially decaying tail, reflecting progressive 5’ RNA degradation.

3) The R2 start position was recalculated based on the original fragment length to prevent the generation of invalid or inconsistent paired-end alignments following R1 repositioning.
4) All resampled read pairs were written to a new BAM file, which was subsequently sorted and indexed for downstream analyses.

Detailed implementation and parameter settings are provided in Supplementary Note 3.

#### Published datasets

The published datasets used in this study are listed in Supplementary Data 1. For the analysis of mouse HSPCs (mHSPCs), we used two Smart-seq2 datasets: an in-house^25^ dataset and the other from Tabula Muris consortium^39^, and two in-house long-read datasets, including ONT and PacBio^25^. We also incorporated a 5’ scRNA-seq dataset generated from PBMCs of female patients recovered from COVID-19^24^, which was prepared following the Chromium Next GEM Single Cell 5’ Reagent Kits v2 protocol (CG000331 Rev E). Libraries were sequenced on the BGI MGISEQ-2000 platform as 150 bp paired-end dual-indexing (150 cycles R1 with 16 cycles 10× Barcode and 10 cycles UMI, 150 cycles R2). In addition, we analysed a publicly available 10× Genomics 5’ scRNA-seq dataset generated from a fresh surgical resection of an NSCLC patient (https://www.10xgenomics.com/datasets/nsclc-tumor-5-gene-expression-1-standard-2-2-0), following the Single Cell V(D)J Reagent Kits protocol (CG000086 Rev C). Libraries were sequenced on the Illumina NovaSeq 6000 platform as 150 bp paired-end single-indexing (150 cycles R1 with 16 cycles 10× Barcode and 10 cycles UMI, 150 cycles R2).

#### Generation of scRNA-seq and scONT datasets of mHSPCs

The female C57BL/6J mice (*n* = 2) were purchased from Jiangsu JCYK Bioscience Co., Ltd. (Jiangsu, China), housed and bred under SPF conditions (Specific Pathogen Free) at the Laboratory Animal Center of West China Second University Hospital, and had *ad libitum* access to food and water. Animal experiments were conducted as described above.

**Isolation of mHSPCs**. The bone marrow cells were obtained from the femur and tibia of C57BL/6 mice aged about 8 weeks and filtered through a 70 μm cell strainer (Corning, #431751). After centrifuging at 300 g for 5 min at 4 °C, the cells were resuspended in 200 μL EasySep Buffer and CD117 MicroBeads (20 μL/mouse) (Miltenyi Biotec, #130-097-146). The whole solution was transferred to a 1.5 mL Eppendorf tube and incubated on ice for 20 ~ 30 min. A volume of 1 mL sample was added to the MS separation column. The MS separation column was washed with EasySep Buffer for 3 times on the Magnetic Separation Rack, and then the MS separation column (Miltenyi Biotec, #130-042-401) was eluted with 5 mL of EasySep Buffer from the Magnetic Separation Rack to obtain lineage negative (LIN^−^) cells. A volume of 20 μL cells was taken and counted, and the remaining samples were centrifuged at 300 g for 5 min at 4 °C. SCM (+) medium was used to resuspend (4 mL/mouse) and then placed in an incubator at 37 °C with 5% CO₂ for 12–24 h.

**mHSPC scRNA-seq**. For 5’ scRNA-seq, single cells were prepared in the Chromium Single Cell Gene Expression Solution using the Chromium Single Cell 5’ Gel Bead, Chip, and Library Kits v2 (10× Genomics) according to Chromium Next GEM Single Cell 5’ Reagent Kits v2 (CG000331 Rev E). We generated a 5’ Cellular Indexing of Transcriptomes and Epitopes by Sequencing (CITE-seq) dataset of mouse LIN^−^ cells from bone marrow, consisting of 388 cells and 8 ADTs, including anti-Ly6A, anti-CD16, anti-CD71, anti-CD117, anti-CD135, anti-CD34, anti-CD127 and anti-CD41. Approximately 10,000 cells were loaded, with about 9000 expected cells recovered and 6200 cells detected. Libraries were sequenced on the Illumina NovaSeq 6000 platform as 150 bp paired-end dual-indexing (150 cycles R1 with 16 cycles 10× Barcode and 10 cycles UMI, 150 cycles R2) at Novogene, Beijing, China.

For 3’ scRNA-seq, single cells were prepared using the Chromium Single Cell 3’ Gel Bead, Chip, and Library Kits v2 (10× Genomics) according to the manufacturer’s protocol, and libraries were sequenced on the Illumina NovaSeq 6000 platform at Novogene, Beijing, China.

**Single-cell Oxford Nanopore Technology sequencing (scONT)**. The mouse LIN^−^ cells (2 × 10^5^ cells/mL) suspended in PBS were loaded onto the microwell chip using the Singleron Matrix® Single Cell Processing System. Barcoding beads were subsequently collected from the microwell chip, followed by reverse transcription of the captured mRNA on the barcoding beads to obtain full-length cDNA and PCR amplification. The amplified full-length cDNA was then ligated with sequencing adapters using the GEXSCOPE® Single Cell RNA Library Kits (Singleron, #4180011)^97^ in combination with the SQK-LSK109 kit (Oxford Nanopore Technologies). Individual libraries were diluted to 4 nM, pooled, and sequenced on a PromethION flow cell following the ONT recommended procedure. On average, 8 samples were loaded per PromethION chip, and sequencing was continued for 72 h until all available wells were exhausted, with raw Fast5 electrical signal data collected for downstream analysis.

#### Analyses of transcriptomic datasets

**scRNA-seq data of mHSPCs, COVID-19 and NSCLC**. Reads were processed using Cell Ranger (v4.0.0) with default parameters. Both 5’ scRNA-seq and 3’ scRNA-seq datasets of mHSPC were mapped to the mouse genome (mm10), while the COVID-19 and NSCLC datasets were mapped to the human genome (hg38). Low-quality cells were filtered by the following criteria: mHSPCs (gene count <400, mitochondrial ratio > 25%), COVID-19 (gene count <500, mitochondrial ratio > 20%), and NSCLC (gene count <300, mitochondrial ratio > 15%). Information on the datasets is provided in Supplementary Data 1.

**mHSPC Smart-seq2 datasets**. Analysis was performed using the Seurat (v4.0.5)^88^ package. For quality control, cells were filtered using dataset-specific thresholds: in-house dataset (gene count <500 or unique molecular identifier [UMI] count <50,000) and Tabula Muris dataset (gene count <500 or > 8000, UMI count <70,000 or > 5,000,000). The filtered datasets were then processed through the standard Seurat workflow, including data normalisation, variable gene selection, dimensionality reduction, clustering, and visualisation to identify distinct cell-types for the downstream analyses.

**mHSPC scONT, ONT and PacBio dataset**. The Pychopper (v2.5.0) was used to identify, orient and trim long-read sequencing reads with default parameters. The number of reads, read length and quality were analysed using NanoComp (v1.33.1)^98^ using the “-raw-store-tsv_stats” and visualised using the R package ggplot2 (v3.5.1)^91^. Pychopper-processed reads were aligned to the mm10 genome using minimap2 (v2.17)^99^ with the following parameters: minimap2 -ax splice -uf -secondary = no -C5 --MD. Then, mismatches, insertions, deletions, and non-canonical splice sites in aligned reads were corrected using TranscriptClean (v2.0.3)^100^. Finally, TranscriptClean-corrected reads were aligned again to the mm10 genome.

**Enrichment analysis**. We downloaded 879 vertebrate TF motifs from JASPAR CORE 2024^101^ (https://jaspar.genereg.net/). The sequences of 1000 bp upstream and downstream of each TSS were extracted using bedtools getfasta (v2.26.0)^102^. TF motifs occurring within these TSS sequences were then identified using FIMO (v4.11.2, MEME-suite)^103^. Occurrences with *q* values < 0.05 were considered significant. R package ggseqlogo (v0.1)^104^ was used to generate the sequence logo of FIMO-searched sequences.

### Section 3: lung cancer relevance scores (LRS)

We developed a machine-learning model that utilises a collection of cancer-related promoters^8^ to predict lung cancer relevant TSSs in the NSCLC 5’ scRNA-seq dataset using the RF algorithm.

The framework of the LRS model consisted of five key steps:

#### Step 1: feature extraction

We extracted 33 features from the identified TSSs falling into four categories, including 19 features of regulatory elements, 8 features of TSS quantification, 3 features of RNA degradation and 3 transcript features as listed in Supplementary Data 7.

##### Regulatory elements

We included 12 histone modification features, each represented by the signal directly at the TSS (H3K4me3_Onsite, H3K4me1_Onsite, H3K27ac_Onsite, H3K27me3_Onsite, H3K36me3_Onsite, POLR2A_Onsite) and the average signal within ±2500 bp (H3K4me3_Avg, H3K4me1_Avg, H3K27ac_Avg, H3K27me3_Avg, H3K36me3_Avg, POLR2A_Avg). TF motifs were assessed using FIMO (v4.11.2, MEME-suite) within ±1000 bp of each TSS, including total counts (TF_Count) and the mean and SD enrichment scores (TF_Avg, TF_SD). CpG-related features included the number of CpG sites (CpG_Count) and distances of TSSs to the nearest CpG islands (CpG_Distance). Conservation features were based on PhastCons scores, calculated based on the TSSs (PhastCons_Onsite) and the average within ±2500 bp of TSSs (PhastCons_Avg).

##### TSS quantification

The ratio of reads (Ratio) was calculated as the number of R1 reads initiating at the TSS divided by the total R1 reads of a given gene. We also included the global raw PSIs ($\psi$) and corrected PSIs ($\theta$), and $\theta$ values calculated across different cell-types (Epithelial, Mast and B) and epithelial subclusters (Malignant and Non-malignant) of all TSSs.

##### RNA degradation

Three features were included: RNA degradation metrics, $\alpha$ and $\beta$, and the spread of R1 reads initiating at the TSS (Region).

##### Transcript features

Using the TSSs from the hg38 genome as ground truth, we calculated the distance between TSS and the ground truth (Distance), transcript strand orientation (Strand), and coding status (Coding) of TSS transcripts.

#### Step 2: defining the positive and negative datasets

Promoters associated with cancer patient survival were downloaded from a large-scale pan-cancer transcriptome study^8^. A promoter is defined as positive if it overlaps with a TSS identified in our study within ±50 bp. These positive TSSs were used to construct the training dataset for the RF model (Supplementary Data 6).

To better address the imbalance between positive and unlabeled samples during training, we used the PU (positive-unlabeled) learning method^105^. The specific steps are as follows. We refer to the just-obtained positive data as a ground truth, and the remaining TSSs were referred to as unlabeled. First, we set a sampling proportion of 0.05, extract this proportion of ground truth and unlabeled samples as Us, and the remaining ground truth samples as Ps. Second, we used the ovun.sample function in the ROSE package (v0.0.4)^106^ for oversampling, with the oversampling positive sample fraction parameter (*p* = 0.5) and a random seed (seed = 1). Next, we used a Naive Bayes classifier for classification prediction, to train the model on the oversampled dataset and generate prediction probabilities for the unlabeled samples. Finally, we used the quantile function to select a threshold based on the prediction probabilities and split the dataset into the final positive and negative samples for further model training or analyses (see Supplementary Note 5).

#### Step 3: tenfold cross-validation

In this study, we used a tenfold cross-validation method^107^ to evaluate and improve the performance of the LRS model. For tenfold cross-validation, the dataset was randomly partitioned into 10 equal-sized subsets with the same proportion of positive and negative subsets. Each subset was used as a validation set, while the remaining subsets were used to train the LRS model. This process was repeated 10 times, with each subset acting as the validation set once, to ensure that every data point was used for both training and validation.

#### Step 4: model training

We hypothesise that the positive and negative sets are separable in the multidimensional feature space, implying that a robust model can be trained to identify relevant TSSs. However, given the high dimensionality of multi-omics features, there exists a risk of overfitting. We therefore employed an RF model, which combines bootstrap aggregation (bagging) and feature randomisation to improve generalisation (see Supplementary Note 6).

First, the RF framework leveraged bagging to reduce model variance. For the training dataset $D$, $T$, bootstrap samples ${D}_{1},\,{D}_{2},\,\ldots,\,{D}_{T}$ were generated by randomly selecting $n$ samples with replacement. Each bootstrap subset ${D}_{i}$ is defined as:

$${D}_{i}=\left\{\left({x}_{1}^{*},\,{y}_{1}^{*}\right),\,\left({x}_{2}^{*},\,{y}_{2}^{*}\right),\,\ldots,\left({x}_{n}^{*},\,{y}_{n}^{*}\right)\right\},$$

where $({x}_{j}^{*},{y}_{j}^{*})$ is independently drawn from $D$ (with replacement). This process ensures diversity among individual decision trees by training each tree on a unique subset of the data, thereby decorrelating their predictions. The final prediction is derived through majority voting for classification, effectively stabilising the model against overfitting.

Second, RF introduces $m$ feature randomisation to further decorrelate trees and enhance robustness. At each node split within a decision tree, a random subset of features is selected from the total $p$ features, where $m$ is typically set to $\sqrt{p}$. This forces individual trees to explore distinct predictive patterns in the feature space rather than relying on dominant features. Mathematically, for a feature set $F$, the subset ${F}_{s}$ used at a split is constrained as:

$${F}_{s}\subset F,\,\left|F\right|=m$$

By limiting the features available for splitting, the model prioritises interactions among less-correlated features, reducing the likelihood of overfitting to noise in high-dimensional multi-omics data. Additionally, the RF framework quantifies feature importance by aggregating impurity reductions across all trees. For a feature ${F}_{j}$, its importance is computed as:

$${Importance}\left({F}_{j}\right)=\frac{1}{T}{\sum }_{I=1}^{T}{\sum }_{t\in {T}_{i}:v\left(t\right)={F}_{j}}\frac{{n}_{t}}{n}{I}_{t},$$

where ${T}_{i}$ represents the *i*-th tree, $v\left(t\right)$ is the feature used at node $t$, ${n}_{t}$ is the number of samples at node $t$, and ${I}_{t}$ is the impurity reduction. This metric highlights critical features for distinguishing TSSs between positive and negative datasets.

To complete the modelling process, scikit-learn 1.3.2 was used in Python 3.8.20. The hyperparameters n_estimators, max_depth, and max_samples were optimised using grid search based on the AUROC (see Supplementary Note 6). Performance of the model was evaluated using tenfold cross-validation, and evaluation metrics included AUROC and AUPRC:

$${TPR}=\frac{{{{\rm{TP}}}}}{{{{\rm{TP}}}}+{{{\rm{FN}}}}}$$

$${FPR}=\frac{{{{\rm{FP}}}}}{{{{\rm{FP}}}}+{{{\rm{TN}}}}}$$

$${Precision}=\frac{{{{\rm{TP}}}}}{{{{\rm{TP}}}}+{{{\rm{FP}}}}}$$

$${Specificity}=\frac{{{{\rm{TN}}}}}{{{{\rm{TN}}}}+{{{\rm{FP}}}}}$$

$${AUROC}=\int _{0}^{1}{TPR}\left(t\right)d\left({FPR}\left(t\right)\right)$$

$${AUPR}C=\int _{0}^{1}{Precision}\left(t\right)d\left({Recall}\left(t\right)\right)$$

#### Step 5: scoring and ranking

To predict the potential of unknown TSSs, the predict_proba method of the trained pipeline was employed. For each unknown TSS, it was passed through every decision tree (100 trees in total) in the RF. The probability from a single tree was determined by the proportion of positive sets in the terminal leaf node where the sample landed. The final “Probability” score for each TSS was then calculated as the average of the probabilities predicted by all individual trees in the forest (Supplementary Data 9). This score represents the confidence of model that the TSS belongs to the positive sets. After determining the probability scores for all TSSs, we ranked and classified the TSSs based on these scores, allowing us to identify and select potential candidate TSSs that might be relevant in lung cancer.

#### Feature importance and ablation

**Gini score**. To evaluate the contribution of each feature to the classification model, we utilised the Gini score, also known as Mean Decrease in Impurity, which is intrinsically calculated during the training of the RF classifier. The importance of a feature is computed as the total reduction in Gini score brought by that feature, averaged over all trees in the forest (100 trees in this study). This score decrease at each split node is weighted by the number of samples reaching that node. The final scores for all features are normalised to sum to one, with a higher score indicating a greater contribution to the classification decisions of the model (Supplementary Data 7).

**Feature ablation study**. To refine the input feature set, we conducted feature ablation analysis using the baseline RF model. All of the 33 features were first ranked by their Gini scores, we sequentially removed features according to their Gini score one at a time, and the performance of the model was assessed after each removal. If performance was not improved for the next five consecutive removals, this performance was deemed as optimal and the corresponding features yielding this performance were used as the final set of features for downstream analyses (Supplementary Data 8).

### Comparison models

In addition to the RF model, we implemented and evaluated three other classification algorithms: a SVM, LR and a MLP neural network. All models were implemented using scikit-learn (v1.3.2) in Python. For all models, features were standardised before training by removing the mean and scaling to unit variance.

#### Support vector machine (SVM)

The SVM aims to find an optimal separating hyperplane that best distinguishes between classes. For a given feature vector $x$, the decision function is formally expressed as:

$$f\left(x\right)={{\mbox{sign}}}\left({w}^{T}{{{\rm{\phi }}}}\left(x\right)+b\right)$$

where $w$ is a weight vector, $b$ is a bias term, and $\phi$ is a kernel function that maps the input features into a higher-dimensional space. We employed a non-linear kernel to capture complex patterns in the data. The model was implemented using the *SVC* class from the scikit-learn library.

#### Logistic regression (LR)

LR is a linear model that calculates the probability of a binary outcome. It uses the logistic (sigmoid) function to map the linear combination of input features to a probability value between 0 and 1. The probability of the positive class (*y* = 1) is modeled as:

$$P\left(y=1 | x\right)=\frac{1}{1+{e}^{-\left({w}^{{Tx}}+b\right)}}$$

where $w$ represents the feature weights and $b$ is the intercept. To counteract potential biases from class imbalance, the model was configured to adjust class weights during training. This was implemented using the LogisticRegression class from the scikit-learn library.

#### Multi-layer perceptron (MLP) neural network

An MLP was implemented to learn potentially complex, non-linear relationships in the data. An MLP is a feedforward artificial neural network composed of an input layer, one or more hidden layers, and an output layer. The output of a single neuron in a hidden layer is calculated as:

$$h\left(x\right)={{{\rm{\sigma }}}}\left({W}^{{Tx}}+c\right)$$

where $x$ is the input vector from the previous layer, $W$ is the weight matrix, $c$ is the bias vector, and $\sigma$ is a non-linear activation function (e.g., ReLU). The final output is generated after the signal propagates through all layers. Our implementation utilised the MLPClassifier class from the scikit-learn library.

### Section 4: experimental analyses of ATSs

#### Cell culture

A549, Calu-1 and HEK293T were originally obtained from the American Type Culture Collection and were cultured in Dulbecco’s Modified Eagle Medium (DMEM; Gibco, #C11995500BT) supplemented with 10% Fetal Bovine Serum (FBS; YEASEN, #40130ES76) and 1% Penicillin-Streptomycin (Hyclone, #SV30010) at 37 °C with 5% CO₂.

#### Plasmid construction

Full-length cDNA sequences of human *RTKN2-L* and *RTKN2-S* were synthesised and codon-optimised by Tsingke Biotechnology and subcloned into the lentiviral expression vector, pLV3-CMV-3 × Flag-MCS-EF1a-mCherry-Puro (MiaoLing Biology, #P61857). Similarly, the *Catalase* (*CAT*) and isoforms of *CCR6-L*, *CCR6-S* and *CCR2-S* were amplified from cDNA, and the corresponding 5’ UTRs were amplified from genomic DNA of HEK293T and also cloned into pLV3-CMV-3 × Flag-MCS-EF1a-mCherry-Puro. cDNA sequences of human *SREBP2* and *HMGCS1* were amplified from HEK293T cDNA and cloned into lentiviral gene expression vectors pCDH-CMV-MCS-EF1-Neo (MiaoLing Biology, #P13674).

For transient transfection, full-length cDNA sequences of *CAT*, *RTKN2-S* and *RTKN2-L* were cloned into the mammalian expression plasmid pcDNA3.1-3 × Flag (Addgene, #182494), and the *SREBP2* cDNA sequence was cloned into plasmid pcDNA3.1-3 × Myc-C (MiaoLing Biology, #P14395).

Primers, DNA sequences and the plasmid information were listed in Supplementary Data 11.

#### Viral transduction

Lentiviruses were produced by co-transfection of lentiviral expression vectors with psPax2 (Addgene, #12260) and pMD2.G (Addgene, #12259) using LipoMax DNA reagent (SUDGEN, #32012) and OptiMEM (Gibco, #51985-026) in HEK293T cells. The viral supernatants were harvested at 24 and 48 h post-transfection and subsequently filtered through 0.45 μm cell strainers (BIOFIL, #FPE404013). Calu-1 or A549 cells (10^6^) were seeded in 6-well plates and incubated overnight with filtered viral supernatants supplemented with 4 μg/mL polybrene (YEASEN, #40804ES76). The culture medium was then replaced with fresh DMEM, and cells were maintained for an additional 2 days before being subjected to selection with puromycin (YEASEN, #60209ES10) or G418 (YEASEN, #60220ES03) for 3 days.

#### Transient transfection

HEK293T cells were transfected with mammalian expression plasmids using LipoMax DNA reagent (SUDGEN, #32012) and OptiMEM (Gibco, #51985-026) and were harvested at 48 h post-transfection for immunoprecipitation.

#### Transwell cell assay

A number of 10^5^ lentiviral transduced Calu-1 or A549 cells were seeded into transwell inserts (Corning, #3422), while the lower chamber contained 500 μL DMEM with 10% FBS. After 24 h culture at 37 °C, non-invading cells were removed, and the migrated cells were fixed with 4% PFA (Beyotime, #P0099) and stained with 0.1% crystal violet (Sigma, #C0775). The fixed and stained transwells were imaged using an inverted microscope (Olympus microscope, #CKX53). The numbers of migration cells were counted in three random 40× fields per insert using ImageJ (v1.52a)^108^ (National Institutes of Health, Bethesda, MD).

#### Cell proliferation assay

Lentiviral transduced A549 or Calu-1 cells were placed in 96-well plates at a density of 2000 cells per well in four replicates and assayed using the CCK-8 kit (YEASEN, #11141ES10). At 0, 24, 48, 60, 72 and 96 h after culture, 10 μL of CCK-8 reagent was added in each well for 1 h at 37 °C. The absorbance of each well was detected at 450 nm using a microplate spectrophotometer (Biotek, #ELX800).

#### qPCR

Total RNA was isolated from the cultured cells using TRIzol reagent (Thermo Fisher Scientific, #15596018), followed by cDNA synthesis using Hifair® Ⅲ 1st Strand cDNA Synthesis SuperMix for qPCR (YEASEN, #11141ES10). RT-qPCR assays were conducted using SYBR mix (YEASEN, #11201ES08), and the relative gene expression levels were calculated using the 2^−ΔΔCt^ method and normalised to GAPDH. Primers were listed in Supplementary Data 11.

#### Immunoprecipitation

Cells were lysed in Cell Lysis Buffer (Cell Signalling Technology, #9803). Cell lysates were incubated with Pierce™ anti-DYKDDDDK (Thermo Fisher Scientific, #A36797) overnight at 4 °C, washed with PBS for 3 times, pelleted and resuspended in 2 × SDS-polyacrylamide gel electrophoresis (PAGE) Protein Loading Buffer (YEASEN, #20315ES05). Eluted proteins and the input cell lysates were denatured at 95 °C for 5 min before being separated by 10% SDS-PAGE and transferred to PVDF (Millipore, #ISEQ00010). The immunoblots were probed using the following antibodies: rabbit anti-Myc-Tag antibody (Cell Signalling Technology, #2278) and rabbit anti-Flag-Tag antibody (Cell Signalling Technology, #14793). The chemiluminescence signal was detected using a CLINX Imaging System (CLINX, # ChemiScope S6).

#### Western blot analysis

Cells were lysed with RIPA Lysis Buffer (Thermo Fisher Scientific, #89901) supplemented with protease inhibitor cocktail PMSF (Thermo Fisher Scientific, #36978) at a final concentration of 0.1% and pelleted at 12,000 × *g* at 4 °C for 20 min. The fractions of nucleus and cytoplasm were prepared using an NE-PER Nuclear Cytoplasmic Extraction Reagent kit (Thermo Fisher Scientific, #78835) following the manufacturer’s instructions.

Protein extracts were separated by 10% SDS-PAGE and transferred to PVDF (Millipore, #ISEQ00010). The blots were probed with the primary antibodies as follows: anti-rabbit Flag-Tag antibody (1:1000) (Cell Signalling Technology, #14793), anti-rabbit SREBP2 antibody (1:1000) (Abcam, #ab30682), anti-mouse HMGCR antibody (1:1000) (Abcam, #ab242315), anti-rabbit HMGCS1 antibody (1:1000) (Cell Signalling Technology, #42201), anti-rabbit FASN antibody (1:1000) (Cell Signalling Technology, #3180), anti-mouse LDLR antibody (1:1000) (Proteintech, #66414), anti-rabbit NF-κB p65 antibody (1:1000) (HuaBio, #HA0815), anti-mouse IκBα antibody (1:1000) (BioVision, #3252), anti-rabbit phospho-NF-κB p65 (Ser536) (1:1000) (Cell Signalling Technology, #3033), anti-mouse GAPDH antibody (1:1000) (Cell Signalling Technology, #2118), anti-rabbit CCR2 antibody (1:1000) (Proteintech, #30420) and anti-rabbit Lamin B1 antibody (1:1000) (Cell Signalling Technology, #13435) in the universal antibody diluent (NCM biotech, #WB500D) at 4 °C overnight, washed 3 times with TBST, and then incubated with the anti-mouse IgG HRP (1:5000) (Cell Signalling Technology, #7074) or anti-rabbit IgG HRP (1:5000) (Cell Signalling Technology, #7076). The chemiluminescence signal was detected using a CLINX Imaging System (CLINX, # ChemiScope S6).

### Reporting summary

Further information on research design is available in the Nature Portfolio Reporting Summary linked to this article.

## Supplementary information

Supplementary Information

Description of Additional Supplementary Files

Supplementary Data 1

Supplementary Data 2

Supplementary Data 3

Supplementary Data 4

Supplementary Data 5

Supplementary Data 6

Supplementary Data 7

Supplementary Data 8

Supplementary Data 9

Supplementary Data 10

Supplementary Data 11

Reporting Summary

Transparent Peer Review file

## Source data

Source Data

## Data Availability

The raw 5’ and 3’ mHSPC scRNA-seq and scONT data generated in this study have been deposited in the Gene Expression Omnibus (GEO) database under accession code GSE302632. The raw sequencing data of in-house mHSPC datasets generated using Smart-seq2, ONT, and PacBio are available in the European Nucleotide Archive (ENA) database under accession code PRJNA706066^25^ (https://www.ebi.ac.uk/ena/browser/view/PRJNA706066). The raw Smart-seq2 data of mHSPCs from the Tabula Muris consortium used in this study are available in the GEO database under accession code GSE109774^39^ (https://www.ncbi.nlm.nih.gov/geo/query/acc.cgi?acc=GSE109774). The raw 5’ scRNA-seq data of human NSCLC sample used in this study is available from 10× Genomics (https://s3-us-west-2.amazonaws.com/10x.files/samples/cell-vdj/2.2.0/vdj_v1_hs_nsclc_5gex/vdj_v1_hs_nsclc_5gex_fastqs.tar). The processed data of COVID-19 5’ scRNA-seq datasets^24^ used in this study, together with the resources required to reproduce the LRS analysis, have been deposited in the Figshare repository (10.6084/m9.figshare.31857199). All newly generated plasmids and other relevant materials are available upon request from the corresponding author. Source data are provided with this paper.

## Code availability

The scATS code, documentation, and tutorials are available on GitHub^109^ (https://github.com/LuChenLab/r-scATS).
