# Supplementary material for: Accurate profiling of single-cell alternative transcript start sites by correcting RNA degradation
Source: Nat Commun. 2026 Apr 28;17:5798. doi: 10.1038/s41467-026-72298-8 (PMC13332059; doi:10.1038/s41467-026-72298-8)
Supplement: Supplementary file 1 — Supplementary Information [file 41467_2026_72298_MOESM1_ESM.pdf]

Supplementary Note 1

The α metric: RNA degradation rate based on EM model

Background

RNA degradation is an inherent challenge in 5' single-cell RNA sequencing (scRNA-seq)[1] that introduces a pronounced bias in read distribution, with short reads preferentially accumulating toward the 3' end and exhibiting an exponential decay toward the 5' end as previously reported[2,3] (**Fig. 1e**). Current 5' scRNA-seq-based transcription start sites (TSSs) quantification methods do not account for this coverage bias, resulting in inaccurate transcript abundance estimations and biased percent of spliced-in (PSI, ψ) values.

To address this widespread issue of uneven transcript coverage in 5' scRNA-seq data, scATS models coverage bias using an exponential decay function and jointly estimates the degradation-corrected PSI (θ) and the degradation level (α) via an expectation–maximisation (EM) algorithm, thereby providing a unique form of quantification not available in existing approaches, including scTSS[4], SCAFE[5] and CamoTSS[6].

RNA degradation model of scATS

For a given gene g (**Supplementary Figure. 1**), final TSS positions are inferred from TSS clusters using the cumulative distribution of read1 (R1) fragment start sites in paired-end RNA-Seq data, and R1 fragments initially assigned to the corresponding TSSs. Let t index a specific TSS cluster for gene g, with a distribution region of length L_{gt}. Let j denote a base within TSS cluster t used for inferring the true TSS (where j=1 for a single base resolution). Let D_{gtj} represent the distance of base j from the 3' end of the TSS cluster t, and define the normalised distance as

$$d_{gtj} = \frac{D_{gtj}}{L_{gt}}, 0 \leq d_{gtj} \leq 1 \quad (1)$$

let I_{gtj} be an indicator matrix for gene g, where $I_{gtj} = 1$ if TSS cluster t includes base j, and $I_{gtj} = 0$ otherwise.

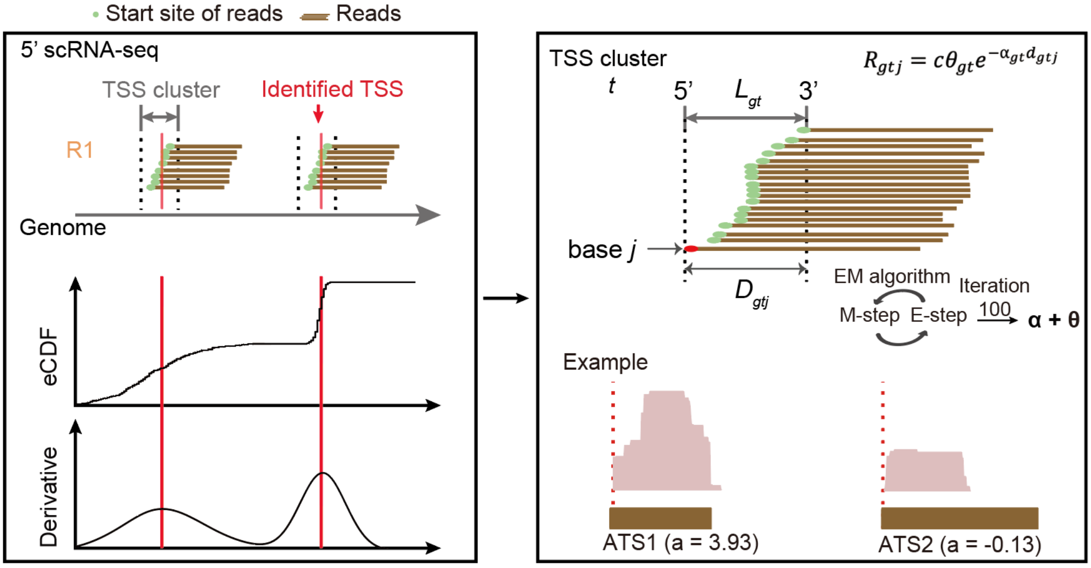

Supplementary Figure. 1 | Overview of the scATS computational framework for joint estimation of the degradation level (α) and degradation-corrected percent spliced-in (PSI, θ).

By mapping R1 reads to the reference genome, we count the number of R1 start sites observed at each base position within a TSS cluster t. Let R_{gtj} denote the observed count of R1 start sites at base j of TSS cluster t for gene g. We found that these counts decrease from the 3' end to the 5' end of the transcript as a result of RNA degradation. Accordingly, we formulate the following mathematical model to quantitatively characterize the impact of RNA degradation on TSS distribution, making it well-suited for 5' scRNA-seq data[7]. We assume that the probability of observing an R1 start site at a given base decreases exponentially with its distance from the TSS due to progressive 5' RNA degradation.

$$R_{gtj} = c\theta_{gt}e^{-\alpha_{gt}d_{gtj}}, \tag{2}$$

where constant c is the normalisation constant. This formulation describes a distance-dependent weighting of expected R1 start-site contributions, rather than assuming an exponential distribution on the observed counts.

Estimation of α and θ at the TSS level

1.1 Notations

To estimate

$$A_g = \{\alpha_{g1}, \alpha_{g2}, \dots, \alpha_{gn}\} \text{ and } \Theta_g = \{\theta_{g1}, \theta_{g2}, \dots, \theta_{gn}\},$$

under the RNA degradation model (see formula 2), the observed R1 start-site counts at individual base positions do not indicate from which TSS cluster or isoform they originate when multiple TSSs are present for a gene. We therefore formulate the inference of TSS-specific α and θ from the observed R1 start-site counts as a missing-data problem, where the latent (missing) variables represent the unknown assignment of observed R1 start-site counts to individual TSS clusters. This formulation leads to an EM framework, in which the E-step estimates the expected contribution of each TSS cluster to the observed R1 start-site counts, and the M-step updates the α and θ accordingly. The observed data are

$$\{R_{g1}, R_{g2}, \dots, R_{gn}\},$$

whereas the missing data are

$$\{R_{gtj}: t = 1, 2, \dots, n;\ j = 1, 2, \dots, x\}$$

Based on the parameters and models defined above, a data table is constructed to facilitate EM-based statistical inference. An illustrative example is provided below:

Supplementary Table 1. Input data structure for the EM algorithm in scATS.

Gene	Position	R1 start site count	Belongs to TSS1	Belongs to TSS2
Gene g	1	44	1	0
Gene g	2	89	1	0
…	…	…	…	…
Gene g	888	100	0	1

Gene g	889	140	0	1

This table details the per-base frequency of R1 start sites in 5’ end mRNA, which serve as the input data for the EM algorithm in scATS. Column descriptions are provided below.

Column	Description
Gene Name	The identifier for the gene being analysed.
Position	The relative coordinate of a single base within the gene.
R1 start site count	The cumulative count of R1 start sites observed at this specific base position. This constitutes the raw observational data for EM.
Belongs to TSS1	A binary indicator (1 if yes, 0 if no) showing whether the R1 start counts at this position is associated with TSS cluster 1.
Belongs to TSS2	A binary indicator (1 if yes, 0 if no) showing whether the R1 start counts at this position is associated with TSS cluster 2.

Because RNA degradation reduces the effective contribution of positions farther from the TSS, we define an effective length for each TSS cluster as the sum of distance-weighted base positions:

$$\omega_{gt} \equiv \sum_j I_{gtj} e^{-\alpha_{gt} d_{gtj}}. \quad (3)$$

This effective length captures the total expected contribution of TSS cluster t under the RNA degradation model and serves as a normalisation term when constructing the likelihood function.

1.2 Statistical estimation of the α and θ

We next utilise an EM algorithm to infer α and θ for all TSSs of a gene g. Specifically, we assume that the observed R1 start-site counts at each base position arise from a mixture of contributions from all TSS clusters of gene g. Under the RNA degradation model, the expected contribution of TSS cluster t to base position j is proportional to $\theta_{gt} e^{-\alpha_{gt} d_{gtj}}$. Because the TSS origin of each

observed count is unobserved, we treat the TSS-specific counts as latent variables and construct the complete-data likelihood accordingly.

For a given TSS, a joint likelihood function is constructed, and the optimal parameters α_{gt} and θ_{gt} are estimated by maximising this function. The likelihood function for gene g is defined as:

$$L_g = \prod_{t,j} \left(\frac{\theta_{gt}\omega_{gt}\frac{l_{gj}e^{-a_{gt}d_{gtj}}}{\omega_{gt}}}{\sum_h \theta_{gh}\omega_{gh}} \right)^{I_{gtj}R_{gtj}}, \quad (4)$$

here, h is an index over all TSS clusters of gene g, and t is a specific TSS cluster among them. The denominator $\sum_h \theta_{gh}\omega_{gh}$ normalizes the contributions across all TSS clusters of gene g, ensuring that the likelihood is properly scaled under the mixture model. Under the constraint $\sum_{i=1}^{n} \theta_{gt} = 1$, the log-likelihood $\log L_g$ is maximised with respect to the parameter set $\left(\theta_{g1}, \theta_{g2}, \ldots, \theta_{gn};\ \alpha_{g1}, \alpha_{g2}, \ldots, \alpha_{gn}\right)$ to obtain the optimal estimates for θ_{gt} and α_{gt}.

The EM algorithm proceeds as follows:

1) E-step

In the E-step, we estimate the expected assignment of the observed R1 start-site counts to each TSS cluster. Given initial values for α_{gt} and θ_{gt}, from iteration x, the expected value of $\log L_g$ is computed:

$$R_{gtj}^{(x)} = E\left(R \middle| \left(R_{g1}, R_{g2}, \ldots, R_{gx}\right), \theta_g^{(x)}, \alpha_g^{(x)}\right) = \frac{R_{gj} I_{gtj} \theta_{gt}^{(x)} e^{-\alpha_g^{(x)} d_{gtj}}}{\sum_h I_{ghj} \theta_{gh}^{(x)} e^{-\alpha_g^{(x)} d_{ghj}}}, \quad (5)$$

where x denotes the iteration number of the EM algorithm, and $R_{gtj}^{(x)}$ is the intermediate expected value calculated in the x-th iteration.

2) M-step

The M-step updates the TSS-specific parameters by maximising the expected complete-data log-likelihood based on the assignments estimated in the E-step. Maximize $\log L_g$, leading to the update of $\hat{\beta}_{gt}^{(x+1)}$:

$$\hat{\beta}_{gt}^{(x+1)} = \frac{\sum_j I_{gtj} R_{gtj}^{(x)}}{R_g}, \tag{6}$$

where x and x+1 represent consecutive iterations of the EM algorithm, and $\hat{\beta}$ indicates an estimated parameter. $\hat{\beta}_{gt}^{(x+1)}$ represents the updated estimated contribution ratio parameter β for the TSS cluster t of gene g in the (x+1)-th iteration.

Here, $\beta_{gt} \equiv \theta_{gt}\omega_{gt}/\sum_h \theta_{gh}\omega_{gh}$, and R_g is the total count of R1 fragments for gene g. The E-step and M-step are iteratively repeated (default 100 times) until the model converges, i.e., the values of α_{gt} and θ_{gt} no longer change. This process yields the optimal α_{gt} and θ_{gt} for different TSSs of the gene g under analysis.

Due to RNA degradation, PSI values calculated by conventional proportion-based methods are systematically underestimated. Our approach, which integrates an RNA degradation model with an EM algorithm, corrects for this bias by inferring the underlying expression level attributable to each TSS. Consequently, degradation-affected TSSs often receive a higher corrected expression value (θ) compared to their raw proportion-based PSI (ψ). For instance, in the *Chchd2* gene, ATS1 shows minimal degradation (α=0.14), while ATS2 exhibits substantial degradation (α=2.76). For the highly degraded ATS2, its raw ψ is only 0.1, whereas our method calculates a corrected θ of 0.27, effectively rescuing its inferred expression level (**Fig. 1f**).

Supplementary Note 2

The β metric: RNA integrity score

To quantitatively assess the integrity of RNA molecules originating from a specific TSS, we define the β metric. This value represents the normalised area under the curve of the empirical cumulative distribution function (ECDF) for R1 initiation sites within a TSS region, serving as a measure of transcript completeness:

$$\beta = \frac{\int Fn(x)dx}{l},$$

where l denotes the length of the genomic interval (in base pairs) spanned by R1 initiation sites for the given TSS. The β metric yields values ranges from 0 to 1 and is interpreted as follows:

We illustrate the biological interpretation of the β metric using two representative cases from **Supplementary Figure. 2**.

1. Low β value (β = 0.45, **Supplementary Figure. 2a**): The ECDF curve increases gradually and lies predominantly below the diagonal line, which represents a random uniform distribution. This means the distribution of R1 start sites is biased towards the 3' end of the locus. Such a pattern is typically caused by 5' degradation of the RNA, where only fragments closer to the 3' end are successfully reverse-transcribed and sequenced. Thus, a β value below 0.5 suggests an unreliable TSS signal due to significant 5' degradation.
2. High β value (β = 0.91, **Supplementary Figure. 2b**): The ECDF curve rises sharply near the 5' end and quickly plateaus, indicating that the vast majority of reads initiate from a precise, narrow genomic region. This pattern is characteristic of high-quality, intact transcripts corresponding to a strong and well-defined TSS. Therefore, a β value approaching 1.0 signifies high TSS signal integrity and TSS specificity.
3. β value near 0.5: A β value close to 0.5 suggests a near-uniform distribution of R1 start sites across the locus, with no clear 5' or 3' bias. This can arise from

either stochastic transcript degradation or the presence of multiple, weak TSSs dispersed across a broad region, lacking a single dominant initiation site.

In summary, the β metric provides an intuitive and quantitative scale (0 to 1) for evaluating the technical quality of a TSS signal, directly reflecting the integrity of the originating RNA molecules.

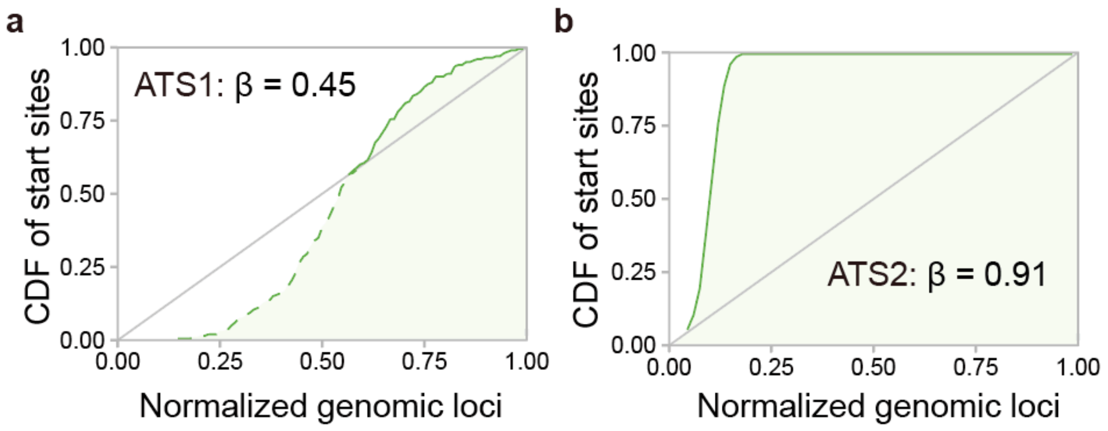

Supplementary Figure. 2 | Relationship between RNA degradation, read distribution, and the β metric.

a-b, Schematic representation of read start distributions and their corresponding ECDFs for transcripts with low (a) and high (b) levels of 5’ RNA degradation, illustrating how the β value quantifies transcript integrity.

Supplementary Note 3

Simulation framework

To systematically evaluate the identifiability and robustness of scATS under realistic 5' scRNA-seq noise, we constructed a hierarchical simulation framework based on the real human genome, transcript sequences, and the experimental workflow of 5' scRNA-seq.

3.1 Construction of the transcript set

We performed simulations using the GRCh38 reference genome with the Ensemble release GTF. To minimize ambiguity and control complexity, we applied stringent filtering criteria:

1) Only protein-coding transcripts from the positive strand exceeding 500 base pairs (bp) in length were retained;
2) Transcripts with TSSs spaced less than 200 bp apart were removed to ensure TSS signal independence.
3) Transcripts exhibiting cross-gene overlaps were excluded to guarantee non-interfering genomic intervals.
4) The final gene set used for downstream simulation consisted exclusively of genes containing a maximum of two TSSs.

3.2 Specification of true transcript usage

For genes with a single transcript, the transcript usage proportion PSI was fixed to one. For genes with two transcripts, a fixed set of true PSI values according to an exponential distribution was assigned and shared across all cells. These PSI values were only used to distribute gene-level expression to transcripts and did not introduce cell-level variability at this stage.

3.3 Simulation of gene-level and transcript-level expression

For each cell, a subset of genes was randomly selected to be expressed, and a total expression count was generated for each expressed gene. Gene-level counts were then allocated to transcripts according to the predefined PSI, resulting in a transcript-by-cell expression matrix. This procedure ensured that transcript counts within a gene summed exactly to the corresponding gene-level expression.

In real 5’ scRNA-seq data, UMI counts typically exhibit overdispersion, which has been reported to follow a Negative Binomial (NB) distribution:

$$N \sim Negative\ Binomial\ (\mu, \phi),$$

where μ denotes the true expected expression level, and ϕ is the overdispersion parameter. When ϕ=0, the distribution reduces to a Poisson distribution with Var(N)=Mean(N). In our simulations, the ideal noise-free expression corresponds to μ, while technical noise is controlled through ϕ.

Using real NSCLC 5’ scRNA-seq data, we further characterised the noise properties of UMI counts at the TSS level. We observed that cell-level counts for individual TSSs deviate substantially from Poisson expectations, with variances consistently exceeding the mean. TSS-level counts are well captured by an NB distribution (**Supplementary Figure. 3a-b**), with overdispersion parameters ϕ clearly greater than zero and varying systematically with the mean TSS count (**Supplementary Figure. 3c**).

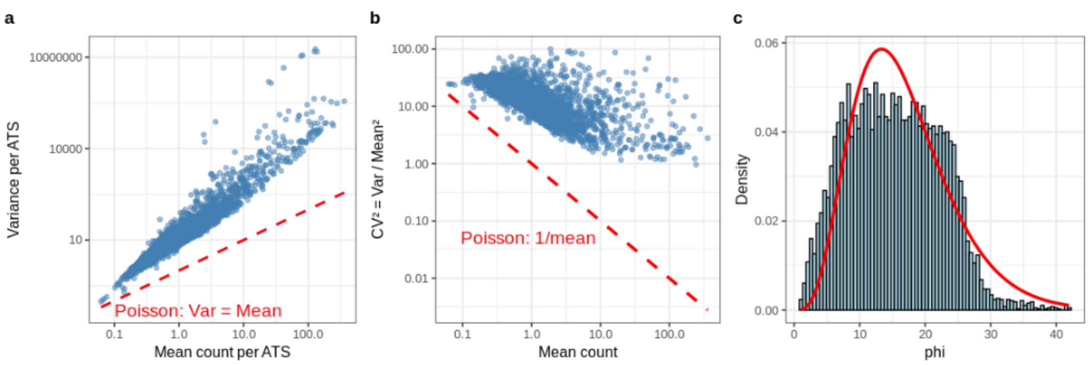

Supplementary Figure. 3 | Technical noise characteristics of TSS-level counts in NSCLC 5’ scRNA-seq.

a, Mean–variance relationship across TSSs. Each point corresponds to one TSS, where the variance of cell-level counts is plotted against the mean. Red dashed line indicates the Poisson expectation (Var = Mean). Most ATS substantially exceed this line, demonstrating pronounced overdispersion.

b, Squared coefficient of variation (CV^2 = Var/Mean^2) versus mean count. Red dashed line indicates the Poisson model with CV^2 = 1/Mean. Real TSS displays much larger CV^2 values, especially at low expression levels, indicating elevated relative technical noise.

c, Distribution of TSS-specific overdispersion parameters ϕ, estimated from empirical variance and mean for each TSS.

3.4 Modeling and implementation of RNA degradation

To simulate RNA degradation[2,3,7], we followed a probabilistic model based on the exponential decay of R1 start sites relative to the TSS. Specifically, the R1 start position is resampled according to an exponential decay model according to formula 2, with the original TSS being preserved with a probability of $e^{-\alpha}$, where α is the degradation rate specific to the gene and TSS. For positions downstream of the TSS, the probability of a given R1 start position d is modeled by:

$$P(d = k \mid d > 0) \propto e^{-\alpha k}, k = 1, \ldots, W,$$

here, W represents the size of the degradation window, which defines the maximum allowed distance from the TSS for resampling. As a result, the distribution of R1 start sites exhibits a peak at the TSS, followed by an exponentially decaying tail, reflecting progressive 5' RNA degradation. The R1 start position was then set to S=T+d, where T denotes the original TSS and d is the resampled offset.

These simulated datasets are designed to capture the impact of RNA degradation on 5' scRNA-seq data, providing a reproducible and biologically consistent benchmark for evaluating the performance of scATS and DeepKINET (v0.2.0) on degradation-affected datasets (**Supplementary Figure. 1g-h**).

Supplementary Note 4

Benchmarking methodology and reproducibility details

To ensure reproducibility, we have provided detailed descriptions and exact command lines for executing each benchmarked method. All tools were run with default parameters, with minimal adjustments applied only in cases when the default settings yielded no or very few ATSs.

4.1 SCAFE

To prepare all required files for SCAFE[5], we followed the official workflow provided in the SCAFE repository (https://github.com/chung-lab/SCAFE/tree/main) and generated each file according to the described steps.

4.1.1 Preparation of the genome files

We generated the required genome files (**Code 1**) and used these files in all subsequent steps of the SCAFE analysis.

Code 1: Preparing genome files.

```
gtf='./cellranger/ref/Homo_sapiens/genes/genes.gtf'
fa='./cellranger/ref/Homo_sapiens/fasta/genome.fa'
mask='./SCAFE/mask.bed'
chrom='./SCAFE/chrom_list.txt'

scafe.tool.cm.prep_genome \
--gtf_path ${gtf} \
--chrom_list_path ${chrom} \
--fasta_path ${fa} \
--mask_bed_path ${mask} \
--outputPrefix hg38_NSCLC \
--outDir ./SCAFE/resources/genome/ \
--overwrite yes
```

4.1.2 Infer TSSs

SCAFE provides two methods for TSS inference, which are scafe.workflow.sc.pool and scafe.workflow.sc.solo, and both methods process the BAM files produced by

Cell Ranger (v4.0.0) without any preprocessing. We ran each method with its default parameters.

4.1.2.1 scafe.workflow.sc.pool

The scafe.workflow.sc.pool is designed for studies that contain multiple samples. For this workflow, each sample is first processed with scafe.tool.sc.bam_to_ctss, and the paths to the resulting files are then organised into a text file that serves as one of the inputs to scafe.workflow.sc.pool.

a. scafe.tool.sc.bam_to_ctss

This step converts each BAM file into a ctss.bed file that carries TSS information (**Code 2**).

Code 2: Running scafe.tool.sc.bam_to_ctss.

```
bam='./possorted_genome_bam.bam'
out='./SCAFE/'

scafe.tool.sc.bam_to_ctss \
--overwrite=yes \
--bamPath=${bam}  \
--genome=hg38_NSCLC \
--outputPrefix=NSCLC \
--outDir=${out} \
>${out}/scafe_bam_to_ctss.log 2>&1
```

b. scafe.workflow.sc.pool

This workflow also receives the matched ATAC data and produces TSS predictions under several thresholds, including all, lenient, default, and robust (**Code 3**). In this study, we used the ‘all’ version for the benchmarking analyses.

Code 3: Running scafe.workflow.sc.pool.

```
out='./SCAFE/'
atac='./ENCODE/human/ATAC/A549/ENCFF399KCR_no_chr.bigwig'

mkdir ${out}/pool/
scafe.workflow.sc.pool \
--lib_list_path=${out}/LUSC_lib.txt \
--genome=hg38_NSCLC \
--run_tag=LUSC \
--run_outDir=${out}/pool/ \
--max_thread=40 \
--training_signal_path=${atac} \
--testing_signal_path=${atac}
```

4.1.2.2 scafe.workflow.sc.solo

This workflow is used when only a single sample is available. It generates the inferred TSSs in one step and does not require any preprocessing of the BAM file. The workflow directly processes the sample and outputs the predicted TSSs under the same threshold settings as the pooled workflow (**Code 4**).

Code 4: Running scafe.workflow.sc.solo.

```
bam='./possorted_genome_bam.bam'
out='./SCAFE/'
atac='./ENCODE/human/ATAC/A549/ENCFF399KCR_no_chr.bigwig'
cellbc='./raw_feature_bc_matrix/barcodes.tsv.gz'

mkdir ${out}/solo/
scafe.workflow.sc.solo \
--overwrite=yes \
--run_bam_path=${bam} \
--run_cellbarcode_path=${cellbc} \
--genome=hg38_NSCLC \
--run_tag=LUSC \
--max_thread=40 \
--training_signal_path=${atac} \
--testing_signal_path=${atac} \
--run_outDir=${out}/solo/
```

4.1.3 TSS quantification

TSS usage values (percent of spliced-in, PSI) were calculated for each gene based on TSS level information derived from the tssCluster.bed.gz file (**Supplementary Table 2**). Gene annotation and genomic coordinates were used to assign TSSs to genes, and the nearest gene for each TSS was determined using the distanceToNearest function from the GenomicRanges package (v1.38.0)[8]. For each

gene, PSI was then computed as the proportion of reads associated with a given TSS relative to the total reads across all TSSs assigned to that gene.

Supplementary Table 2. tssCluster.bed.gz file generated by SCAFE.

Chr	start	End	Cluster id	Count	Strand	Peak start	Peak end
**1**	14731	14830	1_14731_14830_-	34	-	14731	14732
**1**	16719	16905	1_16719_16905_-	12	-	16899	16900
**...**	...	**...**	...	**...**	...	**...**	...
**9**	138222520	138222712	9_138222520_138222712_-	18	-	138222637	138222638
**9**	138280157	138280182	9_138280157_138280182_+	38	+	138280172	138280173

4.2 CamoTSS

4.2.1 Preprocess

To get cleaner bam, we processed the bam files output by Cell Ranger (v4.0.0) based on the following steps (**Code 5**) provided in the CamoTSS[6] GitHub (https://camotss.readthedocs.io/en/latest/preprocess.html)

Code 5: Processing bam file.

```
samtools view  possorted_genome_bam.bam | LC_ALL=C grep "xf:i:25" > body_filtered_sam
samtools view -H possorted_genome_bam.bam > header_filted_sam
cat header_filted_sam body_filtered_sam > possorted_genome_bam_filterd.sam
samtools view -b possorted_genome_bam_filterd.sam > possorted_genome_bam_filterd.bam
samtools index possorted_genome_bam_filterd.bam possorted_genome_bam_filterd.bam.bai
```

4.2.2 Infer TSSs

We perform TSS quantification on the clean BAM files obtained from **Code 1** processing using the TC mode and CTSS mode of CamoTSS. We utilised the 10x

Genomics pre-built mm10 reference (2020-A), which was downloaded from (https://cf.10xgenomics.com/supp/cell-exp/refdata-gex-mm10-2020-A.tar.gz).

To run CamoTSS, we need to prepare the corresponding input files, such as the cell barcode file (**Supplementary Table 3**), bam and genome files, as well as the ref_gene (**Supplementary Table 4**), and ref_TSS files (**Supplementary Table 5**) generated by the built-in functions of CamoTSS.

Supplementary Table 3. Cell barcode file used in CamoTSS.

Cell_id
AAAGATGAGGATTCGG-1
AAAGCAACAAAGCGGT-1
...
TTTACTGTCACAACGT-1
TTTCCTCTCCCAACGG-1

Supplementary Table 4. ref_gene file generated by CamoTSS.

Chromosome	Feature	Start	End	Strand	Gene_id	Gene_name
GL456210.1	gene	123791	124928	+	ENSMUSG00000079192	AC125149.1
GL456210.1	gene	147791	149707	+	ENSMUSG00000094799	AC125149.4
**...**	...	...	...	...	...	...
chrY	gene	90752426	90755467	-	ENSMUSG00000095366	Gm21860
chrY	gene	90838868	90839177	-	ENSMUSG00000096850	Gm21748

Supplementary Table 5. ref_TSS file generated by CamoTSS.

Transcript_id	Gene_id	Gene_name	Chromosome	Strand	TSS
ENSMUST00000111364	ENSMUSG00000079192	AC125149.1	GL456210.1	+	123791
ENSMUST00000115928	ENSMUSG00000079192	AC125149.4	GL456210.1	+	147791
...	...	...	...	...	...
ENSMUST00000177893	ENSMUSG00000095366	Gm21860	chrY	-	90754821
ENSMUST00000179124	ENSMUSG00000096850	Gm21748	chrY	-	90839177

When using default parameters, the 'CTSS/all_ctss.h5ad' file in the output folder contained only 38 TSS sites. Our running code is as follows (**Code 6**).

Code 6: Running CamoTSS with default parameters.

```
cd ./camotss
bamFile=./possorted_genome_bam_filterd.bam
gtfFile=./genes.gtf
fastaFile=./genome.fa
cellbarcodeFile=./all.cell.barcode.tsv

CamoTSS --gtf ${gtfFile} --refFasta ${fastaFile} --bam ${bamFile} \
-c ${cellbarcodeFile} -o run4 --mode TC+CTSS -p 40
```

To increase the diversity and comparability of CamoTSS TSSs, we adjusted the following parameters, ultimately obtaining 1,955 TSSs, which were then used for method comparisons. Our running code is as follows (**Code 7**).

Code 7: Running CamoTSS with custom parameters.

```
cd ./camotss
bamFile=./possorted_genome_bam_filterd.bam
gtfFile=./genes.gtf
fastaFile=./genome.fa
cellbarcodeFile=./all.cell.barcode.tsv

CamoTSS --gtf ${gtfFile} --refFasta ${fastaFile} --bam ${bamFile} \
-c ${cellbarcodeFile} -o run5 --mode TC+CTSS --minCount 10 -p 40 \
--windowSize 5 --minCTSSCount 10 --minFC 1.2
```

In summary, we attribute the limited number of TSS identified by CamoTSS to the following two reasons:

1. Bam file filtering

This step is essential for enriching high-confidence transcriptional evidence by removing reads originating from non-genic regions or ambiguous mappings. The application of this filter reduced the total number of alignments from an initial 131,038,448 to 4,194,732. While this filtering is a critical quality control measure, the resulting loss of over 96% of the initial data is substantial (**Supplementary Figure. 4**). Such a huge reduction, though purifying the signal, is a likely contributor to the low total number of TSSs ultimately identified in the downstream analysis.

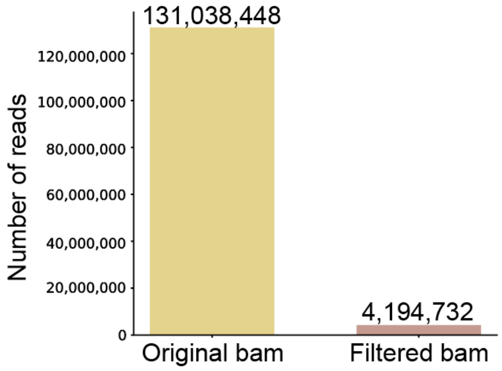

Supplementary Figure. 4 | The read counts before and after filtering.

We also tried using the unfiltered raw BAM, which is the output from Cell Ranger, to run CamoTSS, and then found the following error (**Code 8**).

Code 8: The error message of CamoTSS.

```
hi , this is the start
Traceback (most recent call last):
  File "/mnt/myconda3/envs/ATS/bin/CamoTSS", line 8, in <module>
    sys.exit(main())
  File "/mnt/myconda3/envs/ATS/lib/python3.8/site-packages/CamoTSS/bin/count.py", line 156, in main
    scadata=getTSScount.produce_sclevel()
  File "/mnt/myconda3/envs/ATS/lib/python3.8/site-packages/CamoTSS/utils/get_counts.py", line 464, in produce_sclevel
    extendls,regiondf=self._TSS_annotation()
  File "/mnt/myconda3/envs/ATS/lib/python3.8/site-packages/CamoTSS/utils/get_counts.py", line 423, in _TSS_annotation
    keepdict=self._filter_false_positive()
  File "/mnt/myconda3/envs/ATS/lib/python3.8/site-packages/CamoTSS/utils/get_counts.py", line 293, in _filter_false_positive
    altTSSdict=self._do_hierarchial_cluster()
  File "/mnt/myconda3/envs/ATS/lib/python3.8/site-packages/CamoTSS/utils/get_counts.py", line 254, in _do_hierarchial_cluster
    readinfodict=self._get_gene_reads()
  File "/mnt/myconda3/envs/ATS/lib/python3.8/site-packages/CamoTSS/utils/get_counts.py", line 151, in _get_gene_reads
    geneid=read.get_tag('GX')
  File "pysam/libcalignedsegment.pyx", line 2604, in pysam.libcalignedsegment.AlignedSegment.get_tag
  File "pysam/libcalignedsegment.pyx", line 2643, in pysam.libcalignedsegment.AlignedSegment.get_tag
KeyError: "tag 'GX' not present"
```

2. The minFC argument

A key parameter in the CamoTSS pipeline, minFC, defines the minimum fold change of a signal over background required to call a CTSS. This parameter is critical for discriminating genuine TSS signals from background noise and directly governs the trade-off between sensitivity and precision in TSS identification. While the default value is a stringent 6.0, we employed a highly permissive threshold of 1.2 in our analysis (**Supplementary Figure. 5a**). Despite this significant relaxation, the analysis identified a modest number of TSSs (1,955). Furthermore, other critical filtering parameters, such as minCount (set to 10 vs. the default 50) and minCTSSCount (set to 10 vs. the default 100), were also substantially relaxed to maximize sensitivity. Among 8,419 eligible genes, TSSs were identified in only 0.3% using default parameters. Even with the minFC parameter maximally relaxed, only 7% of genes showed TSS (**Supplementary Figure. 5b**). This outcome suggests two possibilities: either the sequencing depth of our library was insufficient to produce robust signals, or the tool may be less sensitive for detecting TSSs from low-abundance transcripts.

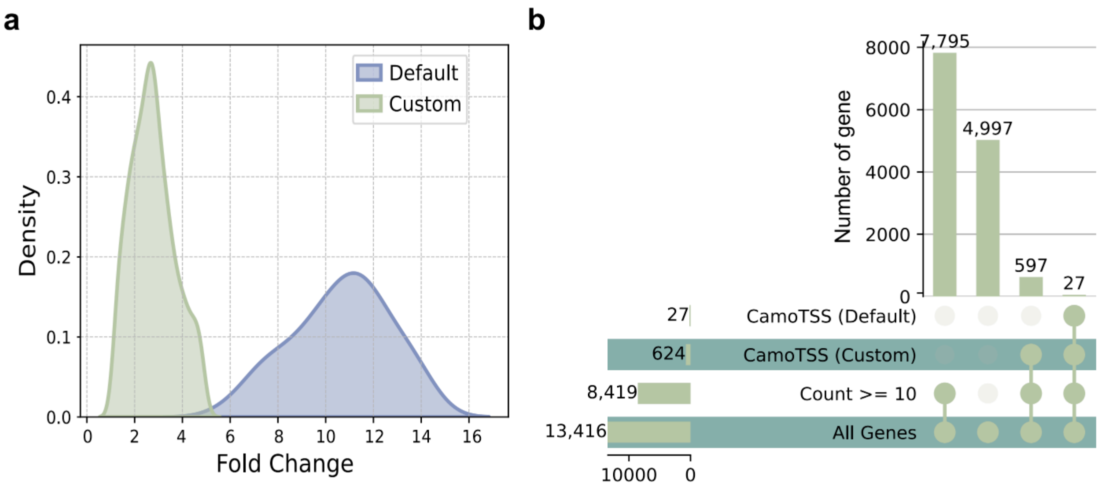

Supplementary Figure. 5 | Effect of CamoTSS parameters on TSS quantification.

a, Fold-change distribution of identified TSSs under different parameters.

b, Comparison of host genes for identified TSSs at different parameters, compared to all and eligible genes.

4.2.3 TSS quantification

TSS level information generated by CamoTSS was obtained from the file 'all_ctss.h5ad' and loaded with Scanpy (v1.9.8) as an AnnData object. In this object, the matrix *X* contains CTSS counts (**Supplementary Table 6**), with rows corresponding to single cells and columns corresponding to individual TSSs, while TSS annotations, including the associated gene, are stored in the var slot (**Supplementary Table 7**). CTSS counts were summed across all cells for each TSS. PSIs of TSS were calculated for each gene as the fraction of CTSS signal contributed by a given TSS relative to the total CTSS signal of all TSSs assigned to that gene.

Supplementary Table 6. CTSS counts stored in X slot from all_ctss.h5ad file.

	**ENSG00000187608*1013413_1013567#1013496@1120$12.464788732394366**	**...**	**ENSG00000198692*20575775_20575879#20575775@619$8.439201451905626**
**AGTCTTTAGCGTTTAC-1**	0	...	48

**ATCATCTGTTCGTCTC** **...**	301	...	690
**ATCATCTGTTCGTCTC** **CGAGAAGCAGTATGCT-1**	109	...	0
**GACGCGTCAAAGCGGT-1**	1	...	10

Supplementary Table 7. TSS level information is stored in the var slot from all_ctss.h5ad file.

clusterID	gene_id	CTSS	counts_dropped_UnencodedG	fold_change	Chr	Feature	Start	End	Strand	Gene_name	len
ENSG00000187608*1013413_1013567#1013496@1120$12.464788732394366	ENSG00000187608	1013496	1120	12.464788732394366	1	gene	1001137	1014540	+	ISG15	13403
...	...	...	...								
ENSG00000198692*20575775_20575879#20575775@619$8.439201451905626	ENSG00000198692	20575775	619	8.439201451905626	Y	gene	20575775	20593154	+	EIF1AY	17379

4.2.4 Explanation of CamoTSS failure on simulated datasets

CamoTSS is a method specifically designed to identify TSSs from 5' scRNA-seq data by integrating hierarchical clustering of read-start positions with a local enrichment–based CTSS (CAGE-based TSS) detection strategy. While this framework is effective for genes with multiple closely spaced candidate TSSs, it is not directly applicable to simulation scenarios characterised by extremely sparse TSS architectures. In our simulation design (see **Supplementary 3**), all genes contain one or two true TSS, the minimum inter-TSS distance exceeds 200 bp.

Under this configuration, CamoTSS fails to produce valid output (**Code 9**), whereas other TSS-detection methods (scATS, SCAFE, TSSr and scTSS) successfully recover the simulated TSSs. Below, we provide a code-level explanation for this behavior.

Code 9: Execution of CamoTSS on simulated datasets and associated runtime error.

```
CamoTSS --gtf ${gtfFile} --refFasta ${fastaFile} \
--bam ./possorted_genome_bam_filterd.bam -c ${cellbarcodeFile} \
-o ./ --mode TC+CTSS -p 20 --minFC 0.5 --minCTSSCount 10 \
--windowSize 3 --InnerDistance 10 --clusterDistance 10

do clustering Time elapsed 2954 seconds.
one_gene_with_two_TSS_fourfeature : 3215
do annotation Time elapsed 3061 seconds.
produce h5ad Time elapsed 3081 seconds.
window sliding Time elapsed 3 seconds.
Traceback (most recent call last):
  File "./miniconda3/envs/ATS/bin/CamoTSS", line 8, in <module>
    sys.exit(main())
  File "./site-packages/CamoTSS/bin/count.py", line 157, in main
    twoctssadata=getTSScount.produce_CTSS_adata()
  File "./site-packages/CamoTSS/utils/get_counts.py", line 642, in produce_CTSS_adata
    ctssadata=ad.AnnData(ctssfinaldf)
  File "./site-packages/anndata/_core/anndata.py", line 291, in __init__
    self._init_as_actual(
  File "./site-packages/anndata/_core/anndata.py", line 432, in _init_as_actual
    X = ensure_df_homogeneous(X, "X")
  File "./site-packages/anndata/utils.py", line 248, in ensure_df_homogeneous
    arr = df.sparse.to_coo().tocsr()
  File "./site-packages/pandas/core/arrays/sparse/accessor.py", line 337, in to_coo
    dtype = find_common_type(self._parent.dtypes.to_list())
  File "./site-packages/pandas/core/dtypes/cast.py", line 1447, in find_common_type
    raise ValueError("no types given")
ValueError: no types given
```

First, CTSS detection in CamoTSS is implemented in the window_sliding function from get_ctss.py, which requires multiple candidate TSS positions within a local window to compute enrichment statistics. When only one or two isolated TSS positions are present, the sliding-window procedure yields no valid CTSS candidates.

Second, empty CTSS candidate sets are propagated to downstream aggregation steps without explicit handling of this edge case, resulting in empty cell-by-CTSS matrices and subsequent runtime errors during data structure construction.

Third, this limitation reflects the intended scope of CamoTSS rather than a deficiency of the method. CamoTSS is designed to resolve complex ATS landscapes where local competition between nearby start sites necessitates enrichment-based discrimination. In contrast, our simulation models a simplified transcriptional architecture in which TSS positions are unambiguous and widely separated, a scenario for which sliding-window–based CTSS detection is neither required nor well-defined. We note that adjusting clustering distance or window size parameters did not resolve this issue, as the absence of multiple local TSS candidates per gene fundamentally violates the assumptions of the CTSS detection module.

For these reasons mentioned above, CamoTSS was not included in the quantitative evaluation on simulated datasets, while remaining applicable to real datasets with more complex TSS structures.

4.3 TSSr

TSSr is an R/Bioconductor package for TSS analysis, compatible with diverse TSS-enriched sequencing protocols[9] (e.g., CAGE, GRO-cap). We rigorously followed its official documentation (https://github.com/Linlab-slu/TSSr) and executed the following workflow. We ran each function with its default parameters.

4.3.1 Input files

We performed TSS quantification on the BAM file generated by Cell Ranger (v4.0.0) without any preprocessing, using the peakclu clustering mode and the interquartile boundary definition of TSSr. The UCSC reference genome

(BSgenome.Hsapiens.UCSC.hg38 or BSgenome.Mmusculus.UCSC.mm10) was used, and these genomes were pre-installed via Bioconductor (see **Code 10**).

Code 10: Preparing input files and TSSr object.

```
library(BSgenome.Hsapiens.UCSC.hg38)
library(TSSr)
bamFile <- "./possorted_genome_bam.bam"
gtfFile <- "./genes/genes.gtf"
TSSr <- new("TSSr",
genomeName = "BSgenome.Hsapiens.UCSC.hg38",
inputFiles = bamFile,
inputFilesType = "bamPairedEnd",
sampleLabels = "single",
sampleLabelsMerged = "single",
refSource = gtfFile,
organismName = "Homo sapiens")
```

4.3.2 Infer TSS

The following steps perform raw TSS calling, signal normalisation, and quality filtering using the default parameters (**Code 11**).

Code 11: Inferring TSSs.

```
getTSS(TSSr)
normalizeTSS(TSSr)
filterTSS(TSSr, method = "TPM")
```

4.3.3 TSS clustering and core promoter inference

The "peakclu" algorithm clusters TSSs via a 100 bp sliding window, identifies local peaks, merges adjacent TSSs (default: ≤25 bp spacing), and calculates interquartile width (10%-90%) for core promoters (**Code 12**). Results are stored in tagClusters and consensusClusters, exportable as bedGraph/BigWig files.

Code 12: Running clusterTSS function.

```
clusterTSS(TSSr,
method = "peakclu",
peakDistance=100,
extensionDistance=30,
localThreshold = 0.02,
clusterThreshold = 1,
useMultiCore=FALSE,
numCores=NULL)
```

4.3.4 TSS quantification

PSI values were calculated for each gene based on TSS level information derived from the tagClusters slot of the TSSr object (**Supplementary Table 8**). Gene annotation and genomic coordinates were used to assign TSSs to genes, and the nearest gene for each TSS was determined using the distanceToNearest function from the GenomicRanges package (v1.38.0)[8]. For each gene, PSI was then computed as the proportion of reads associated with a given TSS relative to the total reads across all TSSs assigned to that gene.

Supplementary Table 8. Summary of clustered TSSs from the TSSr object.

cluster	chr	start	end	Strand	dominant_tss	tags	tags.dominant_tss	q_0.1	q_0.9	interquantile_width
1	chr1	4807877	4807950	+	4807884	22.445347	3.519839	4807879	4807914	36
2	chr1	4857748	4857883	+	4857791	105.646181	14.436441	4857785	4857829	45
...	...	...	...	...	...	...	...	...	...	...
9	chr1	6214633	6214721	+	6214664	13.110123	3.009717	6214637	6214705	69
10	chr1	6248572	6248605	+	6248572	1.020243	0.459109	6248572	6248605	34

4.4 scTSS

The scTSS framework is designed for differential TSS analysis rather than for *de novo* TSS quantification[10]. In its original study, scTSS benchmarked multiple TSS detection and quantification methods and identified the best-performing tools for different library types, recommending SCAFE for paired-end data and TSSr for R2-only data. The scTSS software is available at https://github.com/ShweiSTAT/scTSS.

Following this design principle, we used the intersection of TSS identification and quantification results from SCAFE and TSSr as the final result for scTSS. PSI values from SCAFE and TSSr were averaged to estimate TSS usage. This conservative strategy reduces method-specific bias and provides a robust and consistent basis for comparative analysis.

4.5 TSS-Captur

TSS-Captur is a tool for analysing prokaryotic TSSs that is critically dependent on TSSpredator for identifying primary TSSs[11]. Designed exclusively for prokaryotic transcriptomes, TSSpredator relies on bacterial transcriptional features such as:

- operon-centric genome organisation (dense gene clusters without introns);
- prokaryotic RNA-seq data inputs (e.g., dRNA-seq/Capable-seq with 5' triphosphate enrichment);
- bacterial promoter or terminator logic (e.g., -10/-35 bp promoter motifs and Rho-dependent termination).

Consequently, TSS-Captur has shown good performance for TSS identification in prokaryotic systems, where its underlying assumptions and models are well aligned with genomic organisation and transcriptional features. However, due to its design principles inherited from TSSpredator, the workflow is not well-suited for eukaryotic TSS detection. In particular, it does not consider eukaryote-specific biological features such as RNA 5' end modifications, including cap structures, alternative splicing, or promoter complexity regulated by chromatin. In addition,

its classification models are optimised for prokaryotic ncRNAs and do not readily transfer to eukaryotic contexts. Because the overall toolchain follows prokaryotic genomic logic and the benchmarking framework in this study focuses on eukaryotic single-cell TSS methods, TSS-Captur was not included in the comparative analysis to ensure methodological consistency.

Supplementary Note 5

Negative dataset using semi-supervised PU learning

The analysis began by loading a pre-processed feature matrix containing 33 features of TSSs. This dataset comprised two main categories: a set of already verified positive TSSs (referred to as ground truth) and a larger collection of unlabeled TSSs. The primary challenge addressed by our pipeline was to identify a reliable negative set from this unlabeled pool, given the inherent difficulty and cost of experimentally validating non-relevant TSSs.

To overcome the absence of explicitly labeled negative samples, we employed a PU learning strategy[12], a semi-supervised machine-learning technique, to construct a high-confidence negative training set. This approach leverages a small subset of known positive samples (called "spy" samples) embedded within the unlabeled data to train an initial classifier. Specifically, 10% of the ground-truth positive TSSs were designated as spy samples and combined with the rest of the unlabeled data. A naïve Bayes classifier was then trained on a synthetically balanced dataset, created by oversampling the known positive samples, to learn the probabilistic distinction between positive and unlabeled examples. The trained classifier was subsequently used to predict the likelihood of all unlabeled samples being positive. A critical step involved setting a probability threshold based on the lowest predicted positive probability among the "spy" samples (specifically, the 1st percentile of spy sample predictions). Unlabeled TSSs whose predicted positive probability fell below this conservative threshold were designated as the reliable negative set. This thresholding strategy ensures that only samples highly dissimilar to known positives (even those initially disguised as unlabeled spies) are considered true negatives, thereby generating a robust negative dataset (RN) for subsequent supervised learning. The size of this reliable

negative set was set to match the initial positive set (PN), creating a balanced and high-confidence training dataset.

Random Forest (RF) classification and performance evaluation

With the PN and RN sets established, we proceeded with building a predictive model for relevant TSSs using an RF classifier. This supervised learning algorithm is well-suited for high-dimensional feature spaces and provides robust classification performance. The generalisation capability of the model was rigorously assessed through 10-fold cross-validation (**Supplementary Figure. 6**). In each fold, the combined positive and negative dataset was partitioned into training and test sets. An RF model was trained on the training data, and its performance was then evaluated on the unseen test data. The Receiver Operating Characteristic (ROC) curve and Area Under the Curve (AUC) were computed for each fold, providing a comprehensive measure of the ability of the classifier to distinguish between relevant (positive) and non-relevant (negative) TSSs. The average AUC across all 10 folds served as the final robust performance metric, and a composite ROC plot was generated for visualisation. All trained RF models and performance metrics were saved for reproducibility and future analysis.

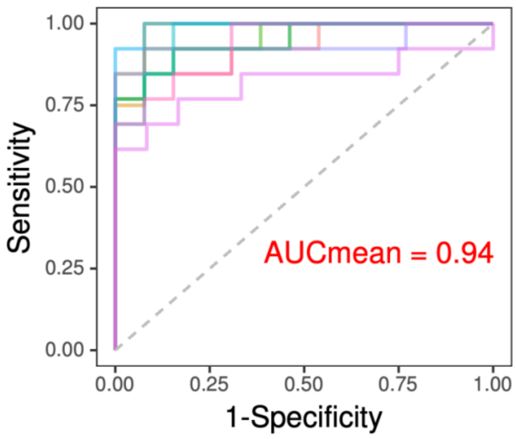

Supplementary Figure. 6 | The ROC curves are used to assess the performance of the training datasets.

Supplementary Note 6

Screening for the best hyperparameters for the RF model

RF hyperparameters were optimised by exhaustive grid search (scikit-learn 1.3.2, Python 3.8.20). For each candidate setting:

Supplementary Table 9. The hyperparameters of the RF model.

Parameters	Settings
Tree number (n_estimators)	{100, 300, 500, 800, 1000}
Features number for a tree (max_features)	{6, 12, 18, 32}
Bootstrap fraction (max_samples)	{0.60, 0.80, 1.00}
maximum depth (max_depth)	{None, 5, 10, 20}

Model selection was based on mean area under the receiver-operating-characteristic curve (AUROC); Grid evaluation used a fixed random seed (42) and was executed on a single 40-core Intel (R) Xeon (R) node; the full sweep ($5 \times 4 \times 3 \times 5 = 300$ configurations) completed within 50 min wall time. The best-performing hyperparameter triple (1,000 trees, unlimited depth, 0.80 sampling fraction) was refit on the entire training set and subsequently assessed on the held-out test data.

Supplementary Note 7

Extension of the LRS framework to glioblastoma dataset

To further demonstrate the extensibility of the LRS (lung cancer relevance score) framework, we applied the workflow to glioblastoma (GBM), which is a common malignant brain tumor. We applied scATS to a publicly available GBM 5' scRNA-seq dataset[13] generated from tumor specimens of 5 patients, and selected the patient with the best sequencing quality for TSS analysis. scATS identified 53,037 TSSs in 16,125 genes across 15,088 cells.

To further demonstrate the extensibility of the LRS framework, we applied the workflow to the GBM 5' scRNA-seq dataset. We first collected 81 TSSs based on a previously published study[14], enabling these TSSs to serve as the positive set. The training set was constructed using the Positive-Unlabeled (PU) learning spy algorithm following the same strategy as in the LRS workflow. Next, we trained an LRS model that predicts TSSs that are closer to the positive set and farther from the negative set. To achieve this, we extracted 28 features spanning four categories from the identified TSSs: (1) regulatory elements (19 features): histone modification signals at the TSS and within ±2,500 bp, transcription factor motifs within ±1,000 bp, CpG-related metrics, and conservation scores; (2) TSS quantification (3 features): TSS expression metrics before (ψ) and after degradation-correction (θ); (3) RNA degradation (3 features): TSS degradation metrics (α and β); (4) transcript features (3 features): properties of the TSS transcripts, including distances to reference TSSs, coding status of TSS transcript, and strand orientations.

We then tested four machine-learning models, including random forest (RF), logistic regression (LR), multilayer perceptron (MLP) and support vector machine (SVM), on the training set using 10-fold cross-validation (**Supplementary Figure.**

7a-d). As the RF model showed the best performance (AUROC = 0.99, mean area under the precision–recall curve [AUPRC] = 0.99) (**Supplementary Figure. 7a**), we selected RF as the final LRS model for subsequent analyses. The importance of all features was evaluated based on their Gini scores (**Supplementary Figure. 7e**).

Next, we applied the LRS model to 53,037 TSSs that were transcribed in all cells and assigned the prediction scores ranging from 0 to 1, where a score closer to 1 indicates a higher likelihood of relevance with GBM. After fitting the LRS distribution of all predicted TSSs with a random-component Gaussian Mixture Model (GMM), we categorised the TSSs into three classes: 'GBM-relevant' (LRS > 0.83), 'non-relevant' (LRS < 0.07) and 'uncertain' (LRS between 0.07 and 0.83). A total of 3,025 TSSs were classified as GBM-relevant, and their LRS values were comparable to those of the known positive TSSs (**Supplementary Figure. 7f).** Disease ontology (DO) analysis revealed enrichment in the "axonal neuropathy", which has been reported as a targetable driver of glioblastoma progression through neuron–glioma interactions[15]. For example, tumor necrosis factor receptor superfamily member 1A (*TNFRSF1A*), enriched in this DO term, has been associated with patient prognosis in GBM[16]. GO analysis highlighted "cerebral cortex development", a pathway linked to glioma aggressiveness via remodeling functional neural circuits[17]. Within this pathway, *TACC3* is frequently amplified and overexpressed in GBM and has been reported to promote tumor progression[18]. Together, these results demonstrate the ability of CRS to identify biologically relevant TSSs in brain cancer.

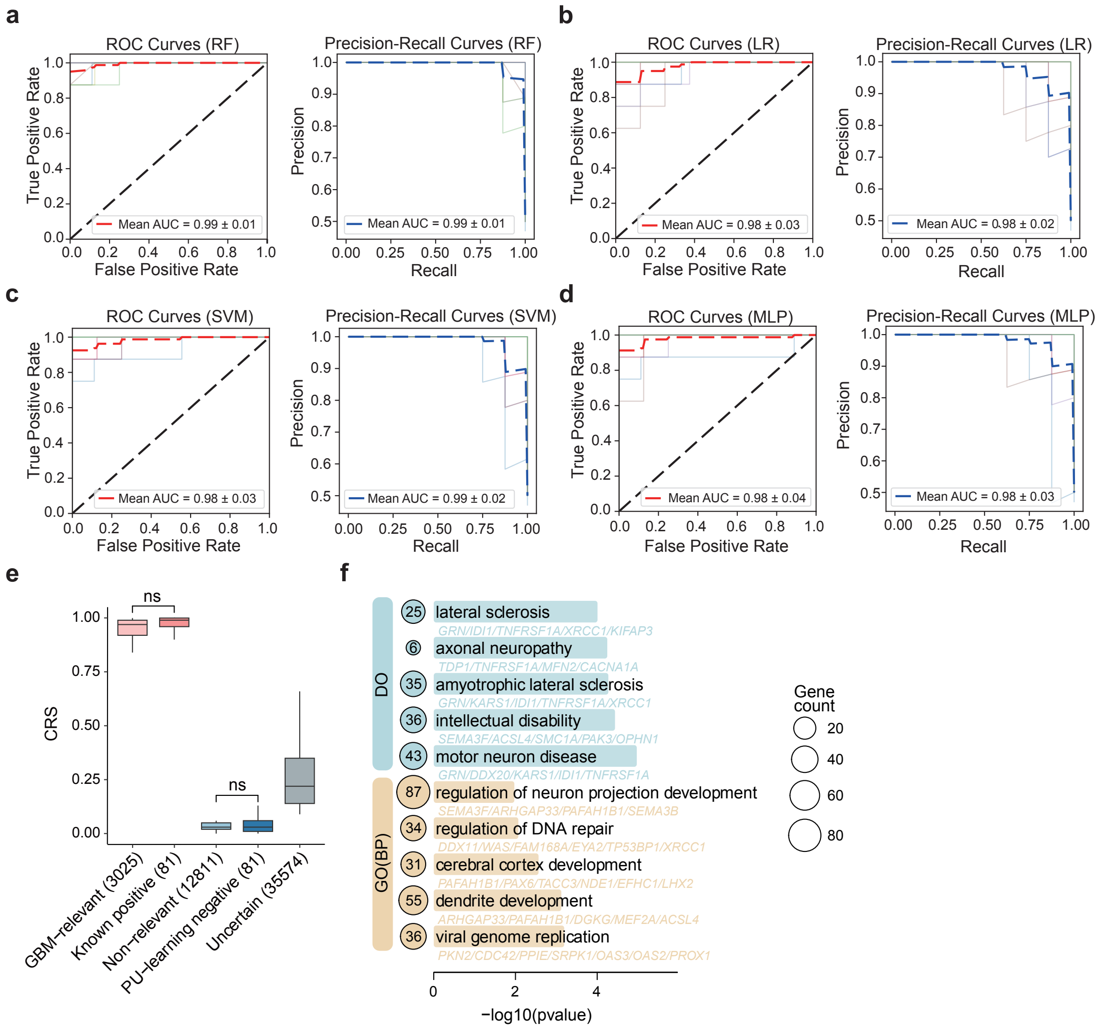

Supplementary Figure. 7 | Single-cell transcriptomic analyses of human GBM and performance of LRS.

a-d. Performance of four models: RF (a), LR (b), SVM (c) and MLP (d) assessed by AUROC (left panels), and AUPRC (right panels). The red and blue dashed lines represent the overall AUROC and AUPRC from 10-fold cross-validation, respectively. The mean AUCs are shown.

e, Box plots showing the distributions of LRS probabilities for GBM-relevant, known positive, non-relevant, PU-learning negative and uncertain TSSs. Number of TSSs in each category shown in parentheses; ns: not significant; a two-sided t-test with BH correction for multiple comparisons.

f, Disease ontology (top panel) and GO enrichment (bottom panel) analyses of the host genes with GBM-relevant TSSs. Numbers to the left of each term indicate the

counts of associated genes, and the contributing host genes are partially listed below. The bars indicate the -log10 (*p* value).

Supplementary Note 8

The parameter settings of scATS and sequencing depth effects

To improve the practical usability of scATS, we provide additional analyses and guidance on TSS detection stringency and sequencing depth requirements. These analyses are intended to assist users in parameter selection and experimental planning across datasets with different coverage levels and cellular compositions.

To address confidence and thresholding in TSS definition, we first examined how gene-level read depth affects the accuracy of TSS inference using simulated data. When the number of reads per gene was limited to 200, the correlation between the estimated alpha values and the ground truth was 0.61. Increasing gene-level read depth substantially improved accuracy, with correlations reaching 0.75 at 300 reads and 0.89 at 500 reads per gene (**Supplementary Figure. 8a**). Because gene-level read depth directly corresponds to the 'MinGeneReads' parameter in scATS, this parameter can be used to control detection stringency. To facilitate parameter selection, we provide a built-in function loadbamgene, which reports the effective number of reads supporting each gene for TSS quantification. Users can examine the read distribution of their own data and choose an appropriate 'MinGeneReads' threshold to retain more reliable TSSs.

In addition to 'MinGeneReads', scATS provides several adjustable parameters that allow users to further tune TSS detection stringency. We evaluated these parameters using the mHSPC 5' scRNA-seq dataset. Increasing the 'MinGeneReads' parameter reduced the number of detected TSSs and genes while increasing the proportion of genes with ATSs (**Supplementary Figure. 8b**). The 'window' parameter, which controls the Gaussian smoothing window size, strongly influenced TSS resolution, with larger window sizes producing smoother signal profiles but substantially reducing the number of detected TSSs, particularly ATSs

(**Supplementary Figure. 8c**). When the window size exceeded 20 bp, no ATSs were detected. The 'greedy' parameter determines whether the most distal annotated TSS is detected even if it does not meet the specified criteria, which increases the number of retained TSSs and genes and provides additional flexibility for exploratory analyses (**Supplementary Figure. 8d**).

To evaluate the effect of sequencing depth on scATS performance, we next performed downsampling of a COVID-19 5' scRNA-seq dataset, which has the highest sequencing depth among the 5' scRNA-seq datasets used in this study. We observed that the number of detected TSSs and genes with ATS increased with sequencing depth and reached saturation at approximately 25,000 reads per cell (**Supplementary Figure. 8e**). This indicates that deep sequencing is required to recover most ATS loci within single cells. We further assessed how sequencing depth influences cell type identification based on TSS usage. Cells were clustered using TSS count matrices generated by scATS and compared with gene-based cell-type annotations using Normalised Mutual Information (NMI)[19] and Adjusted Rand Index (ARI)[20]. At 200 reads per cell, TSS-based clustering showed poor agreement with gene-based annotations. Increasing the depth to 2,000 reads per cell substantially improved clustering performance (**Supplementary Figure. 8f**), while higher depths resulted in only marginal additional gains. These results suggest that sequencing depths around 2,000 reads per cell are sufficient for basic TSS-based cell clustering, whereas comprehensive dissection of ATS landscapes requires more than 25,000 reads per cell.

Together, these analyses demonstrate that both parameter selection and sequencing depth play roles in TSS detection and interpretation using scATS. To further support user-specific applications, we provide a detailed explanation of all parameters in the TSSCDF function and their recommended usage in **Supplementary Table 10**.

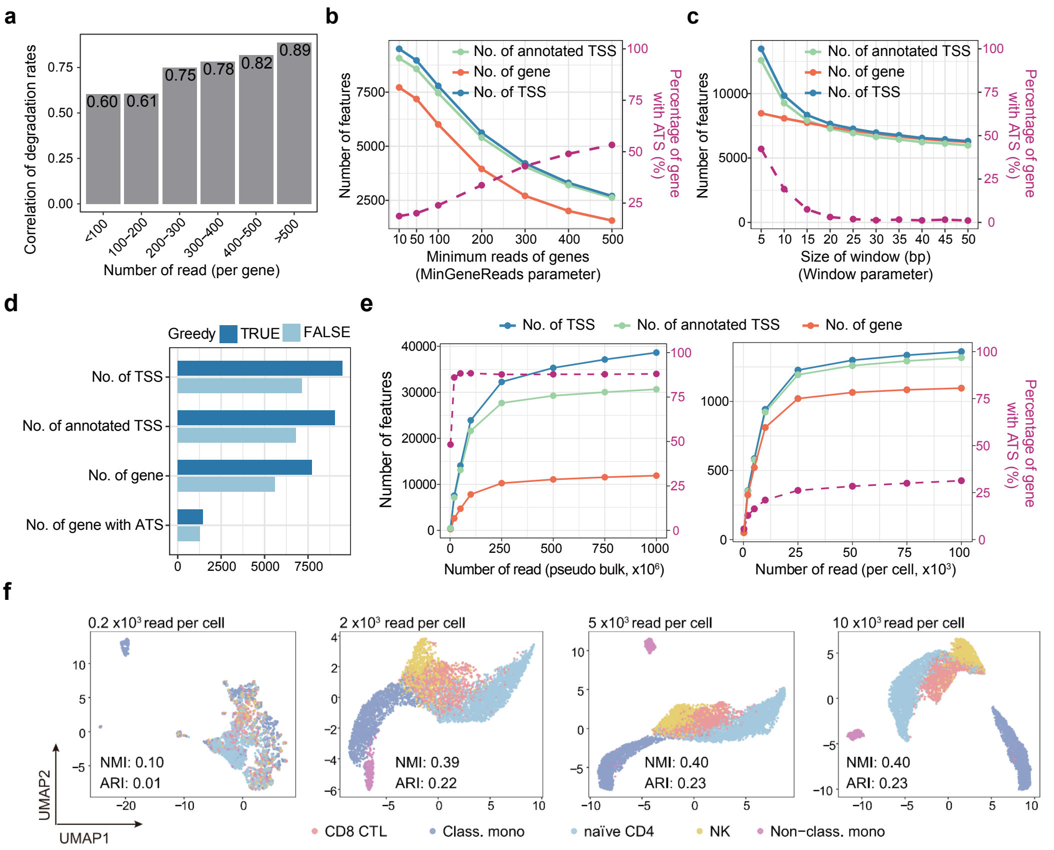

Supplementary Figure. 8 | parameter settings and sequencing depth on TSS detection.

a, Bar plots showing the correlation between estimated alpha values and ground truth across different gene-level read depth groups in the simulated 5’ scRNA-seq dataset. Pearson correlation coefficients are shown in each bar.

b-c, Pseudo-bulk analyses of TSS detection across ‘MinGeneReads’ (b) and ‘window’ (c) parameters in the mHSPCs 5’ scRNA-seq datasets. Line charts show the numbers of detected TSSs, annotated TSSs and host genes (left Y-axis), and red line charts show the percentage of genes with ATSs (right Y-axis). Sequencing depth is indicated on the X-axis. Predicted TSSs located within ±50 bp of CAGE-seq peaks were considered annotated, while others were classified as novel.

d, Bar plots showing the numbers of TSSs and genes with the ‘greedy’ parameter set to TRUE or FALSE.

e, Pseudo-bulk (left panel) and single-cell level (right panel) analyses of TSS detection across downsampled sequencing depths in the COVID-19 5’ scRNA-seq

dataset. Line charts show the numbers of detected TSSs, annotated TSSs and host genes (left Y-axis), and red line charts show the percentage of genes with ATSs (right Y-axis). Sequencing depth is indicated on the X-axis. Predicted TSSs located within ±50 bp of CAGE-seq peaks were considered annotated, while others were classified as novel.

f, UMAP plots of cell clustering based on TSS count matrices at different sequencing depths in the COVID-19 5' scRNA-seq dataset. Clustering performance was quantified using NMI and ARI scores. Cell-types are indicated by different colors.

Supplementary Table 10. Parameters used in the TSSCDF function of scATS.

Arguments	Description
object	A Seurat object.
bam	Path of bam file(s).
genes	Genes used for ATS inference and quantification. By default, all genes in the Seurat object are included.
gtfFile	Path of GTF file or Granges file of GTF file.
txdb	A TxDb object.
UTROnly	Only infer ATS within annotated 5' UTR regions or first exons of transcripts without 5' UTR (default=True).
scDR	Whether to estimate the single-cell level degradation rate of TSS. Setting TRUE triggers an additional inference step and vastly increases runtime (default=False).
MinGeneReads	The minimum reads of a gene for TSS inference (default=500).
MinscTSSReads	Minimum reads of single cell TSS reads for single cell degradation rate estimation when scDR = TRUE (default=5).
min.TSS.percent	Defines the minimum proportion of total supporting reads (i.e., the change in cumulative density, F1 – F0) that a candidate TSS region must contribute. This parameter filters out weak or noise-driven TSS peaks (default=5%).
min.local.percent	Based on the maximum local derivative change, this parameter evaluates whether a candidate TSS region exhibits sufficiently sharp local enrichment. It is used to remove locally insignificant or low-confidence peaks (default=1%).
mapqFilter, isSecondaryAlignment,	parameters for ScanBamParam to read the BAM file(s).

isSupplementaryAlignment, isDuplicate	
p.cutoff	A significance threshold used to determine whether the smoothed derivative exceeds the background derivative distribution, where the cutoff is computed from a fitted normal model. Smaller values of p.cutoff impose stricter criteria and retain only high-confidence TSS candidates (default=0.01).
window	Controls the Gaussian smoothing window and is also used to define local flanking regions and extend candidate TSS boundaries. Larger windows produce smoother trends with reduced resolution, while smaller windows increase sensitivity at the cost of higher noise (default=10).
greedy	greedy for the first annotated TSS? If this parameter is set to TRUE, the most distal annotated TSS will be printed even if it does not satisfy the specified condition (default=True).
cores	The number of cores for parallel working (default=1).
verbose	A logical control if a text progress bar is displayed (default=False).
project.name	The name of the project (default=NULL).

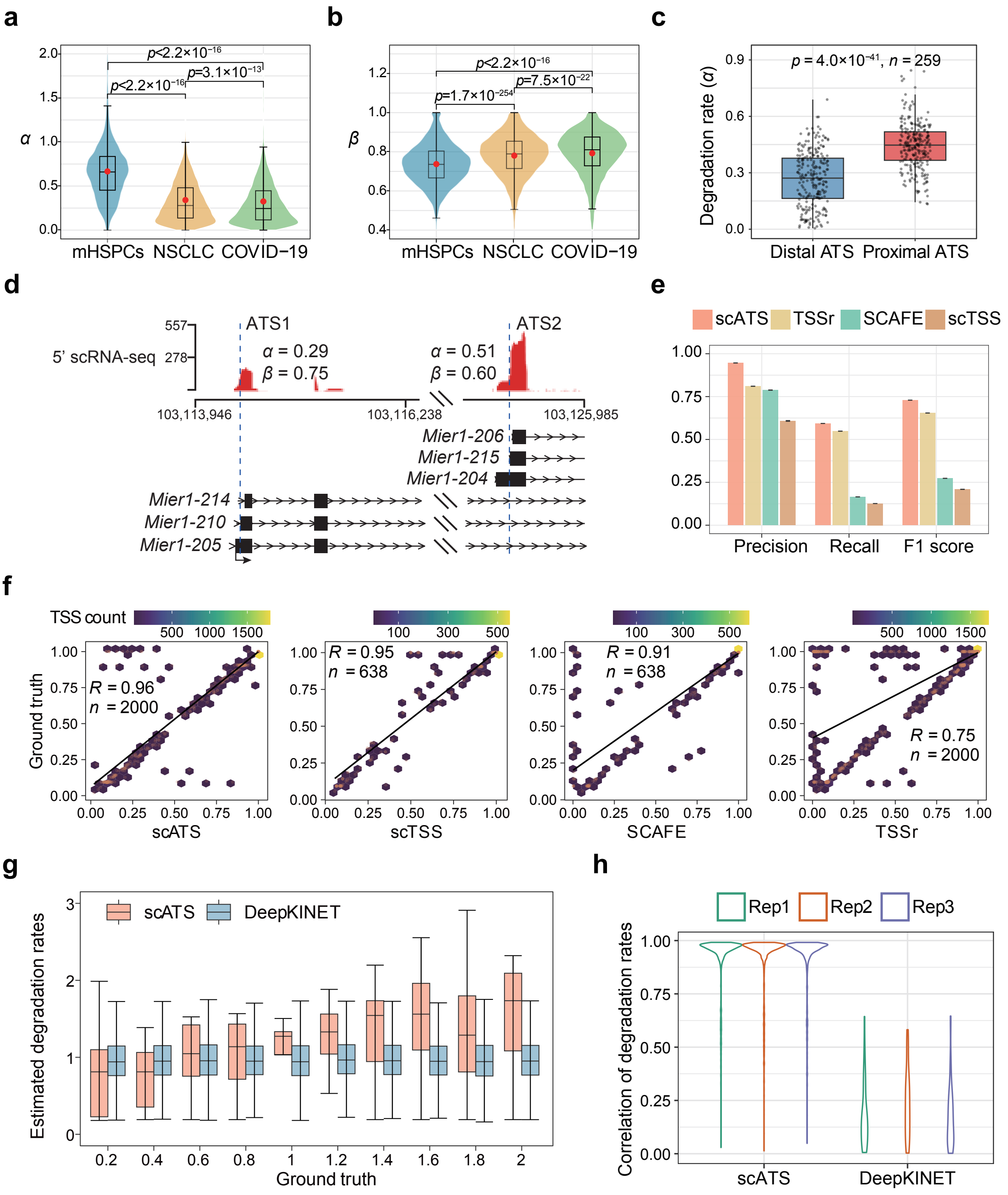

a
p<2.2×10−16
p=3.1×10−13
p<2.2×10−16
α
mHSPCs NSCLC COVID−19
b
p<2.2×10−16
p=7.5×10−22
p=1.7×10−254
β
mHSPCs NSCLC COVID−19
c
p = 4.0×10−41, n = 259
Degradation rate (α)
Distal ATS Proximal ATS
d
5' scRNA-seq
ATS1
ATS2
α = 0.29
β = 0.75
α = 0.51
β = 0.60
103,113,946
103,116,238
103,125,985
Mier1-206
Mier1-215
Mier1-204
Mier1-214
Mier1-210
Mier1-205
e
scATS TSSr SCAFE scTSS
Precision Recall F1 score
f
TSS count
Ground truth
R = 0.96
n = 2000
scATS
R = 0.95
n = 638
scTSS
R = 0.91
n = 638
SCAFE
R = 0.75
n = 2000
TSSr
g
scATS DeepKINET
Estimated degradation rates
Ground truth
h
Rep1 Rep2 Rep3
Correlation of degradation rates
scATS DeepKINET

Supplementary Fig. 1 | Performance of scATS on real and simulated datasets (related to Fig. 1).

a-**b**, Violin plots showing RNA degradation metrics of α (a) and β (b) at the sample level across three 5' scRNA-seq datasets shown in **Fig. 1b**. The red dots represent the mean, the central line represents the median, the box indicates the interquartile range (IQR), and the whiskers represent values within 1.5 times the IQR. The two-sided Wilcoxon test with BH correction for multiple comparisons was used.

c, Boxplots of RNA degradation rates at the pseudo-bulk level, showing higher degradation at proximal ATSs. Two-sided Wilcoxon test was used.

d, Sashimi plots showing the transcription level of *Mier1* in the mHSPC 5' scRNA-seq dataset. The proximal ATS (ATS2) exhibits higher RNA degradation, resulting in a high α value of 0.51 and a relatively low β value (0.60). In contrast, the distal ATS (ATS1) shows mild degradation. The blue dashed lines indicate the loci of predicted TSSs inferred by scATS. GENCODE v25 annotations show below the sashimi plots.

e, Precision, recall and F1 score for TSS identification across four methods on the simulated 5' scRNA-seq datasets.

f, Scatter plots showing the correlation between estimated and true TSS usage for each method on the same simulated TSSs identified in (e). Each point represents one TSS. The black solid line indicates the linear regression fit. Color intensity represents the TSS counts. Pearson correlation coefficients (R) and the number of genes (n) are shown in each panel.

g, Box plots of estimated RNA degradation rates under different ground-truth degradation levels in the simulated dataset at the single-cell level.

h, Violin plots showing the distribution of correlations between estimated and ground-truth degradation rates across three independent simulation replicates, computed from the simulations shown in (g).

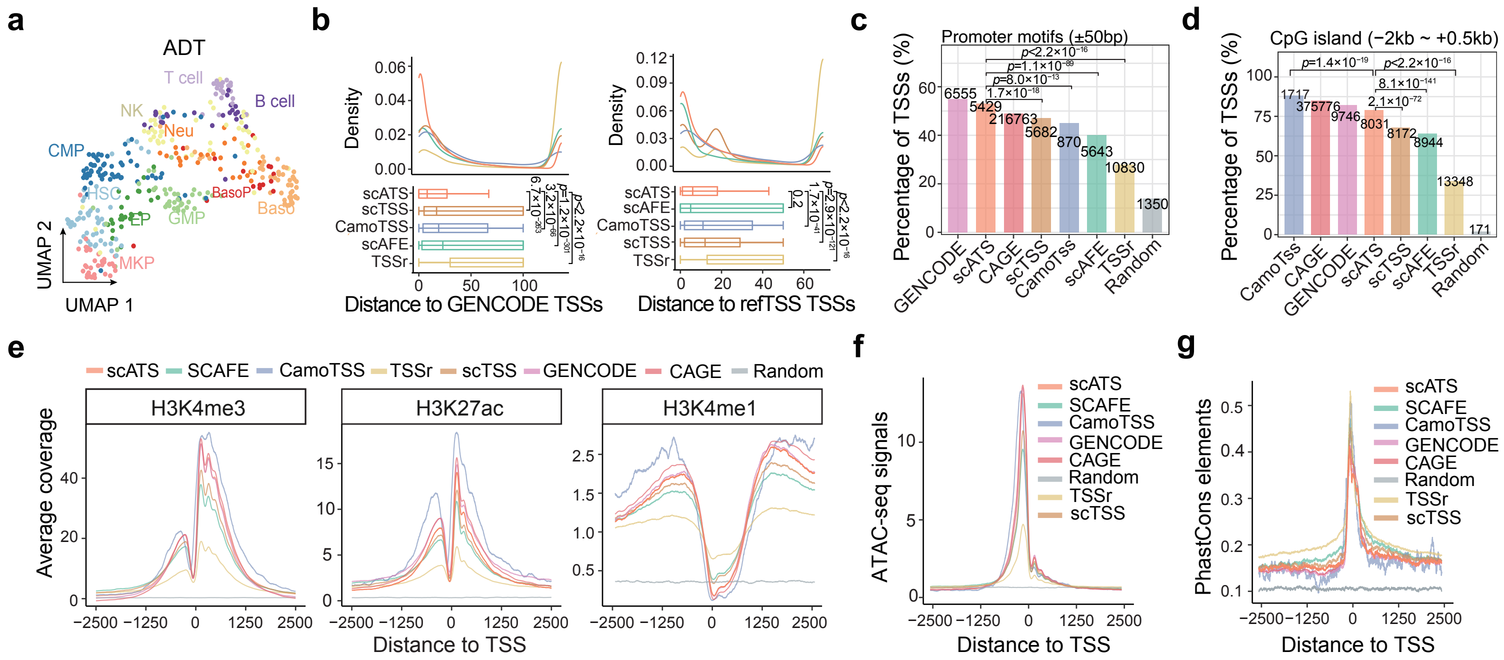

a
ADT
T cell
B cell
NK
Neu
CMP
HSC
BasoP
Baso
EP
GMP
MKP
UMAP 2
UMAP 1
b
Density
Distance to GENCODE TSSs
Distance to refTSS TSSs
scATS
scTSS
CamoTSS
scAFE
TSSr
c
Promoter motifs (±50bp)
Percentage of TSSs (%)
GENCODE
scATS
CAGE
scTSS
CamoTss
scAFE
TSSr
Random
d
CpG island (−2kb ~ +0.5kb)
e
scATS SCAFE CamoTSS TSSr scTSS GENCODE CAGE Random
H3K4me3
H3K27ac
H3K4me1
Average coverage
Distance to TSS
f
ATAC-seq signals
g
PhastCons elements

Supplementary Fig. 2 | Genomic features of identified TSSs in mHSPC datasets (related to Fig. 2).

a, UMAP plot of all cell types identified based on ADTs of the mHSPC 5’ scRNA-seq dataset. Cell-types indicated by different colors.

b, Density (upper panels) of detected TSSs by scATS, scTSS, SCAFE, CamoTSS and TSSr, and distance (lower panels) to GENCODE-annotated (left panel) and refTSS-annotated (right panel) TSSs. Box plot, the central line represents the median, the box indicates the IQR, and the whiskers represent values within 1.5 times the IQR. The distances of TSSs detected by different methods to annotated TSSs were compared.

c-g, Distribution of promoter motifs (c), CpG islands (d), histone modifications (e), ATAC-seq signals (f) and PhastCons elements (g) within the indicated distance around the GENCODE-annotated and identified TSSs. Random, genomic positions randomly sampled outside annotated TSSs. Statistical tests were performed on the percentages of TSSs in pairwise comparisons. The numbers of TSSs are shown above the bars (c) and (d).

For statistical analysis, the following tests were used: two-sided Wilcoxon test with BH correction (b) and two-sided proportion tests with BH correction (c, d). Exact *p* values are provided in the figure.

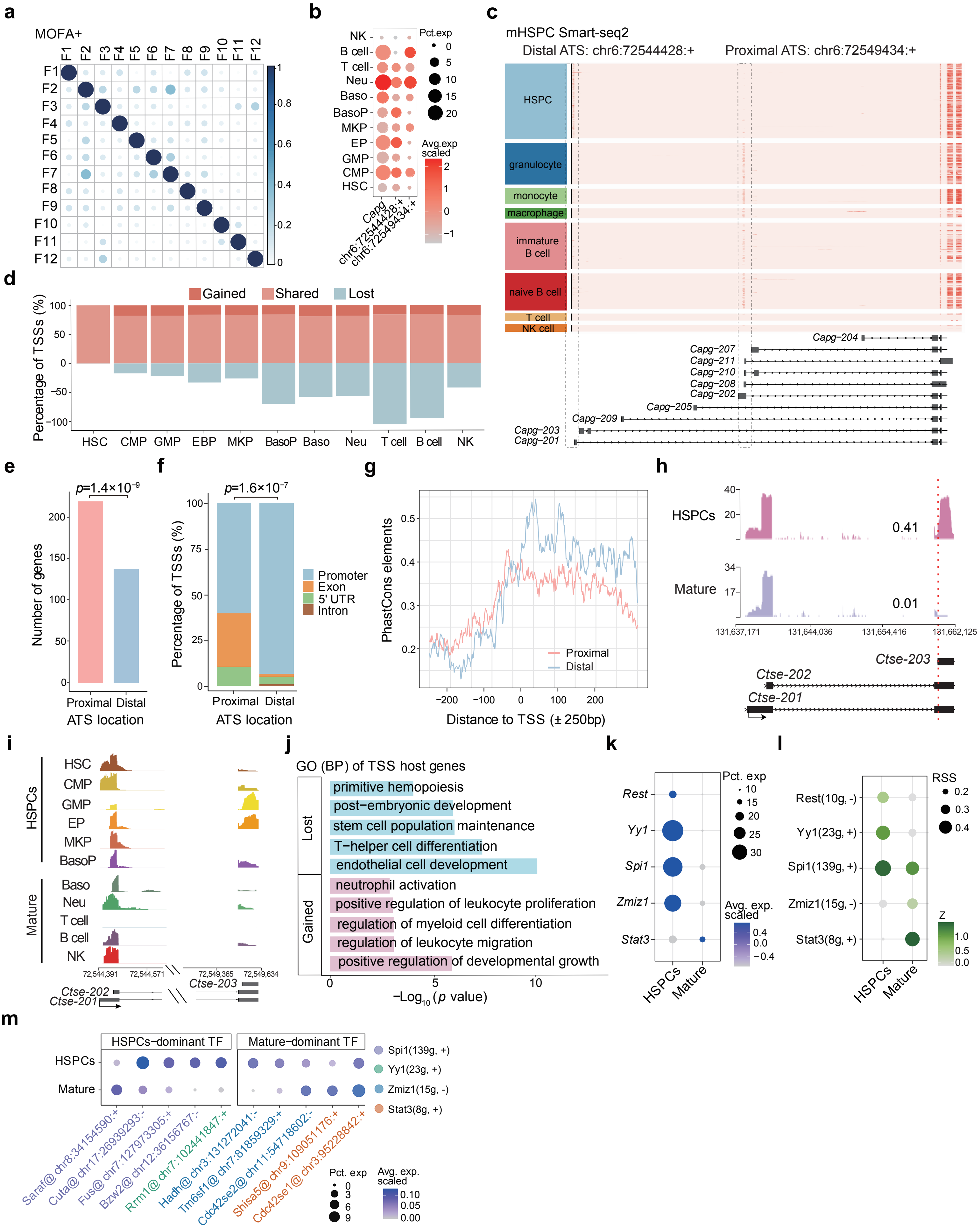

a
MOFA+
F1 F2 F3 F4 F5 F6 F7 F8 F9 F10 F11 F12
1 0.8 0.6 0.4 0.2 0
b
NK
B cell
T cell
Neu
Baso
BasoP
MKP
EP
GMP
CMP
HSC
Capg
chr6:72544428:+
chr6:72549434:+
Pct.exp
0 5 10 15 20
Avg.exp scaled
2 1 0 −1
c
mHSPC Smart-seq2
Distal ATS: chr6:72544428:+
Proximal ATS: chr6:72549434:+
HSPC
granulocyte
monocyte
macrophage
immature B cell
naive B cell
T cell
NK cell
Capg−204
Capg−207
Capg−211
Capg−210
Capg−208
Capg−202
Capg−205
Capg−209
Capg−203
Capg−201
d
Gained
Shared
Lost
Percentage of TSSs (%)
100 50 0 −50 −100
HSC CMP GMP EBP MKP BasoP Baso Neu T cell B cell NK
e
p=1.4×10−9
Number of genes
200 150 100 50 0
Proximal Distal
ATS location
f
p=1.6×10−7
Percentage of TSSs (%)
100 75 50 25 0
Promoter
Exon
5' UTR
Intron
Proximal Distal
ATS location
g
PhastCons elements
0.5 0.4 0.3 0.2
Proximal
Distal
−200 −100 0 100 200
Distance to TSS (± 250bp)
h
HSPCs
40 20
0.41
Mature
34 17
0.01
131,637,171
131,644,036
131,654,416
131,662,125
Ctse-203
Ctse-202
Ctse-201
i
HSPCs
HSC
CMP
GMP
EP
MKP
BasoP
Mature
Baso
Neu
T cell
B cell
NK
72,544,391
72,544,571
72,549,365
72,549,634
Ctse-203
Ctse-202
Ctse-201
j
GO (BP) of TSS host genes
Lost
primitive hemopoiesis
post−embryonic development
stem cell population maintenance
T−helper cell differentiation
endothelial cell development
Gained
neutrophil activation
positive regulation of leukocyte proliferation
regulation of myeloid cell differentiation
regulation of leukocyte migration
positive regulation of developmental growth
0 5 10
−Log10 (p value)
k
Rest
Yy1
Spi1
Zmiz1
Stat3
HSPCs
Mature
Pct. exp
10 15 20 25 30
Avg. exp. scaled
0.4 0.0 −0.4
l
Rest(10g, -)
Yy1(23g, +)
Spi1(139g, +)
Zmiz1(15g, -)
Stat3(8g, +)
HSPCs
Mature
RSS
0.2 0.3 0.4
Z
1.0 0.5 0.0
m
HSPCs−dominant TF
Mature−dominant TF
HSPCs
Mature
Spi1(139g, +)
Yy1(23g, +)
Zmiz1(15g, -)
Stat3(8g, +)
Saraf@chr8:34154590:+
Cuta@chr17:26939293:-
Fus@chr7:127973305:+
Bzw2@chr12:36156767:-
Rrm1@chr7:102441847:+
Hadh@chr3:131272041:-
Tm6sf1@chr7:81859329:+
Cdc42se2@chr11:54718602:-
Shisa5@chr9:109051176:+
Cdc42se1@chr3:95228842:+
Pct. exp
0 3 6 9
Avg. exp. scaled
0.10 0.05 0.00

Supplementary Fig. 3 | Summary of identified TSS in mHSPC datasets (related to Fig. 2).

a, Heatmap of Pearson correlations among all MOFA+ factors (F). Pearson correlation coefficient (*R*) indicated by color.

b, Dot plots showing the expression level of the *Capg* gene and ATSs shown in **Fig. 2h**. Fraction of expressing cells (dot size) and scaled average expression (color) indicated.

c, Sashimi plots showing transcription level of *Capg* in mHSPC Smart-seq2 dataset from Tabula Muris consortium. Dashed boxes outline ATSs, with annotations below.

d, TSS usage in HSPCs and mature cells identified in mHSPC 5'scRNA-seq dataset: shared (pink), mature-gained (red), and mature-lost (blue).

e-f, Bar charts showing the distribution of the position (e) and localisation (f) of mature-loss TSSs. Statistical tests were performed on the host gene (e) and 'Exon' ATS (f) between proximal and distal mature-lost TSSs.

g, Distribution of conservation scores (PhastCons) within the indicated distance around the proximal (pink) and distal (blue) mature-lost TSSs.

h-i, Sashimi plots showing the transcription level of *Ctse* in HSPCs and mature cells (h) and the individual cell-types (i). Proximal TSS (red dashed line), PSI values and annotations indicated.

j, GO enrichment analysis of host genes with TSSs gained or lost in mature cells (d). The bars depict -log10 (*p* value). BP, biological processes.

k-l, Dot plots showing gene expression (k) and activity level (l) of transcription factors (TFs). The dot size represents the percentage of cells expressing each gene (k) and the regulon specificity score (RSS) of TFs (l), and the color intensity indicates the scaled average expression level (k) and the Z-score (l). TF labels indicate the number of targeted genes (g) and the direction of regulation (+ or −).

m, Dot plot showing the expression level of mature-gained or lost TSSs. Dot size represents the percentage of cells expressing each gene, while color intensity indicates the scaled average expression level, with different colors indicating

distinct TFs.

For statistical analysis, the following tests were used: two-sided proportion tests (e, f) and one-sided hypergeometric tests (j). Exact *p* values are provided in the figure.

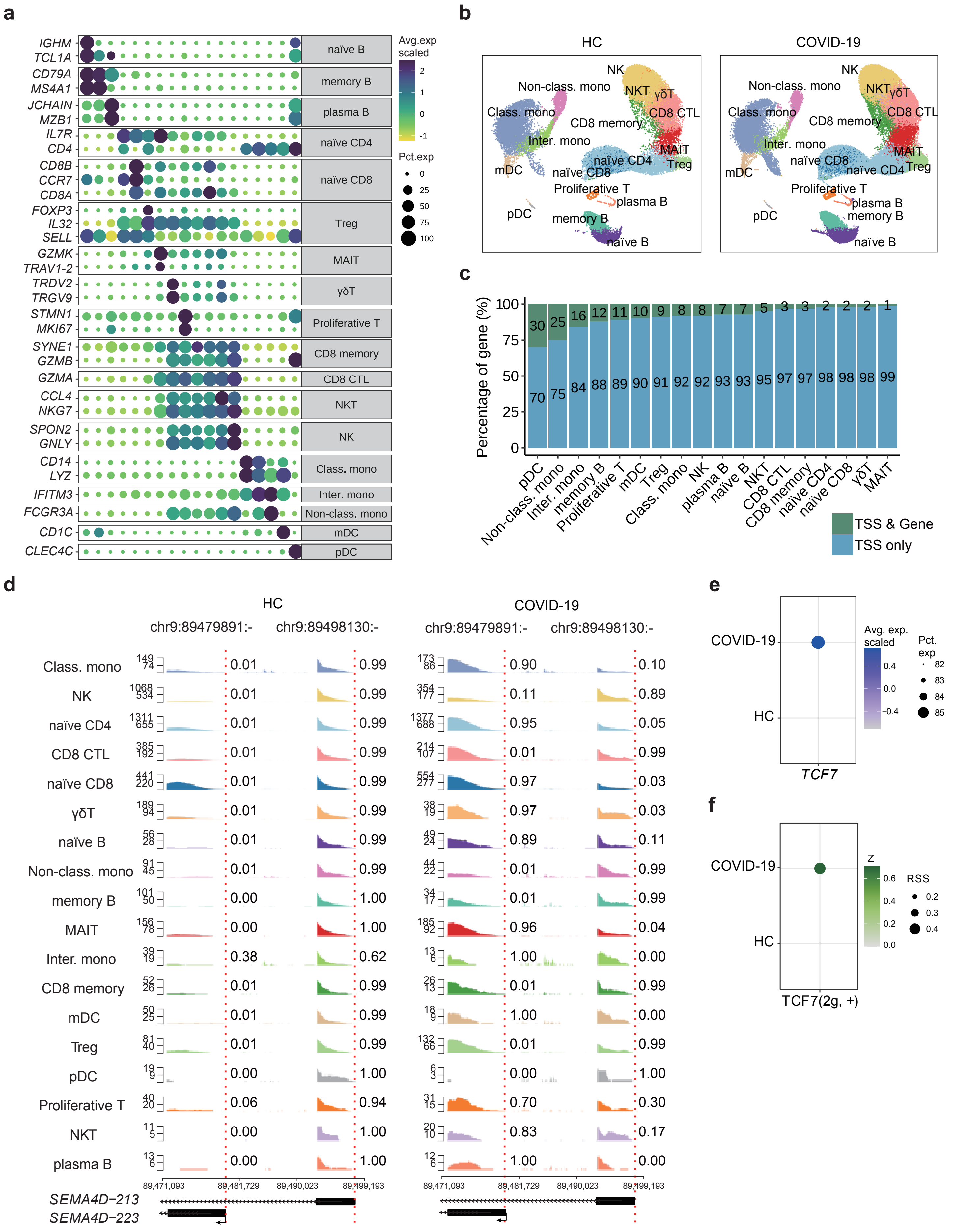

Supplementary Fig. 4 | Cell clustering and identified TSSs in the COVID-19 dataset (related to Fig. 3).

a, Dot plots visualising the expression level of the marker genes of 18 cell types. The size of the dots represents the percent of cells expressing the marker genes, while the color intensity represents the scaled average expression level.

b, UMAP plots of cells clustering based on gene expression in HCs (left panel) and COVID-19 (right panel) groups. Cell-types are indicated by different colors.

c, Proportion of host genes of TSS markers with or without support from gene markers in COVID-19 5' scRNA-seq datasets.

d, Sashimi plots showing the transcription level of the *SEMA4D* in HC (left panel) and COVID-19 (right panel) groups. The red dashed lines indicate the TSS loci with corresponding PSIs shown near the peaks. Annotations of the isoforms are shown below the sashimi plots.

e-f, Dot plots showing gene expression (e) and activity level (f) of TCF7 TF. The dot size represents the percentage of cells expressing *TCF7* gene (e) and the RSS values of TCF7 TF (f), and the color intensity indicates the scaled average expression level (e) and the Z-score (f). TF label indicate the number of targeted genes (g) and the direction of regulation (+ or −).

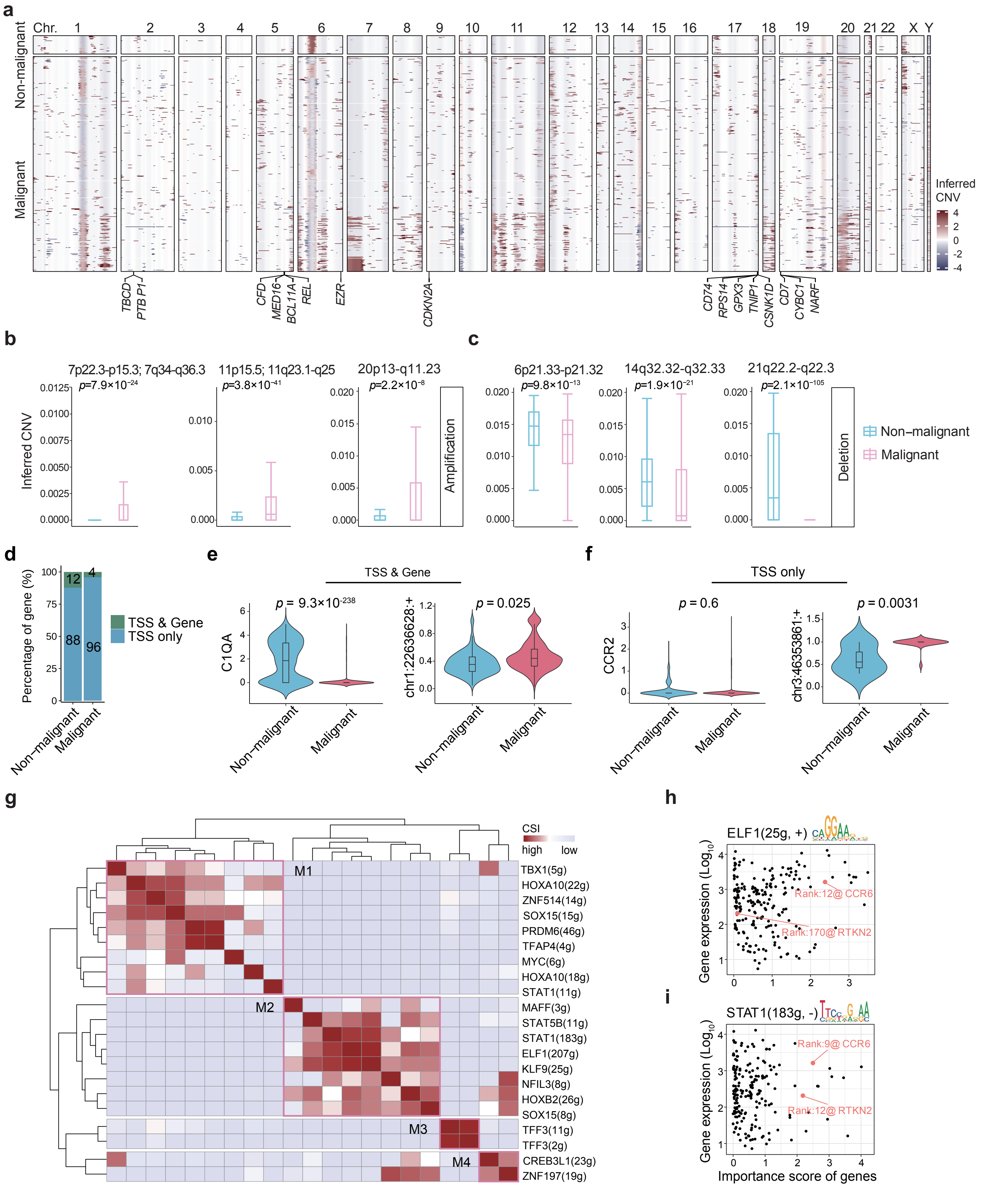

a
Chr. 1 2 3 4 5 6 7 8 9 10 11 12 13 14 15 16 17 18 19 20 21 22 X Y
Non-malignant
Malignant
Inferred CNV
4 2 0 -2 -4
TBCD PTB P1 CFD MED16 BCL11A REL EZR CDKN2A CD74 RPS14 GPX3 TNIP1 CSNK1D CD7 CYBC1 NARF
b
7p22.3-p15.3; 7q34-q36.3
p=7.9×10⁻²⁴
11p15.5; 11q23.1-q25
p=3.8×10⁻⁴¹
20p13-q11.23
p=2.2×10⁻⁸
Inferred CNV
Amplification
c
6p21.33-p21.32
p=9.8×10⁻¹³
14q32.32-q32.33
p=1.9×10⁻²¹
21q22.2-q22.3
p=2.1×10⁻¹⁰⁵
Deletion
Non–malignant
Malignant
d
Percentage of gene (%)
TSS & Gene
TSS only
12 4 88 96
Non–malignant Malignant
e
TSS & Gene
p = 9.3×10⁻²³⁸
C1QA
p = 0.025
chr1:22636628:+
f
TSS only
p = 0.6
CCR2
p = 0.0031
chr3:46353861:+
Non–malignant Malignant
g
CSI
high low
M1 M2 M3 M4
TBX1(5g)
HOXA10(22g)
ZNF514(14g)
SOX15(15g)
PRDM6(46g)
TFAP4(4g)
MYC(6g)
HOXA10(18g)
STAT1(11g)
MAFF(3g)
STAT5B(11g)
STAT1(183g)
ELF1(207g)
KLF9(25g)
NFIL3(8g)
HOXB2(26g)
SOX15(8g)
TFF3(11g)
TFF3(2g)
CREB3L1(23g)
ZNF197(19g)
h
ELF1(25g, +)
Rank:12@ CCR6
Rank:170@ RTKN2
Gene expression (Log10)
i
STAT1(183g, -)
Rank:9@ CCR6
Rank:12@ RTKN2
Gene expression (Log10)
Importance score of genes

Supplementary Fig. 5 | Single-cell transcriptomic analyses of human NSCLC dataset (related to Fig. 4).

a, Heatmap of genetic subclones identified from the inferred amplifications and deletions in malignant and non-malignant cells of the human NSCLC dataset across the chromosome regions shown as cytobands (Chr.). Genes reported to have CNVs (Copy Number Variants) in lung cancer are shown below the corresponding chromosomes.

b-c, Box plots showing CNV amplifications on chromosomes 7, 11 and 20 (b), and deletions on chromosomes 6, 14 and 21 (c). Chromosomal locations with CNV changes are shown in the cytobands in (a). Chromosome arms are designated as p (short arm) and q (long arm).

d, Proportion of host genes of TSS markers with or without gene markers support in NSCLC 5' scRNA-seq dataset.

e-f, Violin plots depicting single-cell expression levels at gene (left panels) or TSS level (right panels) in *C1QA* (e) and *CCR2* (f) genes. The central line represents the median, the box indicates the IQR, and the whiskers represent values within 1.5 times the IQR.

g, Modules (M) based on regulon connection specificity index (CSI) matrix, along with corresponding TF regulons.

h-i, Dot plot showing targeted genes of the ELF1 (h) and STAT1 (i) regulons. X-axis indicates the importance scores of targeted genes inferred by SCENIC, and the Y-axis represents the expression levels of the targeted genes, with the number of targeted genes (g) and the direction of regulation, positive (+) or negative (−) are shown. The importance ranking of *CCR6* and *RTKN2* from TF regulons are highlighted in red text. The sequence logos were generated from FIMO-identified motif occurrences within the *CCR6* and *RTKN2*.

For statistical analysis, the following tests were used: two-sided Wilcoxon test with BH correction (b, c, e, f). Exact *p* values are provided in the figure.

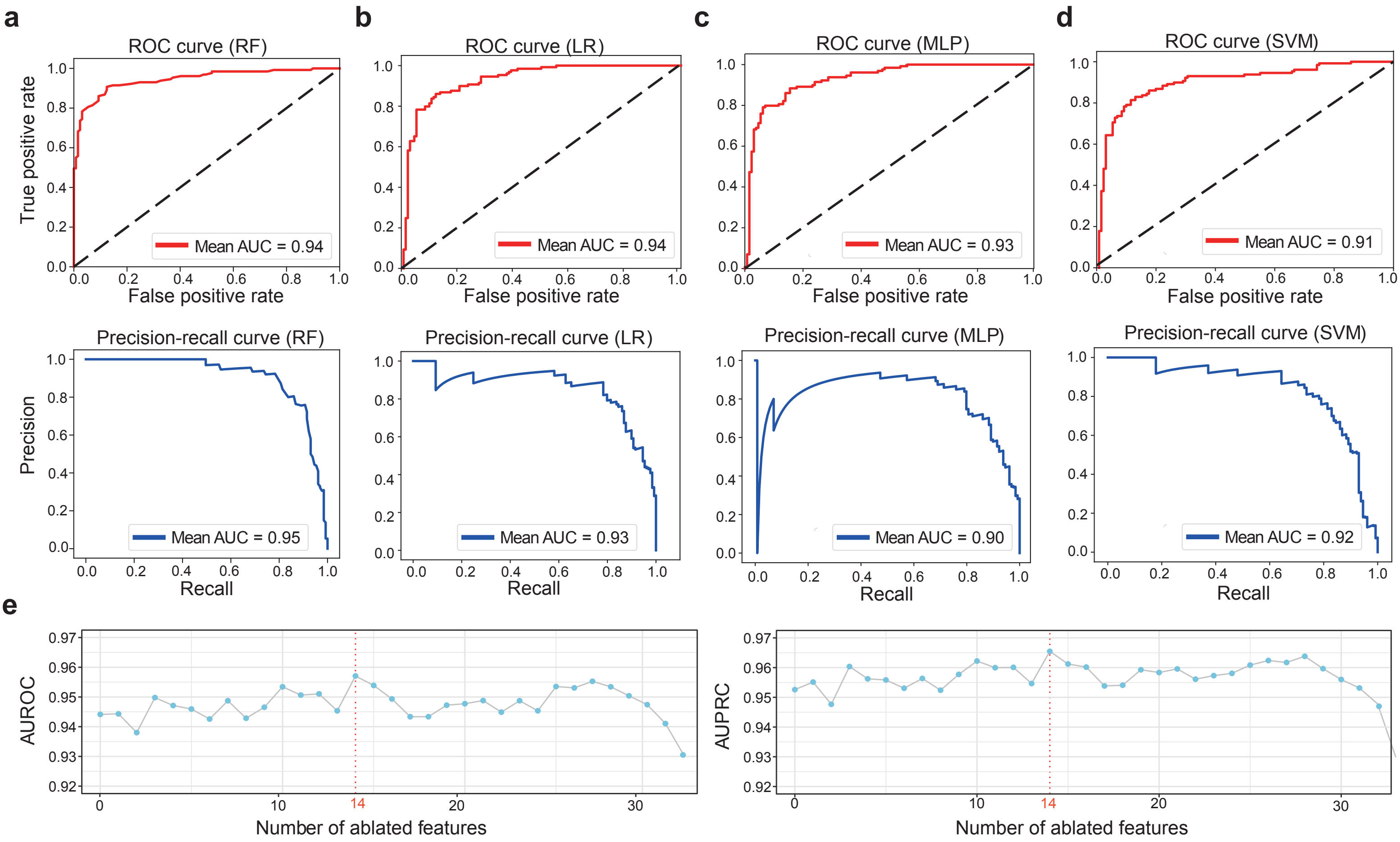

a
b
c
d
ROC curve (RF)
ROC curve (LR)
ROC curve (MLP)
ROC curve (SVM)
True positive rate
False positive rate
Mean AUC = 0.94
Mean AUC = 0.94
Mean AUC = 0.93
Mean AUC = 0.91
Precision-recall curve (RF)
Precision-recall curve (LR)
Precision-recall curve (MLP)
Precision-recall curve (SVM)
Precision
Recall
Mean AUC = 0.95
Mean AUC = 0.93
Mean AUC = 0.90
Mean AUC = 0.92
e
AUROC
AUPRC
Number of ablated features
14

Supplementary Fig. 6 | Performance of classification models and feature ablation analysis (related to Fig. 4).

a-**d**, Performance of four models: RF (a), LR (b), MLP (c), and SVM (d) assessed by AUROC (upper panels), and AUPRC (lower panels). The red and blue dashed lines represent the overall AUROC and AUPRC from 10-fold cross-validation, respectively. The mean AUCs are shown.

e, Line plots depict the model performance by AUROC (left panel) and AUPRC (right panel) under the number of ablated features. Red dashed lines indicate the number of ablated features yielding the best performance.

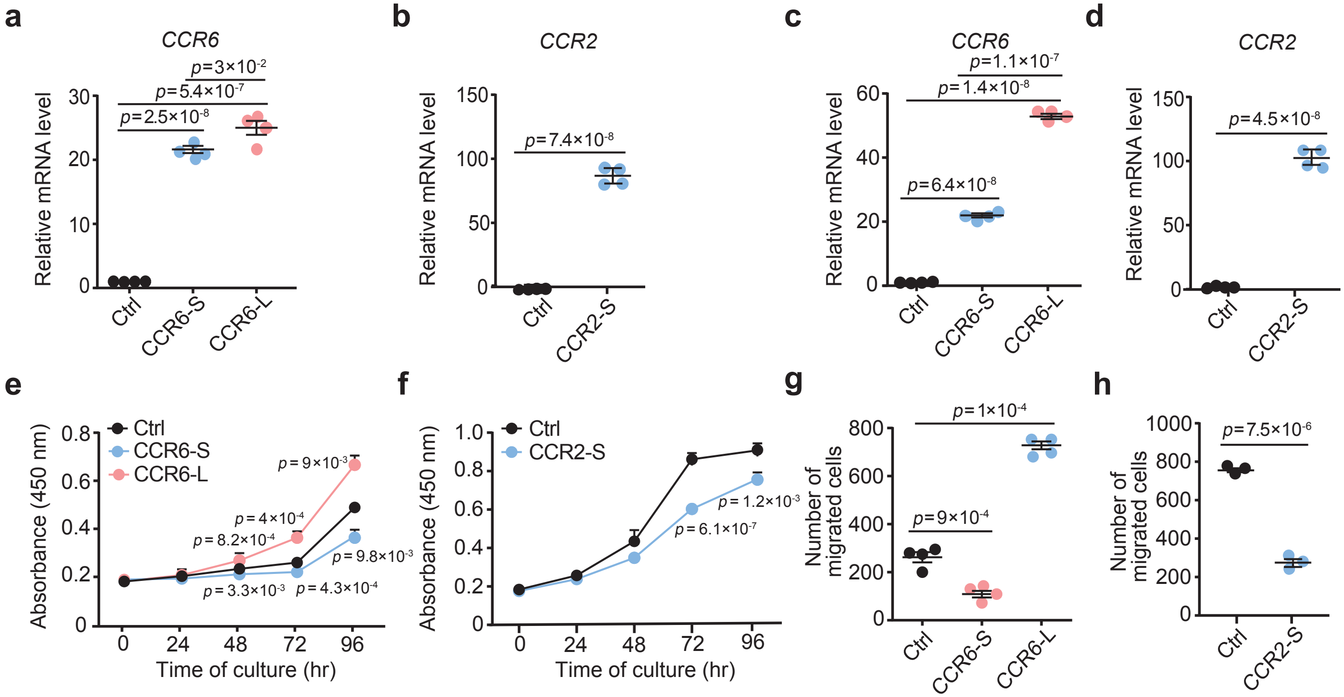
a
CCR6
p=3×10-2
p=5.4×10-7
p=2.5×10-8
Relative mRNA level
0
10
20
30
Ctrl
CCR6-S
CCR6-L
b
CCR2
p=7.4×10-8
Relative mRNA level
0
50
100
150
Ctrl
CCR2-S
c
CCR6
p=1.1×10-7
p=1.4×10-8
p=6.4×10-8
Relative mRNA level
0
20
40
60
Ctrl
CCR6-S
CCR6-L
d
CCR2
p=4.5×10-8
Relative mRNA level
0
50
100
150
Ctrl
CCR2-S
e
Ctrl
CCR6-S
CCR6-L
Absorbance (450 nm)
0
0.2
0.4
0.6
0.8
p = 9×10-3
p = 4×10-4
p = 8.2×10-4
p = 9.8×10-3
p = 3.3×10-3
p = 4.3×10-4
0
24
48
72
96
Time of culture (hr)
f
Ctrl
CCR2-S
Absorbance (450 nm)
0
0.2
0.4
0.6
0.8
1.0
p = 1.2×10-3
p = 6.1×10-7
0
24
48
72
96
Time of culture (hr)
g
p=1×10-4
p=9×10-4
Number of migrated cells
0
200
400
600
800
Ctrl
CCR6-S
CCR6-L
h
p=7.5×10-6
Number of migrated cells
0
200
400
600
800
1000
Ctrl
CCR2-S

Supplementary Fig. 7 | Overexpression assay of isoforms of *CCR2* and *CCR6* (related to Fig. 5).

a-d, Relative mRNA levels of *CCR6* or *CCR2* in overexpressing Calu-1 cells (a-b) or A549 cells (c-d) (*n* = 4). Ctrl, cells overexpressing Flag-tagged Catalase (CAT); CCR6-S/L, cells overexpressing CCR6 short (S) or long (L) isoforms; CCR2-S, overexpression of CCR2 short (S) isoform. GAPDH was used as an internal control.

e-h, A549 cells were transfected with lentivirus expressing Flag-tagged CAT (Ctrl), CCR6-S, CCR6-L, or CCR2-S and treated with puromycin for 3 days before subjecting to cell counting kit (CCK)-8 or transwell assay. Absorbance at 450 nm (*n* = 4) was measured at different time points (e, f). The numbers of migrated cells are shown for (g) (*n* = 4) and (h) (*n* = 3). Scale bars, 40 μm.

For statistical analysis, the following tests were used: Multiple unpaired two-tailed Student's t-tests (a-h). Experiments of (a-g) were repeated four times and (h) were repeated three times, consistently yielding similar results in each iteration. Data in (a-h) are represented as mean ± SEM. Data in (a-d) pooled from three independent experiments and (e-h) pooled from two independent experiments. Source data are provided as a Source Data file.

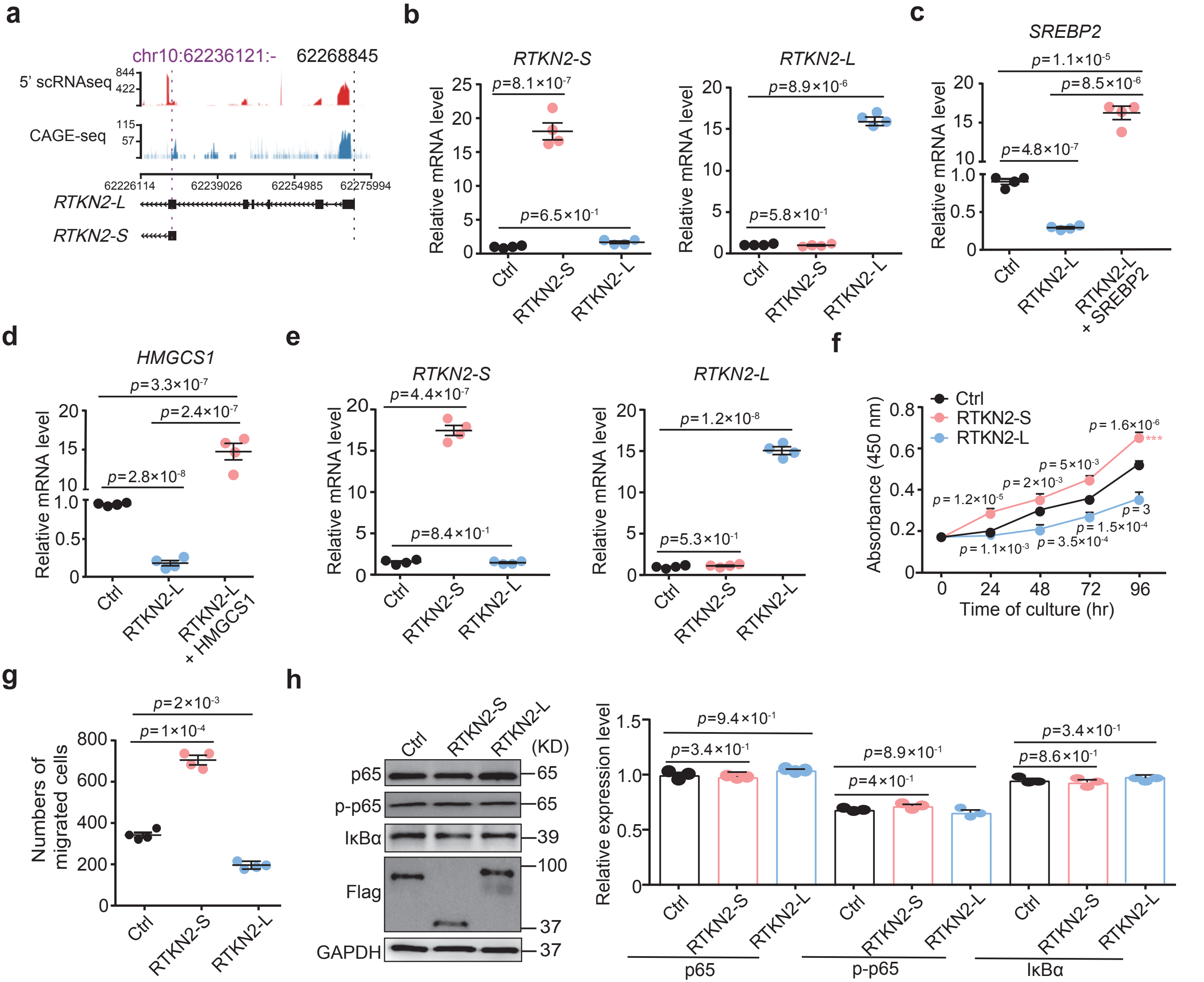
a
chr10:62236121:- 62268845
5' scRNAseq
CAGE-seq
RTKN2-L
RTKN2-S
b
RTKN2-S
RTKN2-L
Relative mRNA level
Ctrl
RTKN2-S
RTKN2-L
c
SREBP2
RTKN2-L + SREBP2
d
HMGCS1
RTKN2-L + HMGCS1
e
f
Absorbance (450 nm)
Time of culture (hr)
g
Numbers of migrated cells
h
p65
p-p65
IκBα
Flag
GAPDH
(KD)
Relative expression level

Supplementary Fig. 8 | Overexpression assay of *RTKN2-L* (related to Fig. 6).

a, Sashimi plots showing the transcription level of RTKN2 in 5' scRNA-seq (red) and CAGE-seq (blue) datasets of NSCLC patients. The corresponding dashed lines indicate the loci of predicted ATSs inferred by scATS. The TSS with higher expression in the malignant cells are highlighted in purple. Annotation shown below the sashimi plots.

b,**e**, Relative mRNA levels of the *RTKN2* isoforms in Calu-1 cells (b) or A549 cells (e) overexpressing RTKN2-S (left panels) and RTKN2-L (right panels) relative to GAPDH (*n*=4). Ctrl, cells overexpressing Flag-tagged Catalase (CAT); RTKN2-S/L, cells overexpressing RTKN2 short (S) or long (L) isoforms.

c-d, Relative mRNA levels of the *SREBP2* (c) and *HMGCS1* (d) in lentiviral transduced Calu-1 cells (*n*=4). Ctrl, cells overexpressing Flag-tagged CAT; RTKN2-L, cells overexpressing RTKN2-L; selected with puromycin for 3 days. RTKN2-L+SREBP2 or HMGCS1, cells transduced with RTKN2-L-expressing and SREBP2- or HMGCS1-expressing lentiviruses; treated with both puromycin and G418 for 3 days. GAPDH was used as an internal control.

f-g, A549 cells were transfected with lentivirus expressing Flag-tagged CAT, RTKN2-S or RTKN2-L and treated with puromycin for 3 days before subjecting to cell counting kit (CCK)-8 (f) or transwell assay (g). Absorbance at 450 nm (*n*=4) was measured at different time points. The numbers of migrated cells (*n*=4) are shown in (f). Scale bars, 40 μm.

h, Representative immunoblots (left panel) of Calu-1 cells overexpressing Flag-tagged CAT (Ctrl), RTKN2-S or RTKN2-L. The levels of CAT, RTKN2-S and RTKN2-L were detected by anti-Flag antibody. The relative signals of p65/GAPDH, p-p65/GAPDH and IκBα/GAPDH were quantified using ImageJ (right panel).

For statistical analysis, the following tests were used: Multiple unpaired two-tailed Student's t-tests (b-h). Experiments of (b-g) were repeated four times, consistently yielding similar results in each iteration. Data in (b-h) are represented as mean ± SEM. Data in (b, e, h) pooled from three independent experiments and (c-d, f-g) pooled from two independent experiments. Source data are provided as a Source Data file.

References

1. Fishman, L. *et al.* Cell-type-specific mRNA transcription and degradation kinetics in zebrafish embryogenesis from metabolically labeled single-cell RNA-seq. *Nat. Commun.* 15, (2024).
2. Pepke, S., Wold, B. & Mortazavi, A. Computation for ChIP-seq and RNA-seq studies. *Nat. Methods* 6, S22 (2009).
3. Wang, Z., Gerstein, M. & Snyder, M. RNA-Seq: a revolutionary tool for transcriptomics. *Nat. Rev. Genet.* 10, 57 (2009).
4. Fu, S. & Li, W. V. Predicting and comparing transcription start sites in single cell populations. *PLoS Comput. Biol.* 21, (2025).
5. Moody, J. *et al.* SCAFE: a software suite for analysis of transcribed cis-regulatory elements in single cells. *Bioinformatics* 38, 5126–5128 (2022).
6. Hou, R., Hon, C. C. & Huang, Y. CamoTSS: analysis of alternative transcription start sites for cellular phenotypes and regulatory patterns from 5' scRNA-seq data. *Nat. Commun.* 14, (2023).
7. Wan, L., Yan, X., Chen, T. & Sun, F. Modeling RNA degradation for RNA-Seq with applications. *Biostatistics* 13, 734–747 (2012).
8. Lawrence, M. *et al.* Software for Computing and Annotating Genomic Ranges. *PLoS Comput. Biol.* 9, (2013).
9. Lu, Z. *et al.* TSSr: an R package for comprehensive analyses of TSS sequencing data. *NAR Genom. Bioinform.* 3, (2021).
10. Fu, S. & Li, W. V. Predicting and comparing transcription start sites in single cell populations. *PLoS Comput. Biol.* 21, e1012878 (2025).
11. Paz, M. W., Vogel, T. & Nieselt, K. TSS-Captur: a user-friendly pipeline for characterizing unclassified RNA transcripts. *NAR Genom. Bioinform.* 6, (2024).
12. Bhardwaj, N., Gerstein, M. & Lu, H. Genome-wide sequence-based prediction of peripheral proteins using a novel semi-supervised learning technique. *BMC Bioinformatics* 11, (2010).
13. Zhao, Y. *et al.* Polymer-locking fusogenic liposomes for glioblastoma-targeted siRNA delivery and CRISPR–Cas gene editing. *Nat. Nanotechnol.* 19, 1869–1879 (2024).
14. Demircioğlu, D. *et al.* A Pan-cancer Transcriptome Analysis Reveals Pervasive Regulation through Alternative Promoters. *Cell* 178, 1465-1477.e17 (2019).
15. Clements, M. *et al.* Axonal injury is a targetable driver of glioblastoma progression. *Nature* 646, 452–461 (2025).
16. Han, M. H. *et al.* Identification of genes from ten oncogenic pathways associated with mortality and disease progression in glioblastoma. *Front. Oncol.* 12, (2022).
17. Hua, T. *et al.* Glioma-neuronal interactions in tumor progression: Mechanism, therapeutic strategies and perspectives (Review). *Int. J. Oncol.* 61, (2022).
18. Duncan, C. G. *et al.* Integrated genomic analyses identify ERRFI1 and TACC3 as glioblastoma-targeted genes. *Oncotarget* 1, 265–277 (2010).
19. Strehl, A. & Ghosh, J. Cluster Ensembles-A Knowledge Reuse Framework for Combining Multiple Partitions. *Journal of Machine Learning Research* 3, 583–617 (2002).
20. Gates, A. J. & Ahn, Y.-Y. The Impact of Random Models on Clustering Similarity. *Journal of Machine Learning Research* 18, 1–28 (2017).
