# Supplementary material for: Accurate profiling of single-cell alternative transcript start sites by correcting RNA degradation
Source: Nat Commun. 2026 Apr 28;17:5798. doi: 10.1038/s41467-026-72298-8 (PMC13332059; doi:10.1038/s41467-026-72298-8)
Supplement: Supplementary file 2 — Description of Additional Supplementary Files [file 41467_2026_72298_MOESM2_ESM.pdf]

Description of Additional Supplementary Files

Supplementary Data 1. Metadata of datasets used in this study.

Supplementary Data 2. Comparison of various features of tools in this study.

Supplementary Data 3. TSS quantification by scATS.

Supplementary Data 4. Cell-type-specific TSSs in COVID-19 5′ scRNA-seq**.**

Supplementary Data 5. GO enrichments of cell-type-specific TSSs in COVID-19 5′ scRNA-seq.

Supplementary Data 6. Host gene information of positive set.

Supplementary Data 7. Features used for LRS model.

Supplementary Data 8. Feature ablation study.

Supplementary Data 9. Prediction by the LRS model.

Supplementary Data 10. Epigenomic and chromatin accessibility datasets used in this study.

Supplementary Data 11. Antibodies and oligos used in this study.
