# Supplementary material for: Accurate profiling of single-cell alternative transcript start sites by correcting RNA degradation
Source: Nat Commun. 2026 Apr 28;17:5798. doi: 10.1038/s41467-026-72298-8 (PMC13332059; doi:10.1038/s41467-026-72298-8)
Supplement: Supplementary file 14 — Reporting Summary [file 41467_2026_72298_MOESM14_ESM.pdf]

nature portfolio

Corresponding author(s): Jing-wen Lin and Lu Chen

Last updated by author(s): Mar 26, 2026

Reporting Summary

Nature Portfolio wishes to improve the reproducibility of the work that we publish. This form provides structure for consistency and transparency in reporting. For further information on Nature Portfolio policies, see our Editorial Policies and the Editorial Policy Checklist.

Statistics

For all statistical analyses, confirm that the following items are present in the figure legend, table legend, main text, or Methods section.

n/a	Confirmed	
☐	☒	The exact sample size (*n*) for each experimental group/condition, given as a discrete number and unit of measurement
☐	☒	A statement on whether measurements were taken from distinct samples or whether the same sample was measured repeatedly
☐	☒	The statistical test(s) used AND whether they are one- or two-sided<br>*Only common tests should be described solely by name; describe more complex techniques in the Methods section.*
☒	☐	A description of all covariates tested
☐	☒	A description of any assumptions or corrections, such as tests of normality and adjustment for multiple comparisons
☐	☒	A full description of the statistical parameters including central tendency (e.g. means) or other basic estimates (e.g. regression coefficient) AND variation (e.g. standard deviation) or associated estimates of uncertainty (e.g. confidence intervals)
☐	☒	For null hypothesis testing, the test statistic (e.g. *F*, *t*, *r*) with confidence intervals, effect sizes, degrees of freedom and *P* value noted<br>*Give P values as exact values whenever suitable.*
☒	☐	For Bayesian analysis, information on the choice of priors and Markov chain Monte Carlo settings
☒	☐	For hierarchical and complex designs, identification of the appropriate level for tests and full reporting of outcomes
☐	☒	Estimates of effect sizes (e.g. Cohen's *d*, Pearson's *r*), indicating how they were calculated

Our web collection on statistics for biologists contains articles on many of the points above.

Software and code

Policy information about availability of computer code

Data collection	The raw 5’ and 3’ mHSPC scRNA-seq and scONT data generated in this study have been deposited in the Gene Expression Omnibus (GEO) database under accession code GSE302632 (https://www.ncbi.nlm.nih.gov/geo/query/acc.cgi?acc=GSE302632). The raw sequencing data of in-house mHSPC datasets generated using Smart-seq2, ONT, and PacBio in this study are available in the European Nucleotide Archive (ENA) database under accession code PRJNA706066 (https://www.ebi.ac.uk/ena/browser/view/PRJNA706066). The raw Smart-seq2 data of mHSPCs from the Tabula Muris consortium used in this study are available in the GEO database under accession code GSE109774 (https://www.ncbi.nlm.nih.gov/geo/query/acc.cgi?acc=GSE109774). The raw 5’ scRNA-seq data of human NSCLC samples used in this study are available from 10x Genomics (https://s3-us-west-2.amazonaws.com/10x.files/samples/cell-vdj/2.2.0/vdj_v1_hs_nsclc_5gex/vdj_v1_hs_nsclc_5gex_fastqs.tar). Source Data are provided with this paper. The processed data of COVID-19 5’ scRNA-seq datasets used in this study, together with the resources required to reproduce the LRS analysis, have been deposited in the Figshare repository (https://doi.org/10.6084/m9.figshare.31857199). All newly generated plasmids and other relevant materials are available upon request from the corresponding author.
Data analysis	The scATS code, documentation, and tutorials are available on GitHub (https://github.com/LuChenLab/r-scATS.git).<br><br>For scRNA-seq data of mHSPCs, COVID-19 and NSCLC: Reads were processed using Cell Ranger software (v4.0.0) with default parameters. Both 5' scRNA-seq and 3' scRNA-seq datasets of mHSPC were mapped to the mouse genome (mm10), while the COVID-19 and NSCLC datasets were mapped to the human genome (hg38). Low-quality cells were filtered using dataset-specific criteria: mHSPCs (gene count < 400, mitochondrial ratio > 25%), COVID-19 (gene count < 500, mitochondrial ratio > 20%), and NSCLC (gene count < 300, mitochondrial ratio > 15%).<br><br>For mHSPC Smart-seq2 datasets: Analysis was performed using the Seurat (v4.0.5) package. For quality control, cells were filtered using

dataset-specific thresholds: in-house dataset (gene count < 500 or unique molecular identifier [UMI] count < 50,000) and Tabula Muris dataset (gene count < 500 or > 8,000, UMI count < 70,000 or > 5,000,000). The filtered datasets were then processed through the standard Seurat workflow, including data normalization, variable gene selection, dimensionality reduction, clustering, and visualization to identify distinct cell-types for the downstream analysis.

For mHSPC scONT, ONT and PacBio datasets: The Pychopper (v2.5.0) software was used to identify, orient and trim long-read sequencing reads with default parameters. The number of reads, read length and quality were analyzed using NanoComp software (v1.33.1) with the parameter of '–raw–store–tsv_stats' and visualized using the R package ggplot2 (v3.5.1). Pychopper-processed reads were aligned to the GRCm38 genome using minimap2 (v2.17) with the following parameters: minimap2 -ax splice -uf --secondary=no -C5 --MD. Then, mismatches, insertions, deletions, and non-canonical splice sites in aligned reads were corrected using TranscriptClean (v2.0.3) software. Finally, TranscriptClean-corrected reads were aligned again to the GRCm38 genome.

For the grey value of the WB quantified by ImageJ (1.52a, https://github.com/imagej)

For manuscripts utilizing custom algorithms or software that are central to the research but not yet described in published literature, software must be made available to editors and reviewers. We strongly encourage code deposition in a community repository (e.g. GitHub). See the Nature Portfolio guidelines for submitting code & software for further information.

Data

Policy information about availability of data

All manuscripts must include a data availability statement. This statement should provide the following information, where applicable:

- Accession codes, unique identifiers, or web links for publicly available datasets
- A description of any restrictions on data availability
- For clinical datasets or third party data, please ensure that the statement adheres to our policy

The raw 5' and 3' mHSPC scRNA-seq and scONT data generated in this study have been deposited in the Gene Expression Omnibus (GEO) database under accession code GSE302632 (https://www.ncbi.nlm.nih.gov/geo/query/acc.cgi?acc=GSE302632). The raw sequencing data of in-house mHSPC datasets generated using Smart-seq2, ONT, and PacBio in this study are available in the European Nucleotide Archive (ENA) database under accession code PRJNA706066 (https://www.ebi.ac.uk/ena/browser/view/PRJNA706066). The raw Smart-seq2 data of mHSPCs from the Tabula Muris consortium used in this study are available in the GEO database under accession code GSE109774 (https://www.ncbi.nlm.nih.gov/geo/query/acc.cgi?acc=GSE109774). The raw 5' scRNA-seq data of human NSCLC samples used in this study are available from 10x Genomics (https://s3-us-west-2.amazonaws.com/10x.files/samples/cell-vdj/2.2.0/vdj_v1_hs_nsclc_5gex/vdj_v1_hs_nsclc_5gex_fastqs.tar). Source Data are provided with this paper. The processed data of COVID-19 5' scRNA-seq datasets used in this study, together with the resources required to reproduce the LRS analysis, have been deposited in the Figshare repository (https://doi.org/10.6084/m9.figshare.31857199). All newly generated plasmids and other relevant materials are available upon request from the corresponding author.

Research involving human participants, their data, or biological material

Policy information about studies with human participants or human data. See also policy information about sex, gender (identity/presentation), and sexual orientation and race, ethnicity and racism.

Reporting on sex and gender	All the data used in our study are publicly available. This information has not been collected.
Reporting on race, ethnicity, or other socially relevant groupings	All the data used in our study are publicly available. This information has not been collected.
Population characteristics	All the data used in our study are publicly available. This information has not been collected.
Recruitment	All the data used in our study are publicly available. This information has not been collected.
Ethics oversight	All the data used in our study are publicly available. This information has not been collected.

Note that full information on the approval of the study protocol must also be provided in the manuscript.

Field-specific reporting

Please select the one below that is the best fit for your research. If you are not sure, read the appropriate sections before making your selection.

☒ Life sciences ☐ Behavioural & social sciences ☐ Ecological, evolutionary & environmental sciences

For a reference copy of the document with all sections, see nature.com/documents/nr-reporting-summary-flat.pdf

Life sciences study design

All studies must disclose on these points even when the disclosure is negative.

Sample size	Two female C57BL/6J mice were used as biological sources. No formal sample size calculation was performed, as the purpose was exploratory profiling rather than hypothesis testing. The sample sizes chosen are sufficient to support the conclusions drawn from in vitro functional assays and provide representative single-cell transcriptomic data.
Data exclusions	No data were excluded from this study.

Replication	All experiments were replicated at least three times. We confirm that all attempts at replication were successful.
Randomization	*Describe how samples/organisms/participants were allocated into experimental groups. If allocation was not random, describe how covariates were controlled OR if this is not relevant to your study, explain why.*
Blinding	For the transwell assay and proliferation assay, All experiments were performed with investigators blinded to group allocation during data collection and analysis. Group identities were coded, and decoding was only performed after all measurements and analyses were completed to ensure unbiased data acquisition.

Reporting for specific materials, systems and methods

We require information from authors about some types of materials, experimental systems and methods used in many studies. Here, indicate whether each material, system or method listed is relevant to your study. If you are not sure if a list item applies to your research, read the appropriate section before selecting a response.

Materials & experimental systems

n/a	Involved in the study
☐	☒ Antibodies
☐	☒ Eukaryotic cell lines
☒	☐ Palaeontology and archaeology
☐	☒ Animals and other organisms
☒	☐ Clinical data
☒	☐ Dual use research of concern
☒	☐ Plants

Methods

n/a	Involved in the study
☒	☐ ChIP-seq
☒	☐ Flow cytometry
☒	☐ MRI-based neuroimaging

Antibodies

Antibodies used	Antibodies used for immunoprecipitation and WB:<br>The blots were probed with the primary antibodies as follows: anti-rabbit Flag-Tag antibody (1:1000) (Cell Signaling Technology, #14793), rabbit anti-Myc-Tag antibody (1:1000) (Cell Signaling Technology, #2278), anti-rabbit SREBP2 antibody (1:1000) (Abcam, #ab30682), anti-mouse HMGCR antibody (1:1000) (Abcam, #ab242315), anti-rabbit HMGCS1 antibody (1:1000) (Cell Signaling Technology, #42201), anti-rabbit FASN antibody (1:1000) (Cell Signaling Technology, #3180), anti-mouse LDLR antibody (1:1000) (Proteintech, #66414), anti-rabbit NF-κB p65 antibody (1:1000) (HuaBio, #HA0815), anti-mouse IκBα antibody (1:1000) (BioVision, #3252), anti-rabbit phospho-NF-κB p65 (Ser536) (1:1000) (Cell Signaling Technology, # 3033), anti-mouse GAPDH antibody (1:1000) (Cell Signaling Technology, #2118), anti-rabbit CCR2 antibody (1:1000) (Proteintech, #30420) and anti-rabbit Lamin B1 antibody (1:1000) (Cell Signaling Technology, #13435) in the universal antibody diluent (NCM biotech, #WB500D) at 4°C overnight, washed 3 times with TBST, and then incubated with the anti-mouse IgG HRP (1:5000) (Cell Signaling Technology, #7074) or anti-rabbit IgG HRP (1:5000) (Cell Signaling Technology, #7076).
Validation	All the antibodies have been validated for the use of immunoprecipitation and WB analyses. Data are available on the manufacturer's website.

Eukaryotic cell lines

Policy information about cell lines and Sex and Gender in Research

Cell line source(s)	A549, Calu-1 and HEK293T were originally obtained from the American Type Culture Collection (ATCC).
Authentication	NO additional authentication was performed.
Mycoplasma contamination	Cell lines were tested negative for mycoplasma.
Commonly misidentified lines (See ICLAC register)	No commonly misidentified cell lines were used in the study.

Animals and other research organisms

Policy information about studies involving animals; ARRIVE guidelines recommended for reporting animal research, and Sex and Gender in Research

Laboratory animals	C57BL/6J mice (n=2) were purchased from Jiangsu JCYK Bioscience Co. Ltd (Jiangsu, China), housed and bred under SPF conditions (Specific Pathogen Free) at the Laboratory Animal Center of West China Second University Hospital
Wild animals	The study did not involve wild animals.
Reporting on sex	Female mice at about 8 weeks were used in the primary experiments. Mouse sex was confirmed by gonadal inspection at 4 weeks of age.Two female C57BL/6J mice were used as biological sources. No formal sample size calculation was performed, as the purpose

	was exploratory profiling rather than hypothesis testing.
Field-collected samples	The study did not involve samples collected from the field.
Ethics oversight	Mice experiments were carried out following the protocols approved by the ethics committee of West China Second University Hospital[(2018) Animal Ethics Approval No.004]

Note that full information on the approval of the study protocol must also be provided in the manuscript.

Plants

Seed stocks	The study did not involve Plants.
Novel plant genotypes	The study did not involve Plants.
Authentication	The study did not involve Plants.
