# Supplementary material for: Accurate profiling of single-cell alternative transcript start sites by correcting RNA degradation
Source: Nat Commun. 2026 Apr 28;17:5798. doi: 10.1038/s41467-026-72298-8 (PMC13332059; doi:10.1038/s41467-026-72298-8)
Supplement: Supplementary file 15 — Transparent Peer Review file [file 41467_2026_72298_MOESM15_ESM.pdf]

nature portfolio

Peer Review File

Accurate profiling of single-cell alternative transcript start sites by correcting RNA degradation

Corresponding Author: Professor Lu Chen

This file contains all reviewer reports in order by version, followed by all author rebuttals in order by version.

Version 0:

Reviewer comments:

Reviewer #1

(Remarks to the Author)
The authors introduce the scATS method to estimate TSS usage in single-cell RNA seq based on a read coverage model that weights alternative TSS clusters by 3' coverage, extending an existing degradation model of exponential 3' coverage decay to explicitly take into account the uniformity of start site products within de-novo TSS clusters. The authors compare predicted TSS selection in single cell blood data to clusters identified by camoTSS and SCAFE. In contrast to these existing approaches, scATS offers a more interpretable locus model, which is trained directly on the data, avoiding the requirement of SCAFE and camoTSS for matched epigenetic data to achieve optimal performance. This provides a significant benefit to users and increases the portability of scATS.
To validate the regularization of TSS discovery in the context of degradation, the authors conducted full length Oxford Nanopore RNA sequencing on matched mHSPC samples as a positive control for non-degraded transcript counts. Finally, the authors conducted a range of functional assays to investigate the signaling implications of lung cancer loci whose relevance was predicted based on embeddings of scATS profiled sequencing data.
Major comments:
1. This manuscript introduces background material highlighting the impact of degradation on the detection of differentially expressed loci and ATS. However, it is unclear to what extent the targets of various functional assays the authors performed were actually substantially degraded. Please clearly state and quantify this phenomenon. In particular, did the LRS components that were interrogated for further study comprise loci whose ATS was undetectable in SCAFE, camoTSS, etc?
2. The full length mHSPC sequencing is a powerful control for degradation sensitive TSS quantitation that scATS provides. However, only a couple genes are described here. Please add some quantitative characterization of the global rate of scATS-mediated dropoff in transcripts that don't show up in the scONT dataset, presumably because they don't have a 3' end that can be primed in full length sequencing, in order to expand on this important benchmark.
3. The mapping efficiency of 10X 5' reads is highly dependent on library construction. Please list detailed run parameters for the NovaSeq 6000 runs including cycle counts
4. SCAFE requires careful pre-processing, including the selection of BAM reads containing un-annotated G's. The authors thoroughly detail pre-processing of data for camoTSS but omit these details for other methods. Please update the methods detailing how BAMs were pre-processed for SCAFE, in particular, any steps that differ from the preprocessing of data for other benchmarked. Additionally, similar to the already included camoTSS, details, please provide precise values for SCAFE, including its window size, min tss count, softclip length, etc.
5. The authors benchmarked scATS against camoTSS and SCAFE, which both utilize pretrained classifiers (which may also be fine-tuned). As such, their main weakness is the
requirement for matched ATAC data, which the authors of the current manuscript do not provide. It is therefore unclear to what extent the newly presented benchmarks simply

exploit the many possible training biases of these existing methods, including their vanilla weights derived from human circulating blood cells and fibroblasts, in contrast to scATS, which fits a de-novo model. The authors actually highlight this training background as a source of benchmarking bias, showing that almost 40% of genes of annotated in which annotated TSS are differentially regulated between in mature circulating and bone marrow cells in fig. 2k.
A more informative comparison would be against another parametric mixture model which also explicitly enumerates de-novo clusters in the indicator-dependent fashion of scATS. Fortunately, such a model has already been published (scTSS, PLOS Comp BIo 2025). The choice of scTSS is deliberate because the authors have already conducted paired end 5'-scRNA seq, which allows scTSS to utilize the R2 read for more accurate TSS selection, and which is expected to provide some automatic resistance to contamination of the read pool by degraded RNA.

Reviewer #2

(Remarks to the Author)
I co-reviewed this manuscript with one of the reviewers who provided the listed reports. This is part of the Nature Communications initiative to facilitate training in peer review and to provide appropriate recognition for Early Career Researchers who co-review manuscripts.

Reviewer #3

(Remarks to the Author)
Summary
The manuscript introduces scATS, a computational method designed to correct RNA degradation effects and improve transcription start site (TSS) identification quantification from single-cell RNA-seq data. The method is implemented as an R package and applied to various biological contexts, including lung cancer, COVID-19, and hematopoietic development. In addition, the authors propose a machine-learning–based lung cancer relevance score (LRS) to identify disease-associated TSSs. At the end, the results are supported by several isoform-level experimental validations.
While the study addresses a biologically significant question — accurate detection and quantification of TSSs at single-cell resolution, the manuscript's methodological novelty and resource contribution are limited. Although the transcription start sites topic is of high significance, more systematic benchmark and clarification for the higher performance of scATS and more rigorous presentation are required to support the claimed advances.

Here are the points that I think is helpful to improve the manuscript:

1. Method
The paper describes three core components of scATS: RNA degradation evaluation, TSS identification, and TSS quantification. It remains unclear how degradation correction is implemented and how much improvement it yields compared with other tools addressing degradation (like DeepKINET, https://genomebiology.biomedcentral.com/articles/10.1186/s13059-024-03367-8). It would be helpful to use some of the simulated degradation data which the ground truth is known to evaluate the scATS in correcting degradation.
Scores such as alpha and beta are not clearly explained — which of them actually reflects degradation level?
The identification and quantification of TSSs should be evaluated independently, using appropriate benchmarks. The methods included in the benchmark are not systemic, some of existing methods are missing (TSSr, TSS-Captur. etc).
Some important data, like CAGE-seq data which is considered a gold standard for TSS validation is missing.
The confidence or thresholding strategy for defining TSSs is not described. Does scATS provide adjustable parameters to control detection stringency?
In Figure 1c, the CamoTSS line appears flat. Is there any cut-off for CamoToss?
In Figure 1e, the statement "5′ scRNA-seq datasets exhibit highly variable evenness and 3′ bias" is not visually supported by the plot. The three curves look nearly identical, and the zoomed-in view seems inconsistent with the top panel.
The LRS model appears disease-specific. It is unclear whether it can generalize to other cancer types or disease conditions.

2. Data analysis
The data provided in this study is not systematic. If it's just used as an application example, then the method is not strong enough and the application is too much. If it's provided as "accurate systemic profiling of single-cell alternative transcript start sites" as title then, it's maybe not systematic/representitive.
The analysis sections (e.g., pathway enrichment) are superficial and unsystematic. More comprehensive functional analysis related to the start site would strengthen the biological interpretation.
Additional validation on independent datasets would be valuable. The current examples feel more illustrative than conclusive.

3. Writing
The structure of the abstract is not that clear. The abstract mentioned cancer but no COVID-19, however COVID-19 is one of the keywords in the first page. The abstract and the keywords should be reorganized so that people can mainly/roughly understand that it is the paper and there shouldn't be too many small things in the abstracts and keywords. Too many key words that confuse people.

Reviewer #4

(Remarks to the Author)
General Assessment
The study by Xu et al. presents a robust and thoughtfully executed piece of work introducing a novel software tool that substantially advances current methods for identifying transcription start sites (TSS) and distinguishing between degraded and intact transcripts. The results are noteworthy, as the authors demonstrate clear improvements in accuracy and resolution across multiple datasets compared with existing approaches, highlighting the software's potential value for transcriptomic studies.
The work is likely to be of significant interest to researchers in transcriptomics and related fields, as it addresses an important analytical gap with a well-designed computational solution. The conclusions are generally well supported by the data, and the inclusion of experimental validation adds credibility to the claims. The methodology appears sound and meets accepted standards in the field, although providing additional detail on certain implementation parameters and recommended sequencing depths would enhance reproducibility and user adoption.

Specific Comments
Software
• It would be helpful if the authors could provide guidance on the recommended sequencing depth for optimal performance of this software. Given that transcription start site identification and classification (e.g., degraded vs. intact transcripts) can be highly sensitive to read coverage, particularly around 5′ ends, information on minimal and ideal read depths would assist users in experimental planning. This is especially relevant for complex datasets containing multiple cell types, where the effective sequencing depth per cell type can vary substantially depending on relative cell abundances and transcriptional activity. Clarifying these requirements or providing benchmarking data across a range of sequencing depths would strengthen the practical utility of the tool.
• I noticed that the software repository does not list dependencies (e.g., required Python libraries) needed for installation. Including this information would improve ease of installation and reproducibility for new users.

Manuscript
• Line 193: "TDD identified by both scATS and SCAFE account for 69%..." — The second half of this sentence does not make sense ("whole only…"). Please revise for clarity.
• Line 238: The introduction of Capg in the second part of the paragraph is confusing. The first part clearly describes the 12 factors identified from MOFA analysis, but the rationale for introducing Capg here is unclear. Was Capg a major contributor to the F2 signal?
• Figure 3i / Capg discussion: For neurons, there appear to be two TSSs, each contributing roughly 50% of the signal. However, there is also some broad signal near the start of transcript Capg-204. Was this signal considered, or was it interpreted as degradation and therefore excluded from the final analysis? Clarification would help readers understand how diffuse signals are treated by the method.
• Figure 2k and associated text: Was there a trend in the loss of TSS (i.e., were distal or proximal sites preferentially lost)? Including this information could provide additional biological insight.
• Line 278: Typographical error — "sperated" should be changed to "separated."
• Figure 6 (Rtkn2): The alternating transcription start sites of Rtkn2 is convincing. However, the Rtkn2 gene model suggests two transcript lengths can arise from the distal ATS (refer GENCODE annotations). Can the authors be confident that this distal site produces only the long isoform? A cross-reference to GTEx IGV data shows that the short isoform can also be expressed (coverage of the 3′UTR at chr10:62,238,149–62,239,643). Discussing this would clarify whether the distal site is strictly associated with the long isoform.

Summary
Overall, this manuscript introduces a valuable methodological advance and provides convincing supporting data. With minor clarifications in the manuscript text, additional benchmarking on sequencing depth requirements, and improvements to software documentation, the work will be further strengthened and broadly useful to the community.

Version 1:

Reviewer comments:

Reviewer #1

(Remarks to the Author)
I co-reviewed this manuscript with one of the reviewers who provided the listed reports. This is part of the Nature Communications initiative to facilitate training in peer review and to provide appropriate recognition for Early Career Researchers who co-review manuscripts.

(Remarks on code availability)

Reviewer #2

(Remarks to the Author)
I co-reviewed this manuscript with one of the reviewers who provided the listed reports. This is part of the Nature Communications initiative to facilitate training in peer review and to provide appropriate recognition for Early Career Researchers who co-review manuscripts.

(Remarks on code availability)
The r-scATS package appears complete with a nice github repo.
However, one omission which none of the reviewers (including myself) brought up is that no paper reproducibility code (e.g. https://zenodo.org/records/7023163) is linked in the r-scATS repo or the paper. Please provide this critical supplement.

Reviewer #3

(Remarks to the Author)
Summary

The authors have improved the manuscript by incorporating evaluations on simulated datasets, expanding their analysis, and providing a more detailed description of their methodology. The updated explanation of the degradation model is much clearer, and this revision addresses the majority of the concerns raised in the previous round of review.

Despite these improvements, the unique utility and novelty of the proposed method remain unconvincing in the following areas:

1. Novelty: the TSS identification and quantification will be valuable only if it can provide additional information that gene and isoform can not provide. Currently, the manuscript does not sufficiently showcase the distinct advantages of scATS .For example, scATS enable to identify the novel TSS which does not exist in annotation, but can be captured by scATS and also supported by the external data like CAGE or ONT or PacBio. Specifically, in the figure 2h, the distal ATS links to Capg-201 and the proximal ATS to Capg-208. All the information about start sites actually can be obtained from isofrom expression. What could be the additional value of this scATS?

2. The enrichment analysis in Figure 3 is potentially misleading.
(1) For example, in figure 3. The authors performed GO enrichment analysis based on the host gene of differently utilised TSS, if these TSS is identified based on the same gene, like major or minor tss, then their host gene should be the same, further the GO enrichment should also be the same. If they are performed across all the genes, the GO enrichment was contributed by the differentially expressed gene. Which I think is a logic bug, if I understand the manuscript correctly.
(2) To convincingly define phenotype-specific (like disease-related or cell-type-specific) TSSs, the authors should demonstrate cases where the overall gene expression remains stable while only the TSS usage or quantification significantly changes.

3. For all the figures indicating alternative TSS and gene structure, it would be very helpful to show the transcript structure as well.
(1) In figure 1f, ATS2 after correction appears to align with the start site of annotated transcript Chchd2-201. Could the authors clarify whether these represent the exact same genomic coordinates?
(2) In figure 3g, based on the illustrated exon structure, it appears that the ZFYVE28-210 isoform is not expressed. Could the authors please verify this or clarify the expression level of this specific transcript.
(3) Same for the figure 5a, it’s not clear whether the left TSS refers to a single exon transcript CCR6-L? For figure 5b, in the malignant sample, the read coverage does not appear to match the annotated exon structure of CCR2-L. Specifically, the high-coverage initial peak suggests the presence of an exon contributing to the signal. If the current model were correct, I would expect a relatively flat coverage profile that only increases at the start of the second TSS. Please check whether an additional exon or an alternative transcript structure is responsible for this peak.
(4) Also in figure 1f, figure 2d, the transcript and exon structure are missing, which is a bit confusing.

4. For figure 2b left, since all compared tools are predictive and do not represent a ground truth, the proximity of predictions from other software is not a sufficient validation of accuracy.
5. The manuscript concludes that “In general, distal TSSs exhibit more pronounced degradation than proximal ones”. Have the authors identified any cases where the proximal ATS degradate more than the distal one?

(Remarks on code availability)
The installation from GitHub was unsuccessful. Furthermore, the package is incompatible with my R version 4.5.1. The documentation states that scATS was developed for R 4.0.0, it should maintain compatibility with more recent R releases. I recommend the authors update the dependencies to ensure support for current versions of the R environment.

Reviewer #4

(Remarks to the Author)
I have reviewed rebuttal and thank the authors for addressing all my concerns. From my view each point was carefully addressed and considerable attention to detail provided for the technical aspects. I believe the additional figures, text and information added has improved manuscript.

The only remaining concern I have is that I had issues installing r-scATS software. Details below

Issue 1: Instructions for installation state to run following command which causes an error:

remotes::install_github("LuChenLab/r-scATS/scATS/")

This should be modified to

remotes::install_github("LuChenLab/r-scATS")

Issue 2: After navigating and resolving issue1 I next obtain the following installation error:

Error in loadNamespace(j <- i[[1L]], c(lib.loc, .libPaths()), versionCheck = vI[[j]]) :
there is no package called 'smoother'

Looking into this library I note that the following link states that this library has been "Archived on 2025-12-19 as issues were not corrected despite reminders"

https://cran.r-project.org/web/packages/smoother/index.html

This will be an issue for any person trying to install for the first time.

(Remarks on code availability)
I can confirm github repository is accessible. I have issues installing software as mentioned above. This will need to be resolved as software cannot be installed until library becomes available.

Version 2:

Reviewer comments:

Reviewer #3

(Remarks to the Author)
The authors have addressed most of the concerns from the previous review. The Sashimi plots are now clearer with the exon structures, and the enrichment analysis has been further clarified. Additionally, the quantification of distal and proximal TSS degradation is much more precise compared to the previous general description. Overall, the manuscript is good to me now.

(Remarks on code availability)
The updated online documentation and installation instructions look good to me.

Open Access This Peer Review File is licensed under a Creative Commons Attribution 4.0 International License, which permits use, sharing, adaptation, distribution and reproduction in any medium or format, as long as you give appropriate credit to the original author(s) and the source, provide a link to the Creative Commons license, and indicate if changes were made.
In cases where reviewers are anonymous, credit should be given to 'Anonymous Referee' and the source.
The images or other third party material in this Peer Review File are included in the article's Creative Commons license, unless indicated otherwise in a credit line to the material. If material is not included in the article's Creative Commons license and your intended use is not permitted by statutory regulation or exceeds the permitted use, you will need to obtain permission directly from the copyright holder.
To view a copy of this license, visit https://creativecommons.org/licenses/by/4.0/

Point-by-point response

MS title: Accurate profiling of single-cell alternative transcript start sites by correcting RNA degradation

Dear reviewers:

We thank the reviewers for their valuable comments and suggestions. In response to the comments, we performed additional analysis and experiments, revised the manuscript (the changes are shown in red font) and updated the accompanying figures and tables. We addressed the concerns point-to-point under each comment. And the changes that we introduced into the revised manuscript (MS) are shown in *italics* with page (Pg.) and line (ln.) numbers in the revised MS indicated.

Yours sincerely,

Lu Chen, PhD

Reviewer #1 (Remarks to the Author):

The authors introduce the scATS method to estimate TSS usage in single-cell RNA seq based on a read coverage model that weights alternative TSS clusters by 3' coverage, extending an existing degradation model of exponential 3' coverage decay to explicitly take into account the uniformity of start site products within de-novo TSS clusters. The authors compare predicted TSS selection in single cell blood data to clusters identified by camoTSS and SCAFE. In contrast to these existing approaches, scATS offers a more interpretable locus model, which is trained directly on the data, avoiding the requirement of SCAFE and camoTSS for matched epigenetic data to achieve optimal performance. This provides a significant benefit to users and increases the portability of scATS.

To validate the regularization of TSS discovery in the context of degradation, the authors conducted full length Oxford Nanopore RNA sequencing on matched mHSPC samples as a positive control for non-degraded transcript counts. Finally, the authors conducted a range of functional assays to investigate the signaling implications of lung cancer loci whose relevance was predicted based on embeddings of scATS profiled sequencing data.

Major comments:

1. This manuscript introduces background material highlighting the impact of degradation on the detection of differentially expressed loci and ATS. However, it is unclear to what extent the targets of various functional assays the authors performed were actually substantially degraded. Please clearly state and quantify this phenomenon. In particular, did the LRS components that were interrogated for further study comprise loci whose ATS was undetectable in SCAFE, camoTSS, etc?

Response: We thank reviewer #1 for the helpful suggestions. First, we examined the distribution of RNA degradation across 8,818 LUSC-related TSSs predicted by the LRS model and observed clear degradation effects, with rates ranging from 0 to 9.8 and a mean of 1.1 (**Figure R1.1a**). We further examined the three genes selected for experimental validation and confirmed that all of them are affected by RNA degradation. Specifically, *CCR2-204* exhibits relatively low degradation, whereas *CCR6-203* and *RTKN2-201* show higher degradation rates at the single-cell level (**Figure R1.1b**). Consistent with these observations, several ATS loci were not detected by existing methods. For example, *RTKN2-201* and *RTKN2-202* were not identified by CamoTSS and TSSr, respectively. In the case of *CCR2*, only scATS successfully detected *CCR2-204*, illustrating that RNA degradation can lead to ATS undetectable by other methods while remaining detectable with scATS (**Figure R1.2**). These results demonstrate that degradation substantially impacts ATS identification and quantification, causing some functional TSSs to be undetectable by existing methods.

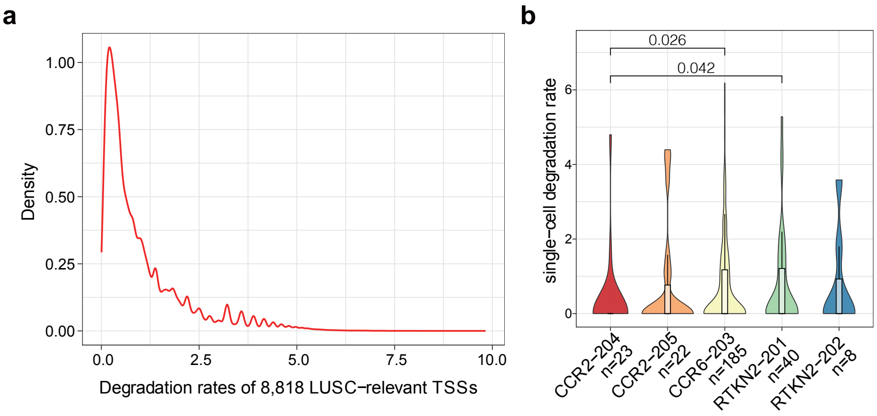

Figure R1.1. Single-cell RNA degradation rates of LUSC-related TSSs in malignant cells.

a, Density distribution of RNA degradation rates across 3,960 malignant cells for 8,818 LUSC-related TSSs predicted by the LRS model.

b, Violin and box plots showing the degradation rates of *RTKN2*, *CCR2* and *CCR6*, with numbers of malignant cells provided in parentheses. The central line represents the median, the box indicates the interquartile range, and the whiskers represent values within 1.5 times the interquartile range; a two-sided Wilcoxon test with BH correction for multiple comparisons.

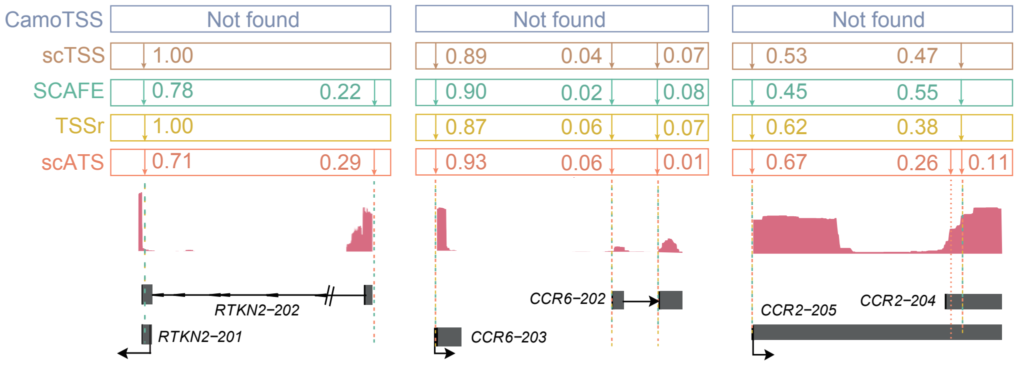

Figure R1.2. Sashimi plots showing the transcription level of *RTKN2* (left), *CCR6* (middle) and *CCR2* (right) in malignant cells from the NSCLC 5' scRNA-seq dataset. Arrows and (upper panels) and the corresponding dashed lines (lower panels) indicate the loci of predicted ATSs inferred by different methods, with PSIs of the corresponding ATS-derived isoform calculated by different methods provided near the peaks. Annotations of isoforms are shown below the sashimi plots.

2. The full length mHSPC sequencing is a powerful control for degradation sensitive TSS quantitation that scATS provides. However, only a couple genes are described here. Please add some quantitative characterization of the global rate of scATS-mediated dropoff in transcripts that don't show up in the scONT dataset, presumably because they don't have a 3' end that can be primed in full length sequencing, in order to expand on this important benchmark.

Response: We acknowledge the reviewer's valuable comment. To benchmark TSS methods, we compared scATS with four other methods, including CamoTSS (Hou et al., 2023), SCAFE (Moody et al., 2022), scTSS (Fu & Li, 2025) and one method developed for bulk RNA-seq, TSSr (Lu et al., 2021). To validate the performance of these methods in the same biological samples, we first conducted long-read sequencing in the mouse bone marrow sample at the single-cell and bulk levels based on both ONT and PacBio platforms. scATS showed the highest validation percentage (65%) by at least one long-read sequencing datasets, which was higher than SCAFE (45%, proportion test, p < 0.02) and TSSr (21%, $p < 3 \times 10^{-9}$; revised **Fig. 2b**). For example, scATS identified two annotated and one novel TSS for *Emg1*, all of which were validated by long-read data, whereas SCAFE, CamoTSS, scTSS and TSSr failed to recover the correct TSS structure. Similarly, scATS was the only method that identified both annotated and novel TSSs for *Idh3g*, while TSSr detected only a single novel TSS and the other methods failed (revised **Fig. 2d**).

We further conducted benchmarking on the mHSPC 5' scRNA-seq dataset. TSSr identified the highest number of loci (39,214), while SCAFE (14,006), scTSS (11,990) and scATS (10,195) identified comparable numbers of loci, and CamoTSS showed the lowest number (n = 1,955) (revised **Fig. 2a**). scATS (90%) has the highest concordance with other methods, followed by SCAFE (87%), CamoTSS (73%) and TSSr (33%). Note that scTSS was completely overlapped due to its reliance on the SCAFE and TSSr intersection and therefore was excluded in this comparison (revised **Fig. 2b**). Next, we assessed the percentage of TSS that can be found in the GENCODE or refTSS database, and scATS showed a significantly higher validation rate (93%), which exceeded SCAFE (78%, proportion test, $p < 0.01$) and TSSr (41%, $p < 6 \times 10^{-13}$; revised **Fig. 2b**).

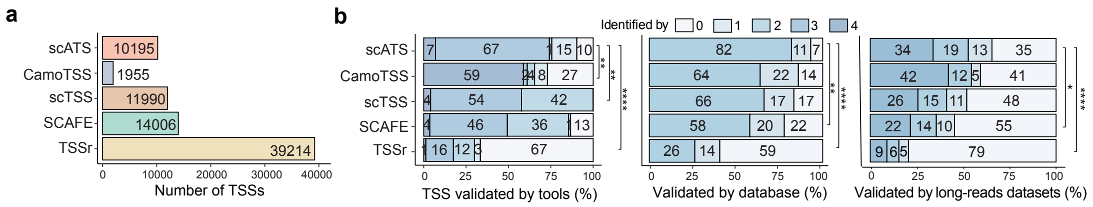

*Revised **Fig. 2a**, Bar plots showing the total number of TSSs identified by scATS, CamoTSS, scTSS, SCAFE, and TSSr.*

***b**, Validation of TSSs identified by different methods using independent evidence with a 50 bp distance threshold to define matched TSSs. Values indicate the number of supporting evidence sources (0, no*

*support). Evidence includes TSS-detection tools (scATS, SCAFE, CamoTSS, TSSr and scTSS; left), annotation databases (GENCODE and refTSS; middle), and long-read datasets (scONT, ONT, and PacBio; right). The percentage of TSSs identified by different methods was assessed using a two-sided proportion test with BH correction; *, p < 0.05; **, p < 0.01; ****, p < 0.0001; comparisons without labels are not significant, unless stated otherwise.*

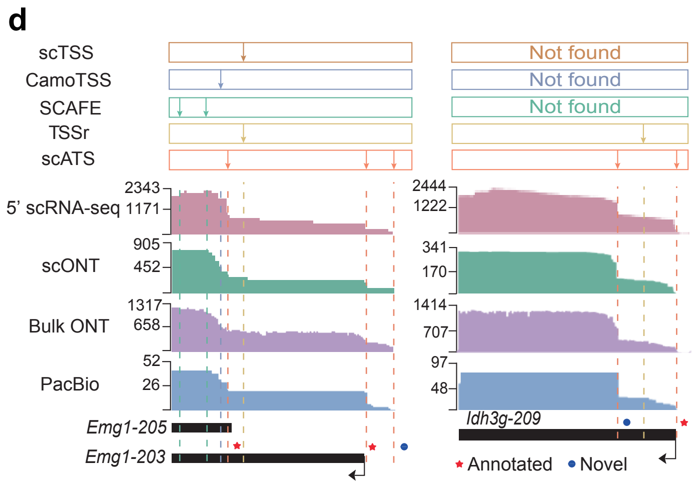

*Revised **Fig. 2d.** Sashimi plots showing the transcription level of Emg1 (left panel) and Idh3g (right panel) in 5' scRNA-seq (pink), scONT (green), ONT (purple) and PacBio (blue) datasets of mHSPC. Arrows (upper panels) and the corresponding dashed lines indicate the loci of predicted ATSs inferred by different methods. Annotation shown below the sashimi plots with annotated and novel TSSs indicated.*

We revised the 'Results' section as follows (Pg. 8, ln. 224-236):

> *"To evaluate the performance of scATS, we applied scATS, along with four existing methods, SCAFE, CamoTSS, TSSr and scTSS, to quantify TSSs during hematopoiesis using the mHSPC 5' scRNA-seq dataset (**Extended Data Fig. 2a**). TSSr, which is developed for bulk RNA-seq, identified most TSSs (n = 39,214). Three single-cell methods SCAFE (n = 14,006), scTSS (n = 11,990) and scATS (n = 10,195; **Supplementary Table 3**), reported comparable numbers of TSSs, and CamoTSS showed the lowest number of TSSs (n = 1,955) (**Fig. 2a**; see **Supplementary Note 4**). Next, we assessed the concordance among these five methods by calculating the percentages of TSSs supported by at least one other method. scATS (90%) has the highest concordance with other methods, followed by SCAFE (87%), CamoTSS (73%) and TSSr (33%). Note that scTSS was completely overlapped due to its reliance on the SCAFE and TSSr intersection and therefore was excluded in this comparison (**Fig. 2b**, left). "*

In Pg. 9-10, ln. 265-278*:*

> *"To validate the accuracy of scATS in TSS identification, we further generated long-read sequencing datasets for mHSPCs, including single-cell Oxford Nanopore Technology (scONT), bulk ONT and PacBio datasets (**Supplementary Table 1**). scATS showed the highest validation percentage (65%) by at least one long-read sequencing datasets, higher than that in SCAFE (45%, proportion test, p = 0.02) and TSSr (21%, p = 3 × 10-9; **Fig. 2b**, right) and other methods. For the Emg1 gene, scATS identified two annotated and one novel TSS in the 5' scRNA-seq dataset, and all of the 3 TSSs, including the novel one was validated by long-read datasets, whereas the other four methods incorrectly identified the TSSs. Similarly, scATS was the only model that identified 2 TSSs in the Idh3g gene, one annotated and one novel TSS, but TSSr detected only a single novel TSS and the other methods failed to recover the full TSS structure (**Fig. 2d**). In sum, scATS provides a balanced framework for accurate TSS identification in 5′ scRNA-seq, with support from annotation concordance, regulatory features and long-read validation."*

3. The mapping efficiency of 10X 5' reads is highly dependent on library construction. Please list detailed run parameters for the NovaSeq 6000 runs including cycle counts

Response: We thank the reviewer for raising this important point. To address this, we have provided the detailed sequencing run parameters for all 5' scRNA-seq datasets used in this study (**Table R1.1**).

Table R1.1. Sequencing run parameters for all 5' scRNA-seq datasets used in this study.

**Datasets**	**Platforms**	**Run parameters**
mHSPC<br>5' scRNA-seq	NovaSeq 6000	● Read 1: 150 cycles (including 16 cycles 10x Barcode and 10 cycles UMI)<br>● i7 index read: 10 cycles<br>● i5 index read: 10 cycles<br>● Read 2: 150 cycles
COVID-19<br>5' scRNA-seq	MGISEQ-2000	● Read 1: 150 cycles (including 16 cycles 10x Barcode and 10 cycles UMI)<br>● i7 index read: 10 cycles

		● i5 index read: 10 cycles<br>● Read 2: 150 cycles
NSCLC<br>5' scRNA-seq	NovaSeq 6000	● Read 1: 150 cycles (including 16 cycles 10x Barcode and 10 cycles UMI)<br>● i7 index read: 8 cycles<br>● i5 index read: 0 cycles<br>● Read 2: 150 cycles

We revised the 'Methods' section, for the mHSPC 5' scRNA-seq (Pg. 34-35, Ln. 1078-1089):

> *"**mHSPC scRNA-seq.** For 5' scRNA-seq, single cells were prepared in the Chromium Single Cell Gene Expression Solution using the Chromium Single Cell 5' Gel Bead, Chip, and Library Kits v2 (10x Genomics) according to Chromium Next GEM Single Cell 5' Reagent Kits v2 (CG000331 Rev E). ... , Libraries were sequenced on the Illumina NovaSeq 6000 platform as 150 bp paired-end dual-indexing (150 cycles R1 with 16 cycles 10x Barcode and 10 cycles UMI, 150 cycles Read 2) at Novogene, Beijing, China."*

For the COVID-19 and NSCLC 5' scRNA-seq (Pg. 33, Ln. 1041-1052):

> *"We also incorporated a 5' scRNA-seq dataset generated from PBMCs of female patients recovered from COVID-19[25], which was prepared following the Chromium Next GEM Single Cell 5' Reagent Kits v2 protocol (CG000331 Rev E). Libraries were sequenced on the BGI MGISEQ-2000 platform as 150 bp paired-end dual-indexing (150 cycles R1 with 16 cycles 10x Barcode and 10 cycles UMI, 150 cycles Read 2). In addition, we analysed a publicly available 10x Genomics 5' scRNA-seq dataset generated from a fresh surgical resection of an NSCLC patient (https://www.10xgenomics.com/datasets/nsclc-tumor-5-gene-expression-1-standard-2-2-0), following the Single Cell V(D)J Reagent Kits protocol (CG000086 Rev C). Libraries were sequenced on the Illumina NovaSeq 6000 platform as 150 bp paired-end single-indexing (150 cycles R1 with 16 cycles 10x Barcode and 10 cycles UMI, 150 cycles Read 2)."*

4. SCAFE requires careful pre-processing, including the selection of BAM reads containing un-annotated G's. The authors thoroughly detail pre-processing of data for camoTSS but omit these details for other methods. Please update the methods detailing how BAMs were pre-processed for SCAFE, in particular, any steps that differ from the preprocessing of data for other benchmarked.

Additionally, similar to the already included camoTSS, details, please provide precise values for SCAFE, including its window size, min tss count, softclip length, etc.

Response: We acknowledge this important point, and have revised the **Supplementary Note 4** to provide detailed descriptions of the usage of SCAFE, TSSr and scTSS for TSS identification and quantification. All three tools were applied using their official workflows and recommended parameters.

5. The authors benchmarked scATS against camoTSS and SCAFE, which both utilize pretrained classifiers (which may also be fine-tuned). As such, their main weakness is the requirement for matched ATAC data, which the authors of the current manuscript do not provide. It is therefore unclear to what extent the newly presented benchmarks simply exploit the many possible training biases of these existing methods, including their vanilla weights derived from human circulating blood cells and fibroblasts, in contrast to scATS, which fits a de-novo model. The authors actually highlight this training background as a source of benchmarking bias, showing that almost 40% of genes of annotated in which annotated TSS are differentially regulated between in mature circulating and bone marrow cells in fig. 2k. A more informative comparison would be against another parametric mixture model which also explicitly enumerates de-novo clusters in the indicator-dependent fashion of scATS. Fortunately, such a model has already been published (scTSS, PLOS Comp BIo 2025). The choice of scTSS is deliberate because the authors have already conducted paired end 5'-scRNA seq, which allows scTSS to utilize the R2 read for more accurate TSS selection, and which is expected to provide some automatic resistance to contamination of the read pool by degraded RNA.

Response: We apologize for the oversight in the ‘Methods’ section. We did use matched ATAC-seq datasets for benchmarking CamoTSS and SCAFE, but this was not clearly stated in the original ‘Methods’. The corresponding ATAC-seq datasets and sample information are now summarised in revised **Supplementary Table 10**. We thank the reviewer for pointing out the potential bias related to pretrained classifiers and training backgrounds, which we agree is an important consideration for fair benchmarking.

We have now included scTSS in all benchmarking analyses (revised **Extended Data Fig. 1c-d**, revised **Fig. 2a-f**, revised **Extended Data Fig. 2b-g**). Revised **Supplementary Note 4** has been updated to describe the TSS identification and quantification procedures used by scTSS.

Revised ***Supplementary Table 10.*** *Epigenomic and chromatin accessibility datasets used in this study.*

Category	Species	Cells/Cell Lines/tissues	Website	Accession ID	Reference	Related Analyses
ATAC-seq	Homo sapiens	cerebellum	ENCODE	ENCFF160VHY	GRCh38	TSS identification on simulated 5' scRNA-seq
ATAC-seq	Homo sapiens	A549	ENCODE	ENCFF399KCR	GRCh38	TSS identification on NSCLC 5' scRNA-seq
ATAC-seq	Mus musculus	HSC	ENCODE	ENCFF351DEI	GRCm38	TSS identification on mHSPC 5' scRNA-seq

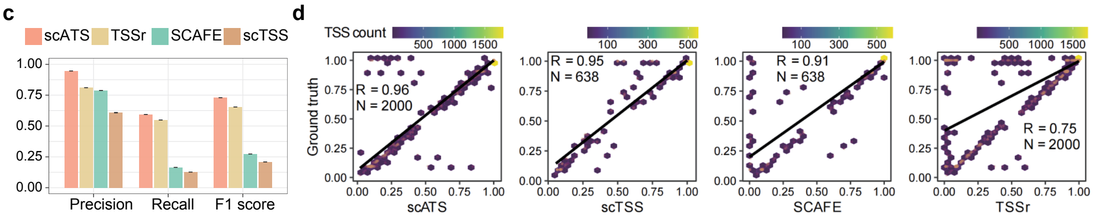

Revised ***Extended Data Fig. 1c,*** *Precision, recall and F1 score for TSS identification across four methods on the simulated 5' scRNA-seq datasets.*

d, *Scatter plots showing the correlation between estimated and true TSS usage for each method on the same simulated TSSs identified in c. Each point represents one TSS. The black solid line indicates the linear regression fit. The color intensity of the dots represents the TSS counts. Pearson correlation coefficients (R) and the number of genes (N) are shown in each panel.*

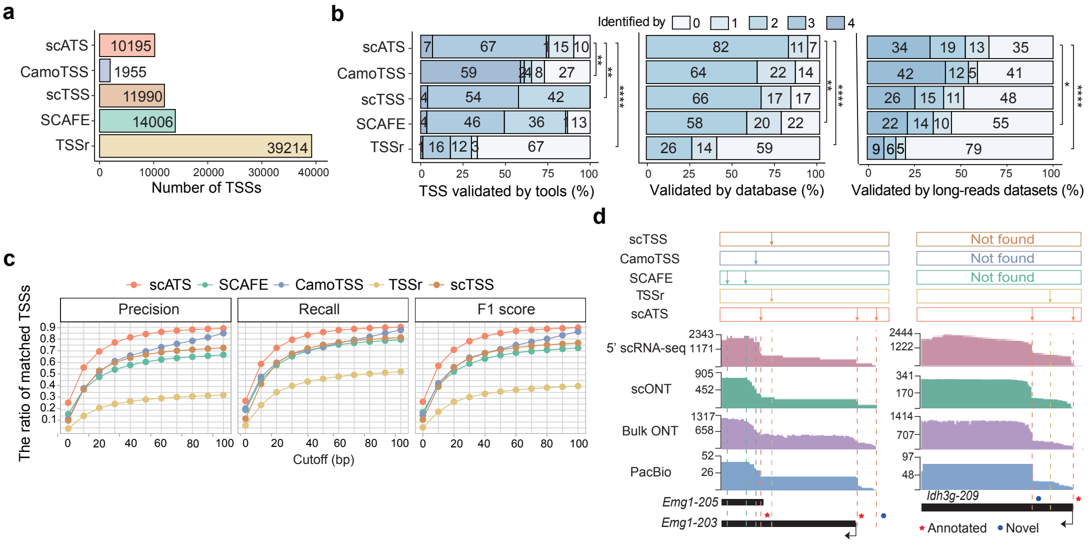

*Revised **Fig. 2a**, Bar plots showing the total number of TSSs identified by scATS, CamoTSS, scTSS, SCAFE, and TSSr.*

***b**, Validation of TSSs identified by different methods using independent evidence with a 50 bp distance threshold to define matched TSSs. Values indicate the number of supporting evidence sources (0, no support). Evidence includes TSS-detection tools (scATS, SCAFE, CamoTSS, TSSr and scTSS; left), annotation databases (GENCODE and refTSS; middle), and long-read datasets (scONT, ONT, and PacBio; right). The percentage of TSSs identified by different methods was assessed using a two-sided proportion test with BH correction; *, p < 0.05; **, p < 0.01; ****, p < 0.0001; comparisons without labels are not significant, unless stated otherwise.*

***c**, TSS detection capability of scATS, SCAFE, CamoTSS, TSSr and scTSS. Precision (left panel), recall (middle panel) and F1 score (right panel) show the ratio of matched TSSs at varying cutoff basepairs (bp) around GENCODE-annotated TSSs.*

***d,** Sashimi plots showing the transcription level of Emg1 (left panel) and Idh3g (right panel) in 5' scRNA-seq (pink), scONT (green), ONT (purple) and PacBio (blue) datasets of mHSPC. Arrows (upper panels) and the corresponding dashed lines indicate the loci of predicted ATSs inferred by different methods. Annotation shown below the sashimi plots with annotated and novel TSSs indicated.*

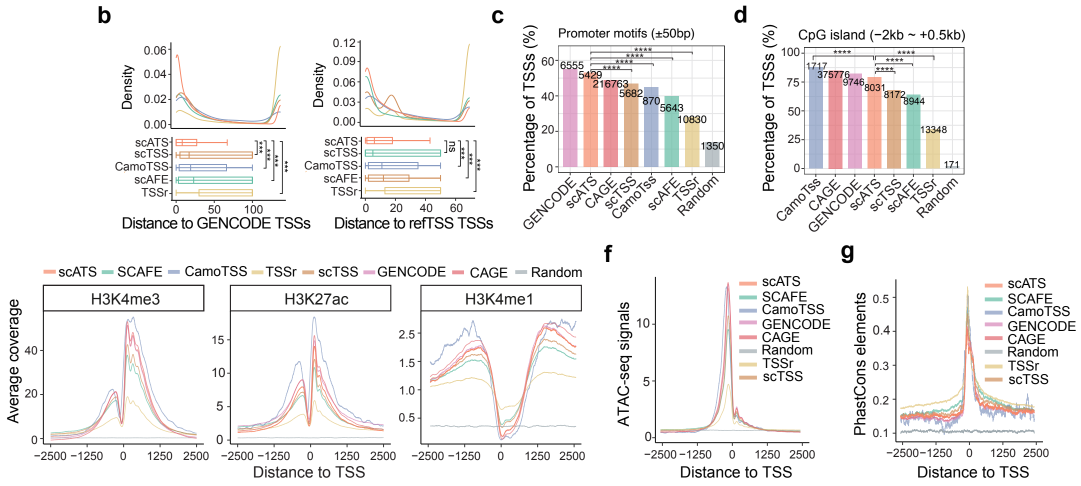

*Revised **Extended Data Fig. 2b**, Density (upper panel) of detected TSSs by scATS (Orange), SCAFE (green) and CamoTSS (blue) and distance (lower panel) to GENCODE (left panel) and refTSS-annotated (right panel) TSSs. Box plot, the central line represents the median, the box indicates the interquartile range, and the whiskers represent values within 1.5 times the interquartile range. The distances of TSSs detected by different methods to annotated TSSs were compared using a two-sided Wilcoxon test with BH correction for multiple comparisons; ***, p < 0.001; ****, p < 0.0001; ns: not significant, unless stated otherwise.*

***c-g**, Distribution of promoter motifs (c), CpG islands (d), histone modifications (e), ATAC-seq signals (f) and PhastCons elements (g) within the indicated distance around the GENCODE-annotated and identified TSSs. Random, genomic positions randomly sampled outside the regions of annotated TSSs. The two-sided proportion tests with BH correction were performed on the percentages of TSSs in pairwise comparisons. The numbers of TSSs are shown above the bars (c) and (d).*

We revised the "Introduction" section (Pg. 3-4, ln. 74-77):

"Despite these well-characterised technical challenges, the existing tools for single-cell TSS inference, including SCAFE[21], CamoTSS[22], scTSS[23] and one method developed for bulk RNA-seq, TSSr[24], lack robust strategies to quantify and correct the systematic biases introduced by RNA degradation."

We revised the 'Methods' section (Pg. 27-28, ln. 853-865) :

*"**Benchmark analyses of TSS identification and quantification***

*We compared scATS with several TSS identification tools, including SCAFE, CamoTSS, TSSr and scTSS (**Supplementary Table 2**). SCAFE (v1.0.0) and CamoTSS (v0.1.7) were run with their recommended workflows using the matched ATAC-seq datasets corresponding to each 5' scRNA-seq dataset (**Supplementary Table 10**). When the output of CamoTSS contained no predicted TSSs, the detection threshold was lowered. TSSr (v0.99.6) was run using the default parameters. scTSS (v0.1.0) relies on an external TSS set to perform TSS cluster quantification and differential TSS usage analysis. Following the recommended workflow, we therefore used the intersection of TSS identification and quantification results from SCAFE (paired-end) and TSSr (R2-based) as the input TSS set for scTSS-based analyses. All command-line parameters, input files and implementation details are provided in **Supplementary Note 4**."*

We revised the "Discussion" section (Pg. 20, ln. 605-609):

"Third, although methods such as SCAFE and CamoTSS can leverage ATAC-seq information to assist TSS inference, matched ATAC–RNA datasets are not routinely available. By contrast, scATS infers TSS usage de novo from RNA reads alone, making it broadly applicable without requiring additional chromatin data."

Reviewer #2 (Remarks to the Author):

I co-reviewed this manuscript with one of the reviewers who provided the listed reports. This is part of the Nature Communications initiative to facilitate training in peer review and to provide appropriate recognition for Early Career Researchers who co-review manuscripts.

Response: We sincerely thank the reviewer and the co-reviewer for their valuable efforts.

Reviewer #3 (Remarks to the Author):

Summary

The manuscript introduces scATS, a computational method designed to correct RNA degradation effects and improve transcription start site (TSS) identification quantification from single-cell RNA-seq data. The method is implemented as an R package and applied to various biological contexts, including lung cancer, COVID-19, and hematopoietic development. In addition, the authors propose a machine-learning–based lung cancer relevance score (LRS) to identify disease-associated TSSs. At the end, the results are supported by several isoform-level experimental validations.

While the study addresses a biologically significant question — accurate detection and quantification of TSSs at single-cell resolution, the manuscript's methodological novelty and resource contribution are limited. Although the transcription start sites topic is of high significance, more systematic benchmark and clarification for the higher performance of scATS and more rigorous presentation are required to support the claimed advances.

Here are the points that I think is helpful to improve the manuscript:

Method

1. The paper describes three core components of scATS: RNA degradation evaluation, TSS identification, and TSS quantification. It remains unclear how degradation correction is implemented and how much improvement it yields compared with other tools addressing degradation (like DeepKINET, https://genomebiology.biomedcentral.com/articles/10.1186/s13059-024-03367-8). It would be helpful to use some of the simulated degradation data which the ground truth is known to evaluate the scATS in correcting degradation.

Response: We agree with these suggestions, and constructed simulations based on the human GRCh38 reference genome and transcriptome (revised **Supplementary Note 3**). We retaining protein-coding transcripts longer than 500 bp and genes with at most two TSSs in non-overlapping genomic intervals. True transcript usage proportions were predefined and used to allocate simulated gene-level expression to transcripts across cells. The total counts for each gene were

generated from a Negative Binomial distribution. In addition, RNA degradation was introduced at the BAM level by resampling R1 start positions relative to the true TSS according to an exponential decay model (Pepke et al., 2009; Wan et al., 2012; Wang et al., 2009). Together, this simulation framework provides a realistic and reproducible benchmark to assess how effectively scATS accommodates RNA degradation effects under known ground-truth conditions.

In addition, we thank the reviewer for suggesting DeepKINET for RNA degradation evaluation (Mizukoshi et al., 2024). DeepKINET is designed to capture splicing and degradation rates in internal exon usage along the transcript body, whereas our analysis focuses on degradation at the 5' end that affects TSSs. Because DeepKINET operates at the gene level and does not model TSS-specific degradation, we restricted the comparison to genes with a single annotated TSS to ensure a fair and comparable benchmark. Under this setting, we performed three independent simulation replicates with known ground-truth degradation rates. scATS accurately recovered the simulated degradation parameters across a wide range of degradation levels at both the single-cell and pseudo-bulk levels (**Figure R3.1**), with a mean correlation of 0.91 between estimated and true degradation rates (**Figure R3.2**). In contrast, DeepKINET showed little sensitivity to increasing degradation severity (mean correlation = 0.20), consistent with its design for modelling internal transcript structure rather than 5' end-specific degradation relevant to TSS inference.

In sum, the simulation-based evaluation confirms that scATS explicitly models and corrects RNA degradation in a manner that is well aligned with the technical characteristics of 5' scRNA-seq, leading to improved robustness of TSS inference under degradation-prone conditions.

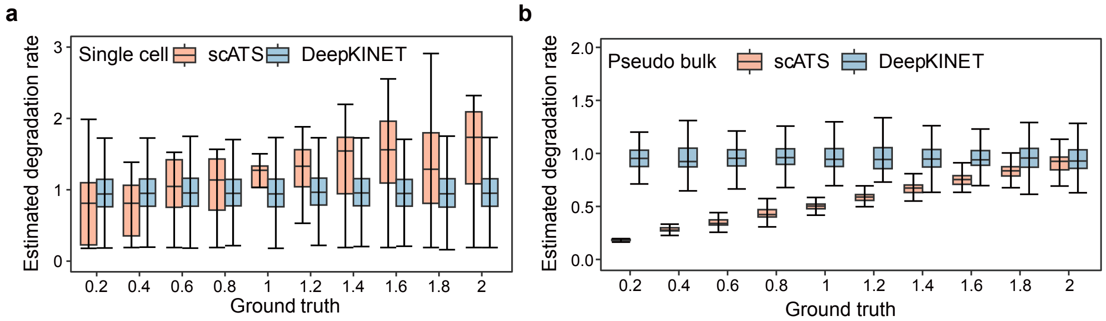

Figure R3.1a-b, Boxplots of estimated RNA degradation rates under different ground-truth degradation levels in the simulated 5' scRNA-seq dataset, shown at the single-cell level (a) and pseudo-bulk level (b). The central line represents the median, the box indicates the interquartile range, and the whiskers represent values within 1.5 times the interquartile range.

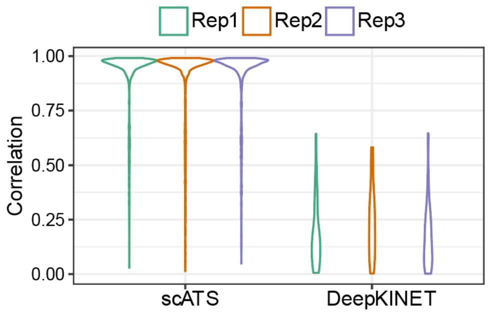

Figure R3.2. Violin plots showing the distribution of correlations between estimated and ground-truth degradation rates across three independent simulation replicates, computed at pseudo-bulk level from the same simulations shown in **Figure R3.1**.

We revised the 'Results' section (Pg. 8, ln. 209-221):

> *"In real 5' scRNA-seq datasets, we observed that more than 99% of estimated α values were below 2, indicating that higher degradation levels rarely occurred or are beyond the capability of scATS to identify a TSS. Therefore, we adopted this empirical range and simulated RNA degradation ranging from 0.2 to 2, with three independent replicates generated for each condition. DeepKINET[29] is a recently proposed method designed to model RNA splicing and degradation effects in scRNA-seq data. We next compared scATS with DeepKINET using these simulated datasets. scATS accurately recovered the simulated α values (**Extended Data Fig. 1e**), with a mean correlation of 0.91 between the estimated and true α values (**Extended Data Fig. 1f**). In contrast, DeepKINET showed limited sensitivity to increasing degradation severity (mean correlation = 0.20), consistent with its design focus on modelling internal transcript structure for inferring kinetic rates, cell clustering, and trajectories rather than 5′ end–specific degradation."*

We revised the 'Methods' section (Pg. 29, ln. 898-908; Pg. 31-33, ln. 985-1035):

> *"**Benchmark analyses of RNA degradation rate***

To benchmark RNA degradation rate estimation, we compared scATS with DeepKINET (v0.2.0), a gene-level kinetic modelling framework. Because DeepKINET operates at the gene level and does not model TSS specific degradation, we restricted the comparison to genes with a single annotated TSS on simulated 5' scRNA-seq datasets, to ensure a fair and interpretable benchmark. Spliced and unspliced read counts were generated using the Python package velocyto (v0.17.17)[89], with the run10x mode and a masked GTF file. The resulting loom files were processed with DeepKINET to estimate splicing and degradation rates at the gene level. For each dataset, cells were filtered and normalised, and the top 2,000 genes were selected for model fitting following the official tutorial."

*"**Preparing simulated datasets with RNA degradation***

To assess the performance of scATS, we simulated data that mimics real 5' scRNA-seq as follows:

1) *Genes were sampled from the reference set based on the reference genome (GRCh38), and only protein-coding positive-strand genes were selected.*
2) *The number of transcripts owned with length ≥ 500 nt from gene set are* $\in \{1,2\}$*, and transcripts with close ATS (200 nt) were excluded.*
3) *Expressed transcripts were sampled from a gene set with expression levels drawn from a normal distribution* $N(0.8, 0.2)$*, and transcript counts per cell were generated using a negative binomial distribution.*
4) *The sequences of R1 and R2 were extracted from gene set mentioned before, with the length 111 nt and 150 nt respectively.*
5) *Start sites of R1 were determined by sampling from a discrete probability distribution, with 90% of the probability assigned to the primary transcription start site and 10% distributed across downstream positions. The end sites of R2 were followed normal distribution* $N(400, 50)$*.*

After generating the simulated paired-end FASTQ files, Cell Ranger (v4.0.0) was applied to generate aligned BAM files, which were used as input for degradation simulation to introduce transcript-specific RNA degradation as follows:

1) *Only read pairs whose R1 reads were mapped to annotated genes were subjected to RNA degradation simulation, whereas all other read pairs were retained unchanged.*

2) *The R1 start position was resampled by drawing a distance from the original TSS according to the exponential decay model defined in formula 2, which was parameterised by a gene- and TSS-specific degradation rate α. In the absence of RNA degradation ($\alpha = 0$), all R1 starts were retained at the original TSS ($d = 0$), whereas increasing values of α shifted probability mass toward larger downstream distances ($d > 0$).*

a) *Without degradation*

In the absence of RNA degradation (α=0), all probability mass was concentrated at the TSS:

$$P(d = 0) = 1,$$

where d represents the distance between the observed R1 start site and the corresponding TSS.

b) *With degradation*

Conditional on the read not starting at the TSS (d > 0), downstream distances follow a truncated exponential distribution:

$$P(d = k \mid d > 0) \propto e^{-\alpha k}, k = 1, \dots, W$$

where W represents the size of the degradation window. As a result, the resulting R1 start-site distance distribution exhibits a peak at the TSS followed by an exponentially decaying tail, reflecting progressive 5′ RNA degradation.

3) *The R2 start position was recalculated based on the original fragment length to prevent the generation of invalid or inconsistent paired-end alignments following R1 repositioning.*

4) *All resampled read pairs were written to a new BAM file, which was subsequently sorted and indexed for downstream analyses.*

Detailed implementation and parameter settings are provided in ***Supplementary Note 3.****"*

2. Scores such as alpha and beta are not clearly explained — which of them actually reflects degradation level?

Response: We apologize for the lack of clarity. We use two distinct but complementary metrics to quantify RNA degradation, alpha (α), which directly reflects the RNA degradation rate, and beta (β), which measures transcript integrity. These two alternative metrics used to evaluate RNA

degradation from different perspectives. As detailed in revised **Supplementary Notes 1** and **2**, the rationale and calculation for each metric are as follows:

Briefly, α is the parameter estimated by the EM model that quantifies the RNA degradation rate from the 5' end toward the 3' end for each TSS and for each sample (**Supplementary Note Fig. 1**). Thus, larger α values correspond to stronger 5' degradation, making α a direct and quantitative measure of degradation severity at the TSS and sample level in our analyses.

Second, the β score is a normalised area under the ECDF of R1 start sites for a given TSS, yielding a value between 0 and 1. It primarily captures the integrity of the 5' signal of each transcript (**Supplementary Note Fig. 2**). When the 5' end of a transcript is degraded, reads are lost from the 5' start site and shift downstream, leading to a slower rise in the cumulative distribution and thus a lower β value. Consequently, β is inversely related to the degradation level and serves as a complementary, integrity-based score.

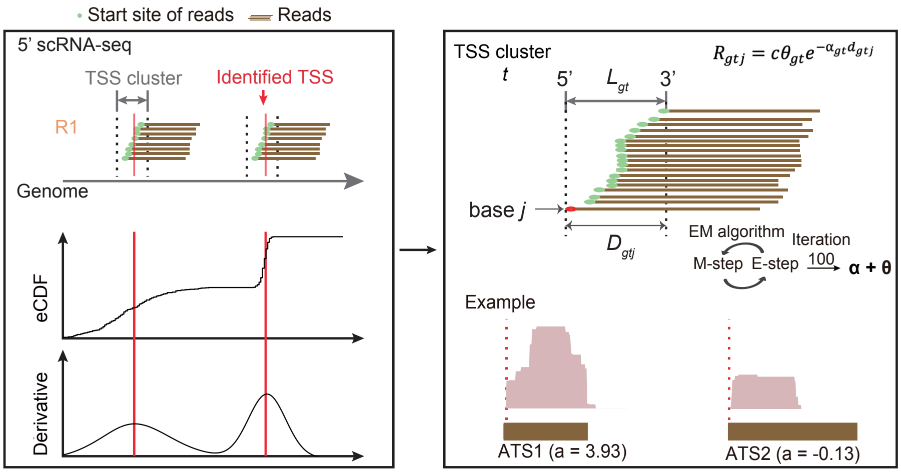

Supplementary Note Fig. 1. *Overview of the scATS computational framework for joint estimation of the degradation level (α) and degradation-corrected percent spliced-in (PSI, θ).*

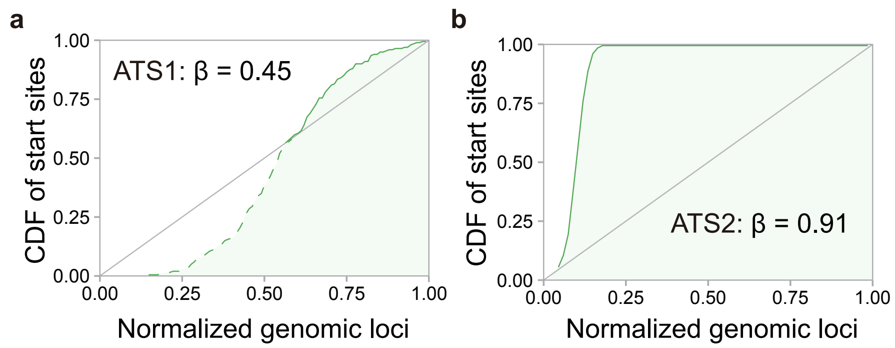

Supplementary Note Fig. 2. Relationship between RNA degradation, read distribution, and the β metric.

a-b*, Schematic representation of read start distributions and their corresponding ECDFs for transcripts with low (a) and high (b) levels of 5′ RNA degradation, illustrating how the β value quantifies transcript integrity.*

We revised the 'Results' section (Pg. 6, ln. 144-147):

> *"To correct the 3' positional bias caused by RNA degradation, we developed two complementary metrics: α, representing the RNA degradation rate, and β, reflecting the transcript integrity. Both metrics can be applied at the TSS and sample levels."*

3. The identification and quantification of TSSs should be evaluated independently, using appropriate benchmarks. The methods included in the benchmark are not systemic, some of existing methods are missing (TSSr, TSS-Captur. etc).

Response: We appreciate this important suggestion from the reviewer. We evaluated the TSS identification and quantification independently using appropriate benchmarks. We therefore performed simulations of 5' scRNA-seq data with 2,000 genes across 3,000 cells (revised **Supplementary Note 4**). TSSr was originally developed for bulk RNA-seq and CAGE-like data that rely on R2-only information (Lu et al., 2021), and therefore does not fully exploit the read structure of paired-end 5' scRNA-seq. Nevertheless, we included TSSr to provide a broader comparison.

For identification, scATS and TSSr detected TSSs from all simulated genes, whereas SCAFE and scTSS detected TSSs from 638 genes. scATS showed the highest precision, recall and F1 score among all methods (revised **Extended Data Fig. 1c**). For quantification, both scATS and scTSS showed strong agreement with the ground truth, achieving Pearson correlation coefficients of 0.96

and 0.95, respectively. In contrast, TSSr showed lower concordance with a correlation coefficient of 0.75 (revised **Extended Data Fig. 1d**). We incorporated TSSr into all relevant TSS identification and quantification benchmarks on real datasets (revised **Fig. 2a-d**, revised **Extended Data Fig. 2b-g**). These results indicate scATS provides more accurate TSS identification under realistic 5' scRNA-seq noise and degradation settings.

We did not include TSS-Captur in the benchmarking because it was developed specifically for prokaryotic transcriptomes and relies on bacterial promoter architecture and operon structures that do not apply to eukaryotic 5' scRNA-seq data (Paz et al., 2024). For this reason, we considered it unsuitable as a benchmark in the current context.

Together, these additions clarify the independent evaluation of TSS identification and quantification, broaden the benchmarking landscape, and further support the robustness of scATS for degradation-aware TSS analysis in 5' scRNA-seq data.

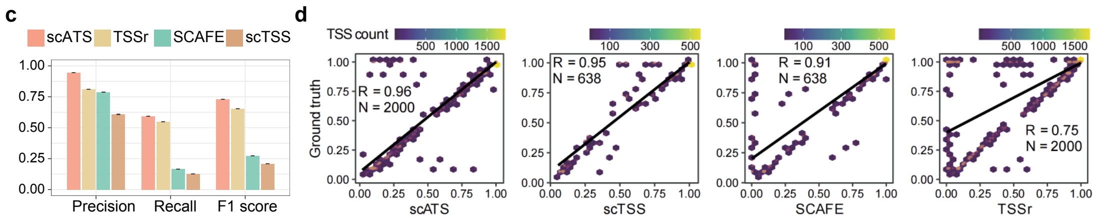

Revised ***Extended Data Fig. 1c,*** *Precision, recall and F1 score for TSS identification across four methods on the simulated 5' scRNA-seq datasets.*
d, *Scatter plots showing the correlation between estimated and true TSS usage for each method on the same simulated TSSs identified in c. Each point represents one TSS. The black solid line indicates the linear regression fit. The color intensity of the dots represents the TSS counts. Pearson correlation coefficients (R) and the number of genes (N) are shown in each panel.*

We revised the 'Results' section on Pg. 7, ln. 190-207:

> "***Performance of scATS on simulated datasets***
> *To evaluate the robustness of scATS to RNA degradation, we simulated a 5' scRNA-seq dataset consisting of 3,000 cells and 2,000 genes based on the GRCh38 reference genome, using a Negative Binomial distribution to account for biological variability and technical noise, as described in a previous study[28.] Raw reads were first aligned to generate BAM files, after which RNA degradation was simulated by applying an exponential decay function to R1 start sites (see* ***Methods*** *and* ***Supplementary Note 3****).*

*We first benchmarked the performance of different methods (**Supplementary Table 2**) using simulated data with mild RNA degradation (α = 0.2). For TSS identification, scATS and TSSr detected TSSs from all simulated genes, whereas SCAFE and scTSS detected TSSs from 638 genes. CamoTSS was excluded from this comparison, whose design focuses on TSS cluster–level analysis and therefore detected no TSSs under this simulation setting (see **Supplementary Note 4**). scATS showed the highest precision, recall and F1 score among all methods in TSS identification (**Extended Data Fig. 1c**), and also achieved the highest correlation with the ground truth (R = 0.96, Pearson correlation, **Extended Data Fig. 1d**).”*

4. Some important data, like CAGE-seq data which is considered a gold standard for TSS validation is missing.

Response: We thank the reviewer for this valuable suggestion. We have added CAGE-seq and ATAC-seq datasets (revised **Supplementary Table 10**) for TSS identification analysis and updated the results in revised **Extended Data Fig. 2c-g**. We found that 85% (375,776) of CAGE-seq TSSs contained core promoter motifs (revised **Extended Data Fig. 2c)**. Moreover, 49% (216,763) of CAGE-seq TSSs, co-localised with CpG islands as shown in revised **Extended Data Fig. 2d**. Furthermore, we observed a characteristic depletion of promoter-associated markers, specifically H3K4me3 and H3K27ac, in the region spanning approximately -300 to +50 bp around the TSSs. In contrast, the enhancer-associated marker H3K4me1 was predominantly enriched in downstream genomic regions (revised **Extended Data Fig. 2e**). These profiles matched the patterns of annotated TSSs and were not observed at randomly selected background loci. In addition, the CAGE-seq TSSs showed a high degree of evolutionary conservation and chromatin accessibility as supported by ATAC-seq peaks (revised **Extended Data Fig. 2f**) and PhastCons conservation score (revised **Extended Data Fig. 2g**). Additionally, the short and long isoforms of *RTKN2*, which is one of the genes further validated in downstream experiments, are also supported by the CAGE-seq data (revised **Extended Data Fig. 7a**).

*Revised **Supplementary Table 10.** Epigenomic and chromatin accessibility datasets used in this study.*

Category	Species	Cells/Cell Lines/tissues	Website	Accession ID	Reference	Related Analyses
CAGE-seq	Homo sapiens	Bronchial Epithelial Cell	FANTOM5	11511-119G8	GRCh38	TSS identification on NSCLC 5’ scRNA-seq

CAGE-seq	Homo sapiens	Alveolar Epithelial Cells	FANTOM5	11510-119G7	GRCh38	TSS identification on NSCLC 5' scRNA-seq
CAGE-seq	Mus musculus	stem cell (cKit+ Sca1- lineage-)	FANTOM5	12121-128C6	GRCm38	TSS identification on mHSPC 5' scRNA-seq
ATAC-seq	Homo sapiens	cerebellum	ENCODE	ENCFF160VHY	GRCh38	TSS identification on simulated 5' scRNA-seq
ATAC-seq	Homo sapiens	A549	ENCODE	ENCFF399KCR	GRCh38	TSS identification on NSCLC 5' scRNA-seq
ATAC-seq	Mus musculus	HSC	ENCODE	ENCFF351DEI	GRCm38	TSS identification on mHSPC 5' scRNA-seq

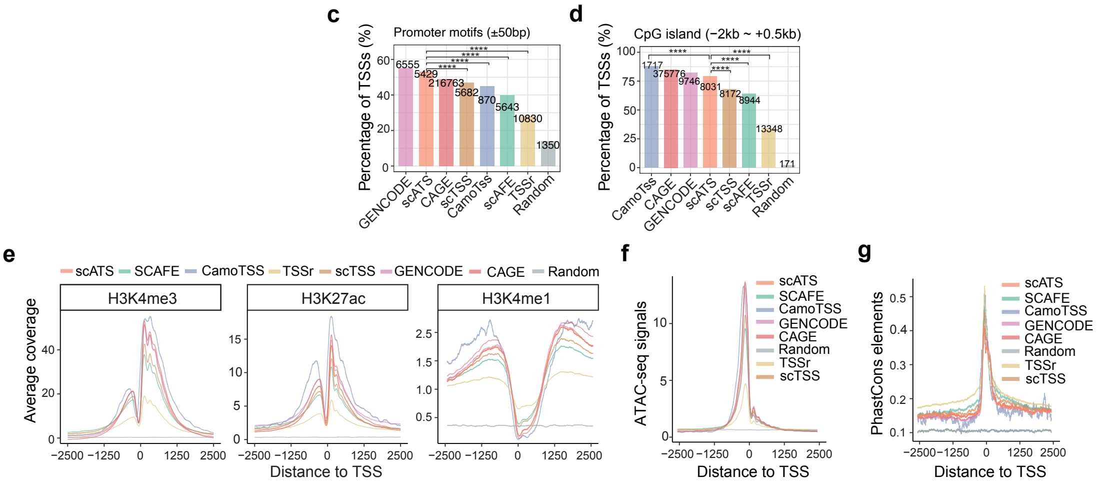

*Revised **Extended Data Fig. 2c-g**, Distribution of promoter motifs (c), CpG islands (d), histone modifications (e), ATAC-seq signals (f) and PhastCons elements (g) within the indicated distance around the GENCODE-annotated and identified TSSs. Random, genomic positions randomly sampled outside the regions of annotated TSSs. The two-sided proportion tests with BH correction were performed on the percentages of TSSs in pairwise comparisons. The numbers of TSSs are shown above the bars (c) and (d).*

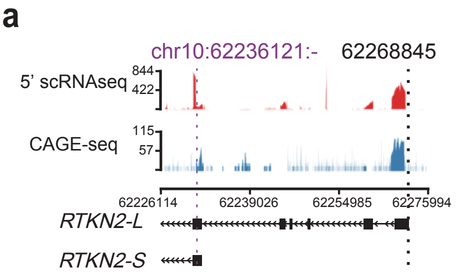

*Revised **Extended Data Fig. 7a,** Sashimi plots showing the transcription level of RTKN2 in 5' scRNA-seq (red) and CAGE-seq (blue) datasets of NSCLC patients. The corresponding dashed lines indicate the loci of predicted ATSs inferred by scATS. The TSS with higher expression in the malignant cells are highlighted in purple. Annotation shown below the sashimi plots.*

5. The confidence or thresholding strategy for defining TSSs is not described. Does scATS provide adjustable parameters to control detection stringency?

Response: We thank the reviewer for raising this important point. scATS provides adjustable parameters that allow users to control the stringency of TSS detection, which are described in revised **Supplementary Note 8**. Briefly, we evaluated three main parameters on the mHSPC 5' scRNA-seq dataset:

1. 'MinGeneReads' defines the minimum number of reads required for a gene to be considered for TSS inference. Increasing this value filters out lowly expressed genes, leading to a gradual decrease in the number of detected TSSs and genes, while increasing the proportion of ATSs among the remaining genes (revised **Supplementary Note Fig. 8b**).

2. The 'window' parameter controls the Gaussian smoothing window size and defines the local flanking region used to extend candidate TSS boundaries. Larger window sizes produce smoother signal profiles with lower resolution. In our analysis, increasing the window size markedly reduced the number of detected TSSs, especially ATSs, and the proportion of ATSs dropped to zero when the window exceeded 20 bp (revised **Supplementary Note Fig. 8c**).

3. The "greedy" parameter determines whether the most distal annotated TSS is reported even if it does not meet the specified criteria, providing additional flexibility for exploratory analyses. When this parameter is set to TRUE, more TSSs and genes are retained (revised **Supplementary Note Fig. 8d**).

Together, the effects of these parameters can vary across datasets, and we hope that our evaluation provides practical guidance for users when choosing appropriate settings.

Second, to address confidence and thresholding in TSS definition, we performed additional simulations to examine how gene-level read depth affects the accuracy of TSS detection (revised **Supplementary Note 4**). Using our simulated data, we found that when the number of reads per gene was limited to 200, the correlation between the estimated alpha value and the ground truth was 0.61. Increasing the sequence depth can substantially improve the correlation, reaching 0.75 and 0.89 when 300 and 500 reads are available, respectively (revised **Supplementary Note Fig.**

8a). Because gene-level read depth directly corresponds to the 'MinGeneReads' parameter in scATS, this parameter can be used to control detection stringency.

Finally, we provide a detailed explanation of all parameters in the 'TSSCDF' function, which is used for TSS identification and quantification, along with their recommended usage in **Table R3.1**, allowing users to adjust detection stringency according to their specific datasets. In addition, we have added **Supplementary Note 8** to describe how users can pre-examine gene-level coverage in their own data to guide the choice of the 'MinGeneReads' parameter, and to evaluate the impact of sequencing depth on TSS detection.

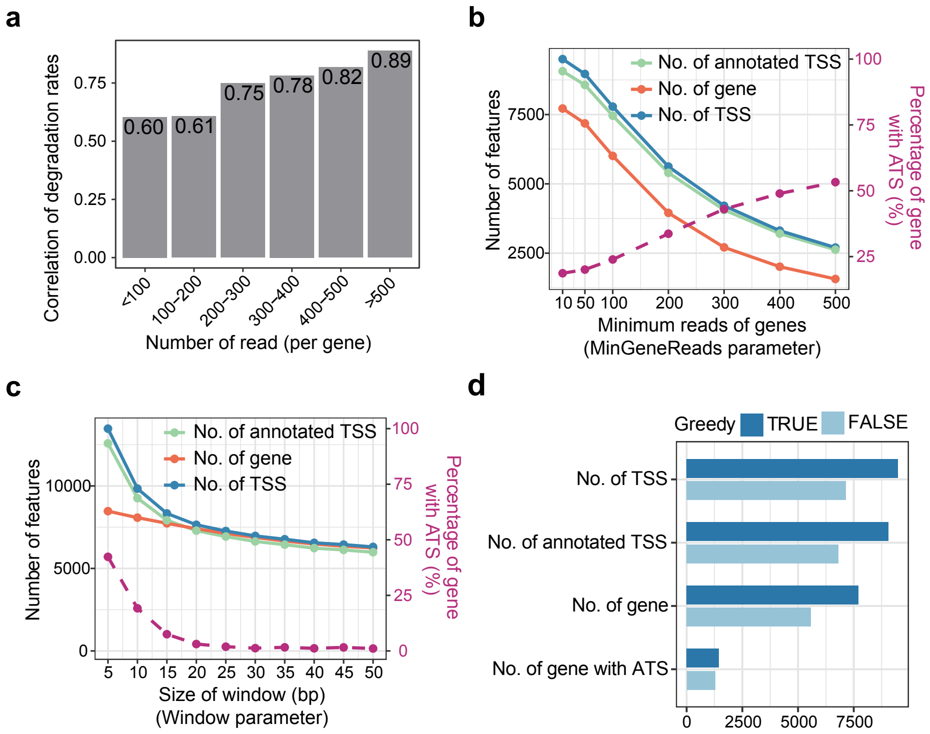

*Revised **Supplementary Note** **Fig. 8a**, Bar plots showing the correlation between estimated alpha values and ground truth across different gene-level read depth groups in the simulated 5' scRNA-seq dataset. Pearson correlation coefficients are shown in each bar.*

***b-c**, Pseudo-bulk analyses of TSS detection across 'MinGeneReads' (b) and 'window' (c) parameters in the mHSPCs 5' scRNA-seq datasets. Line charts show the numbers of detected TSSs, annotated TSSs and host genes (left Y-axis), and red line charts show the percentage of genes with ATSs (right Y-axis). Sequencing depth is indicated on the X-axis. Predicted TSSs located within ±50 bp of CAGE-seq peaks were considered annotated, while others were classified as novel.*

***d**, Bar plots showing the numbers of TSSs and genes with the 'greedy' parameter set to TRUE or FALSE.*

Table R3.1. Parameters used in the 'TSSCDF' function of scATS, as updated in the GitHub repository.

Arguments	Description
object	A Seurat object.
bam	Path of bam file(s).
genes	Genes used for ATS inference and quantification. By default, all genes in the Seurat object are included.
gtfFile	Path of GTF file or Granges file of GTF file.
txdb	A TxDb object.
UTROnly	Only infer ATS within annotated 5' UTR regions or first exons of transcripts without 5' UTR (default=True).
scDR	Whether to estimate the single-cell level degradation rate of TSS. Setting TRUE triggers an additional inference step and vastly increases runtime (default=False).
MinGeneReads	The minimum reads of a gene for TSS inference (default=500).
MinscTSSReads	Minimum reads of single-cell TSS reads for single-cell degradation rate estimation when scDR = TRUE (default=5).
min.TSS.percent	Defines the minimum proportion of total supporting reads (i.e., the change in cumulative density, F1 – F0) that a candidate TSS region must contribute. This parameter filters out weak or noise-driven TSS peaks (default=5%).
min.local.percent	Based on the maximum local derivative change, this parameter evaluates whether a candidate TSS region exhibits sufficiently sharp local enrichment. It is used to remove locally insignificant or low-confidence peaks (default=1%).
mapqFilter, isSecondaryAlignment, isSupplementaryAlignment, isDuplicate	parameters for ScanBamParam to read the BAM file(s).
p.cutoff	A significance threshold used to determine whether the smoothed derivative exceeds the background derivative distribution, where the cutoff is computed from a fitted normal model. Smaller values of p.cutoff impose stricter criteria and retain only high-confidence TSS candidates (default=0.01).
window	Controls the Gaussian smoothing window and is also used to define local flanking regions and extend candidate TSS boundaries. Larger windows produce smoother trends with reduced resolution, while smaller windows increase sensitivity at the cost of higher noise (default=10).
greedy	greedy for the first annotated TSS? If this parameter is set to TRUE, the most distal annotated TSS will be printed even if it does not satisfy the specified condition (default=True).
cores	The number of cores for parallel working (default=1).
verbose	A logical control if a text progress bar is displayed (default=False).
project.name	The name of the project (default=NULL).

6. In Figure 1c, the CamoTSS line appears flat. Is there any cut-off for CamoToss?

Response: We apologize for the mistake. The curve for CamoTSS in **Fig. 1c** is part of a schematic illustration rather than a plot presenting actual benchmark results. It was based on an older version of our simulation workflow and no longer reflects the analyses included in the submitted MS. We have updated this schematic figure in revised **Fig. 1c**, and the full set of revised simulation results has been provided in revised **Extended Data Fig. 1c-f**.

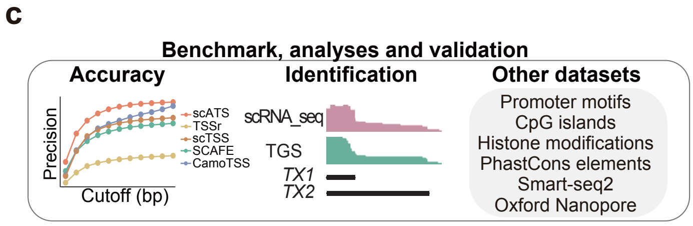

*Revised **Fig. 1c**, The mHSPC and COVID-19 datasets were used to assess the accuracy of scATS, cross-validated with datasets of different aspects.*

7. In Figure 1e, the statement “5′ scRNA-seq datasets exhibit highly variable evenness and 3′ bias” is not visually supported by the plot. The three curves look nearly identical, and the zoomed-in view seems inconsistent with the top panel.

Response: We thank the reviewer. The apparent inconsistency in the original figure was caused by the visualisation strategy rather than the underlying analysis. In the initial version, read coverage was computed using RSeQC package separately for full transcript bodies and for first-exon regions, which are based on different coordinate systems and are not directly comparable. Zooming in on the first-exon profile therefore led to a misleading impression that the curves were similar.

To more clearly illustrate RNA degradation–associated 3′ bias, we have revised the figure by zooming in on the first 10% of the transcript body, which allows a direct comparison within the same coordinate system. The figure has been updated accordingly.

e

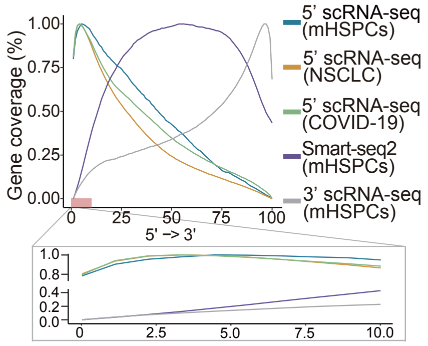

*Revised **Fig. 1e**, Visualisation of the overall read coverage from 5' to 3' of the gene body in different datasets. The read coverage of the first 10% of the gene is shown in the lower panel.*

8. The LRS model appears disease-specific. It is unclear whether it can generalize to other cancer types or disease conditions.

Response: We thank the reviewer for raising this important point. We added another cancer type, glioblastoma (GBM), in **Supplementary Note 7** to demonstrate that this model can generalise to other cancer types or disease conditions. We note that a comprehensive pan-cancer analysis of functional TSSs extends beyond the scope of the current study. This will be the focus of our subsequent work, which we aim to report in the near future.

The disease conditions should meet the following two conditions: (1) sufficient positive TSSs associated with cancer or disease conditions are available for model training; (2) high-quality 5' scRNA-seq datasets.

Briefly, we applied scATS to another publicly available 5' scRNA-seq dataset generated from tumour specimens of a GBM patient (Zhao et al., 2024), and identified 53,037 TSSs across 16,125 genes in 15,088 cells. Based on 81 positive TSSs from a previous study (Demircioğlu et al., 2019), we evaluated different models, including RF, LR, MLP and SVM (revised **Supplementary Note Fig. 7a-d**), and found that the RF model also showed the best performance (AUROC = 0.99, AUPRC = 0.99). We then used the RF model as the final LRS model for downstream analysis and identified 3,025 GBM-associated TSSs (revised **Supplementary Note Fig. 7e**). Disease ontology (DO) analysis revealed enrichment in the "axonal neuropathy", which has been reported as a targetable

driver of glioblastoma progression through neuron–glioma interactions (Clements et al., 2025). For example, tumor necrosis factor receptor superfamily member 1A (*TNFRSF1A*), enriched in this DO term, has been associated with patient prognosis in GBM (Han et al., 2022). GO analysis highlighted “cerebral cortex development”, a pathway linked to glioma aggressiveness via remodelling functional neural circuits (Hua et al., 2022). Within this pathway, *TACC3* is frequently amplified and overexpressed in GBM and has been reported to promote tumor progression (Duncan et al., 2010) (revised **Supplementary Note Fig. 7f**). Together, these results demonstrate the ability of LRS to identify biologically relevant TSSs in brain cancer.

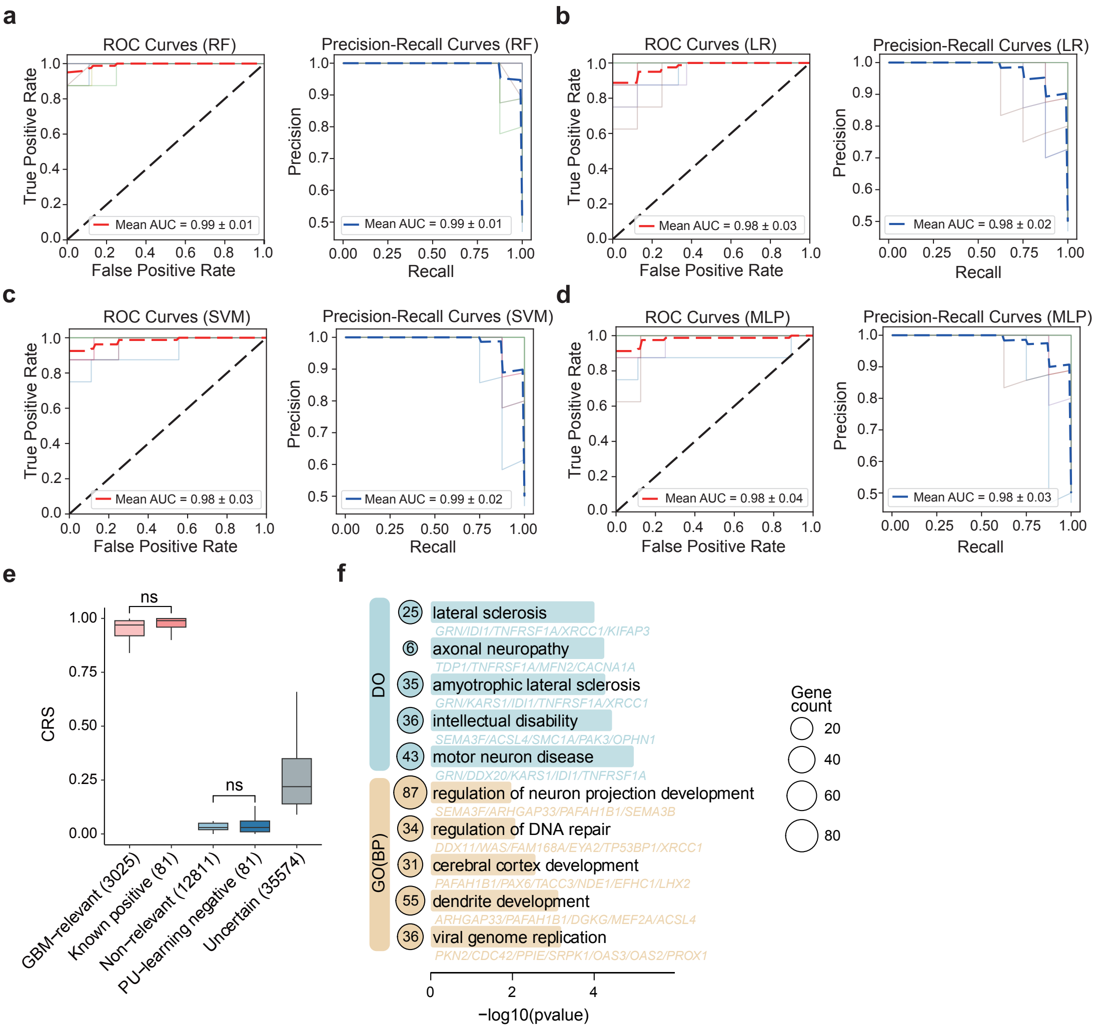

Revised ***Supplementary Note Fig. 7. Single-cell transcriptomic analyses of human GBM and performance of LRS.***

a-d*. Performance of four models: RF (a), LR (b), SVM (c) and MLP (d) assessed by AUROC (left panels), and AUPRC (right panels). The red and blue dashed lines represent the overall AUROC and AUPRC from 10-fold cross-validation, respectively. The mean AUCs are shown.*

e, *Box plots showing the distributions of LRS probabilities for GBM-relevant, known positive, non-relevant, PU-learning negative and uncertain TSSs. Number of TSSs in each category shown in parentheses; ns: not significant; a two-sided t-test with BH correction for multiple comparisons.*
f, *Disease ontology (top panel) and GO enrichment (bottom panel) analyses of the host genes with GBM-relevant TSSs. Numbers to the left of each term indicate the counts of associated genes, and the contributing host genes are partially listed below. The bars indicate the -log10 (p value).*

We have updated the 'Discussion' section (Pg. 21, ln. 648-655):

> *"The LRS model is constrained by (1) sufficient positive TSSs associated with cancer or disease conditions for model training; and (2) high-quality 5' scRNA-seq datasets. Curation with larger datasets of functionally validated ATSs across cancer subtypes would improve its accuracy and application in other cancer types. In this study, we further applied the same modelling strategy to glioblastoma datasets and observed good performance (see* ***Supplementary Note 7****), supporting the general applicability of the LRS approach."*

Data analysis

1.The data provided in this study is not systematic. If it's just used as an application example, then the method is not strong enough and the application is too much. If it's provided as "accurate systemic profiling of single-cell alternative transcript start sites" as title then, it's maybe not systematic/representitive.

Response: We thank the reviewer for this constructive comment and agree with the concern. We have revised the title to delete the word "systematic". In addition, to improve the strength of the method and the biological representativeness of our study, we have added several new analyses to address this concern and to further demonstrate the applicability of our approach:

1. Adding additional public datasets from CAGE-seq, ATAC-seq revised (**Extended Data Fig. 2c-g**), and long-read sequencing based on both ONT and PacBio platforms (revised **Fig. 2b, d**).
2. Expanding the benchmark analyses, incorporating simulation (revised **Extended Data Fig. 1c-f**) and expanded comparisons with the existing four methods to evaluate performance comprehensively (revised **Fig. 2a-d;** revised **Extended Data Fig. 2b-g**).

3. Integrating additional GBM 5' scRNA-seq datasets for the LRS model, which improves the breadth and representativeness of the biological applications (revised **Supplementary Note 7**).
4. Including an analysis of the impact of sequencing depth on TSS identification (revised **Supplementary Note 8**) and degradation-rate estimation (revised **Extended Data Fig. 1e-f**), showing that the core estimates remain robust across a wide range of depths.
5. Adding more detailed methodological documentation on GitHub, including clearer explanations of scATS workflow, thresholding criteria, and parameter behaviours.

2.The analysis sections (e.g., pathway enrichment) are superficial and unsystematic. More comprehensive functional analysis related to the start site would strengthen the biological interpretation.

Response: We appreciate the reviewer's insightful suggestion. We have added the SCENIC analyses in mHSPCs, COVID-19 and NSCLC 5' scRNA-seq. In the hematopoietic system, genes with differential TSSs were linked to distinct regulons. Foe example, the Spi1 regulon, which regulates hematopoietic lineage commitment (Loughran et al., 2020), was associated with mature lost TSSs in HSPCs, whereas the Stat3 regulon, involved in T cell differentiation (Sun et al., 2023), was linked to mature gained TSSs in mature cells (revised **Extended Data Fig. 3k-m**), reflecting stage-specific regulatory programs. In naïve CD8 T cells from COVID-19 patients, differential TSSs were enriched in the TCF7 regulon (revised **Extended Data Fig. 4d-e**), which is known to regulate T cell self-renewal and antiviral responses (Tu et al., 2024), indicating a functional connection between TSS usage and immune activation. In lung cancer, differential TSSs were organised into regulon modules driven by TFs such as STAT1 and ELF1 (revised **Extended Data Fig. 5c-e**), which are associated with immune infiltration and tumor invasiveness (Kamal & Mohager, 2025; Yang et al., 2024), and these regulons preferentially targeted malignant dominant TSS host genes. These results demonstrate that changes in TSS usage are linked to specific TFs and biological functions. We have revised the 'Methods' and 'Results' sections as follows:

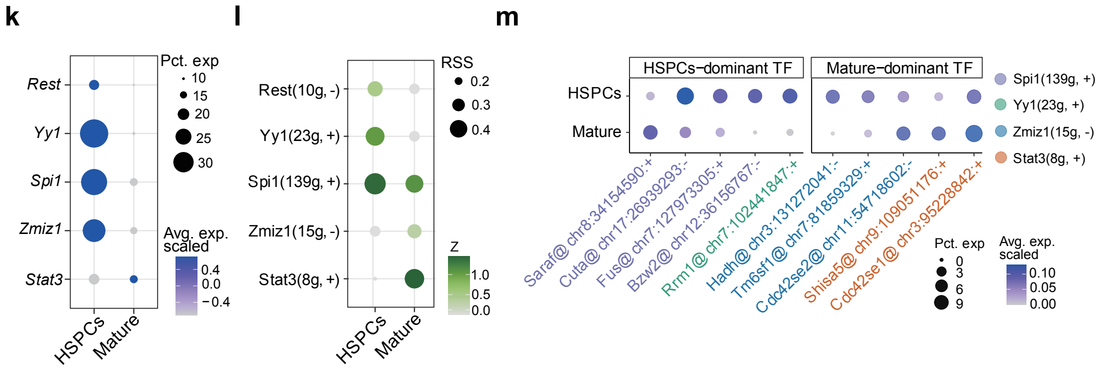

Revised ***Extended Data Fig. 3k-l****, Dot plots showing gene expression (k) and activity level (l) of transcription factors (TFs). The dot size represents the percentage of cells expressing each gene and the regulon specificity score (RSS) of TFs, and the color intensity indicates the scaled average expression level and the Z-score in panels p and q, respectively. TF labels indicate the number of targeted genes (g) and the direction of regulation (+ or −).*

m*, Dot plot showing the expression level of mature-gained or lost TSSs. Dot size represents the percentage of cells expressing each gene, while color intensity indicates the scaled average expression level, with different colors indicating distinct TFs.*

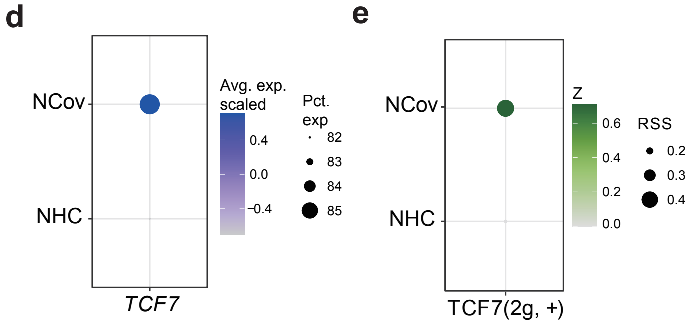

Revised ***Extended Data Fig. 4d-e****, Dot plots showing gene expression (d) and activity level (e) of TCF7 TF. The dot size represents the percentage of cells expressing TCF7 gene and the RSS values of TCF7 TF, and the color intensity indicates the scaled average expression level and the Z-score in panels c and d, respectively. TF label indicate the number of targeted genes (g) and the direction of regulation (+ or −).*

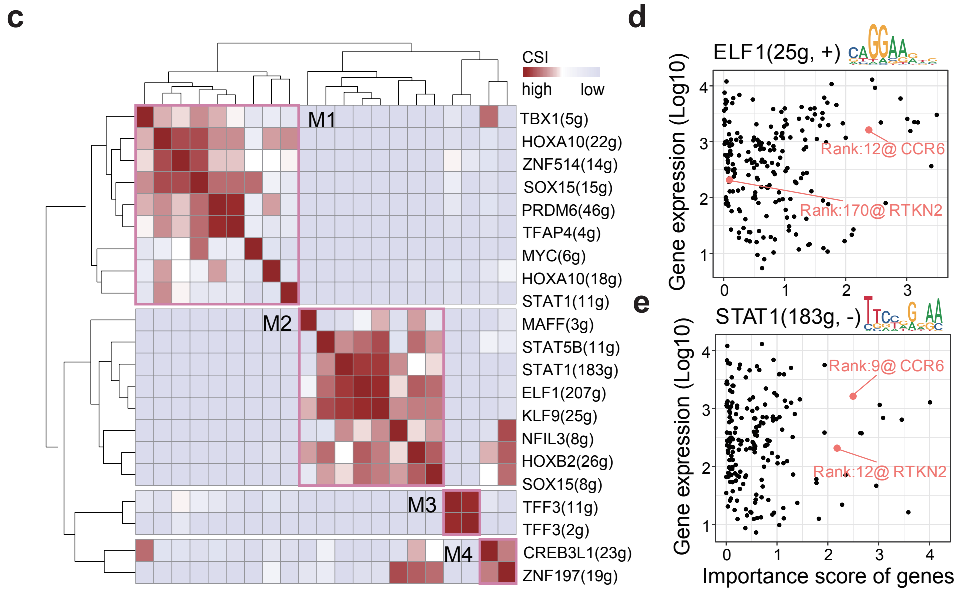

*Revised **Extended Data Fig. 5c**, Modules (M) based on regulon connection specificity index (CSI) matrix, along with corresponding TF regulons.*
***d-e**, Dot plot showing target genes of the ELF1 (d) and STAT1 (e) regulons. X-axis indicates the importance scores of target genes inferred by SCENIC, and the Y-axis represents the expression levels of the target genes, with the number of targeted genes (g) and the direction of regulation, positive (+) or negative (−) were shown. The importance ranking results of CCR6 and RTKN2 from TF regulons are highlighted in red text. The sequence logos were generated from FIMO-identified motif occurrences within the CCR6 and RTKN2.*

We updated the 'Results' section for the mHSPCs 5' scRNA-seq dataset (Pg. 11, ln. 322-331):

> *"To reconstruct gene regulatory networks (GRN) affected by TSS, we analysed transcription factor (TF) activity using SCENIC[40]. This analysis identified five differential regulons from 504 host genes carrying gain or loss TSSs (**Extended Data Fig. 3k-l**). Among these, Spi1, a TF reported to regulate hematopoietic lineage commitment[41], exhibited a higher regulon specificity score (RSS) and higher gene expression level in HSPCs and preferentially targeted genes with mature-lost TSSs. In contrast, Stat3, which has been reported to regulate CD8+ T cell differentiation[42], was highly activated at both the regulon and gene level in mature cells and preferentially targeted genes carrying mature-gained TSSs (**Extended Data Fig. 3m**)."*

For the COVID-19 5' scRNA-seq dataset (Pg. 13, ln. 375-379):

> *"Furthermore, we applied SCENIC to identify upstream TFs regulating SEMA4D in naïve CD8 cells and revealed TCF7 as a potential regulator. TCF7 has been reported to play a crucial role*

*in maintaining T cell self-renewal and antiviral immune function[45], which is also associated with increased regulon activity and gene expression levels in COVID-19 individuals (**Extended Data Fig. 4d-e**).”*

For the NSCLC 5’ scRNA-seq (Pg. 13, ln. 400-404):

*“To characterise the potential regulation between TFs and TSSs, we identified 26 regulons organised into 4 major modules (**Extended Data Fig. 5c**). For example, the STAT1 and ELF1 TFs were linked to immune-related and invasive processes in NSCLC[58,59], and both preferentially targeted genes with dominant TSS usage in malignant cells, including CCR6 and RTKN2 (**Extended Data Fig. 5d-e**).”*

We updated the ‘Methods’ section in Pg. 31, ln. 963-974:

*“**SCENIC gene regulation network analysis***

To examine the regulatory activity of transcription factors (TF), we applied the single-cell regulatory network inference and clustering (pySCENIC, v0.12.1)[40] by using normalised expression matrices of 5’ scRNA-seq. The pipeline comprises three main steps: infer gene regulatory network (GRN) based on co-expression patterns, predict regulon based on motif discovery (cisTarget), and quantify the predicted regulon activity with AUCell scores. The regulon connection specificity index (CSI) matrix was calculated as previously described[95]. Using this method, we compared (a) the host genes carrying gain or loss TSSs in the mHSPCs dataset, (b) host genes with differential TSSs usage in naïve CD8 T cells in the COVID-19 dataset, and (c) host genes with differential TSSs usage in malignant cells in the NSCLC dataset.”

In Pg. 36, ln. 1145-1146:

“R package ggseqlogo (v0.1)[103] was used to generate the sequence logo of FIMO-searched sequences.”

3.Additional validation on independent datasets would be valuable. The current examples feel more illustrative than conclusive.

Response: We thank the reviewer for this valuable suggestion. To address this concern, we have substantially expanded the validation and comparison using public and in-house independent datasets as follows:

1. We added additional long-read sequencing datasets for this study, enabling direct validation of TSS usage and degradation patterns detected by scATS. Across these newly included datasets, we found that the proportion of TSSs supported by all three long-read platforms is higher for scATS than for SCAFE, Camotss, TSSr and scTSS, demonstrating the improved reliability of our method (revised **Fig. 2b, d**).
2. We further incorporated other evidence from CAGE-seq and ATAC-seq, both of which support the TSS locations inferred by scATS (revised **Extended Data Fig. 2c-g**). Importantly, the TSS associated with our functionally validated gene *RTKN2* is also supported by CAGE-seq signals (revised **Extended Data Fig. 7a**), providing additional confidence in the biological relevance of our predictions.

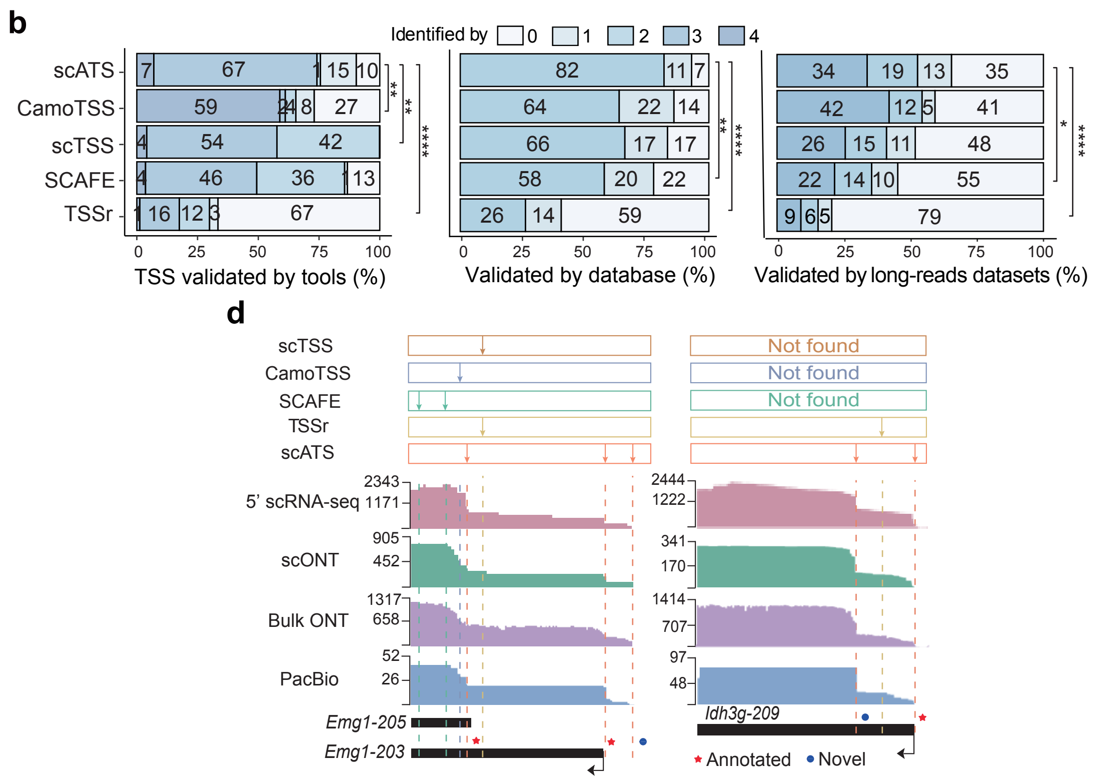

*Revised **Fig. 2b**, Validation of TSSs identified by different methods using independent evidence with a 50 bp distance threshold to define matched TSSs. Values indicate the number of supporting evidence*

*sources (0, no support). Evidence includes TSS-detection tools (scATS, SCAFE, CamoTSS, TSSr and scTSS; left), annotation databases (GENCODE and refTSS; middle), and long-read datasets (scONT, ONT, and PacBio; right). The percentage of TSSs identified by different methods was assessed using a two-sided proportion test with BH correction; *, p < 0.05; **, p < 0.01; ****, p < 0.0001; comparisons without labels are not significant, unless stated otherwise.*

***d**, Sashimi plots showing the transcription level of Emg1 (left panel) and Idh3g (right panel) in 5' scRNA-seq (pink), scONT (green), ONT (purple) and PacBio (blue) datasets of mHSPC. Arrows (upper panels) and the corresponding dashed lines indicate the loci of predicted ATSs inferred by different methods. Annotation shown below the sashimi plots with annotated and novel TSSs indicated.*

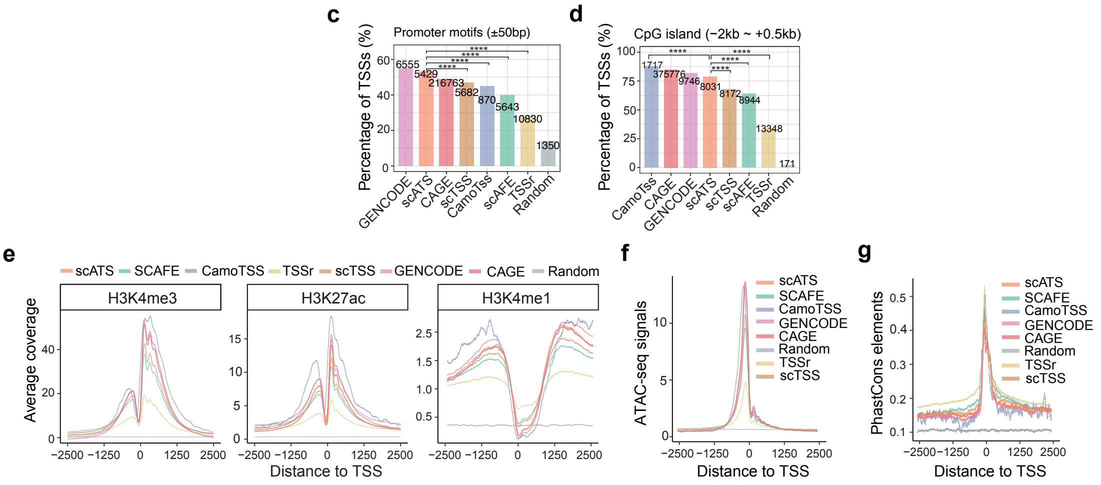

Revised **Extended Data Fig. 2c-g**, *Distribution of promoter motifs (c), CpG islands (d), histone modifications (e), ATAC-seq signals (f) and PhastCons elements (g) within the indicated distance around the GENCODE-annotated and identified TSSs. Random, genomic positions randomly sampled outside the regions of annotated TSSs. The two-sided proportion tests with BH correction were performed on the percentages of TSSs in pairwise comparisons. The numbers of TSSs are shown above the bars (c) and (d).*

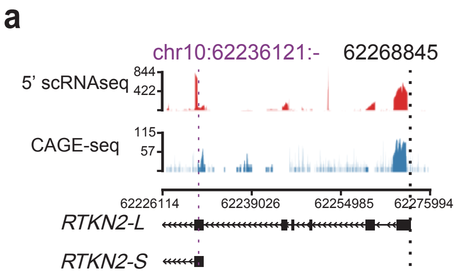

Revised **Extended Data Fig. 7a**, *Sashimi plots showing the transcription level of RTKN2 in 5' scRNA-seq (red) and CAGE-seq (blue) datasets of NSCLC patients. The corresponding dashed lines indicate the loci of predicted ATSs inferred by scATS. The TSS with higher expression in the malignant cells are highlighted in purple. Annotation shown below the sashimi plots.*

Writing

1. The structure of the abstract is not that clear. The abstract mentioned cancer but no COVID-19, however COVID-19 is one of the keywords in the first page. The abstract and the keywords should be reorganized so that people can mainly/roughly understand that it is the paper and there shouldn't be too many small things in the abstracts and keywords. Too many key words that confuse people.

Response: We apologize for the lack of clarity. We agree that the COVID-19-related analysis represents a relatively small part of the study and is not a major focus of the abstract. We have removed COVID-19 from the list of keywords and reorganised the abstract to better reflect the main scope and contributions of the work:

In the revised 'keywords' (Pg. 1, ln. 20-22):

> *"Alternative transcription start sites, RNA degradation, cell differentiation, lung cancer, single-cell RNA sequencing."*

Reviewer #4 (Remarks to the Author):

General Assessment：

The study by Xu et al. presents a robust and thoughtfully executed piece of work introducing a novel software tool that substantially advances current methods for identifying transcription start sites (TSS) and distinguishing between degraded and intact transcripts. The results are noteworthy, as the authors demonstrate clear improvements in accuracy and resolution across multiple datasets compared with existing approaches, highlighting the software's potential value for transcriptomic studies.

The work is likely to be of significant interest to researchers in transcriptomics and related fields, as it addresses an important analytical gap with a well-designed computational solution. The conclusions are generally well supported by the data, and the inclusion of experimental validation adds credibility to the claims. The methodology appears sound and meets accepted standards in the field, although providing additional detail on certain implementation parameters and recommended sequencing depths would enhance reproducibility and user adoption.

Specific Comments

Software

1. It would be helpful if the authors could provide guidance on the recommended sequencing depth for optimal performance of this software. Given that transcription start site identification and classification (e.g., degraded vs. intact transcripts) can be highly sensitive to read coverage, particularly around 5′ ends, information on minimal and ideal read depths would assist users in experimental planning. This is especially relevant for complex datasets containing multiple cell types, where the effective sequencing depth per cell type can vary substantially depending on relative cell abundances and transcriptional activity. Clarifying these requirements or providing benchmarking data across a range of sequencing depths would strengthen the practical utility of the tool.

Response: We thank the reviewer for this helpful suggestion regarding recommended sequencing depth for optimal use of scATS. To guide sequencing depth, we first down-sampled sequencing depths of the COVID-19 5' scRNA-seq dataset (revised **Supplementary Note 8**), as this dataset has the highest sequence depth. We observed that the numbers of detected TSS and genes with ATSs increased with sequencing depth and reached saturation around 25,000 reads per cell (revised **Supplementary Note Fig. 8e**). Next, we used the two commonly employed clustering metrics, Normalised Mutual Information (NMI) (Strehl & Ghosh, 2002) and adjusted Rand index (ARI) (Gates & Ahn, 2017) to evaluate the required sequencing depth for a correct cell clustering based on TSSs counts. At 200 reads per cell, TSS-based clustering showed poor agreement with gene-based cell type annotations (NMI = 0.10; ARI = 0.01), whereas increasing the depth to 2,000 reads per cell substantially improved clustering performance (NMI = 0.39; ARI = 0.22), with only marginal gains at higher depths (revised **Supplementary Note Fig. 8f**). These results suggest that sequencing depths around 2,000 reads per cell may provide the basic cell clustering according to TSS, but to dissect the full ATS landscape and identify most of ATS loci in a single cell require over 25,000 reads per cell.

Second, to address confidence and thresholding in TSS definition, we performed additional simulations to examine how gene-level read depth affects the accuracy of TSS detection (revised **Supplementary Note 3**). Using simulated data, we found that when the number of reads per gene was limited to 200, the correlation between the estimated alpha value and the ground truth was 0.61. Increasing the sequence depth can substantially improve the correlation, reaching 0.75 and 0.89 when 300 and 500 reads are available, respectively (revised **Supplementary Note Fig. 8a**). Because gene-level read depth directly corresponds to the 'MinGeneReads' parameter in scATS, this parameter can be used to control detection stringency.

Finally, we have added **Supplementary Note 8** to describe how users can pre-examine gene-level coverage in their own data to guide the choice of the 'MinGeneReads' parameter, and details to evaluate the impact of sequencing depth.

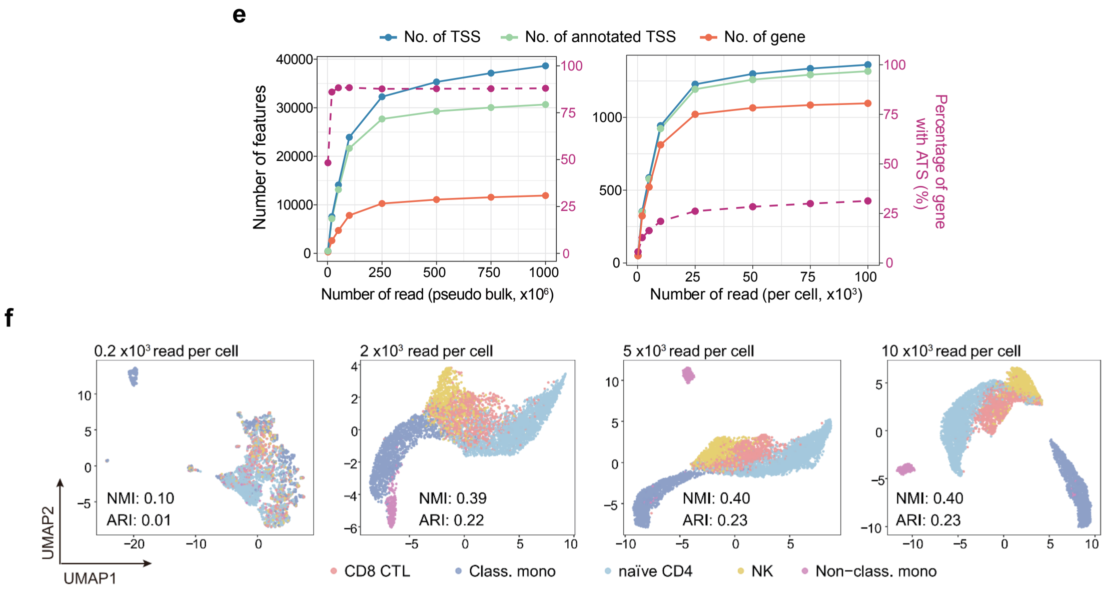

*Revised **Supplementary Note Fig. 8e**, Pseudo-bulk (left panel) and single-cell level (right panel) analyses of TSS detection across downsampled sequencing depths in the COVID-19 5' scRNA-seq dataset. Line charts show the numbers of detected TSSs, annotated TSSs and host genes (left Y-axis), and red line charts show the percentage of genes with ATSs (right Y-axis). Sequencing depth is indicated on the X-axis. Predicted TSSs located within ±50 bp of CAGE-seq peaks were considered annotated, while others were classified as novel.*

***f**, UMAP plots of cell clustering based on TSS count matrices at different sequencing depths in the COVID-19 5' scRNA-seq dataset. Clustering performance was quantified using NMI and ARI scores. Cell-types are indicated by different colors.*

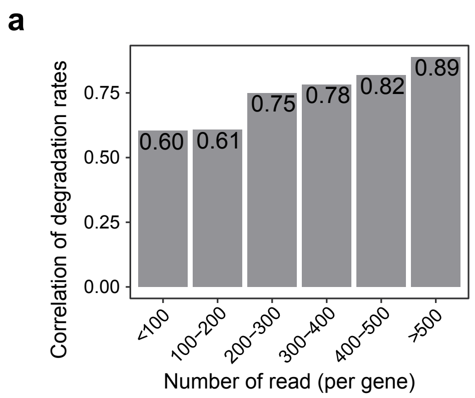

Revised ***Supplementary Note Fig. 8a.*** *Bar plots showing the correlation between estimated alpha values and ground truth across different gene-level read depth groups in the simulated 5' scRNA-seq dataset. Pearson correlation coefficients are shown in each bar.*

2. I noticed that the software repository does not list dependencies (e.g., required Python libraries) needed for installation. Including this information would improve ease of installation and reproducibility for new users.

Response: We apologize for this oversight. We have now added an LRS.yml file to the GitHub repository (https://github.com/LuChenLab/r-scATS) (**Figure R4.1**), which lists all required dependencies (**Table R4.1**) and allows users to recreate the exact conda environment used in our analysis. The online documentation has been updated accordingly, and a screenshot of the revised documentation page is provided in **Figure R4.2**, which is available at https://r-scats.readthedocs.io/en/latest.

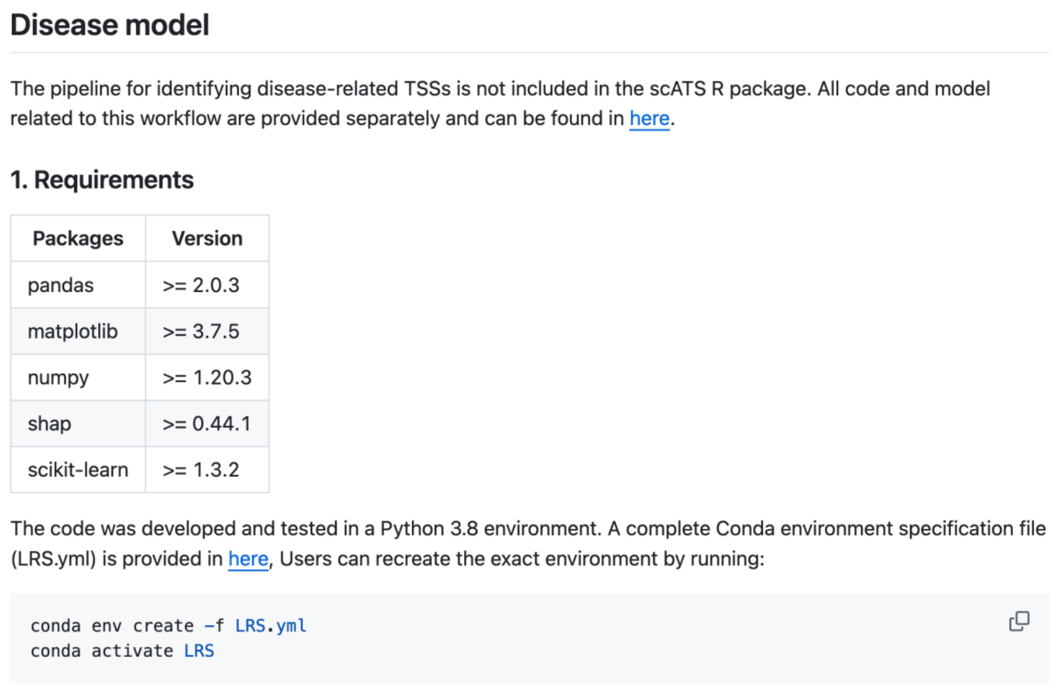

Disease model

The pipeline for identifying disease-related TSSs is not included in the scATS R package. All code and model related to this workflow are provided separately and can be found in here.

1. Requirements

Packages	Version
pandas	>= 2.0.3
matplotlib	>= 3.7.5
numpy	>= 1.20.3
shap	>= 0.44.1
scikit-learn	>= 1.3.2

The code was developed and tested in a Python 3.8 environment. A complete Conda environment specification file (LRS.yml) is provided in here, Users can recreate the exact environment by running:

```
conda env create -f LRS.yml
conda activate LRS
```

Figure R4.1. Screenshot of the updated GitHub repository.

Table R4.1. Python packages included in the LRS conda environment.

Packages	Packages
anyio==4.5.2	nbclient==0.10.1
argon2-cffi==25.1.0	nbconvert==7.16.6
argon2-cffi-bindings==21.2.0	nbformat==5.10.4
arrow==1.4.0	nest-asyncio==1.6.0
asttokens==3.0.1	notebook==7.3.3
async-lru==2.0.4	notebook_shim==0.2.4
attrs==25.3.0	numba==0.58.1
babel==2.17.0	numpy==1.24.4
backcall==0.2.0	overrides==7.7.0
backports.zoneinfo==0.2.1	packaging==25.0
beautifulsoup4==4.14.2	pandas==2.0.3
bleach==6.1.0	pandocfilters==1.5.1
certifi==2025.11.12	parso==0.8.5
cffi==1.17.1	pexpect==4.9.0
charset-normalizer==3.4.4	pickleshare==0.7.5
cloudpickle==3.1.2	pillow==10.4.0
comm==0.2.3	pkgutil_resolve_name==1.3.10
contourpy==1.1.1	platformdirs==4.3.6
cycler==0.12.1	prometheus_client==0.21.1
debugpy==1.8.17	prompt_toolkit==3.0.52

decorator==5.2.1	psutil==7.1.3
defusedxml==0.7.1	ptyprocess==0.7.0
exceptiongroup==1.3.1	pure_eval==0.2.3
executing==2.2.1	pycparser==2.23
fastjsonschema==2.21.2	Pygments==2.19.2
fonttools==4.57.0	pyparsing==3.1.4
fqdn==1.5.1	python-dateutil==2.9.0.post0
h11==0.16.0	python-json-logger==4.0.0
httpcore==1.0.9	pytz==2025.2
httpx==0.28.1	PyYAML==6.0.3
idna==3.11	pyzmq==27.1.0
importlib_metadata==8.5.0	referencing==0.35.1
importlib_resources==6.4.5	requests==2.32.4
ipykernel==6.29.5	rfc3339-validator==0.1.4
ipython==8.12.3	rfc3986-validator==0.1.1
ipywidgets==8.1.8	rpds-py==0.20.1
isoduration==20.11.0	scikit-learn==1.3.2
jedi==0.19.2	scipy==1.10.1
Jinja2==3.1.6	Send2Trash==1.8.3
joblib==1.4.2	shap==0.44.1
json5==0.12.1	six==1.17.0
jsonpointer==3.0.0	slicer==0.0.7
jsonschema==4.23.0	sniffio==1.3.1
jsonschema-specifications==2023.12.1	soupsieve==2.7
jupyter==1.1.1	stack-data==0.6.3
jupyter-console==6.6.3	terminado==0.18.1
jupyter-events==0.10.0	threadpoolctl==3.5.0
jupyter-lsp==2.3.0	tinycss2==1.2.1
jupyter_client==8.6.3	tomli==2.3.0
jupyter_core==5.8.1	tornado==6.4.2
jupyter_server==2.14.2	tqdm==4.67.1
jupyter_server_terminals==0.5.3	traitlets==5.14.3
jupyterlab==4.3.8	typing_extensions==4.13.2
jupyterlab_pygments==0.3.0	tzdata==2025.2
jupyterlab_server==2.28.0	uri-template==1.3.0
jupyterlab_widgets==3.0.16	urllib3==2.2.3
kiwisolver==1.4.7	wcwidth==0.2.14
llvmlite==0.41.1	webcolors==24.8.0
MarkupSafe==2.1.5	webencodings==0.5.1
matplotlib==3.7.5	websocket-client==1.8.0
matplotlib-inline==0.1.7	widgetsnbextension==4.0.15
mistune==3.1.4	zipp==3.20.2

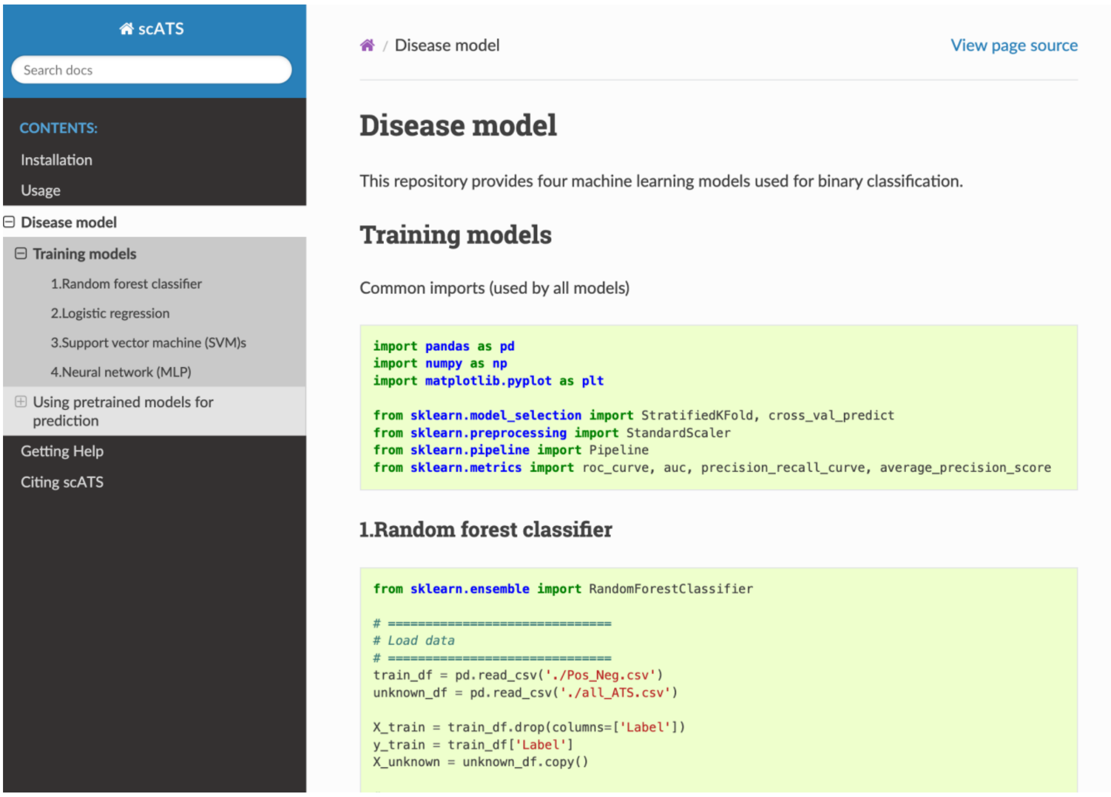

Figure R4.2. Screenshot of the updated online documentation.

Manuscript

1. Line 193: "TDD identified by both scATS and SCAFE account for 69%..." — The second half of this sentence does not make sense ("whole only…"). Please revise for clarity.

Response: We apologize for the confusion. This was a typographical error in the manuscript, where "whole" was mistakenly used instead of "while". In addition, the 'Results' section has been updated to include two additional methods, TSSr and scTSS, following the suggestion of reviewer #3. We revised the 'Results' section as follows (Pg. 8, ln. 226-231):

> *"TSSr, which is developed for bulk RNA-seq, identified most TSSs (n = 39,214). Three single-cell methods SCAFE (n = 14,006), scTSS (n = 11,990) and scATS (n = 10,195;* ***Supplementary Table 3****), reported comparable numbers of TSSs, and CamoTSS showed the lowest number of TSSs (n = 1,955) (****Fig. 2a****; see* ***Supplementary Note 4****)."*

2. Line 238: The introduction of Capg in the second part of the paragraph is confusing. The first part clearly describes the 12 factors identified from MOFA analysis, but the rationale for introducing Capg here is unclear. Was Capg a major contributor to the F2 signal?

Response: Thank you for the comment. MOFA Factor 2 (F2) reflects TSS expression patterns across hematopoietic differentiation. To pinpoint the ATS that are highly variable during hematopoiesis, we performed a pseudo-bulk analysis to compare the changes in ATS from different cell types. The proximal ATS of *Capg* (chr6:72549434:+) showed high variability (variance = 0.080; Standard Deviation, SD = 0.283), and its two TSSs are cell-type specific, indicating its contribution to the cell clustering. Thus, it was used as an example of a TSS that undergoes significant changes during differentiation.

3. Figure 3i / Capg discussion: For neurons, there appear to be two TSSs, each contributing roughly 50% of the signal. However, there is also some broad signal near the start of transcript Capg-204. Was this signal considered, or was it interpreted as degradation and therefore excluded from the final analysis? Clarification would help readers understand how diffuse signals are treated by the method.

Response: We appreciate this insightful question, which highlights a common challenge in single-cell TSS quantification. Several considerations are relevant here.

First, scATS interfaces directly with Seurat, and ATS identification and quantification are performed on the entire dataset, using both the global Seurat object and the corresponding BAM file. As the reviewer noted, the diffuse reads near the annotated start of Capg-204 are observed only in neutrophils, which does not meet our quantitative threshold for calling a TSS. Briefly, as detailed in revised **Supplementary Note 8**, we found that more than 200 reads per gene allow reliable estimation of the degradation rate, and more than 2,000 reads per cell support improved cell clustering.

Second, as shown in **Figure R4.3**, these reads fall within an intronic region of *Capg-204* and lack splice junctions. Notably, neutrophils are known to have overrepresented intron reads (Wong et al., 2013). These may be neutrophil-specific intronic reads. We also assessed the coding potential

of the region corresponding to the observed reads using the CPC2 website (Kang et al., 2017) and obtained a low probability score of 0.07, further supporting a noncoding region.

Third, we considered whether these reads originate from lncRNAs. Only one annotated lncRNA, *Gm38832*, was identified near the *Capg-204* locus, located about 385 kb upstream and without overlap with *Capg-204* (**Table R4.2**). We further analysed this region using EVlncRNA-Dpred (Zhou et al., 2023), a tool for predicting experimentally validated functional lncRNAs, and the result indicated that this region is not a candidate functional lncRNA.

Based on this assessment, the two TSSs shown in revised **Fig. 2h** represent the only robust TSSs supported by uniquely mapped, junction-consistent reads in neutrophils. The intronic reads near the start of *Capg-204* does not meet these criteria and therefore does not affect TSS identification or quantification in neutrophils.

Table R4.2. Genomic coordinates of *Capg-204* and the nearby annotated lncRNA *Gm38832*.

Gene/Tx	Chromosome	Start	End	Strand
Capg-204	Chr6	72552937	72558257	+
Gm38832	Chr6	72162848	72162848	+

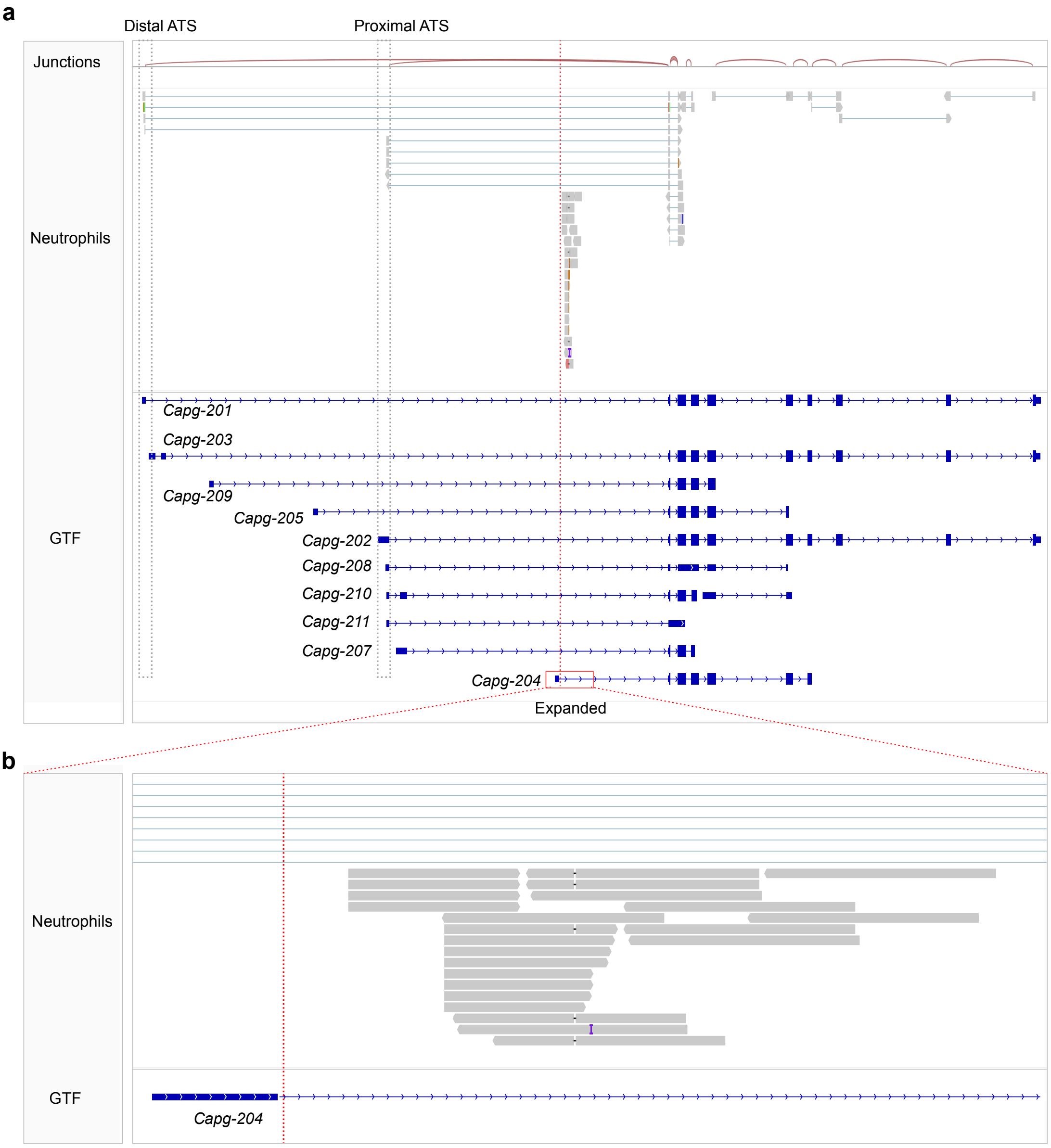

Figure R4.3. IGV visualisation of the *Capg* locus in neutrophil cells of mHSPC 5' scRNA-seq dataset. **a,** Gene locus of *Capg* with junction-coverage and read-coverage tracks from the split BAM file of neutrophil cells. Candidate ATSs are marked by grey dashed boxes. The GTF annotation is shown below with exons in blue and introns indicated by directional arrows. The red "Expanded" box highlights the first-exon region of the *Capg-204* transcript corresponding to panel b, and the red dashed vertical line marks the annotated end of its first exon.
b, Zoom-in view of the first exon of *Capg-204*. Read coverage in neutrophils is displayed above the GTF annotation.

4. Figure 2k and associated text: Was there a trend in the loss of TSS (i.e., were distal or proximal sites preferentially lost)? Including this information could provide additional biological insight.

Response: We thank the reviewer for this helpful suggestion. We observed a clear trend in lost TSSs, with proximal TSSs being preferentially lost during differentiation and accounting for 61%

of all lost TSSs(revised **Extended Data Fig. 3e**). These proximal TSSs showed a higher proportion of exonic localisation (revised **Extended Data Fig. 3f**) and lower downstream sequence conservation (revised **Extended Data Fig. 3g**). This pattern suggests that proximal and distal TSSs are regulated differently during differentiation.

To provide possible biological insight, we considered this observation in the context of haematopoietic differentiation, which is a tightly regulated process that relies on multiple layers of transcriptional and post-transcriptional control. From this perspective, we propose two possible explanations:

1. One possible explanation is that changes in TSS usage shift the TSS along the gene body, which has been reported to alter transcript architecture and isoform composition, and can lead to differences in cDNA length and encoded protein structure (Demircioğlu et al., 2019).
2. The higher loss of proximal TSSs during differentiation may be associated with alternative splicing activity, which could limit the functional contribution of proximal initiation sites (Sarda et al., 2017).

These interpretations remain speculative, but they highlight an interesting biological question and a potential direction for future studies.

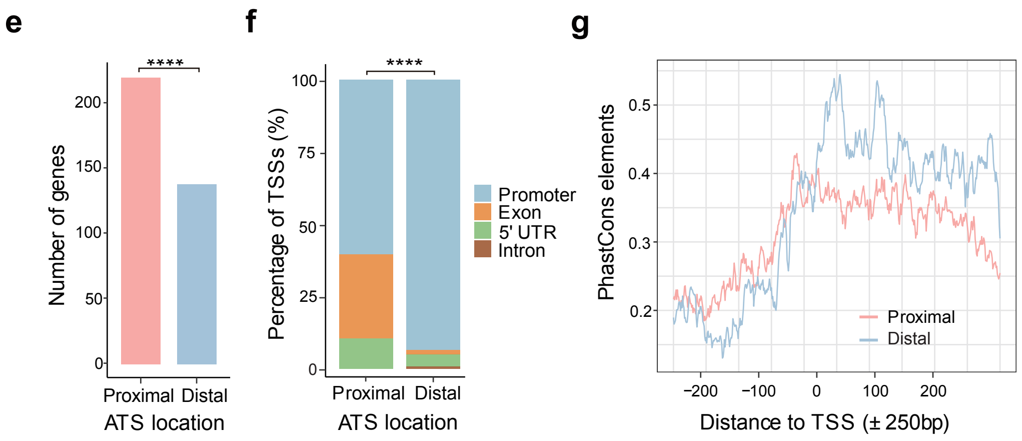

*Revised **Extended Data Fig. 3e-f**, Bar charts showing the distribution of the position (e) and localisation (f) of mature-loss TSSs. Two-sided proportion tests with BH correction were performed on the host gene (e) and 'Exon' ATS (f) between proximal and distal mature-lost TSSs.*
***g**, Distribution of PhastCons elements within the indicated distance around the proximal (pink) and distal (blue) mature-lost TSSs.*

We revised the 'Results' section (Pg. 10-11, ln. 306-309) as follows:

> *"We observed that the majority of mature-lost TSSs originated from proximal sites (61%; **Extended Data Fig. 3e**), and these TSSs showed a higher proportion of exonic localisation (**Extended Data Fig. 3f**) and lower downstream sequence conservation (**Extended Data Fig. 3g**)."*

5. Line 278: Typographical error — "sperated" should be changed to "separated."

Response: We apologise for this typographical error and any confusion it may have caused. We have corrected "sperated" to "separated" in the revised manuscript.

6. Figure 6 (Rtkn2): The alternating transcription start sites of Rtkn2 is convincing. However, the Rtkn2 gene model suggests two transcript lengths can arise from the distal ATS (refer GENCODE annotations). Can the authors be confident that this distal site produces only the long isoform? A cross-reference to GTEx IGV data shows that the short isoform can also be expressed (coverage of the 3′UTR at chr10:62,238,149–62,239,643). Discussing this would clarify whether the distal site is strictly associated with the long isoform.

Response: We thank the reviewer for this comment. To better explain this issue, we included lung cancer 3'scRNA seq datasets (Zhang et al., 2022) and illustrated it in detail in **Figure R4.4**. The IGV zoomed-in visualisation of the 3' scRNA-seq datasets provides important insights into isoform expression. Specifically, the 3' untranslated region (3' UTR) of the *RTKN2-203* isoform is not detected at appreciable levels in LUAD and LUSC samples (highlighted in the red box). The absence or minimal expression of the *RTKN2-203* implies that this isoform is either not transcribed or transcribed at a very low level in the specific lung cancer cell populations. Alternatively, the 3' UTR sequence of *RTKN2-203* fails to occur as predicted by the annotation, or it is present at levels below the detection threshold of our single-cell sequencing methodology.

In contrast to *RTKN2-203*, other isoforms, particularly *RTKN2-201* (*RTKN2-S*, the short form) and *RTKN2-202* (*RTKN2-L*, the long form), are robustly detected across LUAD and LUSC samples. This differential isoform expression pattern suggests that the alternative splice variants, namely *RTKN2-201* and *RTKN2-202*, are the predominant functional transcripts expressed in LUAD and

LUSC patients, and therefore likely represent the biologically relevant isoforms in these malignancies.

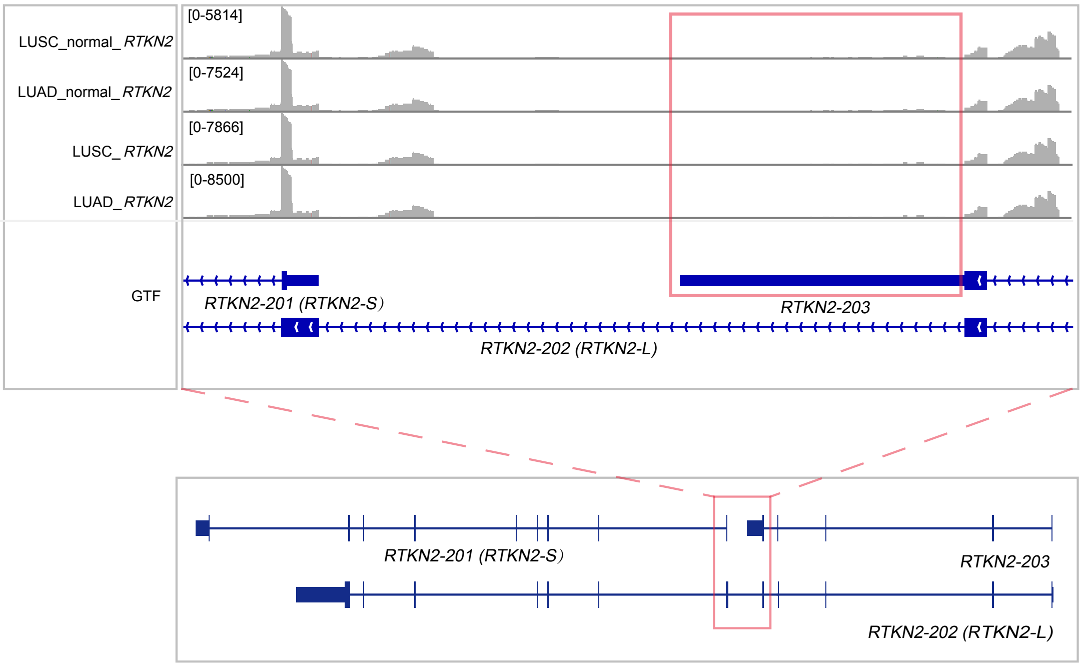

Figure R4.4. *IGV visualisations of the RTKN2 loci in normal and tumor samples from LUSC and LUAD patients based on 3' scRNA seq datasets are shown. Three annotated RTKN2 isoforms, RTKN2-201 (RTKN2-S), RTKN2-202 (RTKN2-L), and RTKN2-203, are displayed below. The last exon and 3' UTR of RTKN2-203 are highlighted with a red box. The lower panel shows a complete view of the upper panel, illustrating the exon–intron structure of the three isoforms.*

Summary

Overall, this manuscript introduces a valuable methodological advance and provides convincing supporting data. With minor clarifications in the manuscript text, additional benchmarking on sequencing depth requirements, and improvements to software documentation, the work will be further strengthened and broadly useful to the community.

Response: We thank the reviewer for the positive and encouraging evaluation of our work.

Reference

Clements, M., Tang, W., Florjanic Baronik, Z., Simpson Ragdale, H., Oria, R., Volteras, D., White, I. J., Beattie, G., Uddin, I., Lenn, T., Lindsay, R., Castro Devesa, S., Karamched, S. R., Lythgoe, M. F., Shahrezaei, V., Weaver, V. M., Sugisawa, R., Roncaroli, F., Marguerat, S., … Parrinello, S. (2025). Axonal injury is a targetable driver of glioblastoma progression. *Nature*, *646*(8084), 452–461.

Demircioğlu, D., Cukuroglu, E., Kindermans, M., Nandi, T., Calabrese, C., Fonseca, N. A., Kahles, A., Lehmann, K. Van, Stegle, O., Brazma, A., Brooks, A. N., Rätsch, G., Tan, P., & Göke, J. (2019). A Pan-cancer Transcriptome Analysis Reveals Pervasive Regulation through Alternative Promoters. *Cell*, *178*(6), 1465-1477.e17.

Duncan, C. G., Killela, P. J., Payne, C. A., Lampson, B., Chen, W. C., Liu, J., Solomon, D., Waldman, T., Towers, A. J., Gregory, S. G., McDonald, K. L., McLendon, R. E., Bigner, D. D., & Yan, H. (2010). Integrated genomic analyses identify ERRFI1 and TACC3 as glioblastoma-targeted genes. *Oncotarget*, *1*(4), 265–277.

Fu, S., & Li, W. V. (2025). Predicting and comparing transcription start sites in single cell populations. *PLoS Computational Biology*, *21*(4).

Gates, A. J., & Ahn, Y.-Y. (2017). The Impact of Random Models on Clustering Similarity. *Journal of Machine Learning Research*, *18*, 1–28.

Han, M. H., Min, K. W., Noh, Y. K., Kim, J. M., Cheong, J. H., Ryu, J. Il, Won, Y. D., Koh, S. H., & Park, Y. M. (2022). Identification of genes from ten oncogenic pathways associated with mortality and disease progression in glioblastoma. *Frontiers in Oncology*, *12*.

Hou, R., Hon, C. C., & Huang, Y. (2023). CamoTSS: analysis of alternative transcription start sites for cellular phenotypes and regulatory patterns from 5' scRNA-seq data. *Nature Communications*, *14*(1).

Hua, T., Shi, H., Zhu, M., Chen, C., Su, Y., Wen, S., Zhang, X., Chen, J., Huang, Q., & Wang, H. (2022). Glioma-neuronal interactions in tumor progression: Mechanism, therapeutic strategies and perspectives (Review). *International Journal of Oncology*, *61*(3).

Kamal, E., & Mohager, S. O. (2025). Dual Roles of Signal Transducer and Activator of Transcription 1 (STAT1) in Cancer: Expression Patterns, Prognostic Significance, and Immune Associations Across Multiple Tumor Types. *Cureus*, *17*(9).

Kang, Y. J., Yang, D. C., Kong, L., Hou, M., Meng, Y. Q., Wei, L., & Gao, G. (2017). CPC2: a fast and accurate coding potential calculator based on sequence intrinsic features. *Nucleic Acids Research*, *45*(W1), W12–W16.

Loughran, S. J., Haas, S., Wilkinson, A. C., Klein, A. M., & Brand, M. (2020). Lineage commitment of hematopoietic stem cells and progenitors: insights from recent single cell and lineage tracing technologies. *Experimental Hematology*, *88*, 1–6.

Lu, Z., Berry, K., Hu, Z., Zhan, Y., Ahn, T. H., & Lin, Z. (2021). TSSr: an R package for comprehensive analyses of TSS sequencing data. *NAR Genomics and Bioinformatics*, *3*(4).

Mizukoshi, C., Kojima, Y., Nomura, S., Hayashi, S., Abe, K., & Shimamura, T. (2024). DeepKINET: a deep generative model for estimating single-cell RNA splicing and degradation rates. *Genome Biology 2024 25:1*, *25*(1), 229.

Moody, J., Kouno, T., Chang, J. C., Ando, Y., Carninci, P., Shin, J. W., & Hon, C. C. (2022). SCAFE: a software suite for analysis of transcribed cis-regulatory elements in single cells. *Bioinformatics (Oxford, England)*, *38*(22), 5126–5128.

Paz, M. W., Vogel, T., & Nieselt, K. (2024). TSS-Captur: a user-friendly pipeline for characterizing unclassified RNA transcripts. *NAR Genomics and Bioinformatics*, *6*(4).

Pepke, S., Wold, B., & Mortazavi, A. (2009). Computation for ChIP-seq and RNA-seq studies. *Nature Methods*, *6*(11 Suppl), S22.

Sarda, S., Das, A., Vinson, C., & Hannenhalli, S. (2017). Distal CpG islands can serve as alternative promoters to transcribe genes with silenced proximal promoters. *Genome Research*, *27*(4), 553–566.

Strehl, A., & Ghosh, J. (2002). Cluster Ensembles-A Knowledge Reuse Framework for Combining Multiple Partitions. *Journal of Machine Learning Research*, *3*, 583–617.

Sun, Q., Zhao, X., Li, R., Liu, D., Pan, B., Xie, B., Chi, X., Cai, D., Wei, P., Xu, W., Wei, K., Zhao, Z., Fu, Y., Ni, L., & Dong, C. (2023). STAT3 regulates CD8+ T cell differentiation and functions in cancer and acute infection. *The Journal of Experimental Medicine*, *220*(4).

Tu, T. H., Grunbaum, A., Santinon, F., Kazanova, A., Rozza, N., Kremer, R., Mihalcioiu, C., & Rudd, C. E. (2024). Decreased progenitor TCF1 + T-cells correlate with COVID-19 disease severity. *Communications Biology*, *7*(1).

Wan, L., Yan, X., Chen, T., & Sun, F. (2012). Modeling RNA degradation for RNA-Seq with applications. *Biostatistics*, *13*(4), 734–747.

Wang, Z., Gerstein, M., & Snyder, M. (2009). RNA-Seq: a revolutionary tool for transcriptomics. *Nature Reviews. Genetics*, *10*(1), 57.

Wong, J. J. L., Ritchie, W., Ebner, O. A., Selbach, M., Wong, J. W. H., Huang, Y., Gao, D., Pinello, N., Gonzalez, M., Baidya, K., Thoeng, A., Khoo, T. L., Bailey, C. G., Holst, J., & Rasko, J. E. J. (2013). Orchestrated intron retention regulates normal granulocyte differentiation. *Cell*, *154*(3).

Yang, Y., Han, X., Sun, L., Shao, F., Yin, Y., & Zhang, W. (2024). ETS Transcription Factors in Immune Cells and Immune-Related Diseases. *International Journal of Molecular Sciences*, *25*(18).

Zhang, L., Zhang, Y., Wang, C., Yang, Y., Ni, Y., Wang, Z., Song, T., Yao, M., Liu, Z., Chao, N., Yang, Y., Shao, J., Li, Z., Zhou, R., Chen, L., Zhang, D., Zhao, Y., Liu, W., Li, Y., … Li, W. (2022). Integrated single-cell RNA sequencing analysis reveals distinct cellular and transcriptional modules associated with survival in lung cancer. *Signal Transduction and Targeted Therapy*, *7*(1).

Zhao, Y., Qin, J., Yu, D., Liu, Y., Song, D., Tian, K., Chen, H., Ye, Q., Wang, X., Xu, T., Xuan, H., Sun, N., Ma, W., Zhong, J., Sun, P., Song, Y., Hu, J., Zhao, Y., Hou, X., … Cai, J. (2024). Polymer-locking fusogenic liposomes for glioblastoma-targeted siRNA delivery and CRISPR–Cas gene editing. *Nature Nanotechnology 2024 19:12*, *19*(12), 1869–1879.

Zhou, B., Ding, M., Feng, J., Ji, B., Huang, P., Zhang, J., Yu, X., Cao, Z., Yang, Y., Zhou, Y., & Wang, J. (2023). EVlncRNA-Dpred: improved prediction of experimentally validated lncRNAs by deep learning. *Briefings in Bioinformatics*, *24*(1).

Point-by-point response

MS title: Accurate profiling of single-cell alternative transcript start sites by correcting RNA degradation

Dear reviewers:

We thank the reviewers for their valuable comments and suggestions. In response to the comments, we performed additional analysis and experiments, revised the manuscript (the changes are shown in red font) and updated the accompanying figures and tables. We addressed the concerns point-by-point under each comment. The changes introduced into the revised manuscript (MS) are shown in *italics*, with the corresponding page (Pg.) and line (ln.) numbers indicated.

Yours sincerely,

Lu Chen, PhD

Reviewer #1 (Remarks to the Author):

I co-reviewed this manuscript with one of the reviewers who provided the listed reports. This is part of the Nature Communications initiative to facilitate training in peer review and to provide appropriate recognition for Early Career Researchers who co-review manuscripts.

Response: We sincerely thank the reviewer and the co-reviewer for their valuable efforts.

Reviewer #2 (Remarks to the Author):

I co-reviewed this manuscript with one of the reviewers who provided the listed reports. This is part of the Nature Communications initiative to facilitate training in peer review and to provide appropriate recognition for Early Career Researchers who co-review manuscripts.

Response: We are grateful to the reviewer and the co-reviewer for their time and valuable suggestions.

Reviewer #2 (Remarks on code availability):

The r-scATS package appears complete with a nice github repo. However, one omission which none of the reviewers (including myself) brought up is that no paper reproducibility code (e.g. https://zenodo.org/records/7023163) is linked in the r-scATS repo or the paper. Please provide this critical supplement.

Response: We appreciate this suggestion. To ensure reproducibility, the full set of scripts used for figure generation in this manuscript has been deposited on Zenodo (https://zenodo.org/records/18829843; **Figure. R2.1**).

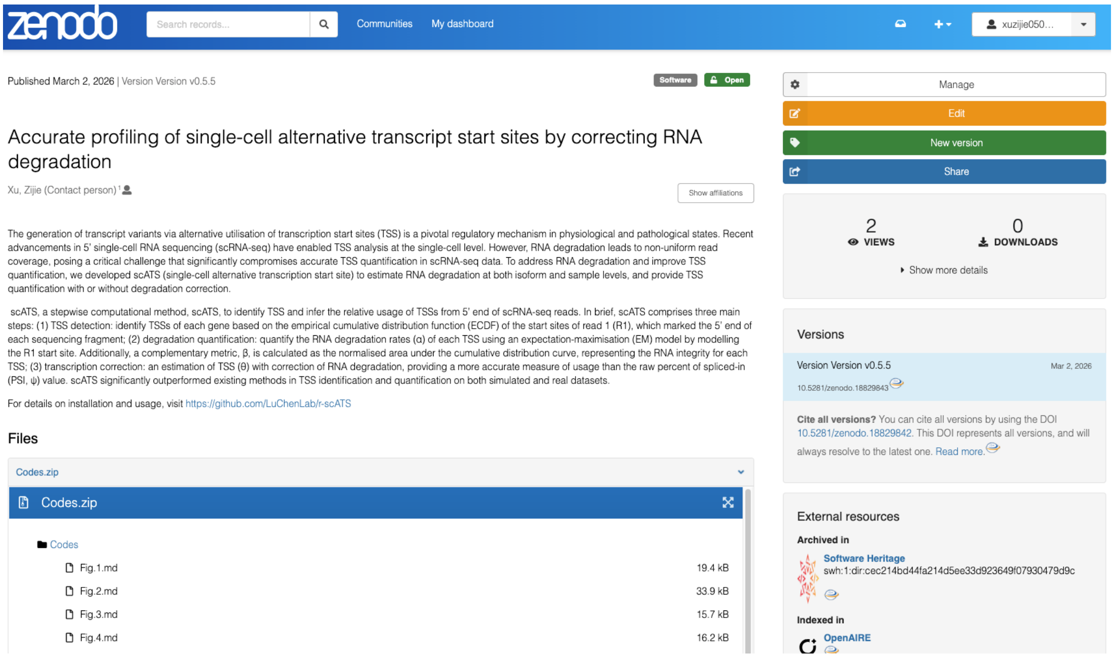

Figure R2.1. Screenshot of the updated scripts on Zenodo.

We revised the 'Code availability' section as follows (Pg. 47, ln. 1473-1476):

> *"The scATS R package is publicly available on Github (https://github.com/LuChenLab/r-scATS), and the analysis scripts used in the manuscript have been deposited on Zenodo[110] (https://zenodo.org/records/18829843)."*

Reviewer #3 (Remarks to the Author):

Summary

The authors have improved the manuscript by incorporating evaluations on simulated datasets, expanding their analysis, and providing a more detailed description of their methodology. The updated explanation of the degradation model is much clearer, and this revision addresses the majority of the concerns raised in the previous round of review.

Despite these improvements, the unique utility and novelty of the proposed method remain unconvincing in the following areas:

1. Novelty: the TSS identification and quantification will be valuable only if it can provide additional information that gene and isoform can not provide. Currently, the manuscript does not sufficiently showcase the distinct advantages of scATS. For example, scATS enable to identify the novel TSS which does not exist in annotation, but can be captured by scATS and also supported by the external data like CAGE or ONT or PacBio. Specifically, in the figure 2h, the distal ATS links to Capg-201 and the proximal ATS to Capg-208. All the information about start sites actually can be obtained from isofrom expression. What could be the additional value of this scATS?

Response: We thank the reviewer for these constructive comments. Firstly, in our mouse bone marrow analysis, 706 (6.9%) identified TSSs lack GENCODE support (novel TSSs), with 111 (15.7%) of these corroborated by the CAGE-seq dataset. A representative example is the *Pafah1b3* gene, which is implicated in haematopoietic differentiation via the regulation of cell activation pathways (Lin et al., 2025). *Pafah1b3* utilises a novel ATS (ATS2) that exhibits higher activity in mature cells, whereas the annotated ATS1 is more prevalent in progenitor cells (**Figure R3.1**). Both sites are cross-validated by CAGE-seq, providing external evidence for our findings.

Secondly, whilst we acknowledge that TSSs represent only a portion of full transcripts, TSS-level analysis offers distinct advantages (Fu & Li, 2025; Hou et al., 2023; Lu et al., 2021; Moody et al., 2022). Short-read sequencing (~150 bp) is generally insufficient for the *de novo* assembly of full-length transcripts (>1 kb) (Bray et al., 2016; Li & Dewey, 2011; Patro et al., 2017), whereas long-read platforms face throughput and error constraints that complicate quantification (Chen et al., 2023; Kovaka et al., 2019; Prjibelski et al., 2023). Given that most scRNA-seq datasets rely on 5'- or 3'-capture protocols, full-length transcript identification is often technically unfeasible (Moro et

al., 2026), making ATS and alternative polyadenylation (APA) analyses (Fahmi et al., 2025) standard in the field. From a biological perspective, transcripts sharing the same TSS typically utilise the same promoter and regulatory factors (Zhan et al., 2025). By providing a more direct and robust assessment of transcription initiation, TSS-based analysis represents both a technically feasible and biologically indispensable approach for studying single-cell transcriptional regulation.

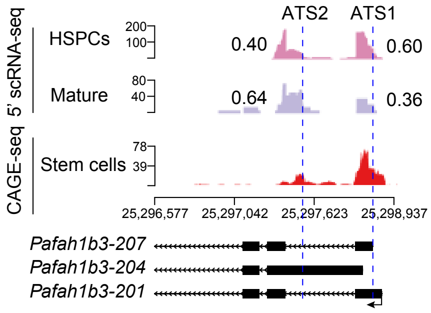

Figure R3.1. Sashimi plots showing the transcription levels of *Pafah1b3* in mHSPC 5' scRNA-seq and CAGE-seq datasets. The dashed lines indicate the loci of predicted ATSs inferred by scATS, with the PSIs of the corresponding ATSs provided near the peaks. Gene annotations are presented below the sashimi plots.

2. The enrichment analysis in Figure 3 is potentially misleading.

(1) For example, in figure 3. The authors performed GO enrichment analysis based on the host gene of differently utilised TSS, if these TSS is identified based on the same gene, like major or minor tss, then their host gene should be the same, further the GO enrichment should also be the same. If they are performed across all the genes, the GO enrichment was contributed by the differentially expressed gene. Which I think is a logic bug, if I understand the manuscript correctly.

Response: We agree with the reviewer that if each gene had only two TSSs, one would be major in one condition and minor in the other, and the host genes of differently utilised TSSs would be the same in both directions. In that case, GO enrichment results would also be the same. In our COVID-19 datasets, 64% of genes have more than two TSSs. This complexity means that genes with differential TSS usage often differ between conditions. For example, the two groups in our original **Fig. 3i** shared only 12% of their host genes. We agree that direction-based separation is hard to interpret, so we revised the GO analysis strategy. Instead of separating by direction, we group all genes that show a significant TSS switch between COVID-19 patients and healthy controls (HC).

Second, we acknowledge that functional enrichment is gene-centric. This is a standard limitation because most annotation resources are curated for genes rather than transcripts. Since no robust framework for TSS-level enrichment currently exists, these results reflect the functional context of genes with differential TSS usage.

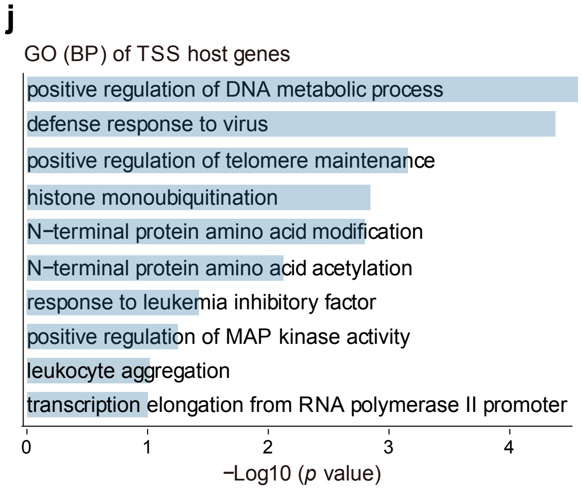

*Revised **Fig. 3j**, Gene Ontology (GO) enrichment analysis of the host genes with differentially used TSSs. BP, biological processes. The bars depict -log10 (p value).*

We revised the 'Results' section as follows (Pg. 13, ln. 380-383):

> *"Next, scATS identified 187 TSSs with differential TSS usage in naïve CD8 cells between COVID-19 and HC individuals (**Fig. 3i**). The host genes including NR2C1, SEMA4D and CD40, were enriched in pathways such as 'defense response to virus' and 'leukocyte aggregation' (**Fig. 3j**)."*

(2) To convincingly define phenotype-specific (like disease-related or cell-type-specific) TSSs, the authors should demonstrate cases where the overall gene expression remains stable while only the TSS usage or quantification significantly changes.

Response: Thank you for this comment. We examined phenotype-specific changes at both the gene and TSS levels in the COVID-19 and lung cancer datasets. In the COVID-19 dataset, fewer than 10% of genes across 13 out of 18 cell types were differentially expressed at both TSS and gene levels, ranging from 1% in MAIT cells to 30% in pDCs (revised **Extended Data Fig. 4c**). In the lung cancer dataset, 96% of disease-specific genes in malignant cells were identified only at the TSS level (revised **Extended Data Fig. 5d**). For example, *C1QA*, which contributes to an

immunosuppressive tumor microenvironment in NSCLC (Nguyen et al., 2025), showed disease specificity at both the gene expression and TSS usage (revised **Extended Data Fig. e**). In contrast, *CCR2*, which is linked to T-cell recruitment and favorable prognosis in NSCLC (Fan et al., 2023), showed disease specificity only at the TSS usage (revised **Extended Data Fig. f**).

c

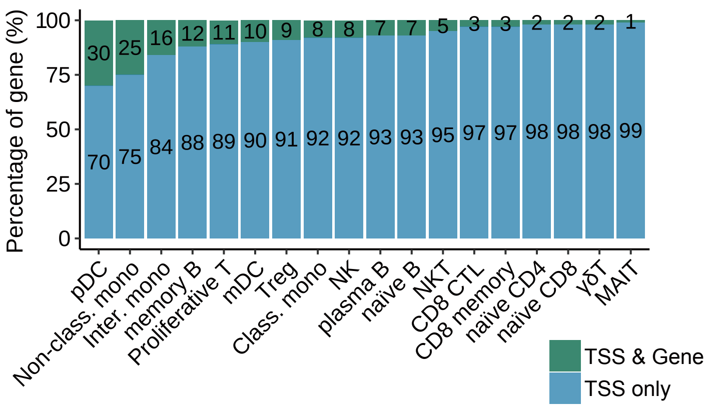

Revised ***Extended Data Fig. 4c,*** *Proportion of host genes of TSS markers with or without gene markers support in COVID-19 5' scRNA-seq datasets.*

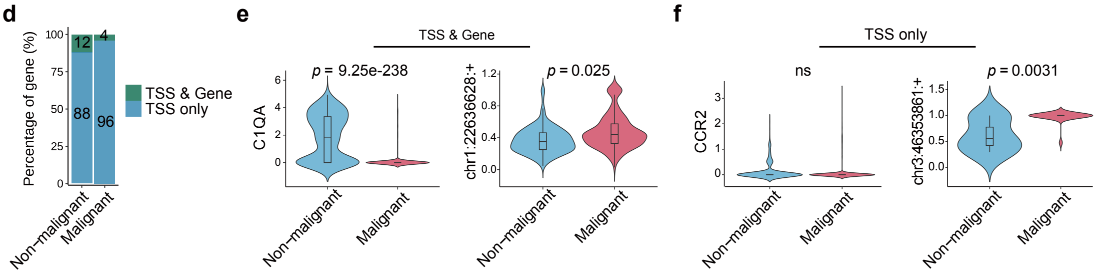

Revised ***Extended Data Fig. 5d,*** *Proportion of host genes of TSS markers with or without gene markers support in lung cancer 5' scRNA-seq dataset.*
e-f, *Violin plots depicting single-cell expression levels at gene (left) or TSS level (right) in C1QA (e) and CCR2 (f) genes. The central line represents the median, the box indicates the interquartile range, and the whiskers represent values within 1.5 times the interquartile range. ns: not significant; a two-sided Wilcoxon test with BH correction for multiple comparisons.*

We revised the 'Results' section as follows (Pg. 12-13, ln. 371-378):

> *"Furthermore, we compared these 5,291 differentially utilised TSSs (representing 3,455 host genes) with 3,441 differentially expressed genes identified across cell types. In 13 out of 18 cell types, fewer than 10% of genes were differentially expressed at both the TSS and gene levels, ranging from 1% in MAIT cells to 30% in pDCs (**Extended Data Fig. 4c**). This observation*

demonstrates that significant shifts in TSS preference can occur even when overall transcript abundance remains stable, highlighting a layer of regulation not captured by gene-level analysis."

In the Pg. 14, ln. 412-420:

*"To dissect these TSS-level shifts, we compared genes with differentially utilised TSSs to those with differential gene expression. Notably, 96% of the genes with differential TSS usage in malignant cells showed no significant change at the gene expression (**Extended Data Fig. 5d**). C1QA, which contributes to an immunosuppressive tumor microenvironment in NSCLC[59], showed disease specificity at both the gene expression and TSS usage (**Extended Data Fig. 5e**). In contrast, CCR2, which is linked to T-cell recruitment and favorable prognosis in NSCLC[60], showed disease specificity only at the TSS usage (**Extended Data Fig. 5f**)."*

3. For all the figures indicating alternative TSS and gene structure, it would be very helpful to show the transcript structure as well.

(1) In figure 1f, ATS2 after correction appears to align with the start site of annotated transcript Chchd2-201. Could the authors clarify whether these represent the exact same genomic coordinates?

Response: We thank the reviewer for pointing this out. As shown in revised **Fig. 1f**, ATS2 is associated with the *Chchd2-201* transcript. In the ATS2 region, high degradation leads to a decay of 5' reads, although approximately 30 reads remain intact. We have rechecked all sashimi plots in the revised MS.

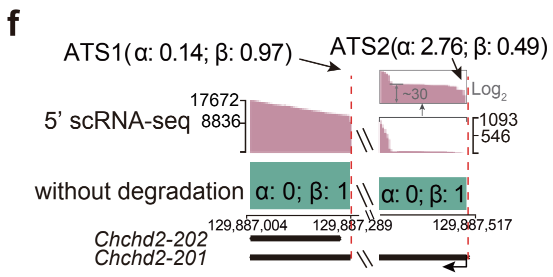

*Revised **Fig. 1f,** Sashimi plots showing the transcription level of Chchd2 in the mHSPC 5' scRNA-seq datasets. The distal ATS (ATS2) exhibits significant RNA degradation, resulting in a high α value of 2.76 and β value as low as 0.49. In contrast, the proximal ATS (ATS1) shows mild degradation. The*

red dashed lines indicate the loci of predicted TSSs inferred by scATS. The green boxes present the non-degraded state. GENCODE v25 annotations presented under the sashimi plots.

(2) In figure 3g, based on the illustrated exon structure, it appears that the ZFYVE28-210 isoform is not expressed. Could the authors please verify this or clarify the expression level of this specific transcript.

Response: We thank the reviewer for this insight. As shown in **Figure R3.2**, *ZFYVE28-210* includes an extended region downstream of *ZFYVE28-203*. We quantified the reads within this unique region and detected 56 reads, representing 2.1% of the total proximal ATS-supporting reads. Given this low expression level, we removed the *ZFYVE28-210* annotation from the revised **Fig. 3g** to avoid ambiguity and focus on the dominant isoforms.

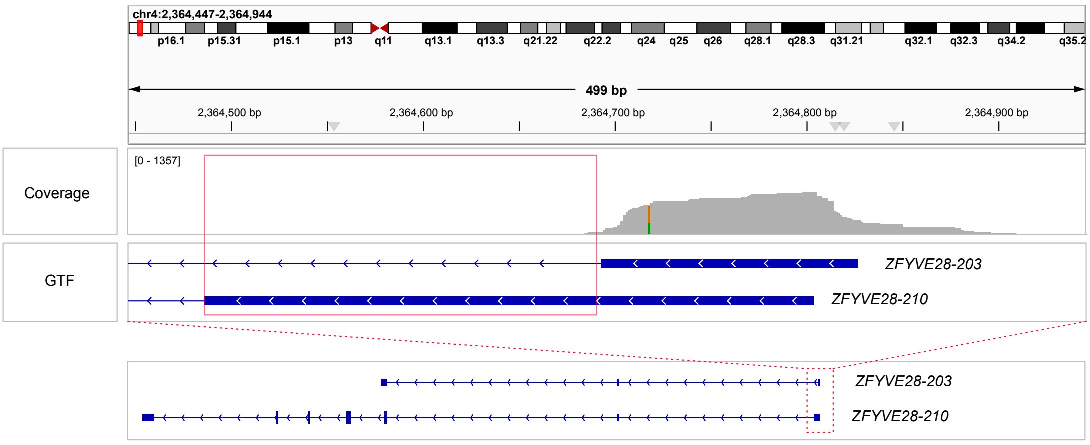

Figure R3.2. IGV visualisations of the *ZFYVE28* loci from COVID-19 5' scRNA-seq datasets are shown. *ZFYVE28-203* and *ZFYVE28-210* are displayed below. The region unique to the longer exon support *ZFYVE28-210* are highlighted with a red box. The lower panel shows a complete view of the upper panel, illustrating the exon–intron structure of the two isoforms.

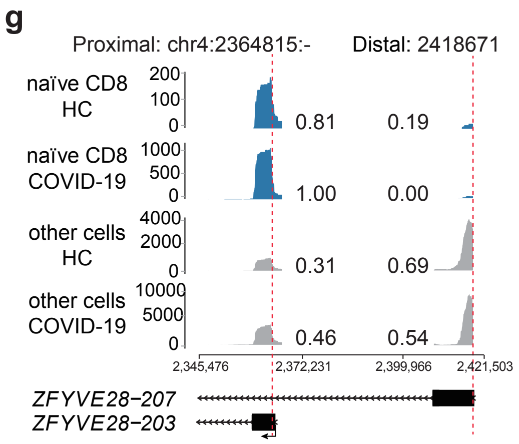

Revised ***Fig. 3g****, Sashimi plots showing the expression levels of ZFYVE28 gene and TSSs in naïve CD8 cells and other cells. The proximal ATS (chr4:2364815:-) was highl y transcribed in naïve CD8 cells of both COVID-19 and HC groups, while the distal ATS (chr4:2418671:-) was more highly utilised by the other cells. The red dashed lines indicate the TSS loci and PSI values of the corresponding ATS-derived isoforms are shown near the peaks. Annotations of the isoforms shown below the sashimi plot.*

(3) Same for the figure 5a, it's not clear whether the left TSS refers to a single exon transcript CCR6-L? For figure 5b, in the malignant sample, the read coverage does not appear to match the annotated exon structure of CCR2-L. Specifically, the high-coverage initial peak suggests the presence of an exon contributing to the signal. If the current model were correct, I would expect a relatively flat coverage profile that only increases at the start of the second TSS. Please check whether an additional exon or an alternative transcript structure is responsible for this peak.

Response: We thank for this comment. Firstly, the intron of *CCR6-L* was not shown in **Fig. 5a**, which led to ambiguity regarding whether the left TSS refers to a single-exon transcript. This has been corrected in the revised **Fig. 5a**.

Secondly, for *CCR2-L*, the sharp peak observed at the 5' end is a result of our specific data processing approach. Our scATS analysis and the corresponding visualisations rely on R1 reads, which are typically around 110 bp in 5' scRNA-seq (Hou et al., 2023). This was confirmed in IGV (**Figure R3.3**). We chose to use only R1 reads to maintain consistency with our analytical pipeline; whilst including R2 reads would produce a flatter coverage profile across the exon, it would not accurately reflect the data used for our signal detection. To avoid further ambiguity, we have added genomic coordinates to the revised **Fig. 5b** and to all other sashimi plots in our revised MS. We thank the reviewer for this helpful observation.

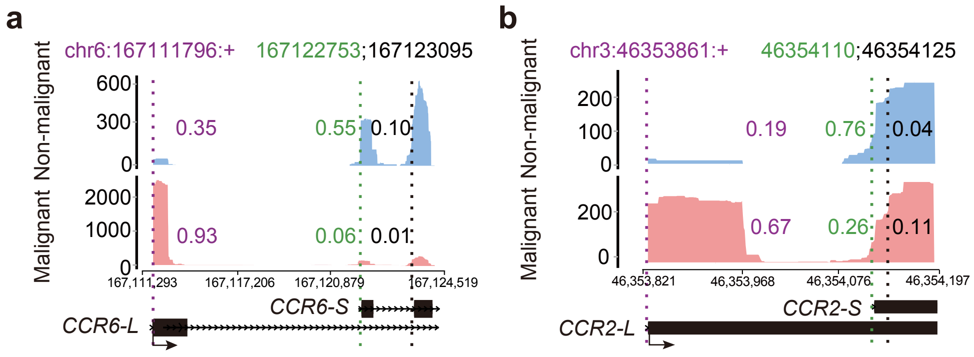

*Revised **Fig. 5a-b**, Sashimi plots showing the transcription level of CCR6 (a) and CCR2 (b) in the malignant (red) and non-malignant cells (blue) of NSCLC 5' scRNA-seq dataset. The dashed lines indicate the loci of predicted ATSs inferred by scATS with PSIs of the corresponding ATS-derived isoform provided near the peaks. The ATSs with higher expression in the malignant cells are highlighted in purple, ATSs transcribed higher in non-malignant cells are highlighted in green, and those with no significant differences are shown in black. Annotations provided below the sashimi plots.*

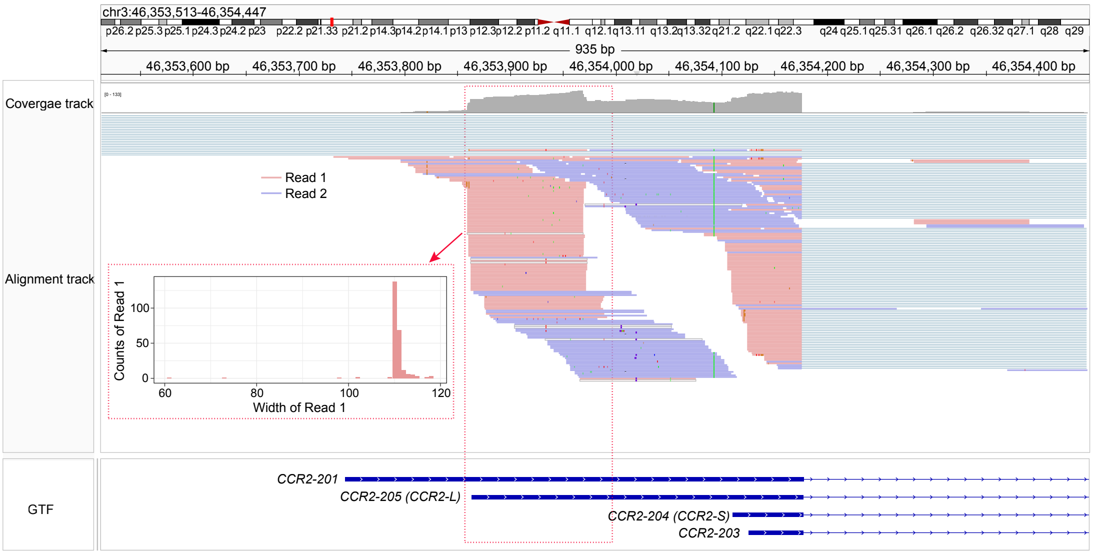

Figure R3.3. IGV visualisation of the *CCR2* loci in malignant cells from NSCLC 5' scRNA-seq dataset is shown. Four annotated *CCR2* isoforms, *CCR2-205* (*CCR2-L*), *CCR2-204* (*CCR2-S*), *CCR2-201* and *CCR2-203* are displayed below. R1 reads from the first exon of *CCR2-L* supporting the most distal ATS are highlighted with a red dashed box, and the distribution of R1 reads is shown on the left.

(4) Also in figure 1f, figure 2d, the transcript and exon structure are missing, which is a bit confusing.

Response: We thank the reviewer for this observation. As 5' scRNA-seq reads are inherently enriched at the 5' end of genes (**Figures R3.4** and **R3.5**), our original sashimi plots focused on these signal-rich regions. To better illustrate the ATS and ensure clarity, we have revised the **Fig. 2d** with curated transcript models. Specifically, we retained transcripts *201*, *203*, *204*, and *205* for *Emg1*, and transcript *201* for *Idh3g*.

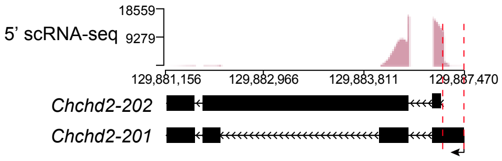

Figure R3.4. Sashimi plots showing the transcription level of the *Chchd2* gene in the 5' scRNA-seq dataset of mHSPCs (related to revised **Fig. 1f**). Corresponding dashed lines indicate the loci of predicted ATSs inferred by scATS. The full gene structures are shown below the sashimi plots.

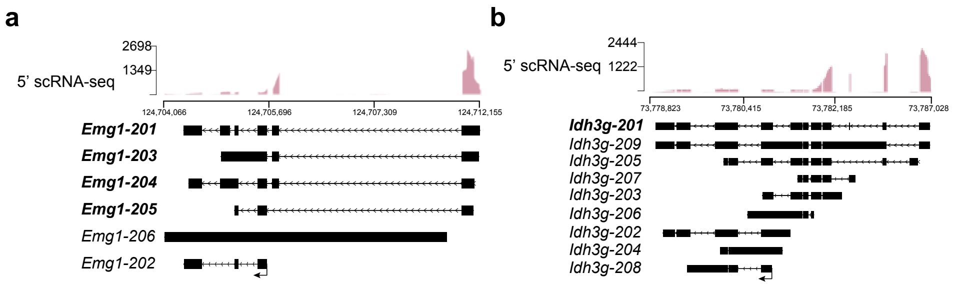

Figure R3.5 Sashimi plots showing the transcription level of the *Emg1* (a) and *Idh3g* (b) in the 5' scRNA-seq dataset of mHSPCs. The full gene structures and all known transcripts are shown below the sashimi plots, with the specific transcripts from revised **Fig. 2d** highlighted in bold.

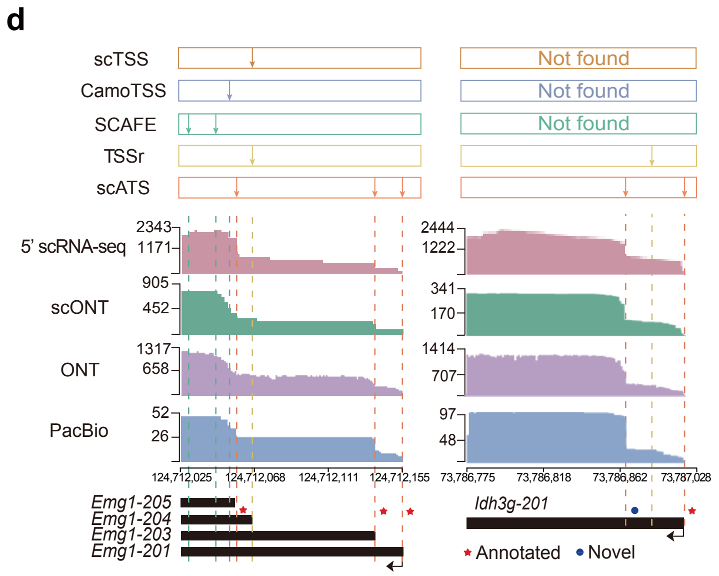

Revised ***Fig. 2d****, Sashimi plots showing the transcription level of Emg1 (left panel) and Idh3g (right panel) in the 5' scRNA-seq (pink), scONT (green), ONT (purple) and PacBio (blue) datasets of mHSPCs. Arrows (upper panels) and the corresponding dashed lines indicate the loci of predicted ATSs inferred by different methods. Annotation shown below the sashimi plots with annotated and novel TSSs indicated.*

We revised the 'Results' section as follows (Pg. 10, ln. 275-279):

> *"For the Emg1 gene, scATS identified three annotated TSSs in the 5' scRNA-seq dataset, all of which were validated by long-read datasets. Whilst TSSr and scTSS identified one annotated TSS that lacked long-read validation, the other methods incorrectly identified the ATSs."*

4. For figure 2b left, since all compared tools are predictive and do not represent a ground truth, the proximity of predictions from other software is not a sufficient validation of accuracy.

Response: We thank the reviewer for this comment. We agree that the comparison of predictive methods does not constitute independent validation, as no absolute ground truth is available. To avoid confusion, we have reorganised the figures. The left panel of the original **Fig. 2b** is now positioned alongside **Fig. 2a** as a descriptive comparison of tool concordance. The revised **Fig. 2b** focuses exclusively on validation against independent sources, specifically annotation databases and long-read sequencing datasets. This ensures that our validation results are supported by external evidence rather than inter-tool agreement.

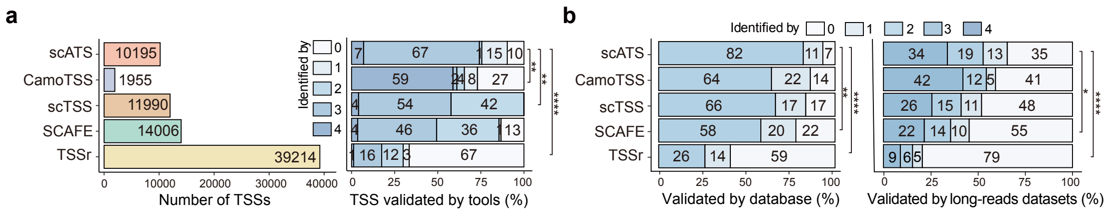

*Revised **Fig. 2a**, Bar plots showing the total number of TSSs (left) identified by scATS, CamoTSS, scTSS, SCAFE, and TSSr, and the proportion of TSSs (right) supported by at least one other method. The percentage of TSSs identified by different methods was assessed using a two-sided proportion test with BH correction; *, p < 0.05; **, p < 0.01; ****, p < 0.0001; comparisons without labels are not significant, unless stated otherwise.*
***b**, Validation of TSSs identified by different methods using independent evidence with a 50 bp distance threshold to define matched TSSs. Values indicate the number of supporting evidence sources (0, no support). Evidence includes annotation databases (GENCODE and refTSS; left), and long-read sequencing datasets (scONT, ONT, and PacBio; right).*

We revised the 'Results' section as follows (Pg. 8, ln. 231-240):

> *"TSSr, which is developed for bulk RNA-seq, identified most TSSs (n = 39,214). Three single-cell methods SCAFE (n = 14,006), scTSS (n = 11,990) and scATS (n = 10,195; **Supplementary Table 3**), reported comparable numbers of TSSs, and CamoTSS showed the lowest number of TSSs (n = 1,955) (**Fig. 2a**, left; see **Supplementary Note 4**). Next, we assessed the overlap among these five methods by calculating the percentages of TSSs supported by at least one other method. scATS (90%) has the highest overlap with other methods, followed by SCAFE (87%), CamoTSS (73%) and TSSr (33%). Note that scTSS was completely overlapped due to its reliance on the SCAFE and TSSr intersection and therefore was excluded in this comparison (**Fig. 2a**, right)."*

5. The manuscript concludes that "In general, distal TSSs exhibit more pronounced degradation than proximal ones". Have the authors identified any cases where the proximal ATS degradate more than the distal one?

Response: We thank the reviewer for raising this important point. To further quantify potential exceptions to the overall trend, we defined proximal ATSs as those located closer to the gene body than the most distal ATS. We found that 259 genes (18.8%) exhibit at least one proximal ATS with a higher degradation rate than that of the most distal ATS (revised **Extended Data Fig. 1c**). For example, *Mier1* is reported to be involved in early haematopoietic development through the regulation of haemogenic endothelium formation (Huang et al., 2013), and the proximal ATS (α = 0.51, β = 0.60) showed higher degradation than the distal ATS (α = 0.29, β = 0.75; revised **Extended Data Fig. 1d**).

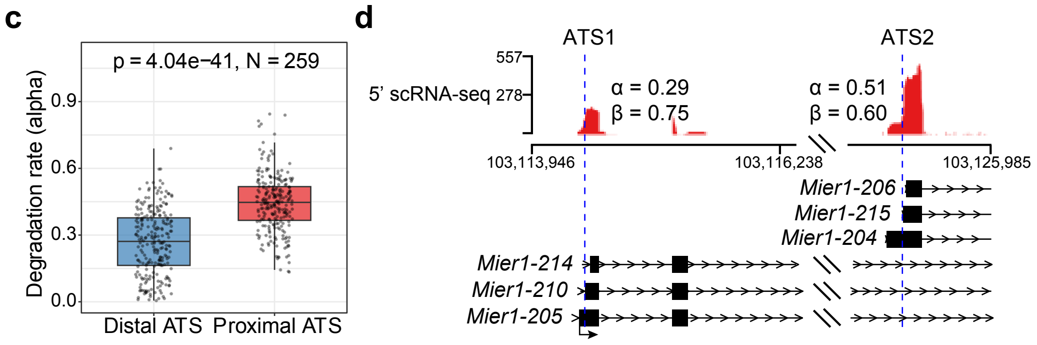

*Revised **Extended Data Fig. 1c,** Boxplots of RNA degradation rates at the pseudo-bulk level, genes with higher degradation at proximal ATSs.*
***d,** Sashimi plots showing the transcription level of Mier1 in the mHSPC 5' scRNA-seq dataset. The proximal ATS (ATS2) exhibits higher RNA degradation, resulting in a high α value of 0.51 and β value as low as 0.60. In contrast, the distal ATS (ATS1) shows mild degradation. The blue dashed lines indicate the loci of predicted TSSs inferred by scATS. GENCODE v25 annotations presented under the sashimi plots.*

We revised the 'Results' section as follows (Pg. 6-7, ln. 172-177):

> *"However, 259 genes (18.8%) exhibit at least one proximal ATS with a higher degradation rate than that of the most distal ATS (**Extended Data Fig. 1c**). Mier1, which is involved in early haematopoietic development through the regulation of haemogenic endothelium formation[28],*

showed higher degradation at the proximal ATS (α = 0.51, β = 0.60) than at the distal one (α = 0.29, β = 0.75; ***Extended Data Fig. 1d****).”*

Reviewer #3 (Remarks on code availability):

The installation from GitHub was unsuccessful. Furthermore, the package is incompatible with my R version 4.5.1. The documentation states that scATS was developed for R 4.0.0, it should maintain compatibility with more recent R releases. I recommend the authors update the dependencies to ensure support for current versions of the R environment.

Response: Thank you for pointing this out. To accommodate different environments, we have released scATS v0.5.6 (developed under R 4.5.2) (**Figure R3.6**), whilst v0.5.5 remains available for users with R versions earlier than 4.4. We have verified the successful installation of both versions in R 4.5.2 and R 4.0.5, and comprehensive installation logs for these environments are now provided on GitHub (https://github.com/LuChenLab/r-scATS/tree/main/logs) for reference.

Furthermore, we now offer multiple installation pathways and a Docker container to eliminate system-specific conflicts (**Figure R3.7**). The online documentation has been updated accordingly (https://r-scats.readthedocs.io/en/latest/md/Installation.html), and we welcome further technical discussions via our GitHub issue tracker (https://github.com/LuChenLab/r-scATS/issues).

```
R version 4.5.2 (2025-10-31)
Platform: x86_64-pc-linux-gnu
Running under: Ubuntu 22.04.5 LTS

Matrix products: default
BLAS:   /usr/lib/x86_64-linux-gnu/blas/libblas.so.3.10.0
LAPACK: /usr/lib/x86_64-linux-gnu/lapack/liblapack.so.3.10.0  LAPACK version 3.10.0

locale:
[1] LC_CTYPE=en_US.UTF-8       LC_NUMERIC=C
[3] LC_TIME=zh_CN.UTF-8        LC_COLLATE=en_US.UTF-8
[5] LC_MONETARY=zh_CN.UTF-8    LC_MESSAGES=en_US.UTF-8
[7] LC_PAPER=zh_CN.UTF-8       LC_NAME=C
[9] LC_ADDRESS=C               LC_TELEPHONE=C
[11] LC_MEASUREMENT=zh_CN.UTF-8 LC_IDENTIFICATION=C

time zone: Asia/Shanghai
tzcode source: system (glibc)

attached base packages:
[1] stats     graphics  grDevices utils     datasets  methods   base

other attached packages:
[1] scATS_0.5.6

loaded via a namespace (and not attached):
[1] RcppAnnoy_0.0.22            splines_4.5.2
[3] later_1.4.4                 BiocIO_1.20.0
[5] filelock_1.0.3              bitops_1.0-9
[7] tibble_3.3.0                R.oo_1.27.1
[9] polyclip_1.10-7             graph_1.88.1
[11] rpart_4.1.24                XML_3.99-0.20
[13] fastDummies_1.7.5           httr2_1.2.2
[15] lifecycle_1.0.4             OrganismDbi_1.52.0
[illegible]
```

Figure R3.6. Screenshot of the updated online documentation.

To install `scATS`, you have two options: either install directly from GitHub or use the compressed source file.

- Via GitHub (only for `v0.5.6`):

```
if (!require("devtools")) install.packages("devtools")
devtools::install_github("LuChenLab/r-scATS", force = TRUE)

# or
if (!require("remotes")) install.packages("remotes")
remotes::install_github("LuChenLab/r-scATS")
```

- Via Source File (for `v0.5.6` and `v0.5.5`):

Alternatively, you can install `scATS` using the source file downloaded from the repository :

```
# Install scATS from a downloaded source file
R CMD INSTALL scATS_0.5.6.tar.gz
```

```
# or
install.packages("scATS_0.5.6.tar.gz", repos=NULL)
```

Installation Logs

To ensure reproducibility, we provide comprehensive installation logs for three different installation methods across multiple R versions:

- Installation Logs for R 4.5.x
- Installation Logs for R 4.0.x

Docker Guide

We provide a pre-configured Docker container to eliminate system-specific dependency conflicts.

- Step 1: Install Docker

Figure R3.7. Screenshot of the updated GitHub repository.

Reviewer #4 (Remarks to the Author):

I have reviewed rebuttal and thank the authors for addressing all my concerns. From my view each point was carefully addressed and considerable attention to detail provided for the technical aspects. I believe the additional figures, text and information added has improved manuscript.

The only remaining concern I have is that I had issues installing r-scATS software. Details below

Issue 1: Instructions for installation state to run following command which causes an error:

remotes::install_github("LuChenLab/r-scATS/scATS/")

This should be modified to

remotes::install_github("LuChenLab/r-scATS")

Response: Thank you for the observation. The erroneous command has been corrected in the GitHub repository (**Fig. R4.1**) and now runs as expected. The online documentation (https://r-scats.readthedocs.io/en/latest/md/Installation.html) has also been updated for clarity.

Installation

To install `scATS`, you have two options: either install directly from GitHub or use the compressed source file.

- Via GitHub (only for `v0.5.6`):

```
if (!require("devtools")) install.packages("devtools")
devtools::install_github("LuChenLab/r-scATS", force = TRUE)

# or
if (!require("remotes")) install.packages("remotes")
remotes::install_github("LuChenLab/r-scATS")
```

- Via Source File (for `v0.5.6` and `v0.5.5`):

Alternatively, you can install `scATS` using the source file downloaded from the repository :

```
# Install scATS from a downloaded source file
R CMD INSTALL scATS_0.5.6.tar.gz
```

```
# or
install.packages("scATS_0.5.6.tar.gz", repos=NULL)
```

Installation Logs

To ensure reproducibility, we provide comprehensive installation logs for three different installation methods across multiple R versions:

- Installation Logs for R 4.5.x
- Installation Logs for R 4.0.x

Figure R4.1. Screenshot of the updated GitHub repository.

Issue 2: After navigating and resolving issue1 I next obtain the following installation error:

Error in loadNamespace(j <- i[[1L]], c(lib.loc, .libPaths()), versionCheck = vI[[j]]) :

there is no package called 'smoother'

Looking into this library I note that the following link states that this library has been "Archived on 2025-12-19 as issues were not corrected despite reminders"

https://cran.r-project.org/web/packages/smoother/index.html

This will be an issue for any person trying to install for the first time.

Response: We thank the reviewer for highlighting this issue. To ensure scATS remains accessible following the archival of the 'smoother' package, we have implemented the following three strategies:

1. Code Update (scATS v0.5.6): To ensure long-term stability, we have rewritten the functions previously provided by the 'smoother' package directly into the scATS source code. Consequently, the 'smoother' package is no longer required for version 0.5.6, which is fully optimised for R 4.4 and above.
2. Manual Installation Workaround: For users who prefer to maintain their current environment, the 'smoother' package can still be manually installed from the CRAN Archive (**Figure R4.2**). Once this dependency is met, scATS will install without error.
3. Docker Environment: We provide a pre-configured Docker container that includes all necessary dependencies (**Figure R4.3**), ensuring a stable and functional environment for all users regardless of local system changes.

We have updated our installation guide (https://r-scats.readthedocs.io/en/latest/md/Installation.html) to reflect these options and welcome any further technical discussions or feedback via our GitHub issue tracker (https://github.com/LuChenLab/r-scATS/issues).

Requirements

Please choose the appropriate version of `scATS` based on your R environment:

- `v0.5.6` (Recommended): For users with R >= 4.4 (developed with `R 4.5.2`).
- `v0.5.5` (Legacy): For users with R < 4.4 (developed under `R 4.0.0`).

1. System Dependencies

Before installing scATS, please ensure that `samtools` is installed and available in your system's PATH.

```
conda install -c bioconda samtools
```

2. R Dependencies

The following R packages are required for all versions of scATS. You can install them using the following command:

```
install.packages(c(
    'R.utils',
    'TTR',
    'VGAM',
    'prettyunits',
    'httr2',
    'mclust',
    'mixtools'))
```

> **Note:** 💡 For v0.5.5, you must manually install the smoother package from the CRAN archive before installing scATS:

```
install.packages('https://cran.r-project.org/src/contrib/Archive/smoother/smoother_1.3.tar.gz',
```

Figure R4.2. Screenshot of the updated requirements on GitHub.

Docker Guide

We provide a pre-configured Docker container to eliminate system-specific dependency conflicts.

- Step 1: Install Docker

If you do not have Docker installed, please follow the official Docker installation guide.

- Step 2: Run the scATS Container

Pull the pre-built image from the GitHub Container Registry and start an interactive session:

```
# Pull the image
docker pull ghcr.io/kayla-xu/scats-evi:r451

# Run the container
docker run -it ghcr.io/kayla-xu/scats-evi:r451
```

- Step 3: Build Docker Locally (Optional)

If you prefer to build the image locally, use the provided Dockerfiles. Building logs are also provided for your reference.

```
# Build the base environment
docker build --no-cache \
  -t scATS/base:r451 \
  -f Dockerfile_base_r451 . \
  2>&1 | tee Dockerfile_base_r451.log

# Build the final scATS image
docker build --no-cache \
  -t scATS/evi:r451 \
  -f Dockerfile_scats_r451 . \
  2>&1 | tee Dockerfile_scats_r451.log
```

Figure R4.3. Screenshot of the updated docker guide on GitHub.

Reviewer #4 (Remarks on code availability):

I can confirm github repository is accessible. I have issues installing software as mentioned above. This will need to be resolved as software cannot be installed until library becomes available.

Response: We thank the reviewer for highlighting these installation concerns. To resolve the issue regarding the 'smoother' package, we have released scATS v0.5.6, which incorporates the required functions directly into the source code to ensure compatibility.

Furthermore, our updated online documentation now provides comprehensive installation logs for reference, alongside a Docker environment for direct use to facilitate a stable setup across diverse systems (https://r-scats.readthedocs.io/en/latest/md/Installation.html). We believe these updates resolve the mentioned library availability issues and remain available to provide further support via our GitHub issue tracker (https://github.com/LuChenLab/r-scATS/issues).

References

Bray, N. L., Pimentel, H., Melsted, P., & Pachter, L. (2016). Near-optimal probabilistic RNA-seq quantification. *Nature Biotechnology 2016 34:5*, *34*(5), 525–527. https://doi.org/10.1038/nbt.3519

Chen, Y., Sim, A., Wan, Y. K., Yeo, K., Lee, J. J. X., Ling, M. H., Love, M. I., & Göke, J. (2023). Context-aware transcript quantification from long-read RNA-seq data with Bambu. *Nature Methods 2023 20:8*, *20*(8), 1187–1195. https://doi.org/10.1038/s41592-023-01908-w

Fahmi, N. A., Saha, S., Song, Q., Lou, Q., Yong, J., & Zhang, W. (2025). Computational methods for alternative polyadenylation and splicing in post-transcriptional gene regulation. *Experimental & Molecular Medicine 2025 57:8*, *57*(8), 1631–1640. https://doi.org/10.1038/s12276-025-01496-z

Fan, F., Gao, J., Zhao, Y., Wang, J., Meng, L., Ma, J., Li, T., Han, H., Lai, J., Gao, Z., Li, X., Guo, R., Cao, Z., Zhang, Y., Zhang, X., & Chen, H. (2023). Elevated Mast Cell Abundance Is Associated with Enrichment of CCR2+ Cytotoxic T Cells and Favorable Prognosis in Lung Adenocarcinoma. *Cancer Research*, *83*(16), 2690–2703. https://doi.org/10.1158/0008-5472.CAN-22-3140,

Fu, S., & Li, W. V. (2025). Predicting and comparing transcription start sites in single cell populations. *PLOS Computational Biology*, *21*(4), e1012878. https://doi.org/10.1371/JOURNAL.PCBI.1012878

Hou, R., Hon, C. C., & Huang, Y. (2023). CamoTSS: analysis of alternative transcription start sites for cellular phenotypes and regulatory patterns from 5' scRNA-seq data. *Nature Communications*, *14*(1). https://doi.org/10.1038/s41467-023-42636-1

Huang, H. T., Kathrein, K. L., Barton, A., Gitlin, Z., Huang, Y. H., Ward, T. P., Hofmann, O., Dibiase, A., Song, A., Tyekucheva, S., Hide, W., Zhou, Y., & Zon, L. I. (2013). A network of epigenetic regulators guides developmental haematopoiesis in vivo. *Nature Cell Biology 2013 15:12*, *15*(12), 1516–1525. https://doi.org/10.1038/ncb2870

Kovaka, S., Zimin, A. V., Pertea, G. M., Razaghi, R., Salzberg, S. L., & Pertea, M. (2019). Transcriptome assembly from long-read RNA-seq alignments with StringTie2. *Genome Biology*, *20*(1). https://doi.org/10.1186/s13059-019-1910-1

Li, B., & Dewey, C. N. (2011). RSEM: accurate transcript quantification from RNA-Seq data with or without a reference genome. *BMC Bioinformatics*, *12*. https://doi.org/10.1186/1471-2105-12-323

Lin, L., Yang, S., Li, X., Zhang, W., & Zheng, J. (2025). Unveiling the role of Pafah1b3 in liver fibrosis: A novel mechanism revealed. *Journal of Pharmaceutical Analysis*, *15*(1), 101158. https://doi.org/10.1016/j.jpha.2024.101158

Lu, Z., Berry, K., Hu, Z., Zhan, Y., Ahn, T. H., & Lin, Z. (2021). TSSr: an R package for comprehensive analyses of TSS sequencing data. *NAR Genomics and Bioinformatics*, *3*(4). https://doi.org/10.1093/NARGAB/LQAB108

Moody, J., Kouno, T., Chang, J. C., Ando, Y., Carninci, P., Shin, J. W., & Hon, C. C. (2022). SCAFE: a software suite for analysis of transcribed cis-regulatory elements in single cells. *Bioinformatics (Oxford, England)*, *38*(22), 5126–5128. https://doi.org/10.1093/BIOINFORMATICS/BTAC644

Moro, G., Brunner, E., & Basler, K. (2026). A practical guide to targeted single-cell RNA sequencing technologies. *Communications Biology 2026 9:1*, *9*(1), 250-. https://doi.org/10.1038/s42003-026-09675-y

Nguyen, Q. A., Wang, H., Verghese, E., Iscaro, J., Steinfort, D., McQualter, J., & Bozinovski, S. (2025). Identification of C1QA as a prognostic marker and regulator of immunosuppressive neutrophils in early-stage lung adenocarcinoma through integrated bioinformatics analyses. *Clinical and Experimental Medicine*, *25*(1). https://doi.org/10.1007/s10238-025-01881-y

Patro, R., Duggal, G., Love, M. I., Irizarry, R. A., & Kingsford, C. (2017). Salmon provides fast and bias-aware quantification of transcript expression. *Nature Methods 2017 14:4*, *14*(4), 417–419. https://doi.org/10.1038/nmeth.4197

Prjibelski, A. D., Mikheenko, A., Joglekar, A., Smetanin, A., Jarroux, J., Lapidus, A. L., & Tilgner, H. U. (2023). Accurate isoform discovery with IsoQuant using long reads. *Nature Biotechnology 2023 41:7*, *41*(7), 915–918. https://doi.org/10.1038/s41587-022-01565-y

Zhan, Y., Hu, Z., Lu, Z., & Lin, Z. (2025). The patterns of alternative TSS usage explain the highly heterogeneous landscape of 5′UTR lengths in eukaryotes. *NAR Genomics and Bioinformatics*, *7*(4), lqaf152. https://doi.org/10.1093/nargab/lqaf152

Point-by-point response

MS title: Accurate profiling of single-cell alternative transcript start sites by correcting RNA degradation

Dear reviewers:

We thank the reviewers for their valuable comments and suggestions. In response to the comments, we performed additional analysis and experiments, revised the manuscript (the changes are shown in red font) and updated the accompanying figures and tables. We addressed the concerns point-by-point under each comment. The changes introduced into the revised manuscript (MS) are shown in *italics*, with the corresponding page (Pg.) and line (ln.) numbers indicated.

Yours sincerely,

Lu Chen, PhD

Reviewer #3 (Remarks to the Author):

The authors have addressed most of the concerns from the previous review. The Sashimi plots are now clearer with the exon structures, and the enrichment analysis has been further clarified. Additionally, the quantification of distal and proximal TSS degradation is much more precise compared to the previous general description. Overall, the manuscript is good to me now.

Response: Thank you for your encouraging remarks. We are delighted that our revisions to the analysis and visualisations have successfully addressed your concerns.

Reviewer #3 (Remarks on code availability):

The updated online documentation and installation instructions look good to me.

Response: We appreciate your feedback. The refined manuals and setup procedures meet your standards.
